# π‐Extended Salphen Scaffolds Enable CO_2_ Electroreduction and Singlet Oxygen Generation

**DOI:** 10.1002/chem.71385

**Published:** 2026-07-12

**Authors:** Vincenzo Langellotti, André Korzun, Morgan J. McKee, Massimo Melchiorre, Taegeun Jo, J. Felipe Estrada‐Ruiz, Gregor Schnakenburg, Stefano Crespi, Francesco Ruffo, Nikolay Kornienko, Alessandro Bismuto

**Affiliations:** ^1^ Department of Chemical Sciences, University of Naples Federico II University Campus of Monte S. Angelo Naples Italy; ^2^ Institute of Inorganic Chemistry University of Bonn Bonn Germany; ^3^ Department of Chemistry–Ångström Laboratory Uppsala University Uppsala Sweden

**Keywords:** catalysis, CO_2_ Electroreduction, salphen ligands, singlet oxygen generation, *π*‐extended System

## Abstract

π‐Extended conjugation offers the opportunity to unveil new frontiers in materials, coupling redox activity with tunable photophysical properties. Here, we report a new family of π‐conjugated salen ligands bearing phenylene bridges and tunable substituents, together with their corresponding boron difluoride complexes. Owing to the extended π‐delocalization, these new compounds show promising results in electrochemical CO_2_ reduction to CO, achieving a remarkable Faradaic efficiency for an organocatalyst (up to 56%). Electrochemical, spectroscopic, and computational analyses support a ligand‐centered mechanism in which single‐electron reduction activates the framework for CO_2_ binding at the imine nitrogen, forming an N‐bound CO_2_H intermediate. Beyond electrocatalysis, coordination to boron rigidifies the π‐framework, producing emissive boron‐salphen complexes with large Stokes shifts (>7000 cm^−^^1^). These complexes function as efficient photosensitizers for singlet‐oxygen generation, enabling mild oxidative cleavage of alkenes under blue‐light irradiation. This work demonstrates that π‐extension transforms the traditional salphen framework into a redox‐active engine for small‐molecule activation, bypassing the need for transition metals in CO_2_ electroreduction while simultaneously unlocking good photosensitizing capabilities.

## Graphical abstract

New family of π‐extended salen ligands bearing phenylene bridge and tunable phenyl‐based substituents, and their corresponding boron difluoride complexes. Thanks to the extended π‐delocalization over the molecule, the ligands themselves show promising results in electrochemical CO_2_ reduction to CO, while the complexes act as efficient photosensitizers for singlet‐oxygen generation.

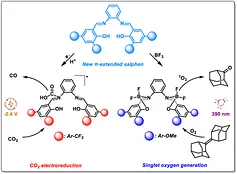

## 1 Introduction

Salen‐type ligands represent one of the most versatile scaffolds in coordination chemistry and homogeneous catalysis. Their modular ONNO dianionic donor framework and structural tunability, enabled by the substitution of the phenolate rings and variation of the diamine backbone have contributed to the broad success of this scaffold across several fields [1, 2]. Classic examples, with transition metals, include Mn(salen)‐catalyzed asymmetric epoxidation (Jacobsen–Katsuki epoxidation), Cr(salen)‐mediated ring opening of epoxides, and Co(salen) complexes used in hydrolytic kinetic resolution and radical processes (Figure 1A) [3, 4, 5, 6, 7, 8, 9, 10, 11, 12, 13, 14].

**FIGURE 1:**
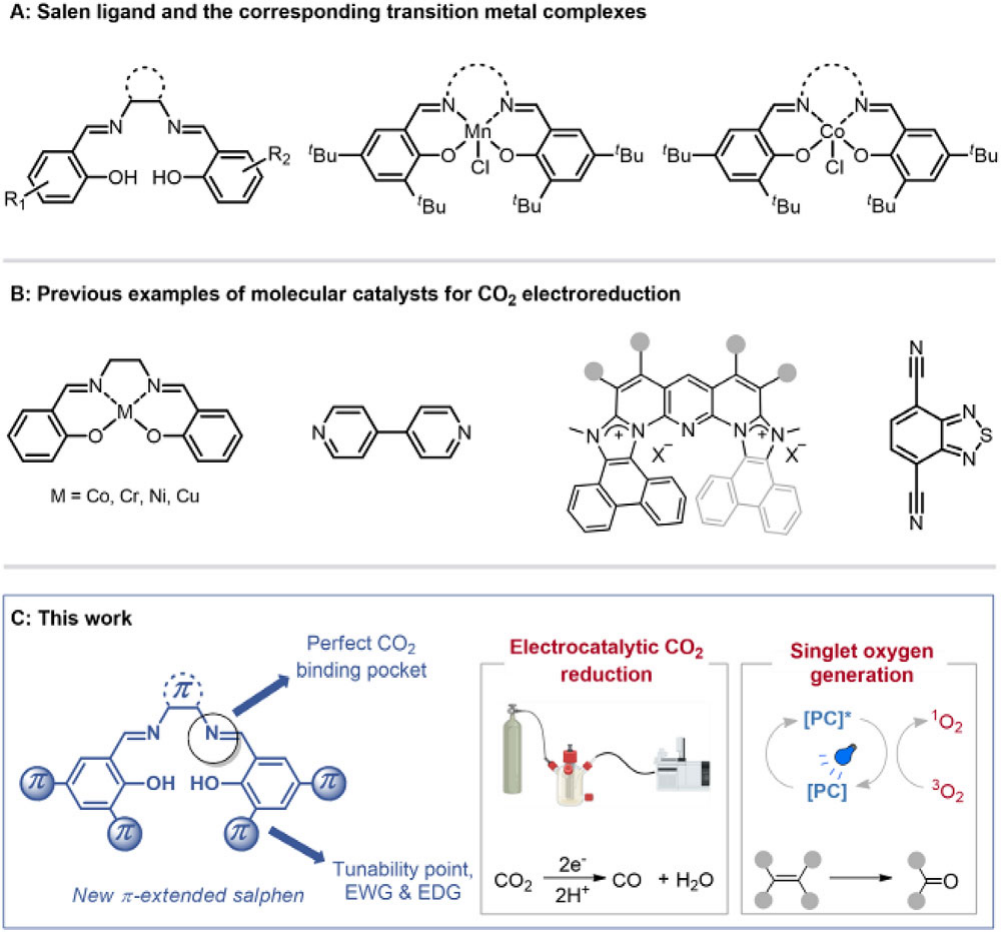
(A) Salen ligand and corresponding transition metal complexes. (B) Previous examples of molecular catalysts for CO_2_ electroreduction. (C) This work: new π‐extended salphen scaffolds and their applications.

More recently, Ni, Mn, and Co salen complexes have also been utilized in electrocatalytic transformations such as CO_2_ and H_2_ generation. This is a flourishing field, as the CO_2_ utilization and conversion into intermediates (*e.g*., CO, methanol) and energy carriers (*e.g*., formic acid, CH_4_) not only provides high‐value compounds, but also contributes to the sustainable development goals within the climate action for the decrease of atmospheric CO_2_ [15, 16]. However, the kinetic inertness of CO_2_ requires the use of a sophisticated catalytic system [17]. Most of the reported catalysts for the CO_2_ reduction rely on metals as discrete molecules as well as materials (*e.g*., NPs, MOFs). A notable example is a Cr‐ONNO catalyst paired with π‐extended redox mediators (RMs), which achieved faster CO formation at lower overpotentials than analogous RMs with smaller π‐systems [18]. There, the aromatic extension of RMs promotes critical association not only through electrostatic interactions but also *via* dispersion forces and π‐system effects (*e.g*., through‐space conjugation, SOMO–LUMO interactions, and pancake bonding) [19, 20, 21].

Within the same field, metal‐free molecular catalysts based on organic molecules such as pyridinium salts, quinones, aromatic imides, and other π‐conjugated scaffolds (Figure 1B) have recently emerged as promising platforms for CO_2_ activation and reduction [22, 23]. These compounds typically interact with carbon dioxide through lone‐pair donation from oxygen or nitrogen atoms, or *via* π–π interactions with their conjugated frameworks, enabling CO_2_‐to‐formate or CO conversion through either direct electron transfer or redox‐cycling pathways [24, 25, 26]. Chean and Tian, for instance, have demonstrated that 2,1,3‐benzothiadiazole and its 4,7‐dicarbonitrile derivative can mediate the electroreduction of CO_2_ and other small molecules [27]. Alternatively, a more elaborate scaffold, a dicationic helicene, has been reported for CO_2_‐to‐formate reduction, operating as an efficient hydride‐shuttling catalyst under electrochemical conditions in the presence of a proton source [28]. This system exhibited improved TON (up to 17.5) and Faradaic efficiency up to 35%. These advances underscore the crucial role that ligand architecture and secondary interactions play in facilitating efficient small‐molecule activation under electrochemical conditions. *However, comparatively little attention has been paid to how electronic delocalization across the ligand framework itself can influence the activation of small molecules and promote catalysis*. Inspired by strategies in main‐group chemistry, where π‐conjugation and electronic delocalization have frequently been employed to tailor redox and optical properties [29], we sought to revisit the salen framework beyond its traditional role as a supporting ligand and instead transform it into a redox‐active molecular platform. Herein, we report a new family of π‐extended salen‐type ligands incorporating phenylene bridges and conjugated aromatic units (namely *salphen*), with different substituents, in *ortho* and *para* positions of the aryl units (Figure 1C), along with their corresponding dinuclear boron complexes. This enhanced π‐delocalization has critically impacted the performance in both electrocatalysis and singlet oxygen generation, transforming the classical salen scaffold into a multifunctional platform for redox and photochemical reactivity.

## 2 Results and Discussion

To evaluate this design strategy, we synthesized a series of π‐extended salphen ligands bearing electronically diverse substituents with both electron‐withdrawing and electron‐donating groups, using modified procedures from the literature (see SI for details, compounds **1–4**) [30, 31]. The new scaffolds (**5–8**) were accessed in good yields by condensation of the appropriate aldehyde with 1,2‐phenylendiamine (Scheme 1 and Supporting Information for more details) [32].

**SCHEME 1:**
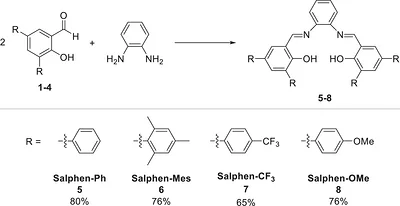
Synthesis of π‐conjugated salphen ligands.

Single‐crystal X‐ray diffraction analysis confirmed the identity of these compounds. The solid‐state structure of compound **7** is C_1_‐symmetric and, as commonly observed for salphen compounds [33], with one phenol ring virtually coplanar with the 1,2‐diaminobenzene unit and the other one twisted out of plane (see Supporting Information). The imine bond C═N (1.280(2) Å) is longer than the reference molecule, due to the presence of an electron‐withdrawing group, a trend shown for salen ligands and amplified by the salphen moiety [34, 35, 36, 37, 38].

In order to better understand the electronic features of these compounds, we recorded absorbance and emission spectra. All compounds displayed two or three absorption bands, with the major one centred at ∼280 nm, typical of the salen scaffold and attributed to π–π* transitions [37], and the others in the range 350–400 nm, depending on the type of substituent (see Figures S30–S33 and S42–S45). To compare the influence of π‐extension on the electronic structure, we computed both salphen and salen derivatives (Figure S58). The HOMO and LUMO of salphen derivatives are generally well delocalized over the entire backbone, indicating efficient π‐conjugation across the ligand framework (Figure 2). In contrast, although salen derivatives also exhibit π‐type orbitals, their delocalization is more limited and does not extend across the full molecular backbone. In agreement with the observed orbital distribution, the calculated HOMO‐LUMO energy gaps (Table S11) for the salphen derivatives are consistently smaller than the corresponding salen analogues. Further support was found in the redox properties of salphen‐CF_3_ where the calculated half‐wave potential (−2.35 eV vs Fc/Fc^+^) is significantly less negative than that of the corresponding salen derivative (−2.83 eV vs Fc/Fc^+^). This discrepancy indicates that the salphen framework is more delocalized *via* π‐extension, stabilizing the reduced species by distributing the added electron density throughout the conjugated backbone.

**FIGURE 2:**
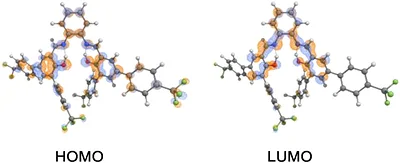
HOMO and LUMO orbitals of compound **7** obtained at the B97‐3c level of theory at an iso‐value of 0.05 a.u.

Once we analyzed the electronic structure through theory, we decided to investigate it further using electrochemistry. Cyclic voltammetry measurements (CVs) of salphen ligands showed the formation of two or three redox events at negative potentials (as an example, CV of compound 7 is reported in Figure 3A and the others in Supporting Information), depending on the nature of the substituent. One of these redox events is reversible and generally appears at ∼ −2.25 V, while the other one or two are irreversible. A similar pattern can also be observed at positive potentials, with one reversible redox event at ∼ 0.75 V, and others irreversible. These CVs are characteristic of the metal salen scaffold [38]. CVs measurements of π‐salphen ligands were also performed after saturation with CO_2_ (Figure 3A, see Supporting Information for further details), with unaltered redox features compared to Ar. However, performing the same CV measurement under CO_2_ saturation led to a pronounced increase in cathodic current, which is consistent with an interaction with carbon dioxide [39]. This current enhancement typically emerges at potentials more negative than approximately −2.0 V, with varying magnitudes depending on ligand structure. Thrilled by this latest observation, we decided to investigate further the interaction with CO_2_.

**FIGURE 3:**
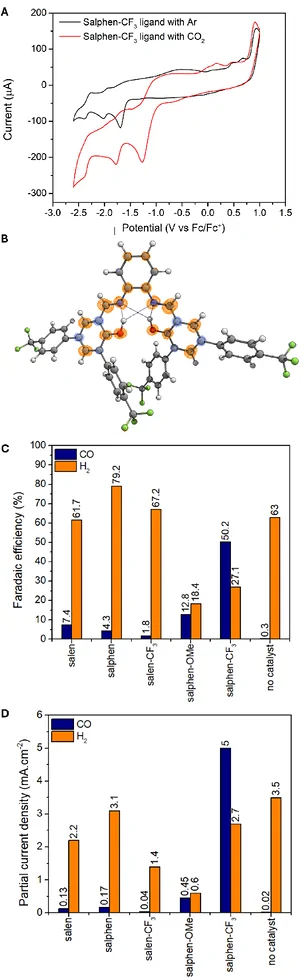
(A) Cyclic voltammogram of 1.0 mM **salphen‐CF_3_** in acetonitrile with 0.10 M TBAPF_6_ supporting electrolyte at a scan rate of 100 mV.s^−1^, with argon (black line) or CO_2_ (red line) atmosphere. (B) Calculated spin density distribution of singly reduced **salphen‐CF_3_** at the B97‐3c level with SMD (MeCN). (C) Faradaic efficiency and (D) partial current density for CO and H_2_ production using 1.0 mM of different ligands at −2.4 V vs Fc/Fc^+^.

Firstly, we sought to compare a series of salen‐ and salphen‐derived ligands, which revealed a pronounced dependence of CO_2_ reduction activity on ligand structure. Under identical conditions, the CO Faradaic efficiency follows the order **salphen‐CF_3_** > **salphen‐OMe** > **salen** > **salphen** > **salen‐CF_3_** (**7'**), with **salphen‐CF_3_** achieving approximately 50% CO selectivity (Figure 3C, D). This trend highlights the importance of combining extended π‐conjugation with electron‐withdrawing substituents to achieve efficient CO_2_ reduction. While increased conjugation alone is insufficient to ensure high activity, the presence of electron‐withdrawing groups appears to stabilize catalytically relevant reduced ligand states (Table S12). Indeed, this hypothesis is confirmed by computing the spin density of the singly‐reduced **salphen**‐**CF_3,_** which is spread over the entire conjugated system (Figure 3B). Thus, demonstrating that π‐extension in the salphen scaffold enhances electronic delocalization and significantly changes its redox properties, something that was only achieved before by the addition of a transition metal to this scaffold [39]. On this ground, the electrochemical CO_2_ reduction mediated by the metal‐free **salphen‐CF_3_** ligand was investigated in MeCN containing 0.10 M TBAPF_6_, using water as a proton source (Figure 4A). Under CO_2_, the system selectively produces CO as the only carbon‐containing product, with H_2_ identified as the main secondary product. No CO formation was detected under open‐circuit conditions, in the absence of catalyst, or under Ar, confirming that CO generation arises from a catalyst‐mediated CO_2_ reduction pathway (Figure S18). At 5% H_2_O, CO Faradaic efficiency (FE) increases with applied potential to a maximum of 31% at −2.4 V, followed by a decline at more negative potentials.

**FIGURE 4:**
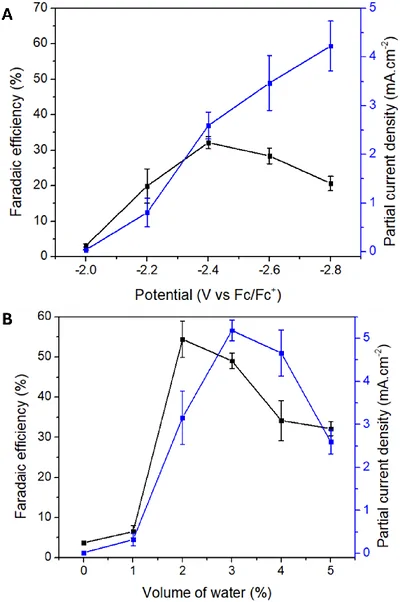
(A) FE and PCD for CO_2_ electroreduction using 1.0 mM **salphen‐CF_3_** in MeCN with 0.10 M TBAPF_6_ and 5% v/v water. (B) FE and PCD as a function of water content (1%–5% v/v).

In contrast, the partial current density (PCD) for CO (j_CO_) increases as more negative potentials are applied, reaching 4.8 mA.cm^−2^ at −2.8 V. This behavior suggests that increasing overpotential primarily increases total current rather than selectively enhancing CO production. We next sought to increase the CO production by a systematic variation of water content, revealing a dependence on both activity and selectivity on proton availability (Figure 4B). Decreasing the water concentration from 5% to 3% enhances CO formation, yielding a maximum CO FE of 50% and a j_CO_ of 5.0 mA.cm^−2^ at −2.4 V. FE was further maximized by reducing the water content to 2%, reaching a peak FE of 56%, albeit at lower current density. As 3% water provides the optimal balance between selectivity and activity, this condition was employed for subsequent studies. Catalyst concentration dependence shows that j_CO_ exhibits a maximum at 1.0 mM **salphen‐CF_3_**, while CO FE remains constant across the concentration range tested at −2.4 V, suggesting saturation of the CO‐forming pathway under these conditions. Long‐term electrolysis was performed at −2.4 V in the presence of 3% water, affording an overall CO Faradaic efficiency of 41% and reaching a turnover number of 103 over the course of 18 h. CO formation was sustained throughout electrolysis, indicating stable catalytic turnover under these conditions with the integrity of catalyst structure being confirmed by UV–vis spectroscopy (see Figure S26).

Alternative proton sources were also examined to assess how proton delivery influences CO_2_ reduction mediated by **salphen‐CF_3_** (see Supporting Information). Trifluoroethanol affords the highest CO selectivity, reaching Faradaic efficiencies of up to 90%, but supports only very low partial current densities (0.08 mAcm^−2^), indicating limited catalytic turnover under these conditions. In contrast, water enables substantially higher CO partial current densities (up to 5 mAcm^−2^) while maintaining moderate to high selectivity, demonstrating a clear trade‐off between CO selectivity and catalytic activity depending on the proton donor. Acetic acid provides only low CO activity and selectivity, and phenol shows no detectable CO formation, highlighting that simple acidity alone is insufficient to promote productive CO_2_ reduction in this system. Notably, no clear correlation between proton donor acidity and catalytic current density is observed, indicating that factors beyond simple Brønsted acidity are responsible for proton delivery in this system [40]. All proton sources were evaluated over a concentration range of 1%–5% v/v, and water consistently delivered the most favorable balance between CO FE and PCD. Consequently, water was selected as the proton source for subsequent mechanistic and spectroscopic investigations. These observations are consistent with trends reported for metal‐based CO_2_ reduction catalysts, where electron‐withdrawing ligand environments similarly enhance CO_2_ activation and catalytic performance [41]. Analysis of product selectivity across the ligand series reveals a clear inverse correlation between CO and H_2_ formation. Under identical conditions, ligands exhibiting low CO Faradaic efficiencies, such as **salen**, **salphen**, and **salen‐CF_3_**, display high H_2_ selectivity (62%–79%), whereas **salphen‐CF_3_**, which affords approximately 50% CO FE, produces substantially less H_2_ (27%). **Salphen‐OMe** shows comparatively low H_2_ formation (18%) but only modest CO selectivity, indicating that suppression of proton reduction alone is insufficient to promote efficient CO_2_ reduction. Control experiments conducted in the absence of catalyst show significant H_2_ production on bare glassy carbon (PCD = 3.5 mA.cm^−2^, FE = 68%), suggesting that hydrogen evolution largely originates from the electrode surface under these conditions. These trends demonstrate that ligand structure plays a crucial role in selectivity between CO_2_ reduction and proton reduction, consistent with a ligand‐centered mechanism in which stabilization of CO_2_‐derived intermediates diverts reactivity away from H_2_ evolution. The observed activity is notable given the complete absence of a metal center and the operation of a ligand‐centered mechanism.

To probe the nature of CO_2_‐derived intermediates formed during catalysis, in situ FTIR spectroscopy (cell scheme in Figure 5A) was performed under electrochemical CO_2_ reduction conditions using **salphen‐CF_3_** in MeCN containing TBAPF_6_ and 3% H_2_O at a range of potentials. Under reductive conditions, three distinct features appear in the carbonyl stretching region at 2099, 2045, and 1935 cm^−1^, whose intensities increase with applied potential before reaching a plateau at approximately −2.4 V (Figure 5B). In addition, a prominent band at 1391 cm^−1^ emerges and grows continuously at more negative potentials. No new vibrational features are observed when the experiment is performed under Ar (Figure S27), confirming that these bands are associated with CO_2_‐derived processes. Isotopic labelling experiments using ^13^CO_2_ resulted in a clear red‐shift of the 1391 cm^−1^ band to 1361 cm^−1^, supporting assignment of this feature to a CO_2_‐derived carbamate species (Figure S28). In contrast, the CO‐region bands are not observed under the ^13^CO_2_ conditions employed, which is attributed to differences in experimental configuration, as the ^13^CO_2_ experiments were conducted under electrolyte saturation rather than continuous gas purging. The appearance of multiple CO‐region bands under ^12^CO_2_ electrolysis indicates the formation of CO‐derived intermediates with different degrees of electronic stabilization by the reduced **salphen‐CF_3_** ligand. The relative intensities of these features, with the 1935 cm^−1^ band being dominant, suggested that strongly stabilized CO species are preferentially populated under catalytic conditions. The progressive red‐shift across the CO bands is consistent with increasing electron density as a function of applied potential. In classical metal‐CO systems, such shifts are typically attributed to increased metal‐to‐CO π‐back bonding or bridging moieties. However, in the absence of a metal center in the present system, the observed behavior instead suggests ligand‐mediated stabilization of the CO intermediate. To further probe the catalytic mechanism, kinetic isotope effect (KIE) experiments (Figure S29) were performed at −2.4 V versus Fc/Fc^+^ using 3% H_2_O and D_2_O as proton sources. A significant decrease in the CO partial current density was observed in D_2_O, corresponding to a KIE value of 2.06. This result indicates that proton transfer is involved in the rate‐determining step of the catalytic process and is consistent with a proton‐coupled electron transfer pathway within the proposed ligand‐centered CO_2_ reduction mechanism.

**FIGURE 5:**
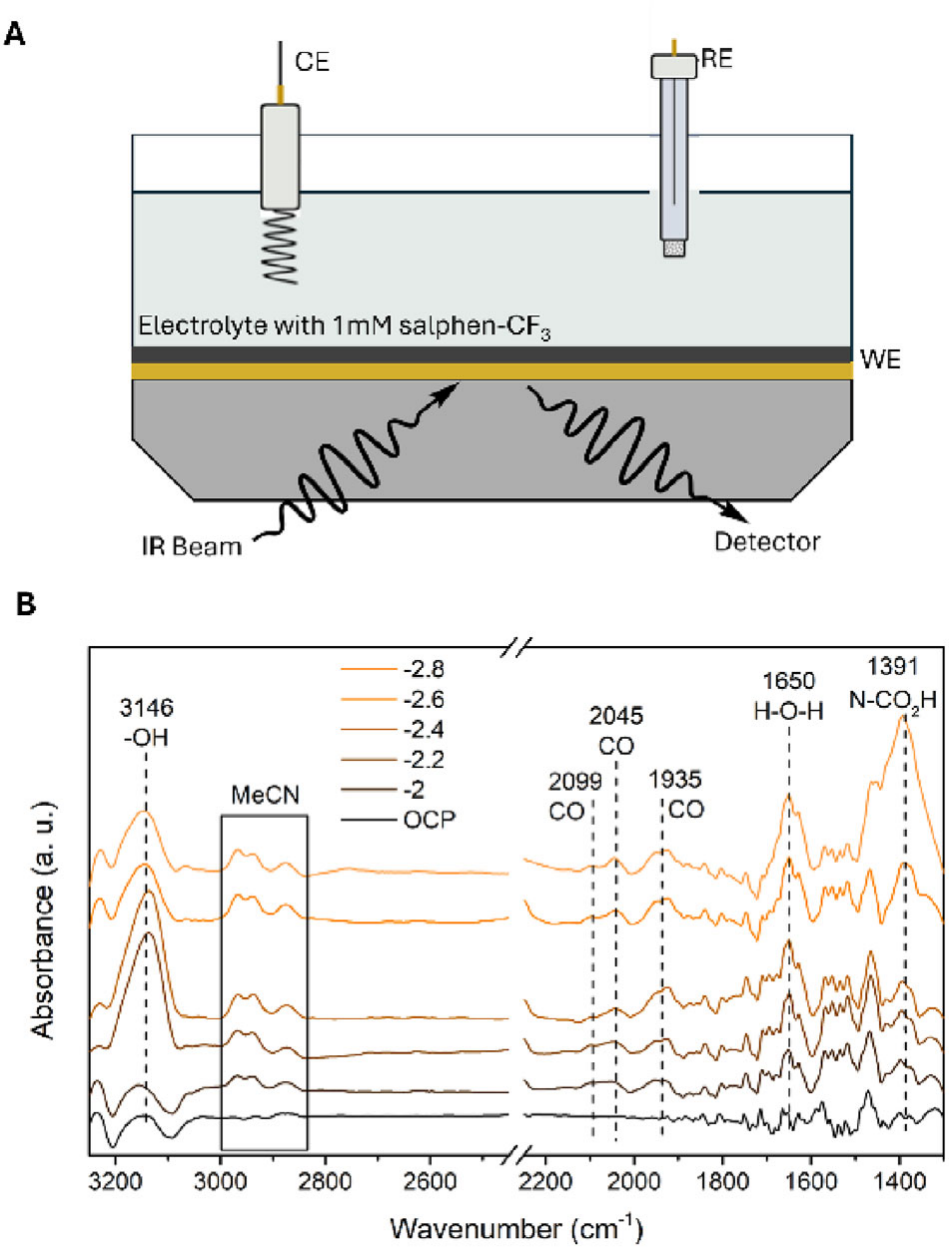
(A) In situ FTIR electrochemical cell. (B) Spectra collected in MeCN containing 0.10 M TBAPF6 and 3% v/v water and continuously purged with CO_2_, using 1.0 mM **salphen‐CF_3_** as catalyst.

Notably, while the CO bands reached a maximum intensity at moderate reductive potentials, the continuous growth of the 1391 cm^−1^ band at a more negative potential points to the accumulation of a reduced CO_2_‐derived intermediate. The confirmed isotopic shift of this feature when using ^13^CO_2_ supports its assignment to an N‐bound carbamate (N–CO_2_^−^) or closely related carboxylate species formed through capture of CO_2_ by the reduced ligand. These spectroscopic signatures provide direct evidence for ligand‐bound CO_2_ intermediates during catalysis. At more negative potentials, the system increasingly populates CO_2_‐bound intermediates rather than enhancing CO turnover, consistent with the trends observed between CO Faradaic efficiency and partial current density.

With these experimental observations in hand, we sought to support the mechanism and the binding mode with theory, by examining first the effect of a reduction event on the system and plausible intermediates arising from the CO_2_ binding. A series of candidate species was considered computationally, including neutral, singly reduced, and doubly reduced species bearing either CO_2_ or CO_2_H adducts (Scheme S6 for more details). Interestingly, the calculated IR spectra of the singly‐reduced **salphen‐CF_3_** species with N‐bound CO_2_H adduct (Figure 6A) exhibit ─NCO_2_H based vibrational modes associated with wagging and bending of the group, approximately at 1390 cm^−1^, showing a good agreement with the experimental observation (Figure 6B).

**FIGURE 6:**
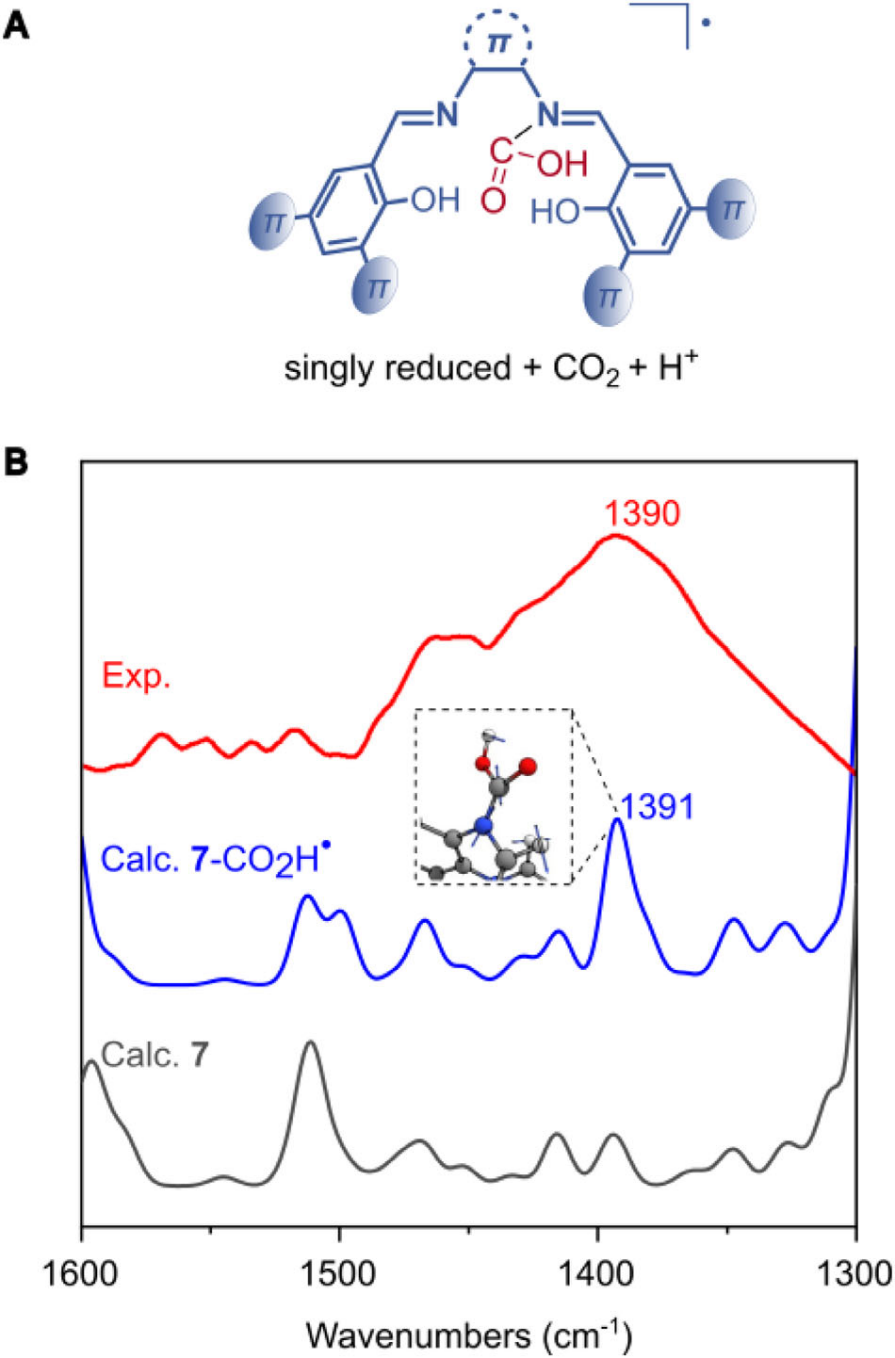
(A) Proposed intermediate after CO_2_ binding. (B) Calculated IR spectra of **salphen‐CF_3_** and singly reduced **salphen‐CF_3_** with a CO_2_H^+^ adduct with experimental data. All spectra were computed at the B97‐3c level with SMD (MeCN) solvation and Boltzmann‐averaged over the optimized conformers.

Although a comparable vibrational feature was also observed for the doubly reduced adduct, we are more prone to suggest the first species as the active one, based on the limited reversibility of the systems, at increasingly negative potentials, provided by the experimental evidence of CV.

Collectively, these computational results corroborate the experimental observations and support a mechanism in which single‐electron reduction activates the ligand framework, enabling CO_2_ binding at the imine nitrogen to form an N‐bound CO_2_H intermediate as the most plausible reactive species under the applied conditions.

As π‐extension was crucial to transform the archetypal salphen framework into an efficient electrocatalyst, the resulting electronic advantages may extend to the modulation of optical properties. Leveraging this common electronic origin, we next explored how the structural rigidity afforded by boron coordination could further amplify these photophysical features for applications in singlet oxygen generation [42, 43, 44].

Although boron‐salen complexes remain relatively rare [45, 46, 47, 48], some examples are reported to form polymers for potential fluorescent materials [49], and, more generally, organic light‐emitting diodes (OLEDs) [50, 51, 52]. These compounds show interesting features, such as a large Stokes‐shift; however, their main absorbance lies often in the UV region, limiting their applications. Thus, extension of the π‐system could be the key to access the visible region.

We found a convenient and efficient protocol using BF_3_·Et_2_O and *N,N*‐diisopropylethylamine (DIPEA) to afford boron difluoride complexes **9–12** in moderate to good yields (Scheme 2)

**SCHEME 2:**
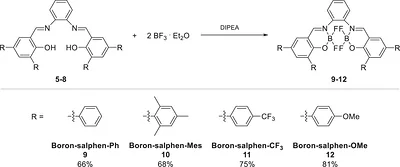
Synthesis of π‐conjugated boron‐salphen complexes.

For complex **9**, its solid‐state structure shows a C_1_‐symmetry, with more pronounced twisting of the phenolate substituents (79.60°, see Supporting Information for further info). The six‐membered ring containing the boron is slightly distorted from planarity, exhibiting a dihedral angle between the O─B─N plane and the C─C─C plane of 31.13°, more than the distortion shown in the boron complex with a simple salen ligand reported in literature [48].

To get a better understanding of the spectroscopic properties, we measured the absorbance and emission spectra and supported them with computational studies (see Figures S30‐33 and Figures S46‐49). Comparing ligands **5–8** with the respective complexes, we noted that both show a strong absorption in the UV region (250–300nm) and a characteristic band in the region of 400 nm (see Supporting Information for more details). Upon complexation with boron, we noted a slight bathochromic shift of the second band, as well as a boost in overall emission intensity (Table 1). Interestingly, complex **10** features the highest fluorescence emission with a relative quantum yield of *Φ*_rel_ = 4.9%, which likely stems from the increased rigidity through the mesityl‐substituents. Furthermore, it is worth pointing out that, besides **10**, the complexes feature Stokes shifts well‐above 7000 cm^−1^, showcasing improvements for this class of compounds as compared to literature (see Table S3) [50].

**TABLE 1:** Photophysical parameters of boron complexes.

Complex	*λ*_abs_ (nm)	*λ*_em_ (nm)^a^	*ε* (M–^1^cm–^1^)^b^	*Φ*_rel_ (%)^c^
9	252; 397	571	8710	1.0
10	274; 380	514	10389	4.9
11	258; 385	555	9550	1.6
12	260; 414	595	7226	0.5

^a^ Details on excitation wavelength are specified in the Supporting Information.

^b^ Reported for second abs. band.

^c^ Relative to Coumarin 153.

With the information obtained from the photophysical properties and based on the literature [53], we set out to test the applicability of boron complexes as photosensitizers for singlet oxygen. We used adamantylideneadamantane **13** as a convenient model substrate to evaluate photooxygenation using **9–12** by ^1^H NMR spectroscopy [54]. The expected product was the 1,2‐dioxetane **14**, but we observed formation of the corresponding ketone **15** as the main product under our working conditions due to photo‐degradation of the dioxetane under high energy irradiation (Scheme 3) [55].

**SCHEME 3:**
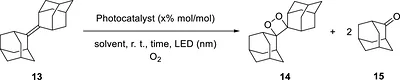
Benchmark oxidation of adamantylideneadamantane *via* singlet oxygen generation.

We started the optimization by evaluating different wavelengths of the light source (Figure 7A), with the best result obtained at *λ* = 390 nm. Then we tested the different boron complexes (Figure 7B), with **12** showing the best activity, probably due to the higher polarizability caused by the ‐OMe substituent. Finally, different solvents (see Supporting Information) and catalyst loading (Figure 7C) were evaluated, obtaining, in CH_2_Cl_2_ and with 500 ppm of boron complex, almost full conversion to ketone after 14 h.

**FIGURE 7:**
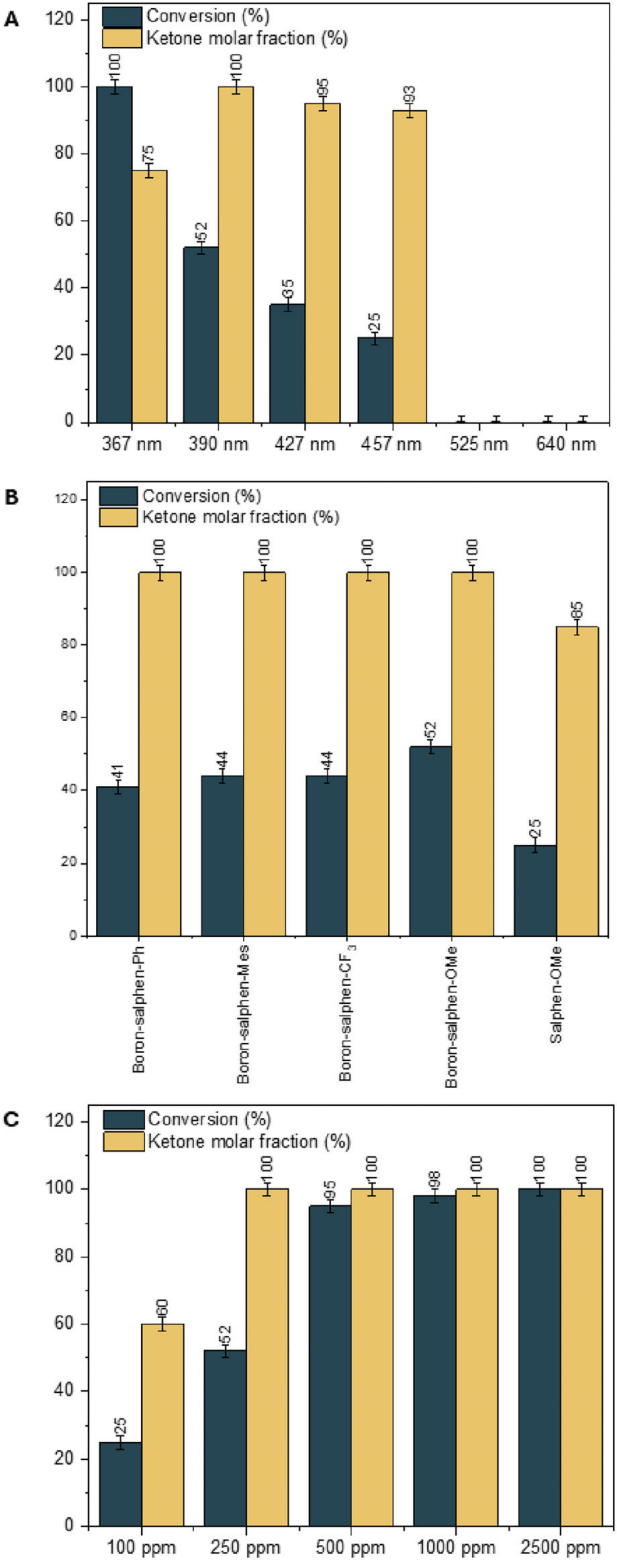
Evaluation of reaction conditions: (A) light wavelength, (B) photocatalyst, (C) catalyst loading (see Supporting Information for details).

To exclude potential background reactions, we carried out control reactions without the catalyst, with boron trifluoride diethyl etherate, as well as in the dark (see the Supporting Information for more information). In all cases, no product formation was observed, highlighting the importance of the photosensitizer for this reaction. Additionally, under identical conditions, but in the presence of one equivalent of 1,4‐diazabicyclo[2.2.2]octane (DABCO) as singlet oxygen quencher, no product formation was observed, hinting at the pivotal role of ^1^O_2_ as oxygen species [56]. To the best of our knowledge, these results showed the first example of salen‐type boron complexes applied as photocatalyst for singlet oxygen formation and double bond oxidative cleavage with very competitive conditions [57].

## 3 Experimental Section

General procedure and specific details for the synthesis of substituted salicylaldehydes (**1‐4**) are reported in the Supporting Information file.

### 3.1 General Procedure for the Preparation of Salphen Ligands (**5–8**)

The corresponding salicylaldehyde (2 equiv., 2.00 mmol) was suspended in methanol (0.1 M), and *ortho*‐1,2‐phenylenediamine (1.1 equiv., 0.119 g, 1.10 mmol) was added. The resulting system was refluxed for 15 h. The reaction mixture was cooled to room temperature, and the product was isolated by filtration. Then, the solid was washed with 30 mL of cold methanol twice and dried under vacuum to get the corresponding salphen ligands **5–8**.

### 3.2 General Procedure for the Preparation of Boron Complexes (**9–12**)

The corresponding ligand (1 equiv., 0.100 mmol) was suspended in toluene (0.02 M), in a closed vial with a septum, and purged with argon for few minutes. Then, *N,N*‐diisopropylethylamine (4 equiv., 0.0695 mL, 0.400 mmol) and boron trifluoride diethyl etherate (5 equiv., 0.123 mL, 1.00 mmol) were added via syringe. The system was stirred at 90°C for 4 h, under argon. After that time, the mixture was cooled down, poured into water, and extracted with DCM (4 × 10 mL). The organic phases were collected, washed with sat. aqueous NH_4_Cl (2 × 15 mL), water (3 × 10 mL), and dried over anhydrous Na_2_SO_4_. After the removal of the solvent, the crude product was washed with hexane (2 × 20 mL) and dried under vacuum to get the corresponding dinuclear boron complexes **9–12**.

### 3.3 General Procedure for CO_2_ Electroreduction

Carbon dioxide electroreduction was performed at various potentials using a Palmsens4 potentiostat in a one‐pot cell with glassy carbon (5 mm) as working electrode, Pt wire as counter electrode and Ag/AgNO_3_ as reference electrode. Each measurement was performed in MeCN with 0.10 M TBAPF_6_ as supporting electrolyte containing 1.0 mM of salen or salphen‐based catalysts under constant CO_2_ or Ar purge, in presence of a certain amount of a proton source. In order to quantify the products of the reaction, gas chromatography was performed with N_2_ as carrier gas under constant flow of CO_2_ or Ar. Gaseous products were quantified through the integration of peak areas corresponding to the products (H_2_ and CO), which were measured using a series of calibration curves.

### 3.4 General Procedure for Singlet Oxygen Generation and Oxidative Cleavage of Double Bond

An 8 mL screw‐cap vial equipped with a rubber septum and a stirring bar was charged with starting material **13** (1 equiv., 0.050 mmol, 0.0133 g), an appropriate amount of catalyst (from stock solution), and solvent (0.5 mL). Oxygen (circa 0.2 bar) was put inside (via FPT: the reaction mixture was prepared in a Schlenk tube, frozen to −196°C, degassed one time and backfilled with oxygen; via balloon: a balloon full of oxygen was connected via needle; via bubbling: oxygen was bubbled inside the system for few seconds). The reaction vessel was irradiated with lights at different wavelengths for a certain time at room temperature. After that, the solvent was removed under reduced pressure and a ^1^H NMR of the crude was recorded with 1,3,5‐trimethoxybenzene (0.6 equiv., 0.0300 mmol, 0.0050 g), as internal standard, in CDCl_3_. The NMR conversions and molar fractions have been determined by integration of diagnostic signals (see Supporting Information for more details).

## 4 Conclusion

In summary, we report a new family of π‐extended salphen ligands that leverage enhanced electronic delocalization across the ligand backbone to modulate redox and photophysical properties. Structural, spectroscopic, electrochemical, and computational studies show that incorporation of phenylene bridges reduces the HOMO–LUMO gap and facilitates ligand‐centered reduction relative to conventional salen frameworks. Among the series, the electron‐deficient **salphen‐CF_3_** derivative mediates metal‐free electrochemical CO_2_ reduction to CO with Faradaic efficiencies up to 56%, an extremely good result for non‐metal‐based system. In situ FTIR spectroscopy and DFT calculations support a ligand‐centered activation pathway in which single‐electron reduction enables CO_2_ capture at the imine nitrogen to form an N‐bound CO_2_H intermediate.

In addition, the corresponding boron difluoride complexes display enhanced fluorescence, large Stokes shifts, and act as efficient photosensitizers for singlet oxygen generation, enabling oxidative cleavage of olefins under mild conditions. More broadly, this work demonstrates how π‐extension can transform classical ligand frameworks into multifunctional platforms capable of ligand‐centred small‐molecule activation and photochemical reactivity.

## Conflicts of Interest

The authors declare no conflicts of interest.

## Supporting information




**Supporting File 1**: chem71385‐sup‐0001‐SuppMat.pdf.

**Supporting File 2**: chem71385‐sup‐0002‐DataSet.zip. The authors have cited additional references within the Supporting Information [58, 59, 60, 61, 62, 63, 64, 65, 66, 67, 68, 69, 70, 71, 72, 73, 74, 75, 76, 77, 78, 79, 80].

## Data Availability

The data that supports the findings of this study are available in the supplementary material of this article.
