# Supplementary material for: π‐Extended Salphen Scaffolds Enable CO2 Electroreduction and Singlet Oxygen Generation
Source: Chemistry. 2026 Jul 12;32(39):e71385. doi: 10.1002/chem.71385 (PMC13715447; doi:10.1002/chem.71385)
Supplement: Supplementary file 1 [file CHEM-32-e71385-s001.pdf]

Supporting Information

π-Extended Salphen Scaffolds Enable CO_2 Electroreduction and Singlet Oxygen Generation

Vincenzo Langellotti,[a] André Korzun,[b] Morgan J. McKee,[b] Massimo Melchiorre,[a] Taegeun Jo,[c] J. Felipe Estrada-Ruiz,[b] Gregor Schnakenburg,[b] Stefano Crespi,*[c] Francesco Ruffo,*[a] Nikolay Kornienko,*[b] Alessandro Bismuto*[b]

[a] Dr. V. Langellotti, Dr. M. Melchiorre, Prof. F. Ruffo
Department of Chemical Sciences
University of Naples Federico II
University Campus of Monte S. Angelo, Via Cintia 21, 80126 Naples, Italy
E-mail: francesco.ruffo@unina.it

[b] Dr. A. Korzun, Mr. M. J. McKee, Mr. J. F. Estrada-Ruiz, Dr. G. Schnakenburg, Prof. N. Kornienko, Prof. A. Bismuto
Institute of Inorganic Chemistry
University of Bonn
Gerhard-Domagk-Str. 1, 53121 Bonn, Germany
E-mail: nkornienko@uni-bonn.de, bismuto@uni-bonn.de

[c] Dr. T. Jo, Prof. S. Crespi
Department of Chemistry – Ångström Laboratory
Uppsala University
Regementsvägen 10, 752 37 Uppsala, Sweden
E-mail: stefano.crespi@kemi.uu.se

Table of Contents

1. General information 4

1.1. Instruments 4

1.2. Solvents and reagents 5

1.3. Electrochemical reaction setup 5

1.4. LED reaction setup 6

2. Compound synthesis 8

2.1. General procedure for the preparation of salicylaldehydes (*A*) (1-4) 8

2.2. General procedure for the preparation of salphen (5-8) and salen (7') ligands (*B*) 10

2.3. General procedure for the preparation of boron complexes (*C*) (10-12) 14

3. Electrochemical data 18

4. CO_2 electroreduction application 27

4.1. Mechanistic investigation*: In situ* attenuated total reflection IR spectroscopy (*in situ* ATR-IR) and kinetic isotope effect (KIE) 35

5. Spectroscopic Studies 37

5.1. General considerations 37

5.2. UV/Vis spectra and determination of molar absorption coefficients (*ε*) 38

5.3. Fluorescence spectroscopy 45

5.4. Relative quantum yield determination 50

5.5. Luminescence lifetime measurements 51

6. Photosensitizer application 53

6.1. Light wavelengths 55

6.2. Oxygen systems 56

6.3. Photocatalysts 57

6.4. Solvents 58

6.5. Catalyst loading 59

6.6. Kinetics 59

6.7. Control experiments 61
7. Theoretical analysis 62
7.1. Computational details 62
7.2. HOMO-LUMO analysis 63
7.3. IR analysis 66
7.4. TD-DFT analysis 69
8. NMR spectra 71
9. Single-crystal X-ray diffraction analysis 88
10. References 91

1. General information

Unless otherwise stated, all experiments were carried out under air. When specified, they were run under an argon atmosphere, using standard Schlenk techniques.

1.1. Instruments

^{1}H, $^{19}F\{^{1}H\}$, $^{11}B\{^{1}H\}$, and $^{13}C\{^{1}H\}$ NMR spectra were recorded in CD_2Cl_2 or $CDCl_3$ at 25°C using a Bruker Avance I 300 MHz, Bruker Avance I 400 MHz, and Bruker Avance III HD Ascend 500 MHz and Bruker Avance III HD Ascend 700 MHz spectrometer. ^{1}H NMR was reported as follows: chemical shift, multiplicity (s = singlet, d = doublet, t = triplet, q = quadruplet, dd = doublet of doublet, dt = doublet of triplet, br = broad, m = multiplet), coupling constant (*J* values) in Hz and integration. Chemical shifts (*δ*) were reported with respect to the corresponding solvent residual peak at 5.32 ppm for CD_2Cl_2 and at 7.26 ppm for $CDCl_3$ for ^{1}H NMR. ^{13}C NMR spectra (^{1}H-broadband decoupled) were reported in ppm using the central peak of CD_2Cl_2 (53.84 ppm) and of $CDCl_3$ (77.16 ppm). If not stated, the NMR signals were singlets. UV/Vis and fluorescence emission spectra were recorded with an Edinburgh Instrument Spectrofluorometer FS5 and JASCO V-750 UV-Visible spectrophotometer using a 10×10 mm quartz sample cell. The temperature of the sample holder was controlled through an external cryostat. Luminescence lifetime was measured with an EPL-365 and EPL-450 pulsed laser diode. Cyclic voltammetry (CV) was performed using a Biologic or Palmsens4 potentiostat. The C, H, and N analyses were carried out in triplicate for each sample using an Elementar Vario Micro

elemental analyzer. The individual C, H, and N values did not differ by more than ±0.3%. The mean C, H, and N values are given for each compound.

1.2. Solvents and reagents

Unless otherwise specified, all reagents were used as received from commercial suppliers (ABCR, AcrosOrganics, Alfa Aesar, BLDPharm, Sigma Aldrich, Tokyo Chemical Industry, Thermo Fisher Scientific). Unsubstituted salen[58] and salphen[59] ligands, and adamantilydeneadamantane (**13**)[60] were synthesized according to literature-reported procedures.

1.3. Electrochemical reaction setup

For electrochemical reactions, a Biologic or Palmsens4 potentiostat was used to perform cyclic voltammetry and chronoamperometric measurements. A one-pot cell was used containing 1.0 mM **salphen-CF$_3$** in MeCN with 0.10 M TBAPF$_6$. Glassy carbon was used as working electrode, Pt wire as counter electrode and Ag/AgNO$_3$ as reference electrode. Carbon dioxide was continuously purged into the cell for the whole duration of the reaction (**Figure S1**).

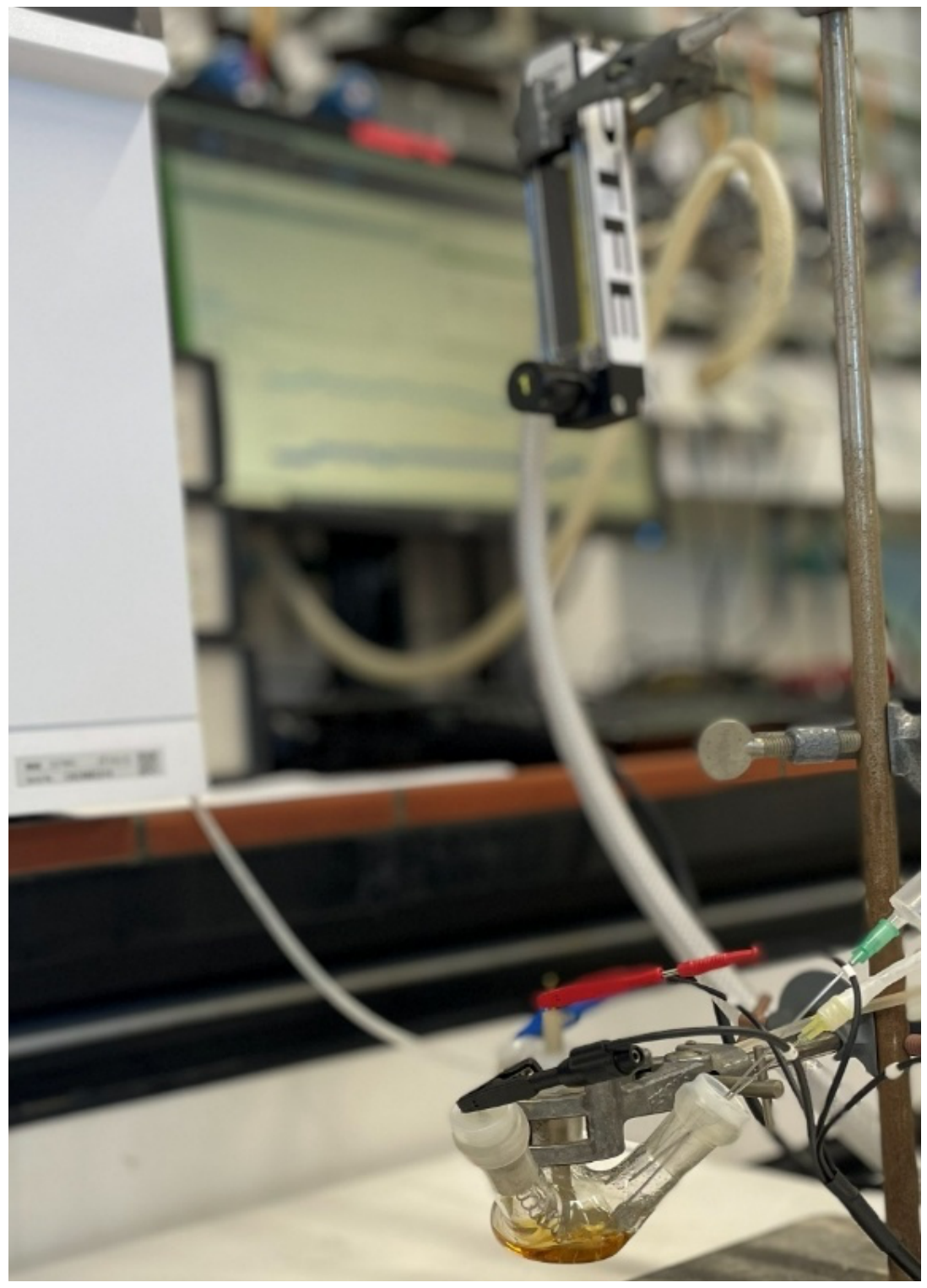

Figure S1. Setup for electrochemical reactions.

1.4. LED reaction setup

LEDs used in this manuscript were purchased from Kessil®: PR160L; 370 nm GEN 2 (max 44W), 390 nm (max 52W), 427 nm (max 45W), 457 nm (max 50W), 525 nm (max 44W), 640 nm (max 44W); 300 mW/cm^2; 11.4 cm x 6.3 cm, beam angle of 56°. Reactions were run in an 8 mL screw-cap vial equipped with a rubber septum, irradiated from 3 cm while stirring and blocked in a vial holder placed on a stirring plate (**Figure S2**).

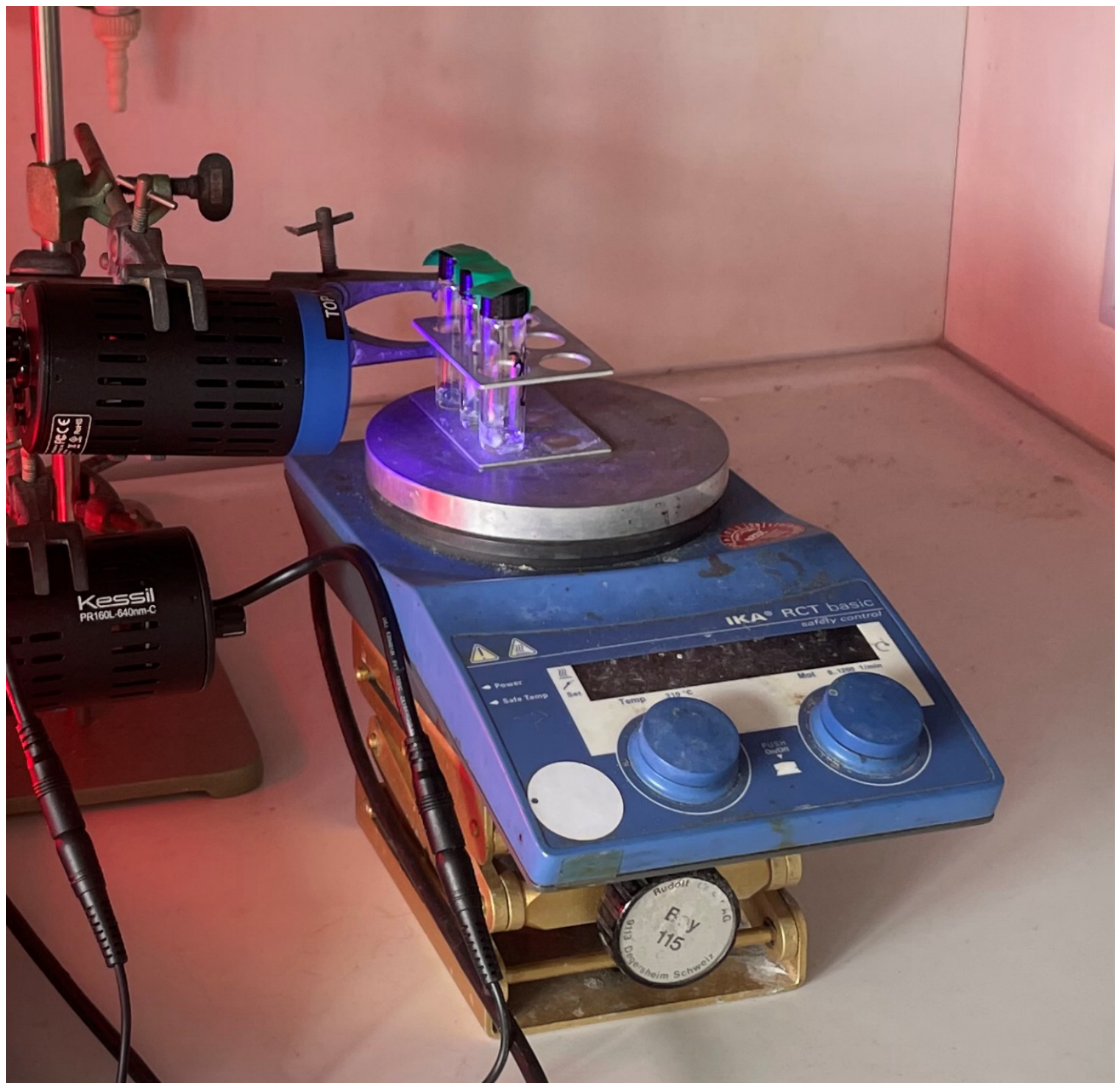

Figure S2. LED setup for photocatalytic applications.

2. Compound synthesis

2.1. General procedure for the preparation of salicylaldehydes (*A*) (1-4)

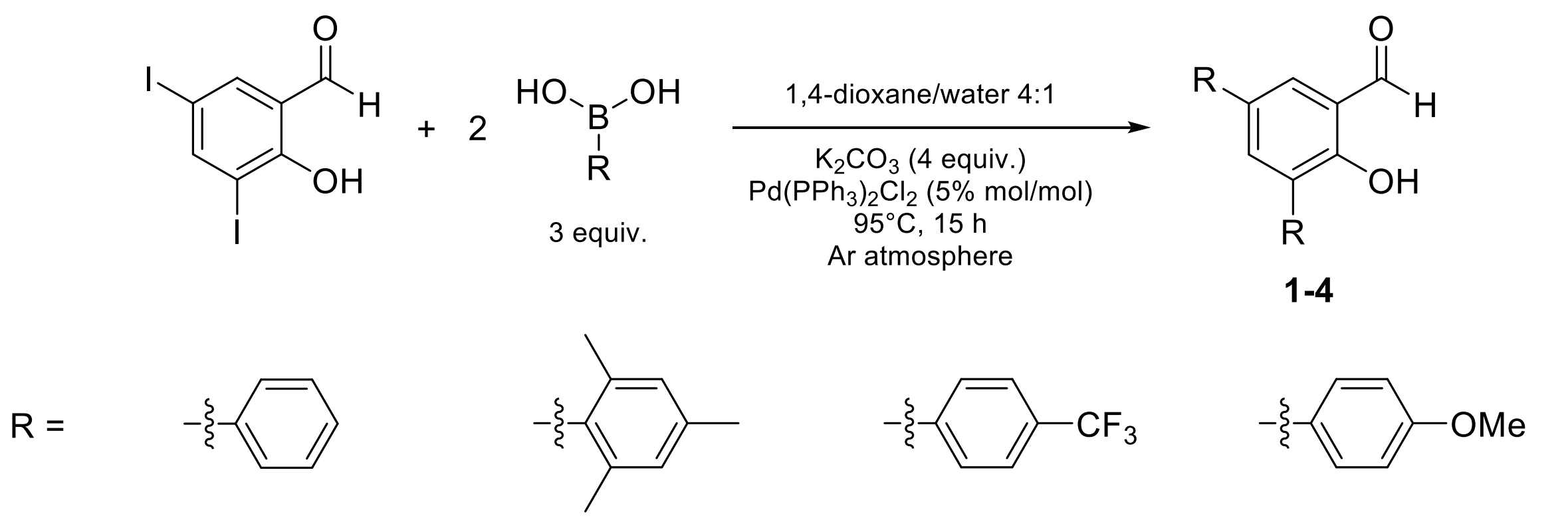

Salicylaldehyde-Ph 1 **Salicylaldehyde-Mes 2** **Salicylaldehyde-CF_3 3** **Salicylaldehyde-OMe 4**

Scheme S1. Synthetic pathway of salicylaldehydes **1-4**.

3,5-disubstituted salicylaldehydes **1-4** were synthesized following modified literature procedures.[30,31] 3,5-diiodosalicylaldehyde (1 equiv., 5.00 g, 10.1 mmol; 0.1 M in 1,4-dioxane/water 4:1), the corresponding boronic acid (3 equiv.), potassium carbonate (4 equiv., 5.58 g, 40.4 mmol) and bis(triphenylphosphine)palladium(II) dichloride (0.05 equiv., 0.354 g, 0.505 mmol) were dried under vacuum in a Schlenk tube under argon atmosphere. 1,4-dioxane/water 4:1 was degassed with argon for 15 minutes, and it was syringed onto the solids. The resulting mixture was stirred for 15 h at 95 °C, under argon. The reaction was then quenched with 2 M HCl solution (same volume of the solvent). After filtration, the crude product was extracted three times with ethyl acetate and the organic layers were washed three times with brine, dried over anhydrous Na_2SO_4, and the solvents were removed in vacuo. The crude product was purified by silica column chromatography to yield salicylaldehydes **1**–**4**. The NMR spectra were in agreement with those reported in the literature.

Salicylaldehyde-Ph (1): The compound was prepared following the general procedure

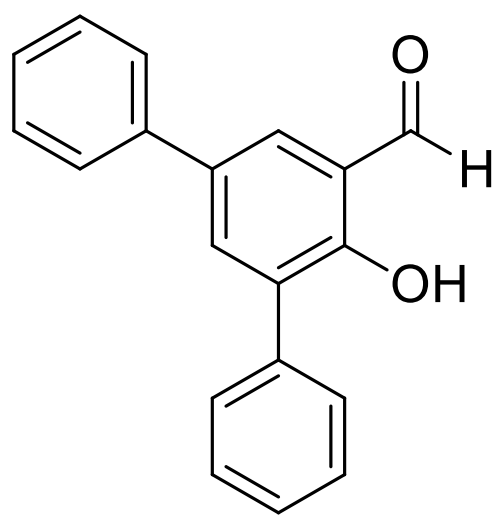

A, using phenylboronic acid (3.69 g, 30.3 mmol). The crude product was purified by column chromatography using as eluent *n*-hexane/ethyl acetate 10:0 to 9:2. The compound was obtained as a pale yellow solid (1.90 g, 52%). **^{1}H NMR** (300 MHz, $CDCl_3$): δ 11.51 (s, 1H), 10.04 (s, 1H), 7.86 (s, 1H), 7.78 (d, *J* = 1.85 Hz, 1H), 7.66-7.59 (m, 4H), 7.50-7.37 (m, 6H).

Salicylaldehyde-Mes (2): The compound was prepared following the general

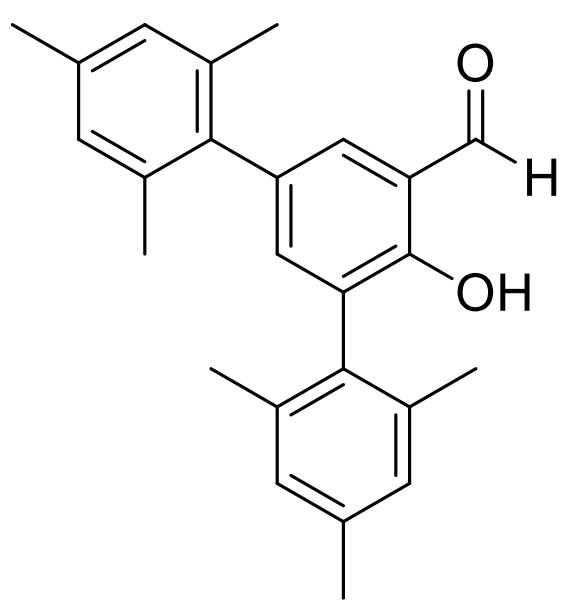

procedure ***A***, using 2,4,6-trimethylphenyl boronic acid (4.97 g, 30.3 mmol). The crude product was purified by column chromatography using as eluent *n*-hexane/ethyl acetate 10:0 to 9:1. The compound was obtained as an off-white solid (5.70 g, 89%). **^{1}H NMR** (300 MHz, $CDCl_3$): δ 11.12 (s, 1H), 9.96 (s, 1H), 7.36 (s, 1H), 7.15 (s, 1H), 6.96 (m, 4H), 2.33 (s, 6H), 2.06 (s, 12H).

Salicylaldehyde-CF_3 (3): The compound was prepared following the general procedure

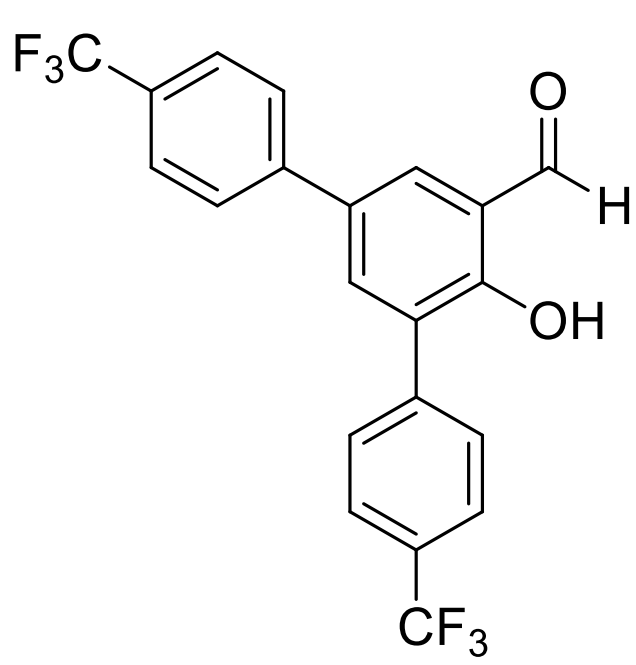

A, using 4-(trifluoromethyl)phenyl boronic acid (5.75 g, 30.3 mmol). The crude product was purified by column chromatography using as eluent *n*-hexane/ethyl acetate 10:0 to 7:3. The compound was obtained as a yellow solid (5.10 g, 92%). **^{1}H NMR** (400 MHz, $CDCl_3$): δ 11.64 (s, 1H), 10.07 (s, 1H), 7.85 (s, 2H), 7.77-7.69 (m, 8H).

Salicylaldehyde-OMe (4): The compound was prepared following the general procedure ***A***, using 4-(methoxy)phenyl boronic acid (4.60 g, 30.3 mmol). The crude product was purified by column chromatography using as eluent *n*-hexane/ethyl acetate 10:0 to 8:2. The compound was obtained as a yellow solid (2.90 g, 64%). **^{1}H NMR** (300 MHz, $CDCl_3$): δ 11.46 (s, 1H), 10.01 (s, 1H), 7.78 (d, *J* = 2.53 Hz, 1H), 7.67 (d, *J* = 2.53 Hz, 1H), 7.58 (d, *J* = 8.63 Hz, 2H), 7.53 (d, *J* = 8.63 Hz, 2H), 7.00 (dd, *J* = 3.51 Hz, *J* = 8.64 Hz, 4H), 3.86 (s, 6H).

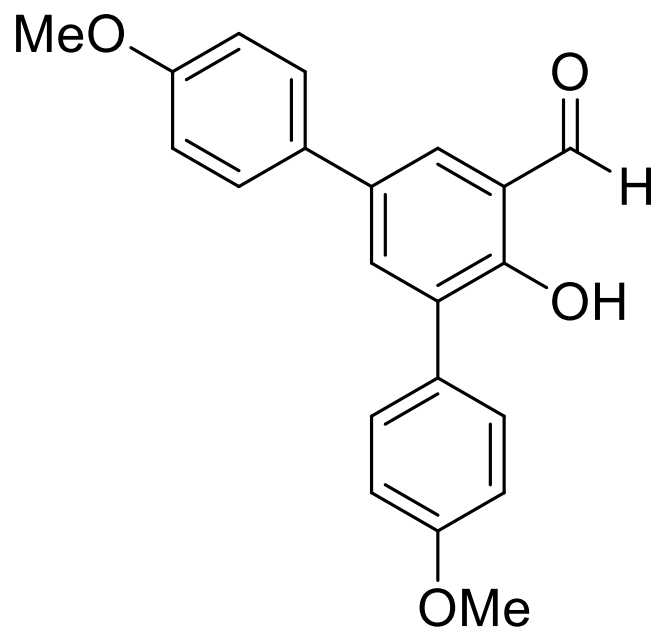

2.2. General procedure for the preparation of salphen (5-8) and salen (7') ligands (*B*)

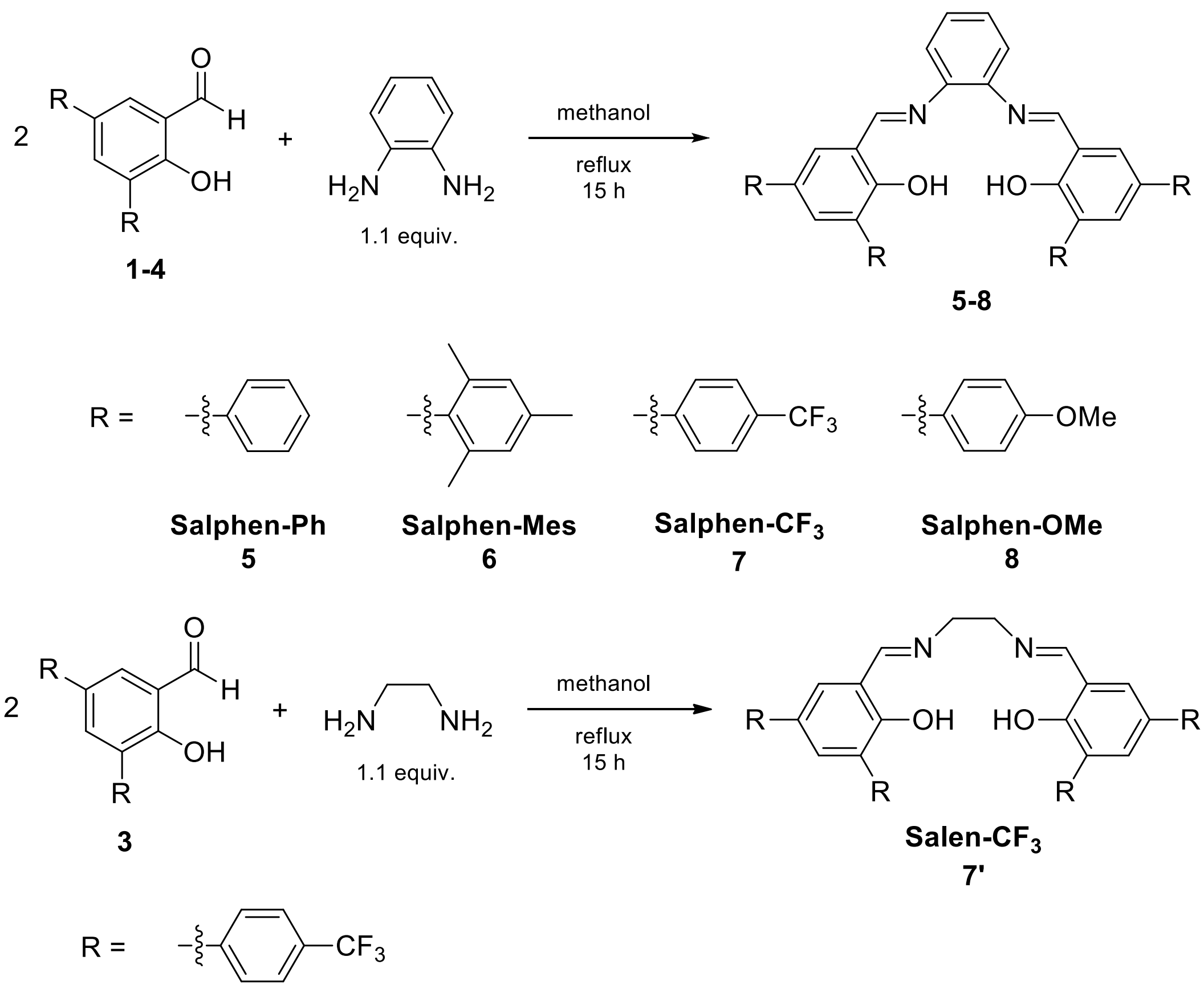

Scheme S2. Synthetic pathway of salphen **5-8** and salen **7'** ligands**.**

Salphen ligands **5-8** were synthesized following a modified literature procedure.[59] The corresponding salicylaldehyde (2 equiv.; 0.1 M in methanol) was suspended in methanol, and *ortho*-1,2-phenylenediamine (1.1 equiv., 0.119 g, 1.10 mmol) was added. The resulting system was refluxed for 15 h. The reaction mixture was cooled to room temperature, and the product was isolated by filtration. Then, the solid was washed with 30 mL of cold methanol twice and dried *in vacuo* to get the corresponding salphen ligand **5-8**.

Salen-CF_3 ligand **7'** was synthesized following the general procedure ***B*** and changing the diamine. The corresponding salicylaldehyde **3** (2 equiv., 0.821 g, 2.00 mmol; 0.1 M in methanol) was suspended in methanol, and 1,2-ethylenediamine (1.1 equiv., 0.0734 mL, 1.10 mmol) was added. The resulting system was refluxed for 15 h. The reaction mixture was cooled to room temperature, and the product was isolated by filtration. Then, the solid was washed with 30 mL of cold methanol twice and dried under vacuum. The product was obtained as yellow solid (0.618 g, 73%).

Salphen-Ph ligand (5): The compound was prepared following the general procedure

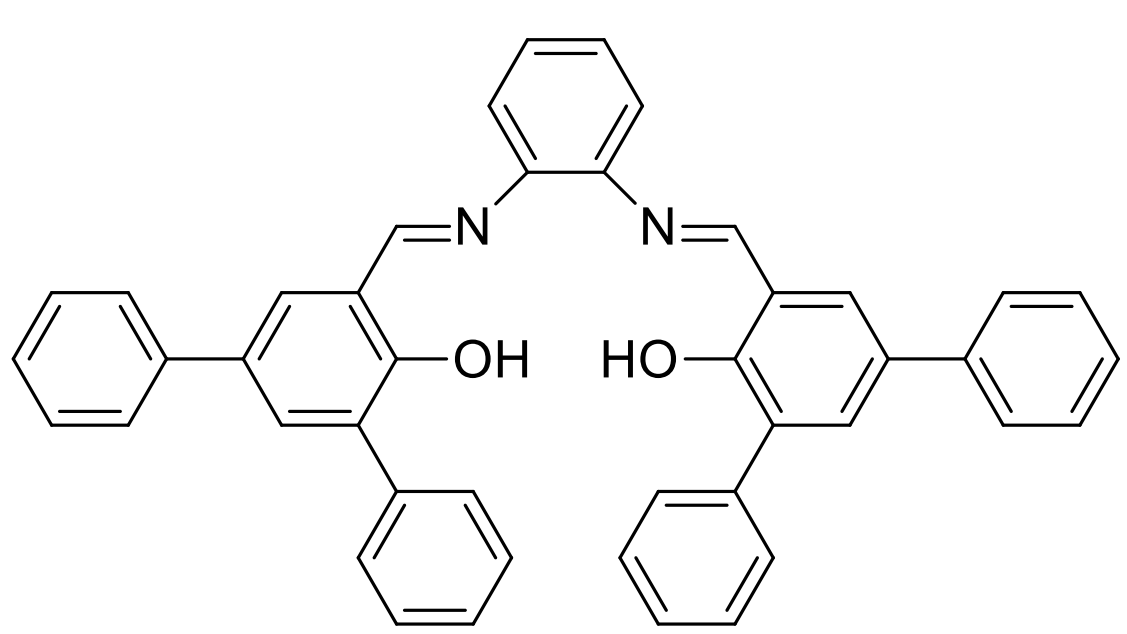

B, starting from salicylaldehyde **1** (0.549 g, 2.00 mmol). The product was obtained as orange solid (0.497 g, 80%). **^{1}H NMR** (400 MHz, $CDCl_3$): δ 13.70 (s, 2H), 8.80 (s, 2H), 7.73 (d, *J* = 2.3 Hz, 2H), 7.69 (d, *J* = 6.9 Hz, 4H), 7.60 (d, *J* = 8.3 Hz, 6H), 7.45 (t, *J* = 7.6 Hz, 4H), 7.40-7.27 (m, 12H). **^{13}C{^{1}H} NMR** (101 MHz, $CDCl_3$) δ 164.2, 158.3, 142.2, 140.2, 137.6, 133.3, 132.3, 130.6, 130.2, 129.5, 129.0, 128.3, 128.0, 127.4, 127.1, 126.7, 120.1, 119.7.

Salphen-Mes ligand (6): The compound was prepared following the general procedure

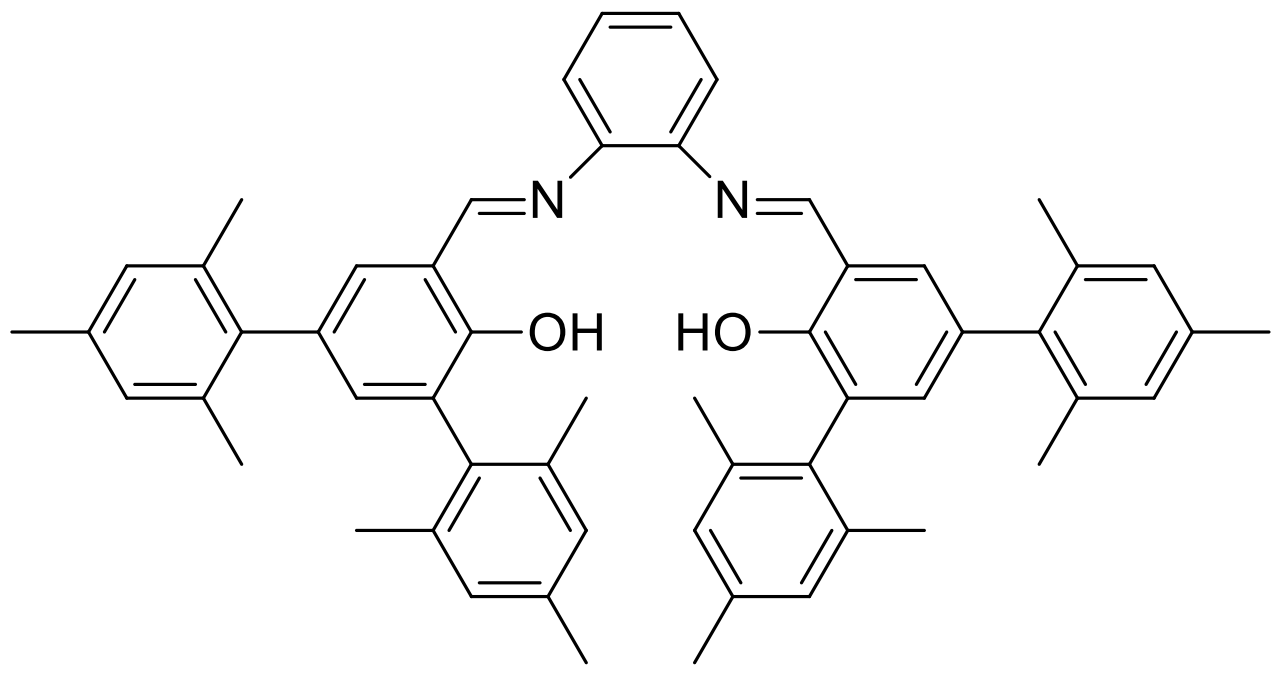

B, starting from salicylaldehyde **2** (0.717 g, 2.00 mmol). The product was obtained as pale yellow solid (0.601 g, 76%). **^{1}H NMR** (500 MHz, $CDCl_3$): δ 13.03 (s, 2H), 8.63 (s, 2H), 7.33 (dd, *J* = 5.9, 3.4 Hz, 2H), 7.19 (dd, *J* = 5.9, 3.4 Hz, 2H), 7.15 (d, *J* = 2.2 Hz, 2H), 6.96 (d, *J* = 2.2 Hz, 2H), 6.95 (s, 8H), 2.34 (s, 6H), 2.33 (s, 6H), 2.08 (s, 12H), 2.03 (s, 12H). **^{13}C{^{1}H} NMR** (126 MHz, $CDCl_3$): δ 164.1, 157.4, 143.1, 138.0, 136.9, 136.8, 136.7, 136.5, 136.2, 134.2, 132.1, 131.7, 129.7, 128.3, 128.2, 127.8, 120.3, 119.5, 21.3, 21.2, 21.1, 20.6.

Salphen-CF_3 ligand (7): The compound was prepared following the general procedure

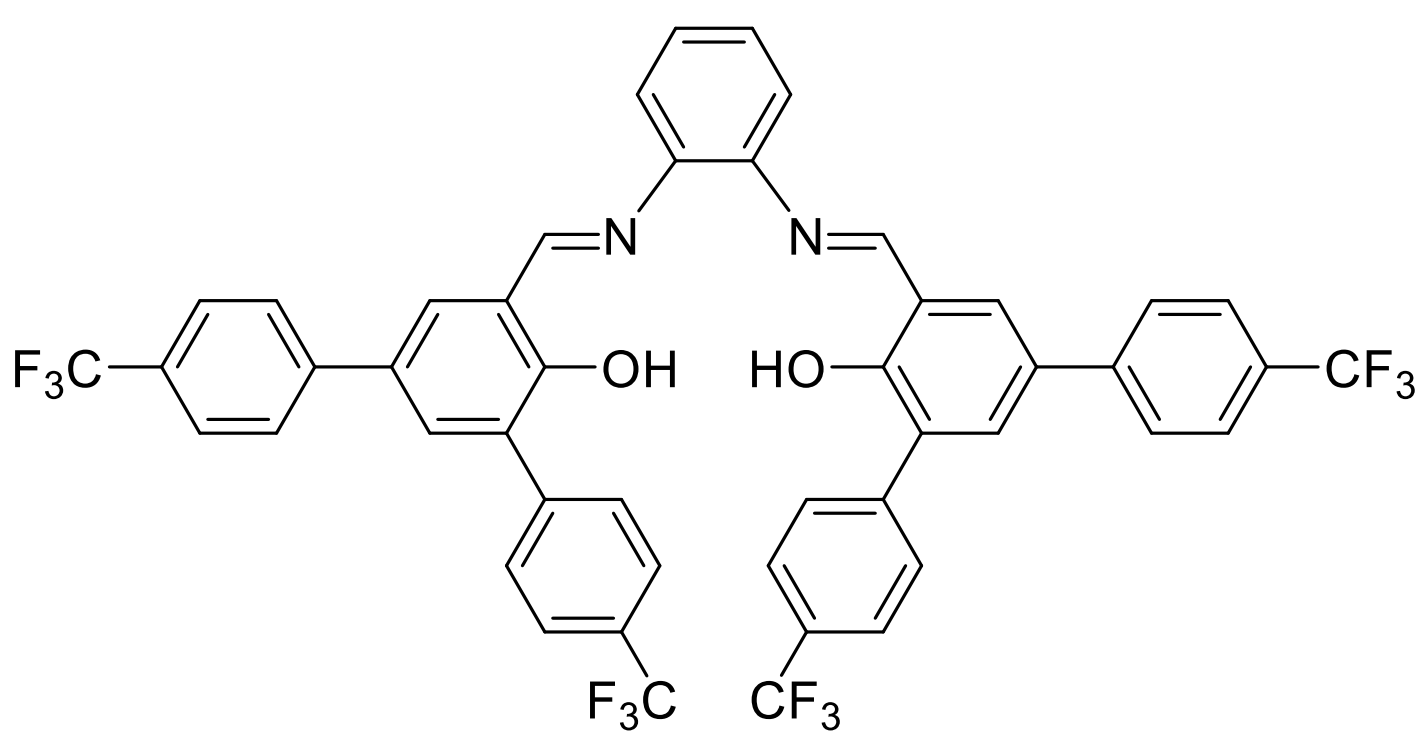

B, starting from salicylaldehyde **3** (0.821 g, 2.00 mmol). The product was obtained as red solid (0.580 g, 65%). **^{1}H NMR** (300 MHz, $CDCl_3$): δ 13.98 (s, 2H), 8.85 (s, 2H), 7.75 (t, *J* = 8.3 Hz, 6H), 7.72-7.67 (m, 10H), 7.57 (d, *J* = 8.3 Hz, 4H), 7.48-7.32 (m, 4H). **^{13}C{^{1}H} NMR** (126 MHz, $CDCl_3$): δ 163.7, 159.1, 143.40 (q, J_{C-F} = 1.2 Hz), 142.1, 140.8, 133.2, 131.1, 131.1, 129.8, 129.7 (q, J_{C-F} = 32.8 Hz), 129.6, 129.5 (q, J_{C-F} = 32.7 Hz), 128.5, 128.5, 126.1 (q, J_{C-F} = 3.7 Hz), 125.39-125.14 (m), 124.4 (q, J_{C-F} = 271.9 Hz), 124.3 (q, J_{C-F} = 272.1 Hz), 120.0, 119.9. **^{19}F{^{1}H} NMR** (470 MHz, $CDCl_3$): δ -64.40, -64.60. Single-crystals suitable for X-ray analysis were grown from a saturated solution of DCM and *n*-hexane at room temperature.

Salphen-OMe ligand (8): The compound was prepared following the general procedure

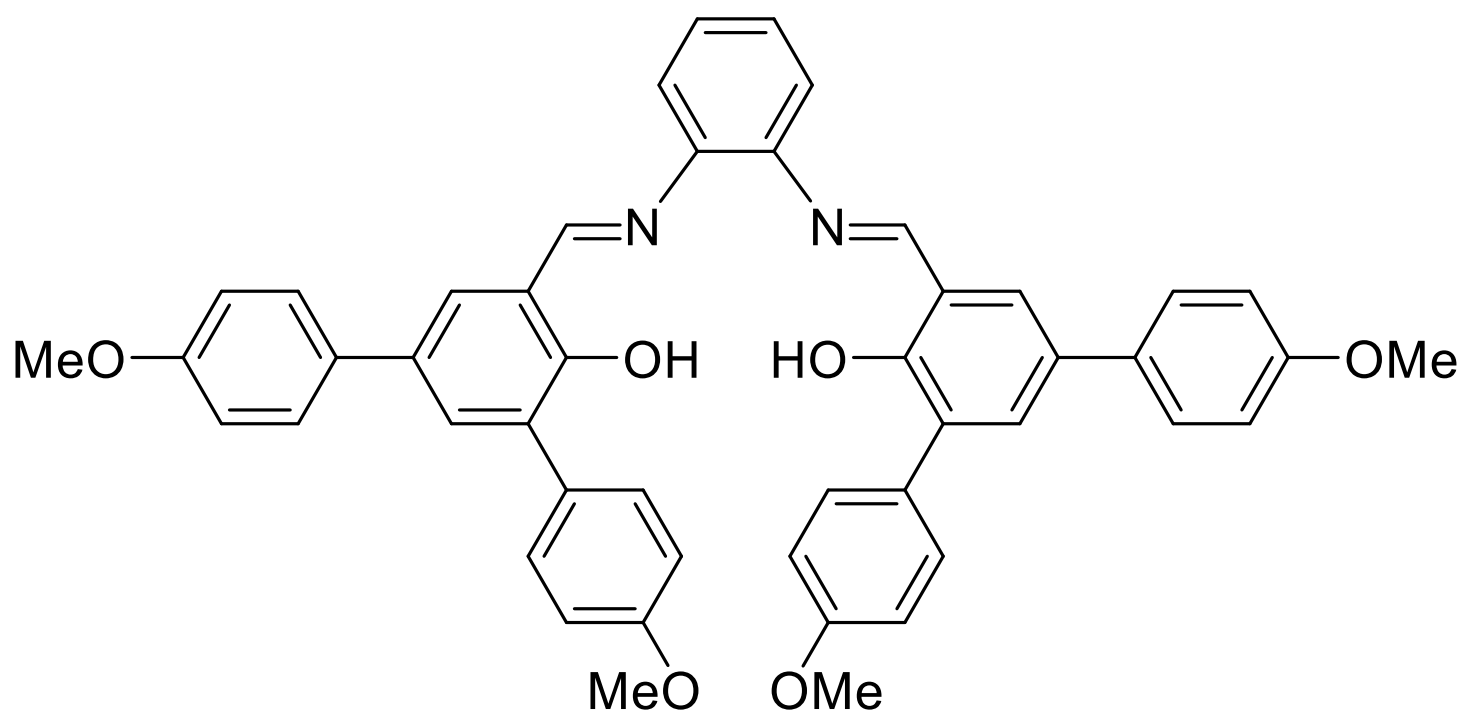

B, starting from salicylaldehyde **4** (0.669 g, 2.00 mmol). The product was obtained as orange solid (0.563 g, 76%). **^{1}H NMR** (400 MHz, $CDCl_3$): δ 13.63 (s, 2H), 8.76 (s, 2H), 7.63 (d, J = 9.6 Hz, 6H), 7.51 (dd, J = 6.0, 2.9 Hz, 6H), 7.34 (m, 2H), 7.27 (m, 2H), 6.97 (d, J = 8.3 Hz, 4H), 6.90 (d, J = 8.3 Hz, 4H), 3.85 (s, 6H), 3.82 (s, 6H). **^{13}C{^{1}H} NMR** (101 MHz, $CDCl_3$): δ 170.2, 159.9, 159.9, 156.3, 139.2, 137.3, 134.3, 132.2, 131.6, 130.9, 130.8, 130.5, 129.4, 128.7, 128.1, 128.0, 114.8, 114.1, 55.7, 55.7.

Salen-CF$_3$ ligand (7'): the compound was prepared following the general procedure ***B***

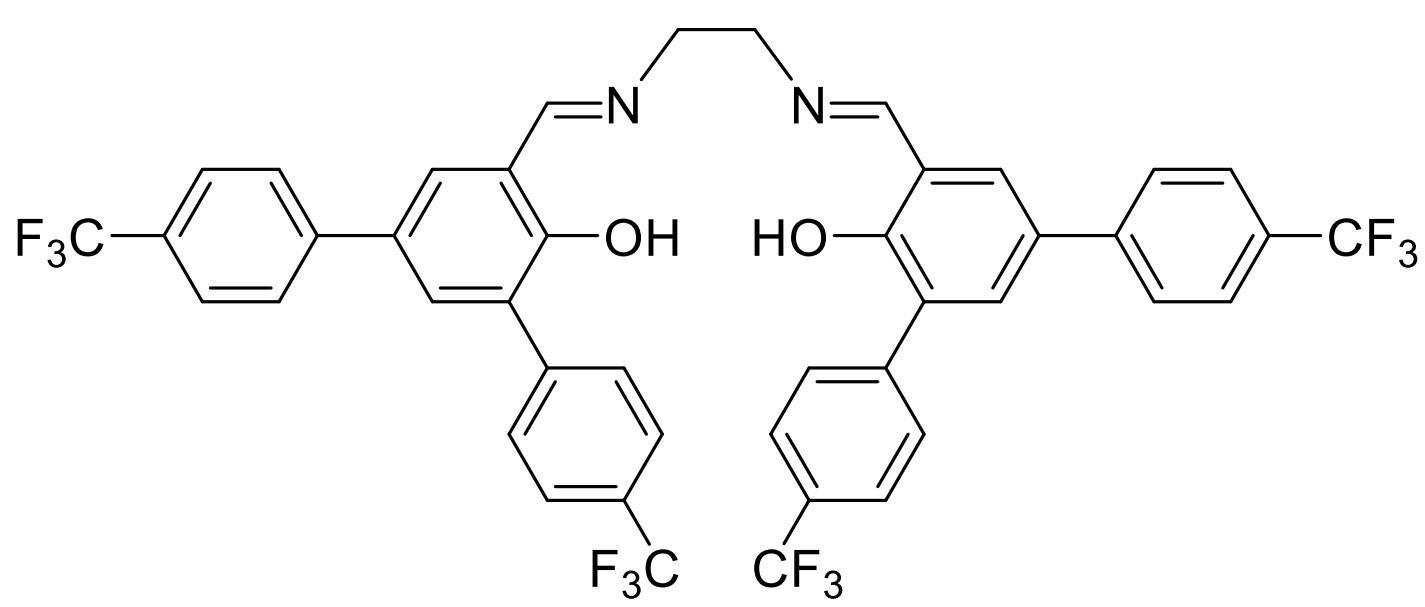

starting from salicylaldehyde **3** as previously described. **^{1}H NMR** (500 MHz, $CDCl_3$): δ 14.09 (s, 2H), 8.53 (s, 2H), 7.75 (d, J = 8.1 Hz, 4H), 7.67 (q, J = 5.0 Hz, 8H), 7.65-7.60 (m, 6H), 7.52 (d, J = 2.4 Hz, 2H), 4.05 (s, 4H). **^{13}C{^{1}H} NMR** (126 MHz, $CDCl_3$): δ 166.8, 158.9, 143.4 (q, $J_{C\text{-}F}$ = 1.3 Hz), 141.0 (q, $J_{C\text{-}F}$ = 1.3 Hz), 132.3, 130.7, 130.3, 129.8, 129.4 (q, $J_{C\text{-}F}$ = 33 Hz, 2C), 129.3, 126.9, 126.1 (q, $J_{C\text{-}F}$ = 3.8 Hz), 125.3 (q, $J_{C\text{-}F}$ = 3.7 Hz), 124.4 (q, $J_{C\text{-}F}$ = 272 Hz, 2C), 119.3, 59.4. **^{19}F{^{1}H} NMR** (470 MHz, $CDCl_3$): δ -62.43, -62.54. Single-crystals suitable for X-ray analysis were grown from a saturated solution of DCM and isopropanol at room temperature.

2.3. General procedure for the preparation of boron complexes (*C*) (10-12)

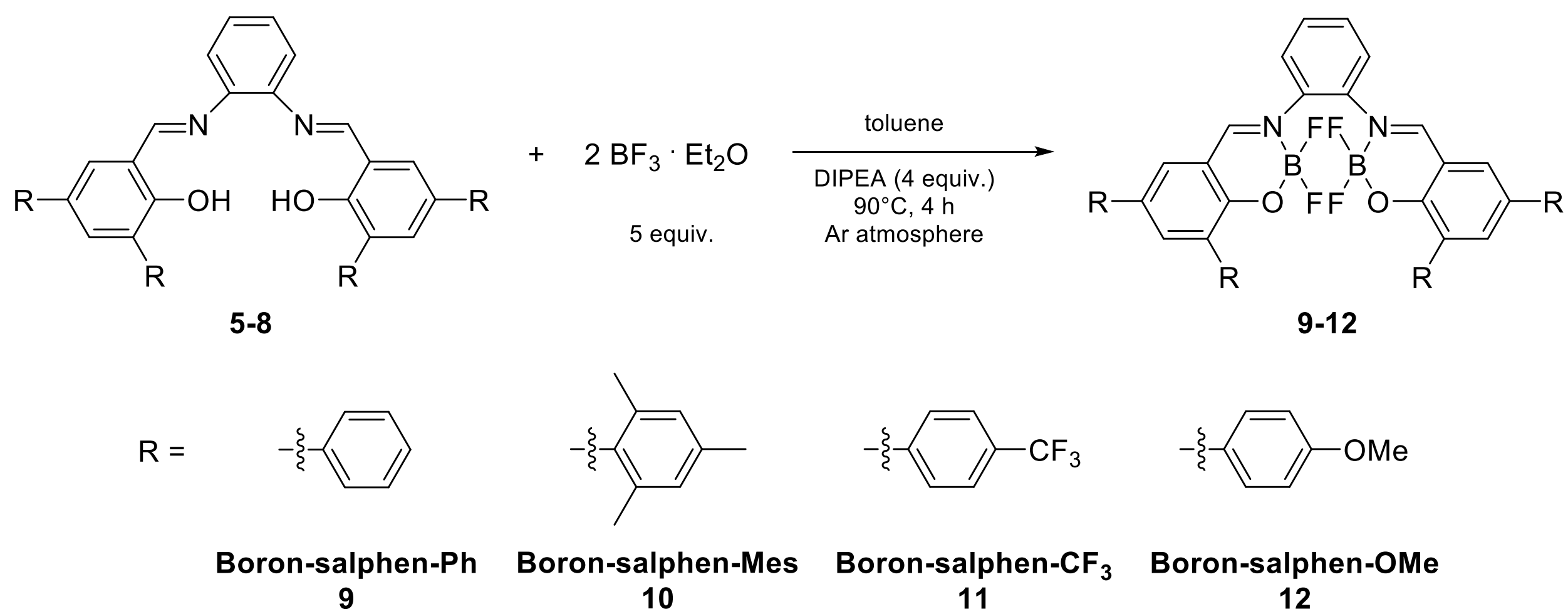

Scheme S3. Synthetic pathway of boron complexes **9-12.**

Boron complexes **9-12** were synthesized following a modified literature procedure.[48] The corresponding ligand (1 equiv.; 0.02 M in toluene) was suspended in toluene, in a closed vial with a septum, and purged with argon for few minutes. Then, *N,N*-diisopropylethylamine (4 equiv., 0.0695 mL, 0.400 mmol) and boron trifluoride diethyl etherate (5 equiv., 0.123 mL, 1.00 mmol) were added via syringe. The system was stirred at 90°C for 4 h, under argon. After that time, the mixture was cooled down, poured into water, and extracted with DCM (4 x 10 mL). The organic phases were collected, washed with sat. aqueous NH_4Cl (2 x 15 mL), water (3 x 10 mL), dried over anhydrous Na_2SO_4. After the removal of the solvent, the crude product was washed with hexane (2 x 20 mL) and dried under vacuum to get the corresponding dinuclear boron complexes **9-12**.

Boron-salphen-Ph (9): The compound was prepared following the general procedure

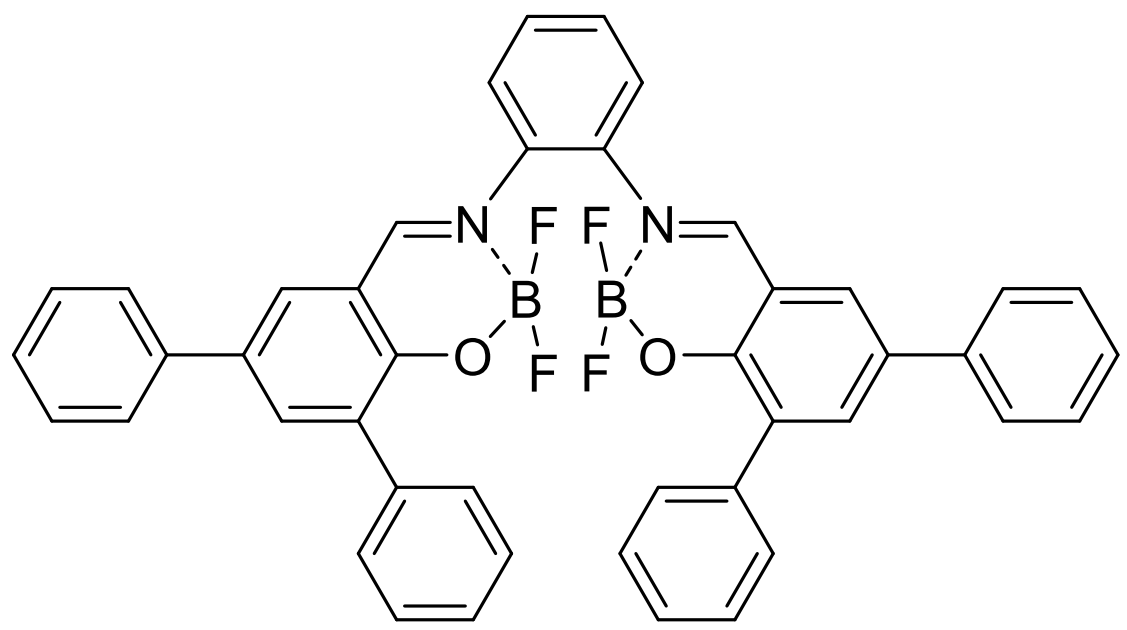

C, starting from salphen ligand **5** (0.0621 g, 0.100 mmol). The product was obtained as dark orange solid (0.0472 g, 66%). **^{1}H NMR** (700 MHz, CD_2Cl_2): δ 8.71 (s, 2H), 7.97 (s, 2H), 7.69 (s, 2H), 7.68 (s, 4H), 7.59 (d, *J* = 8.0 Hz, 4H), 7.55 (d, *J* = 7.6 Hz, 4H), 7.42 (q, *J* = 6.9 Hz, 8H), 7.40-7.33 (m, 4H). **^{13}C{^{1}H} NMR** (176 MHz, CD_2Cl_2): δ 170.2 (t, $J_{C\text{-}F}$ = 5.0 Hz), 156.7, 139.9, 139.0, 137.1, 136.4, 134.5, 132.6, 130.6, 130.6, 129.7, 129.4, 128.7, 128.3, 128.2, 128.1, 127.0, 116.2. **^{11}B{^{1}H} NMR** (160 MHz, CD_2Cl_2): δ 0.65. **^{19}F{^{1}H} NMR** (470 MHz, CD_2Cl_2): δ -134.4. Elemental analysis: Calcd. in % for $C_{44}H_{30}B_2F_4N_2O_2$ (716.24 g/mol): C 73.77, H 4.22, N 3.91; found: C 73.57, H 4.24, N 3.89. Single-crystals suitable for X-ray analysis were grown from a saturated solution of DCM and *n*-dodecane at room temperature.

Boron-salphen-Mes (10): The compound was prepared following the general

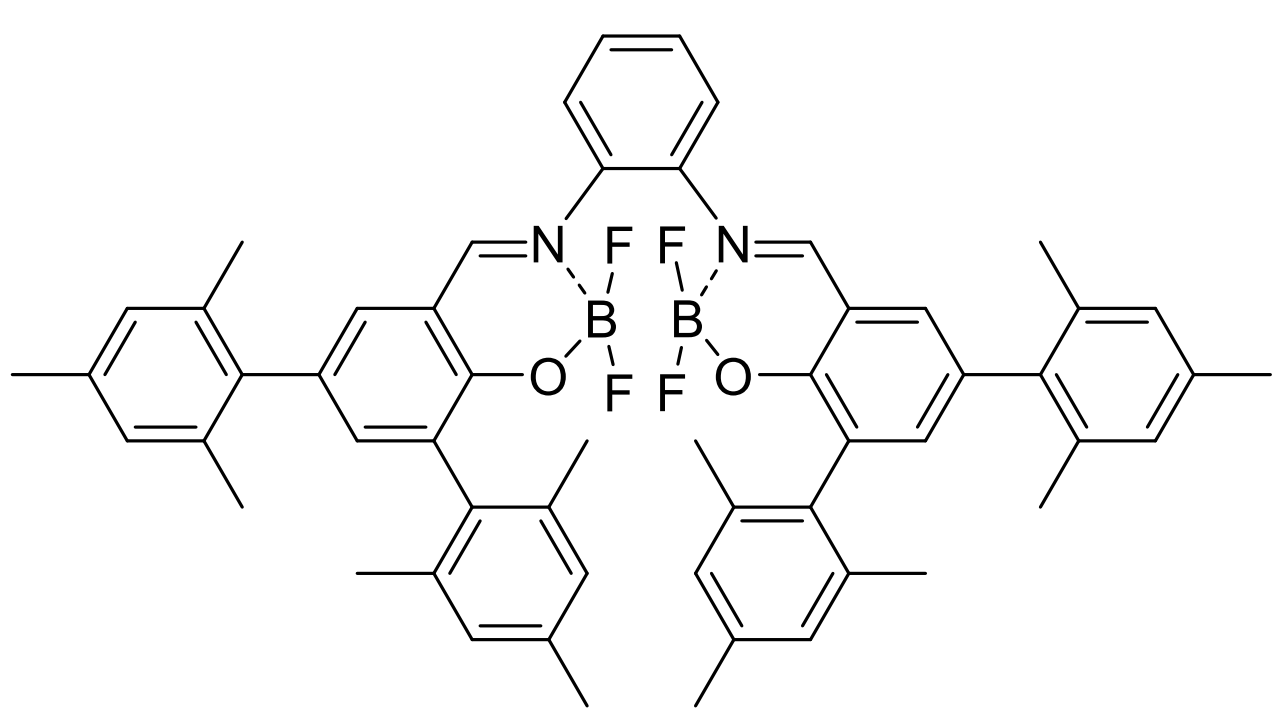

procedure ***C***, starting from salphen ligand **6** (0.0789 g, 0.100 mmol). The product was obtained as dark orange solid (0.0602 g, 68%). **^{1}H NMR** (700 MHz, CD_2Cl_2): δ 8.65 (s, 2H), 7.71-7.58 (m, 4H), 7.25 (d, *J* = 10.4 Hz, 4H), 6.93 (s, 8H), 2.32 (s, 6H), 2.30 (s, 6H), 1.99 (s, 12H), 1.92 (br, 12 H). **^{13}C{^{1}H} NMR** (176 MHz, CD_2Cl_2): δ 170.0 (t, $J_{C\text{-}F}$ = 5.4 Hz), 156.4, 143.5, 137.9, 137.6, 137.1, 136.7, 136.7, 136.2, 134.0, 132.9, 132.8, 131.8, 130.4, 128.6, 128.5, 128.0, 115.7, 21.2, 21.1, 21.0, 20.3. **^{11}B{^{1}H} NMR** (160 MHz, CD_2Cl_2): δ 0.62. **^{19}F{^{1}H} NMR** (470

MHz, CD_2Cl_2): δ -133.9. Elemental analysis: Calcd. in % for $C_{56}H_{54}B_2F_4N_2O_2$ (884.67 g/mol): C 76.03, H 6.15, N 3.17; found: C 75.79, H 6.17, N 3.15.

Boron-salphen-CF_3 (11): The compound was prepared following the general

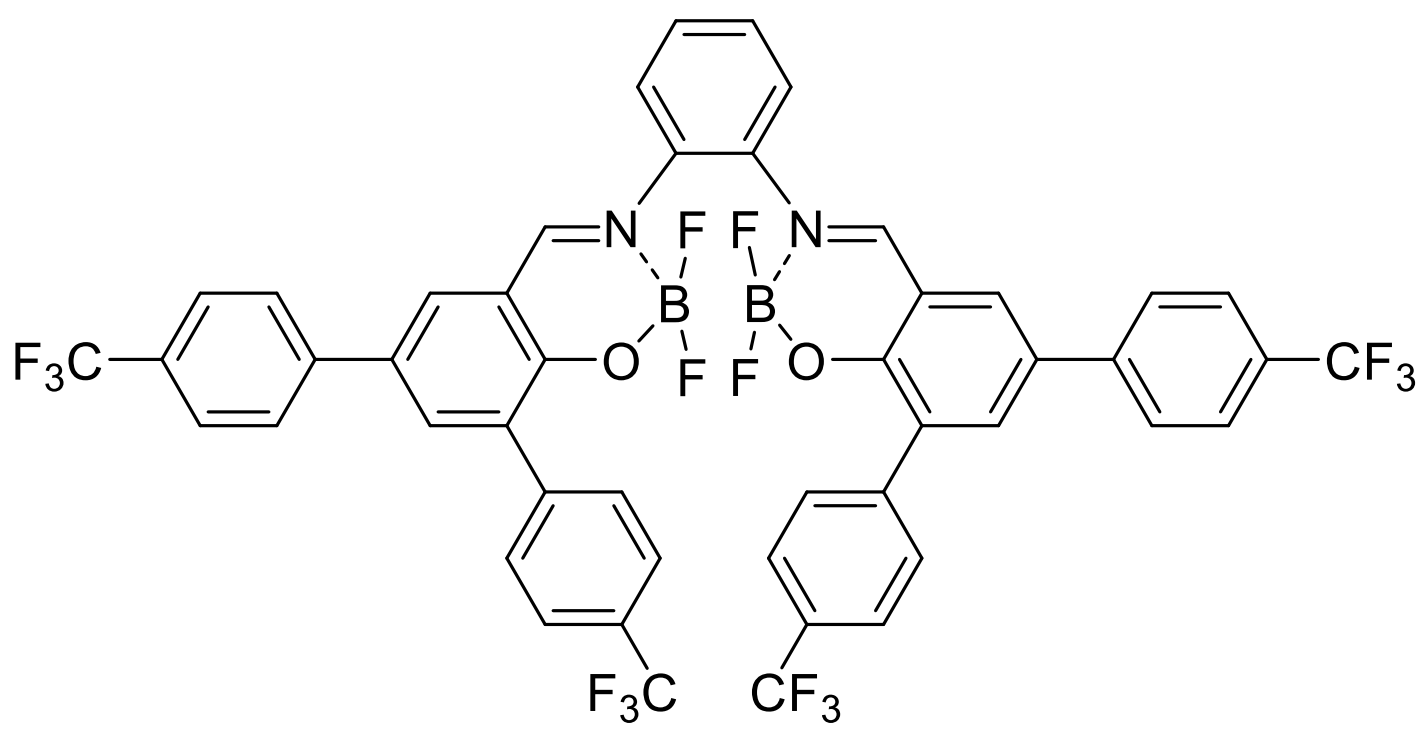

procedure ***C***, starting from salphen ligand **7** (0.0892 g, 0.100 mmol). The product was obtained as red solid (0.0741 g, 75%). **^{1}H NMR** (400 MHz, CD_2Cl_2): δ 8.74 (s, 2H), 7.99 (s, 2H), 7.79 (s, 2H), 7.71 (dt, *J* = 14.9, 8.2 Hz, 20H). **^{13}C{^{1}H} NMR** (101 MHz, CD_2Cl_2): δ 170.3 (t, $J_{C\text{-}F}$ = 4.0 Hz), 157.2, 142.4, 139.8, 139.5, 136.9, 133.2, 131.7, 131.5, 131.0, 130.24 (q, $J_{C\text{-}F}$ = 32.5 Hz), 130.1, 130.10 (q, $J_{C\text{-}F}$ = 32.6 Hz), 128.2, 127.4, 126.4 (q, $J_{C\text{-}F}$ = 3.8 Hz), 125.7 (q, $J_{C\text{-}F}$ = 3.9 Hz), 124.62 (q, $J_{C\text{-}F}$ = 272.1 Hz), 116.5. **^{11}B{^{1}H} NMR** (128 MHz, CD_2Cl_2): δ 0.61. **^{19}F{^{1}H} NMR** (470 MHz, CD_2Cl_2): δ -62.9, -63.0, -134.2, -134.5. Elemental analysis: Calcd. in % for $C_{48}H_{26}B_2F_{16}N_2O_2$ (988.34 g/mol): C 58.33, H 2.65, N 2.83; found: C 58.51, H 2.64, N 2.82.

Boron-salphen-OMe (12): The compound was prepared following the general

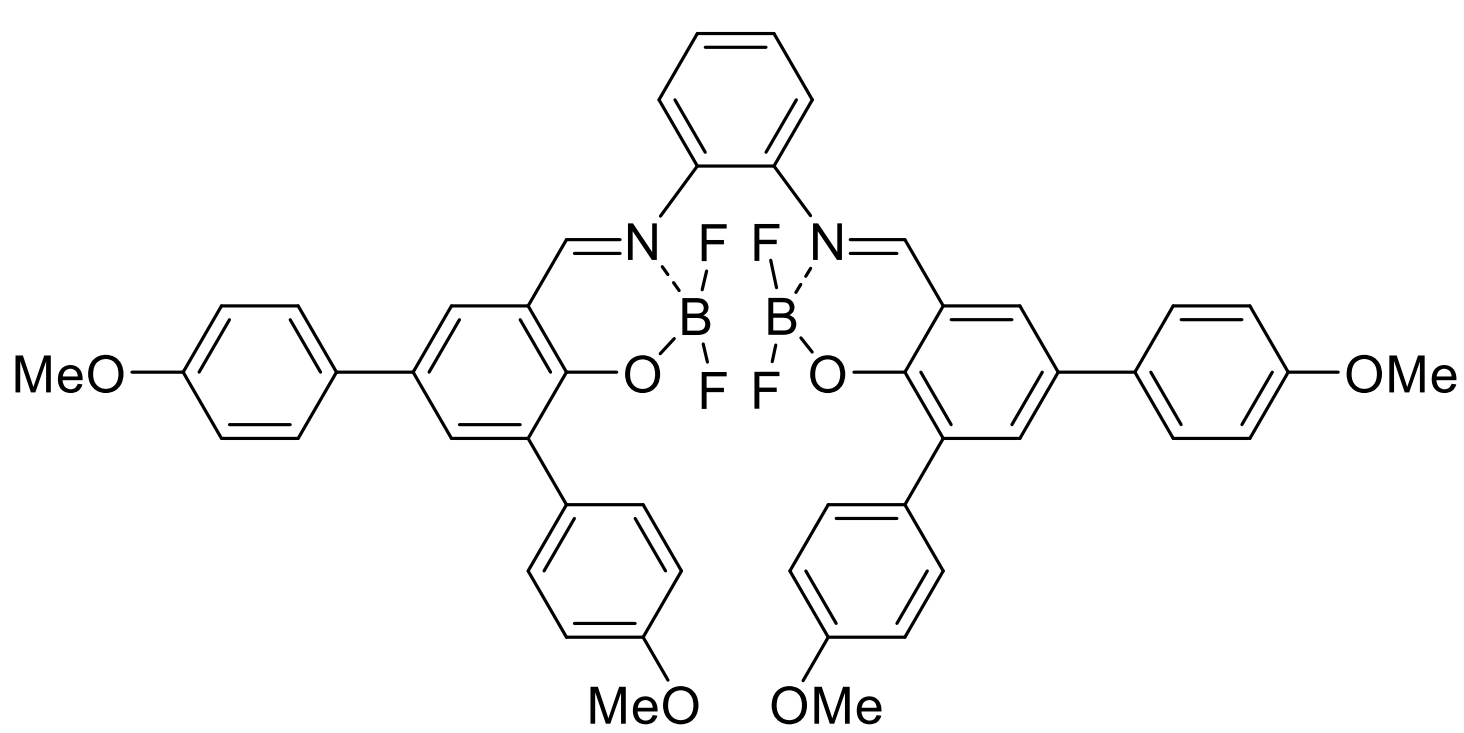

procedure ***C***, starting from salphen ligand **8** (0.0741 g, 0.100 mmol). The product was obtained as red solid (0.0677 g, 81%). **^{1}H NMR** (400 MHz, CD_2Cl_2): δ 8.67 (s, 2H), 7.88 (d, *J* = 2.4 Hz, 2H), 7.67 (s, 4H), 7.57 (d, *J* = 2.6 Hz, 2H), 7.52 (d, *J* = 8.9 Hz, 4H), 7.46 (d, *J* = 8.8 Hz, 4H), 6.97-6.90 (m, 8H), 3.82 (s, 12H). **^{13}C{^{1}H} NMR** (126 MHz,

CD_2Cl_2): δ 170.2 (t, $J_{C\text{-}F}$ = 5.1 Hz), 160.0, 159.9, 156.3, 139.2, 137.3, 134.3, 132.3, 131.6, 130.9, 130.8, 130.5, 129.4, 128.7, 128.1, 128.0, 114.8, 114.1, 55.7, 55.7. **$^{11}B\{^1H\}$ NMR** (128 MHz, CD_2Cl_2): δ 0.78. **$^{19}F\{^1H\}$ NMR** (470 MHz, CD_2Cl_2): δ -136.5. Elemental analysis: Calcd. in % for $C_{48}H_{38}B_2F_4N_2O_6$ (836.45 g/mol): C 68.93, H 4.58, N 3.35; found: C 68.80, H 4.56, N 3.37.

3. Electrochemical data

Cyclic voltammetry (CV) measurements were performed using a glassy carbon as the working electrode, Pt wire as the counter electrode and an Ag/$AgNO_3$ organic reference electrode in a one-compartment cell. These measurements were carried out in acetonitrile with 0.10 M $TBAPF_6$, as supporting electrolyte, which had been dried in an oven to remove any moisture. 1.0 mM of the salphen ligand or boron complex was used, scanning in the range of 1.15 ~ -2.6 V (vs. Ag/$AgNO_3$) at a sweep rate of 100 mV s^{-1}. All CV measurements were conducted using a non-aqueous silver/silver nitrate (Ag/$AgNO_3$) reference electrode (0.01 M $AgNO_3$ in acetonitrile with 0.10 M tetrabutylammonium hexafluorophosphate). To ensure the stability and accuracy of the reference potential, ferrocene (Fc) was used as an internal standard in separate measurements before and after each experimental series to ensure no potential drift. Before the measurement, Ar or CO_2 from cylinders were bubbled into the solution.

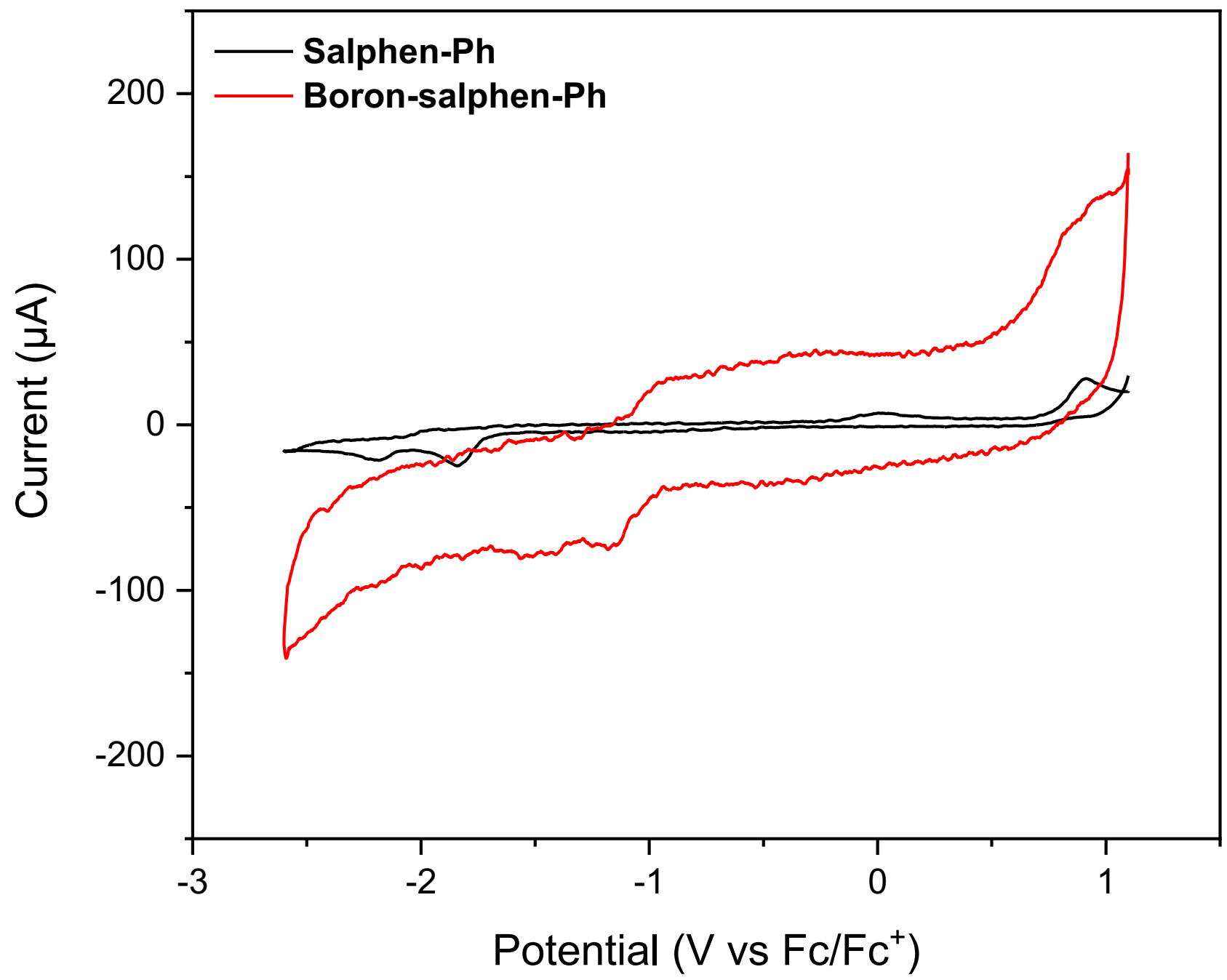

Figure S3. Cyclic voltammogram of 1.0 mM **salphen-Ph** (black line) and **boron-salphen-Ph** (red line) in acetonitrile with 0.10 M $TBAPF_6$ supporting electrolyte at a scan rate of 100 mV/s, with an argon atmosphere.

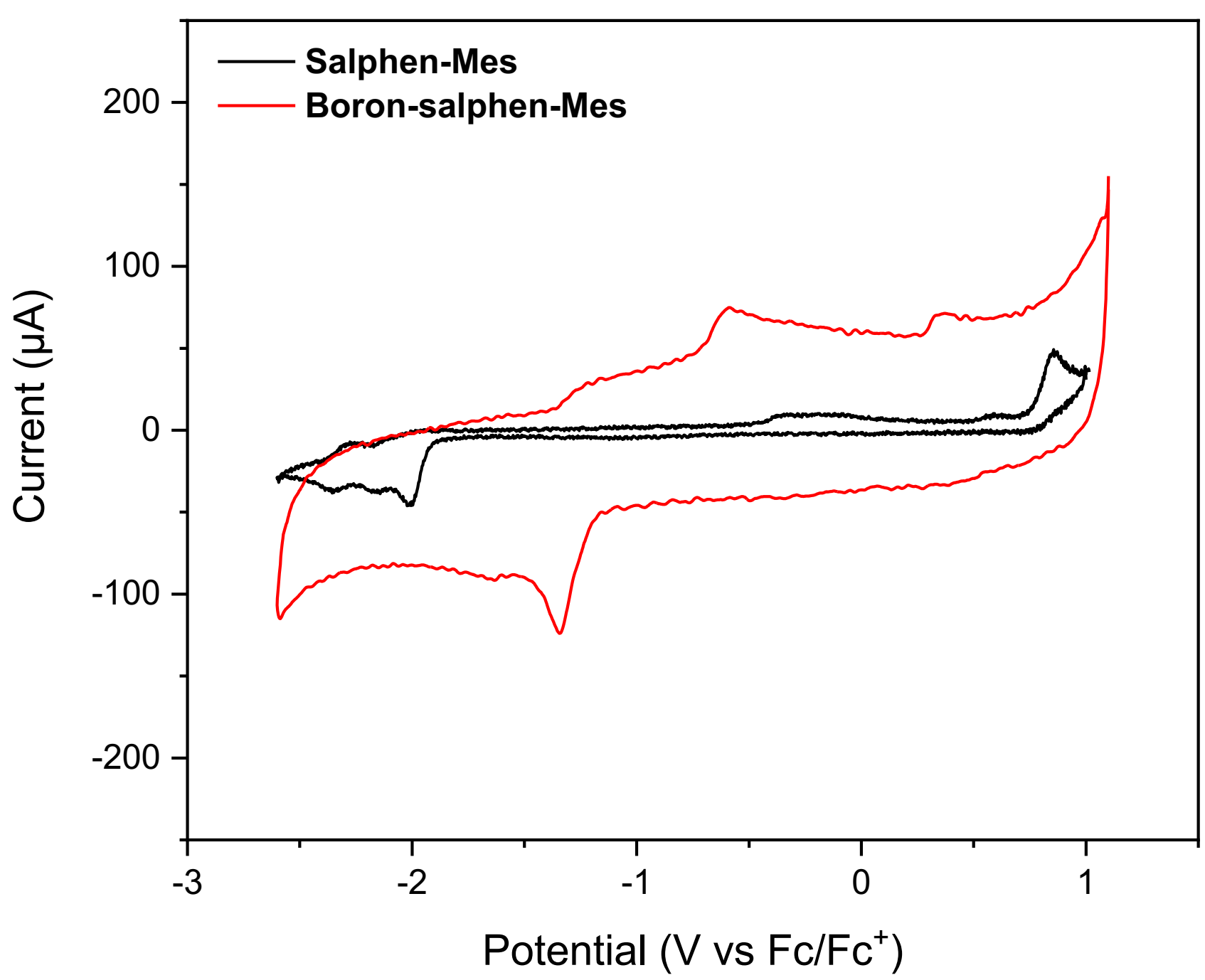

Figure S4. Cyclic voltammogram of 1.0 mM **salphen-Mes** (black line) and **boron-salphen-Mes** (red line) in acetonitrile with 0.10 M $TBAPF_6$ supporting electrolyte at a scan rate of 100 mV/s, with an argon atmosphere.

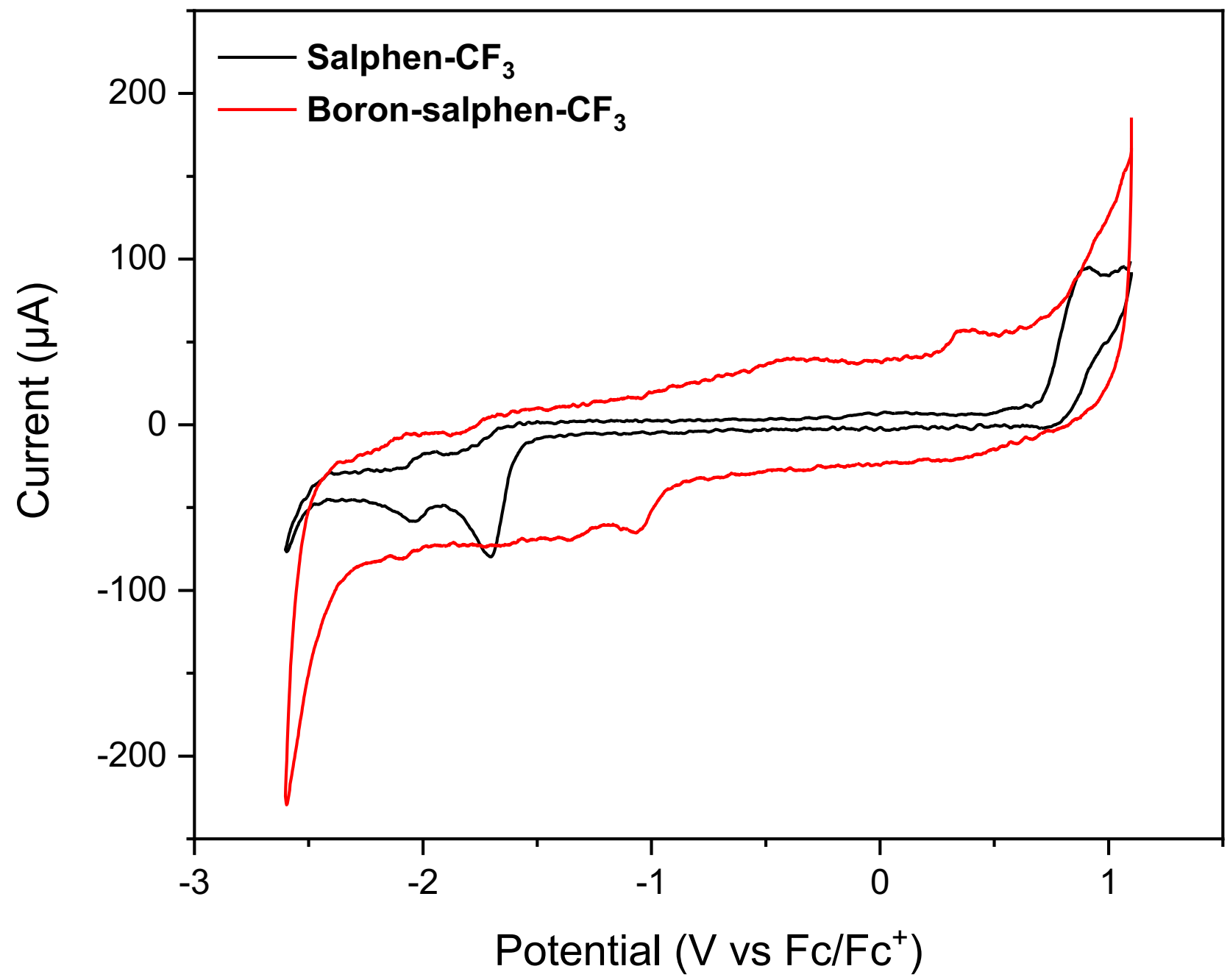

Figure S5. Cyclic voltammogram of 1.0 mM **salphen-CF_3** (black line) and **boron-salphen-CF_3** (red line) in acetonitrile with 0.10 M $TBAPF_6$ supporting electrolyte at a scan rate of 100 mV/s, with an argon atmosphere.

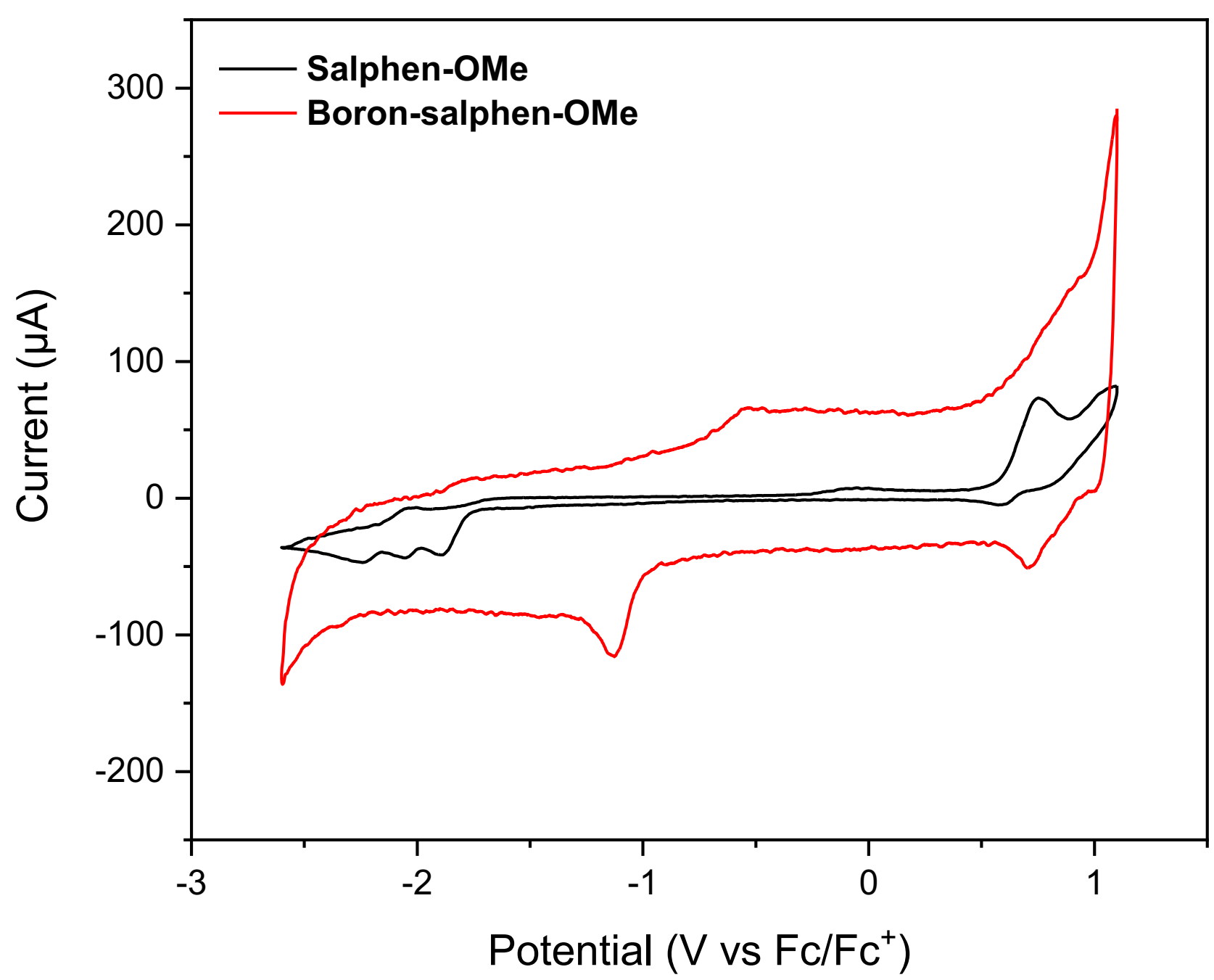

Figure S6. Cyclic voltammogram of 1.0 mM **salphen-OMe** (black line) and **boron-salphen-OMe** (red line) in acetonitrile with 0.10 M $TBAPF_6$ supporting electrolyte at a scan rate of 100 mV/s, with an argon atmosphere.

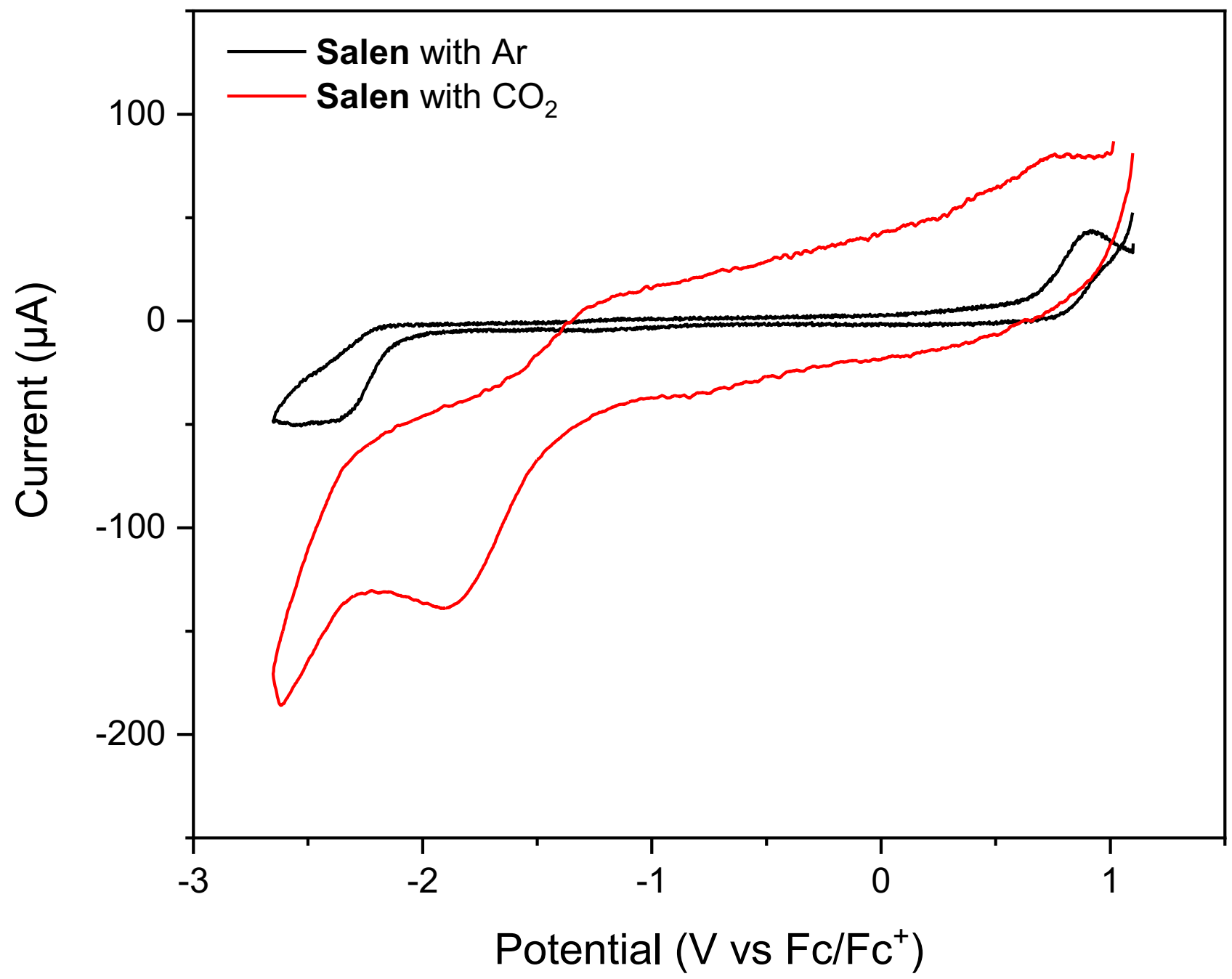

Figure S7. Cyclic voltammogram of 1.0 mM **salen** in acetonitrile with 0.10 M $TBAPF_6$ supporting electrolyte at a scan rate of 100 mV/s, with an argon (black line) or CO_2 (red line) atmosphere.

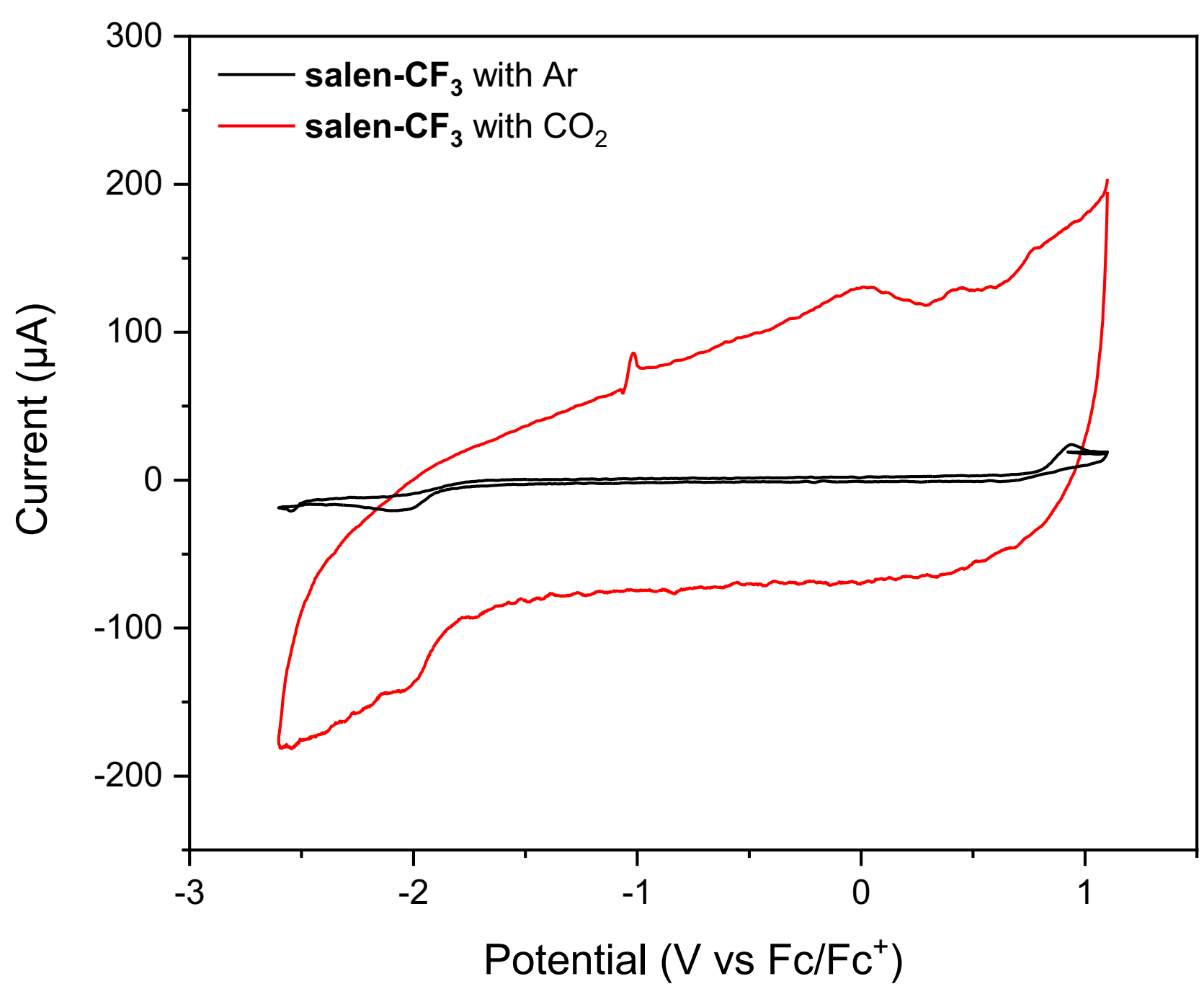

Figure S8. Cyclic voltammogram of 1.0 mM **salen-CF$_3$** in acetonitrile with 0.10 M TBAPF$_6$ supporting electrolyte at a scan rate of 100 mV/s, with an argon (black line) or CO_2 (red line) atmosphere.

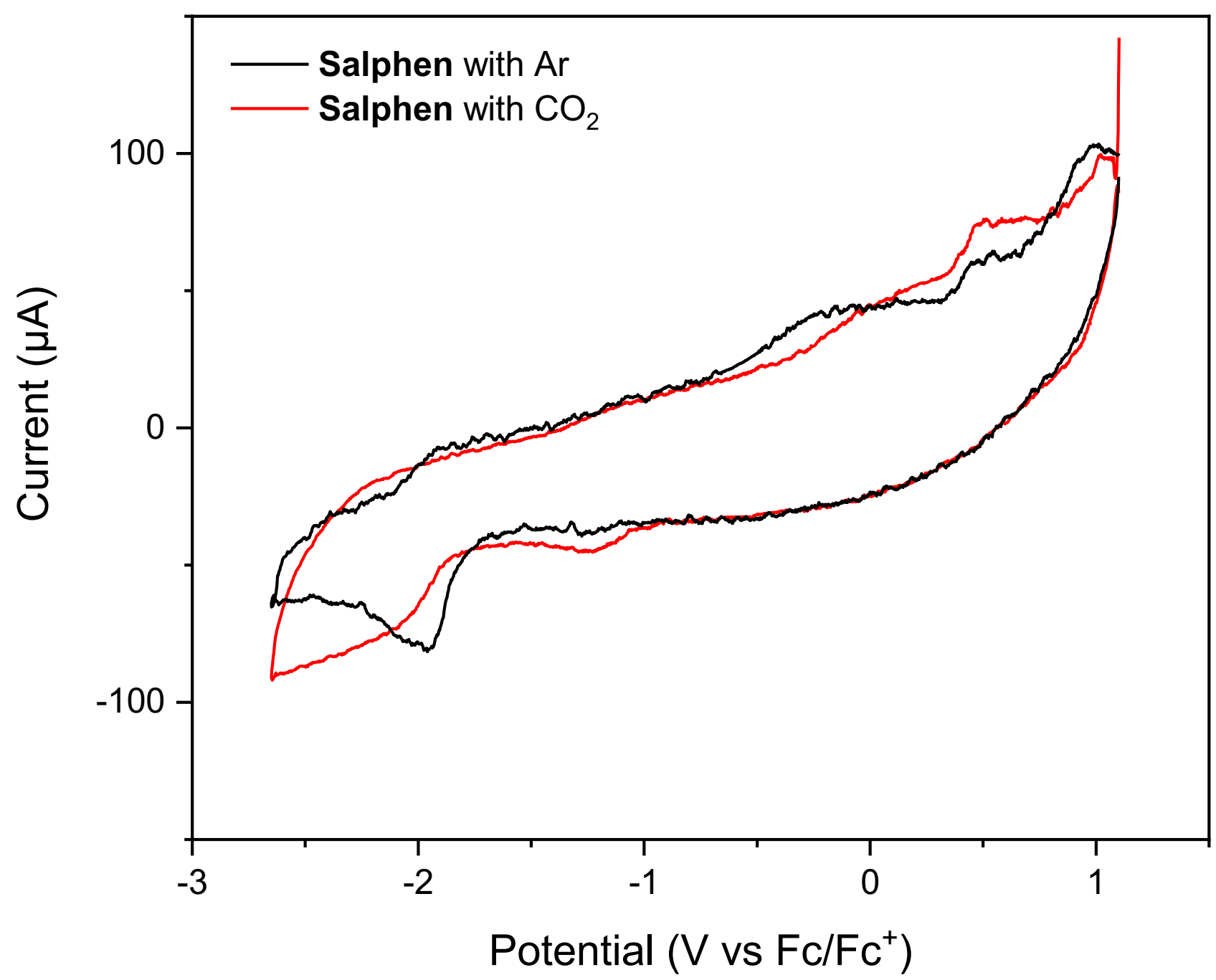

Figure S9. Cyclic voltammogram of 1.0 mM simple **salphen** in acetonitrile with 0.10 M TBAPF$_6$ supporting electrolyte at a scan rate of 100 mV/s, with an argon (black line) or CO_2 (red line) atmosphere.

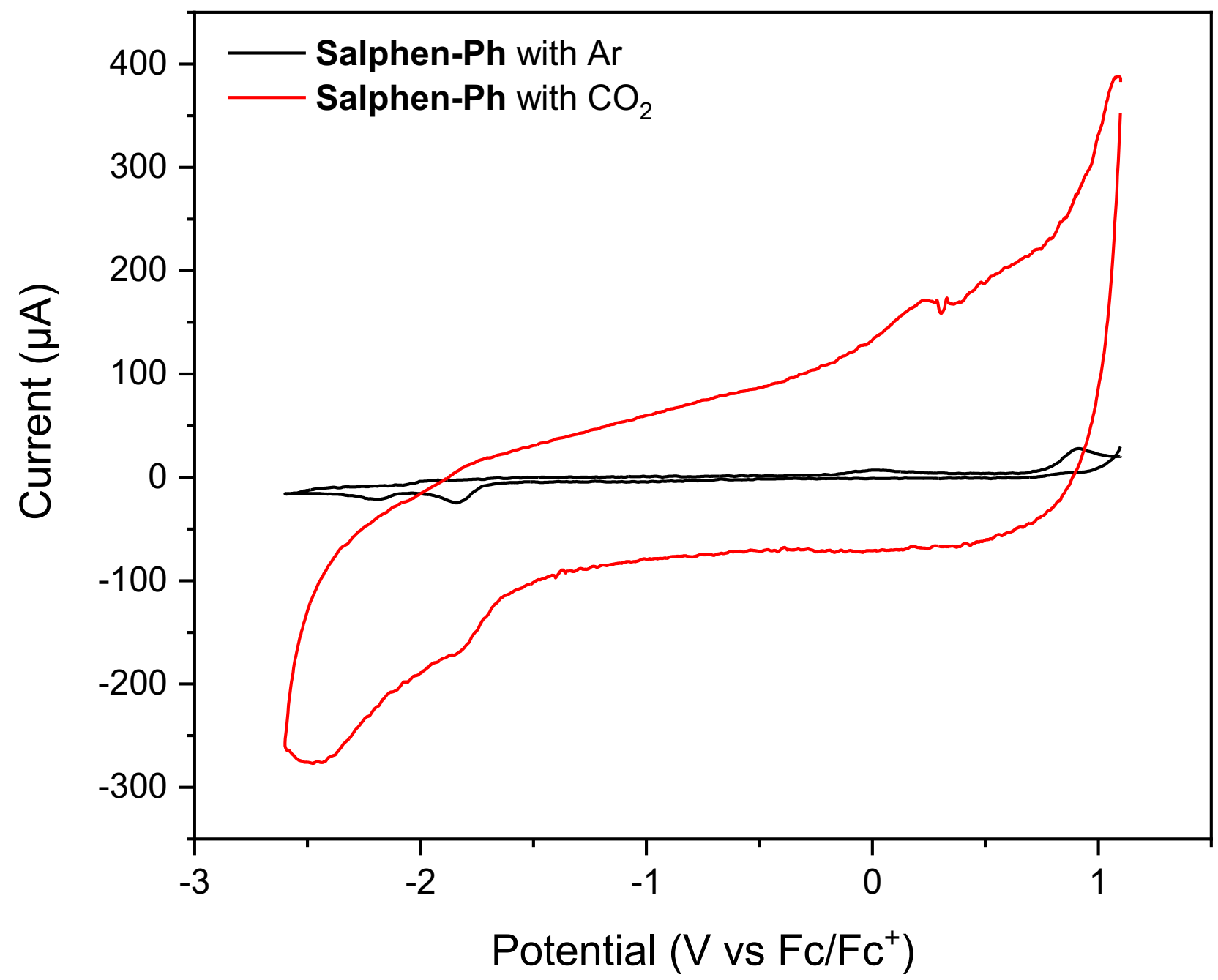

Figure S10. Cyclic voltammogram of 1.0 mM **salphen-Ph** in acetonitrile with 0.10 M $TBAPF_6$ supporting electrolyte at a scan rate of 100 mV/s, with an argon (black line) or CO_2 (red line) atmosphere.

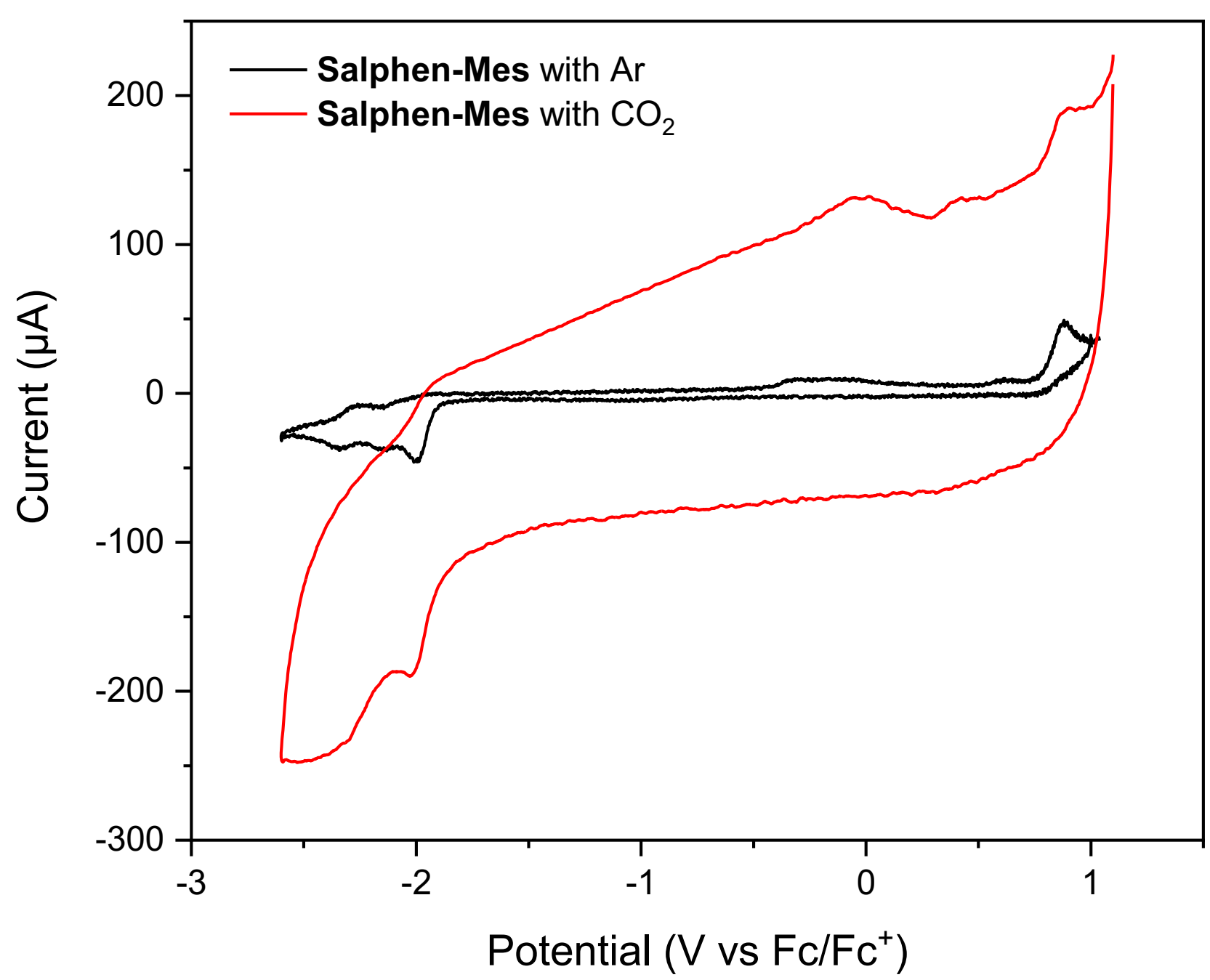

Figure S11. Cyclic voltammogram of 1.0 mM **salphen-Mes** in acetonitrile with 0.10 M $TBAPF_6$ supporting electrolyte at a scan rate of 100 mV/s, with an argon (black line) or CO_2 (red line) atmosphere.

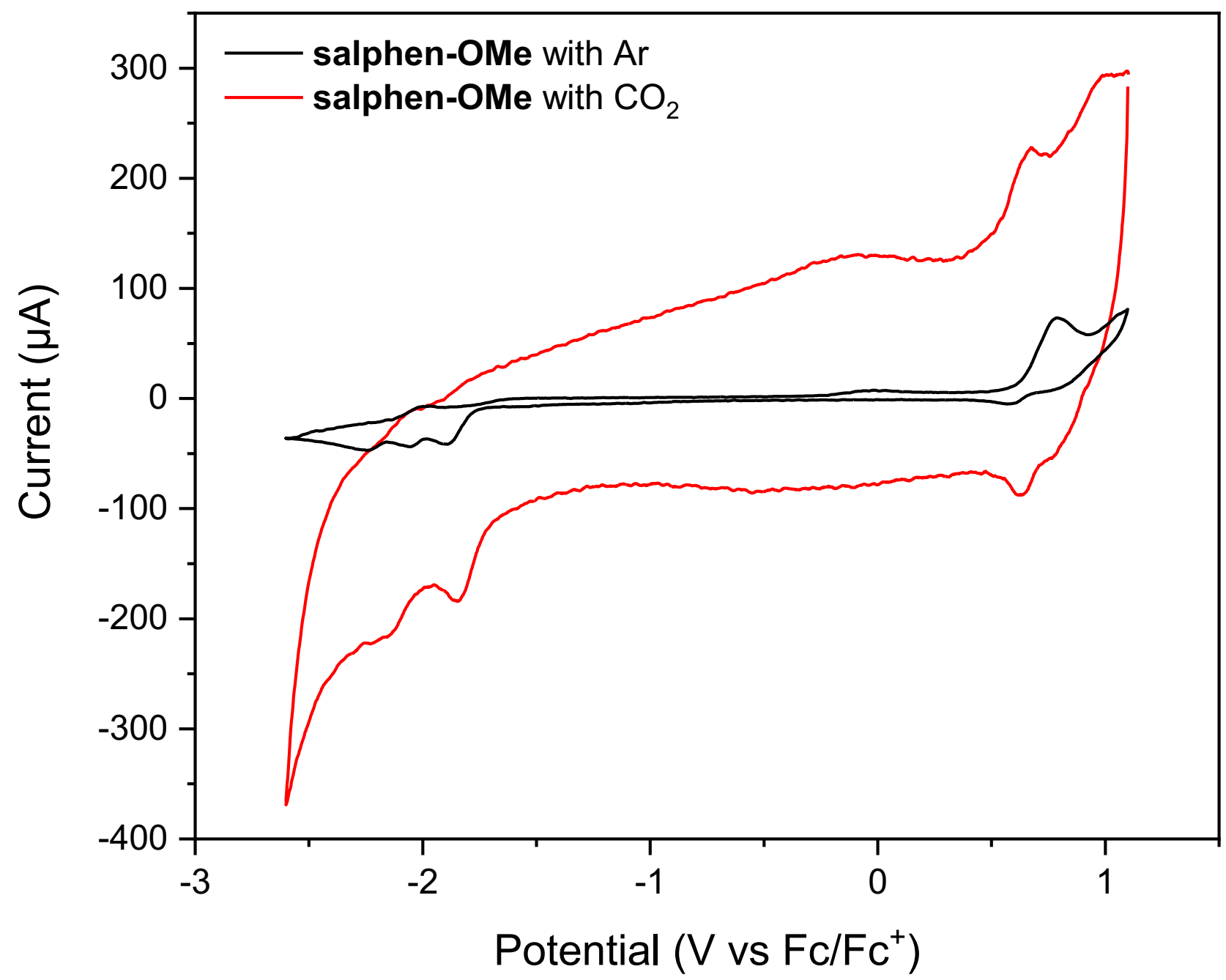

Figure S12. Cyclic voltammogram of 1.0 mM **salphen-OMe** in acetonitrile with 0.10 M $TBAPF_6$ supporting electrolyte at a scan rate of 100 mV/s, with an argon (black line) or CO_2 (red line) atmosphere.

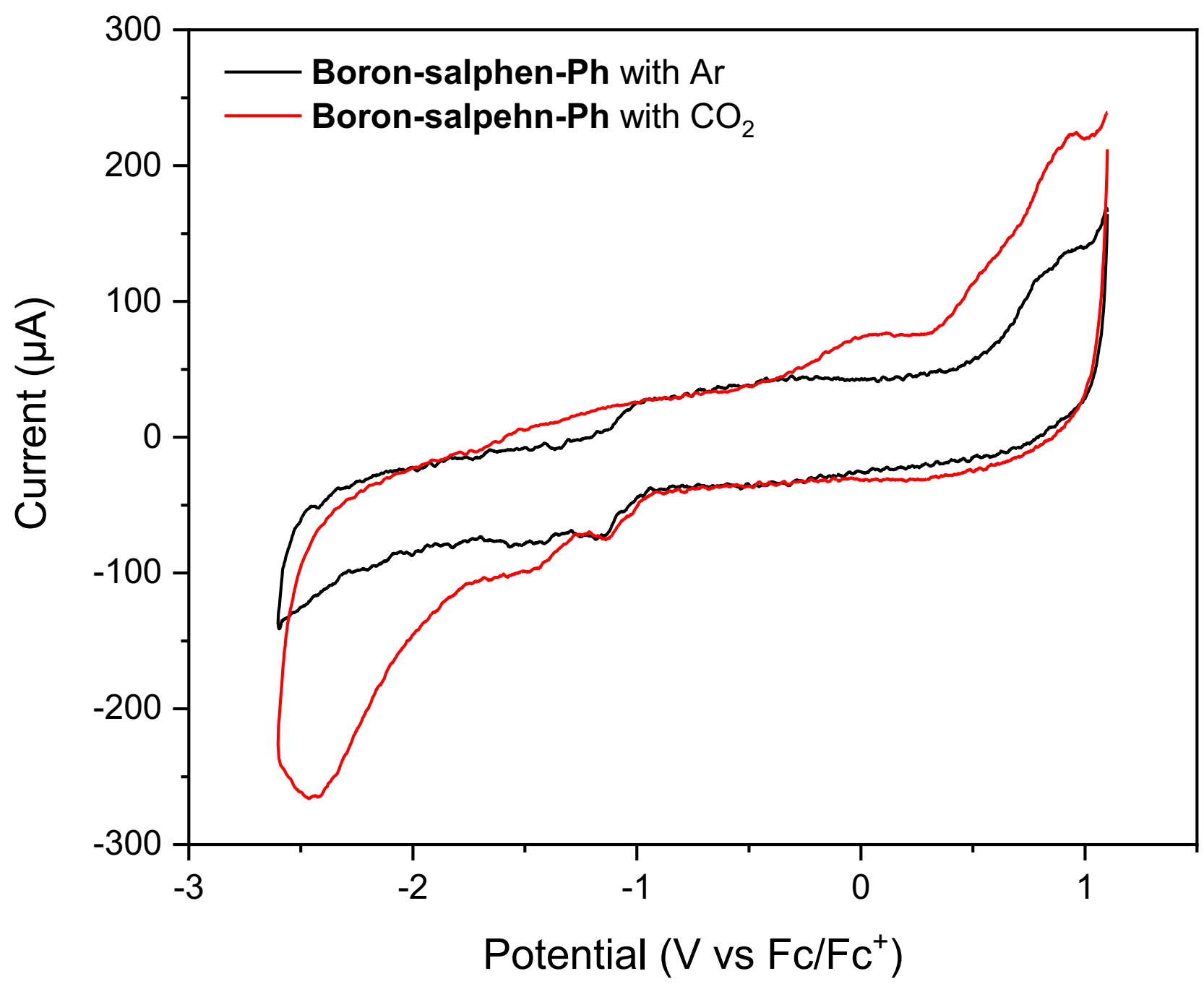

Figure S13. Cyclic voltammogram of 1.0 mM **boron-salphen-Ph** in acetonitrile with 0.10 M $TBAPF_6$ supporting electrolyte at a scan rate of 100 mV/s, with an argon (black line) or CO_2 (red line) atmosphere.

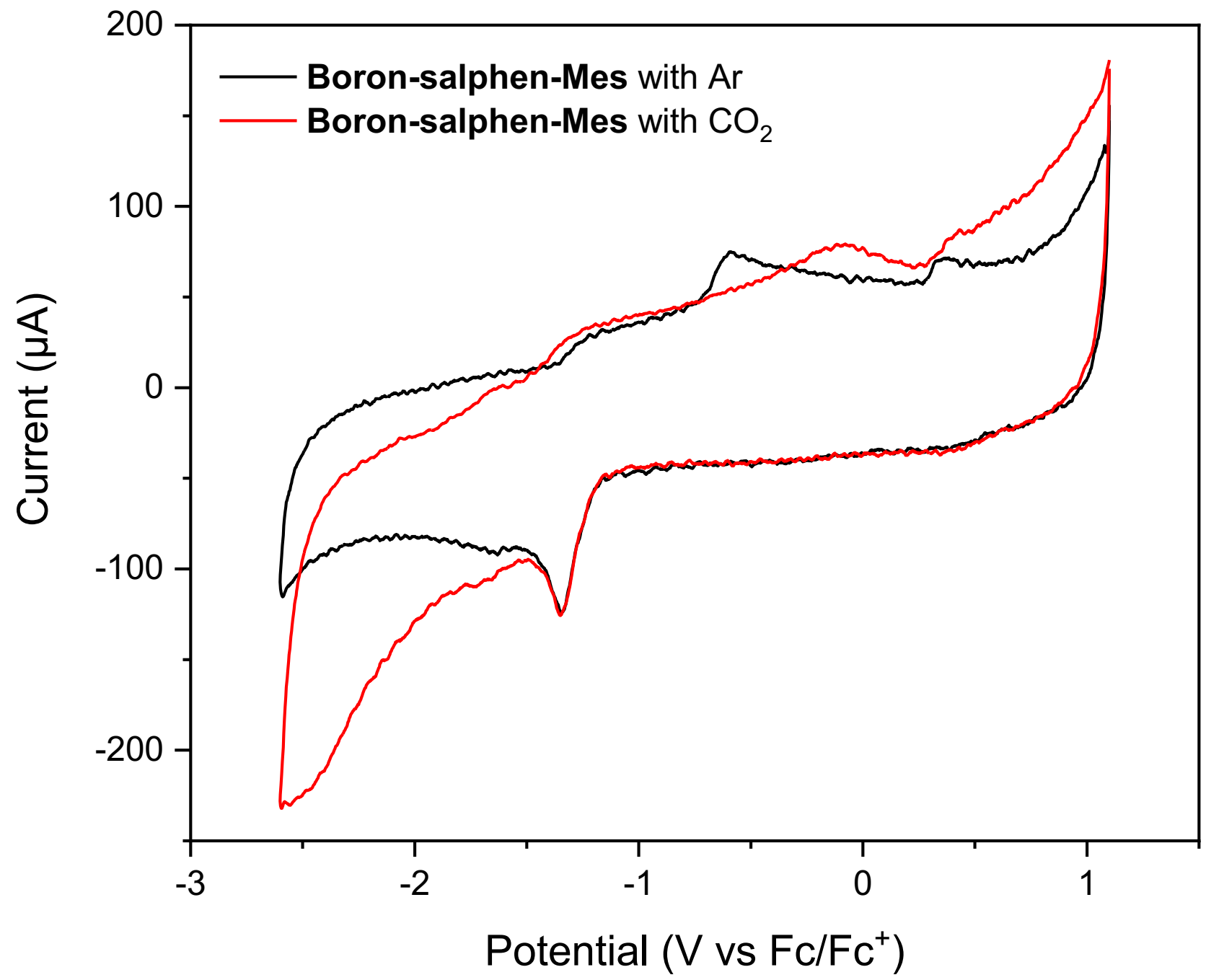

Figure S14. Cyclic voltammogram of 1.0 mM **boron-salphen-Mes** in acetonitrile with 0.10 M $TBAPF_6$ supporting electrolyte at a scan rate of 100 mV/s, with an argon (black line) or CO_2 (red line) atmosphere.

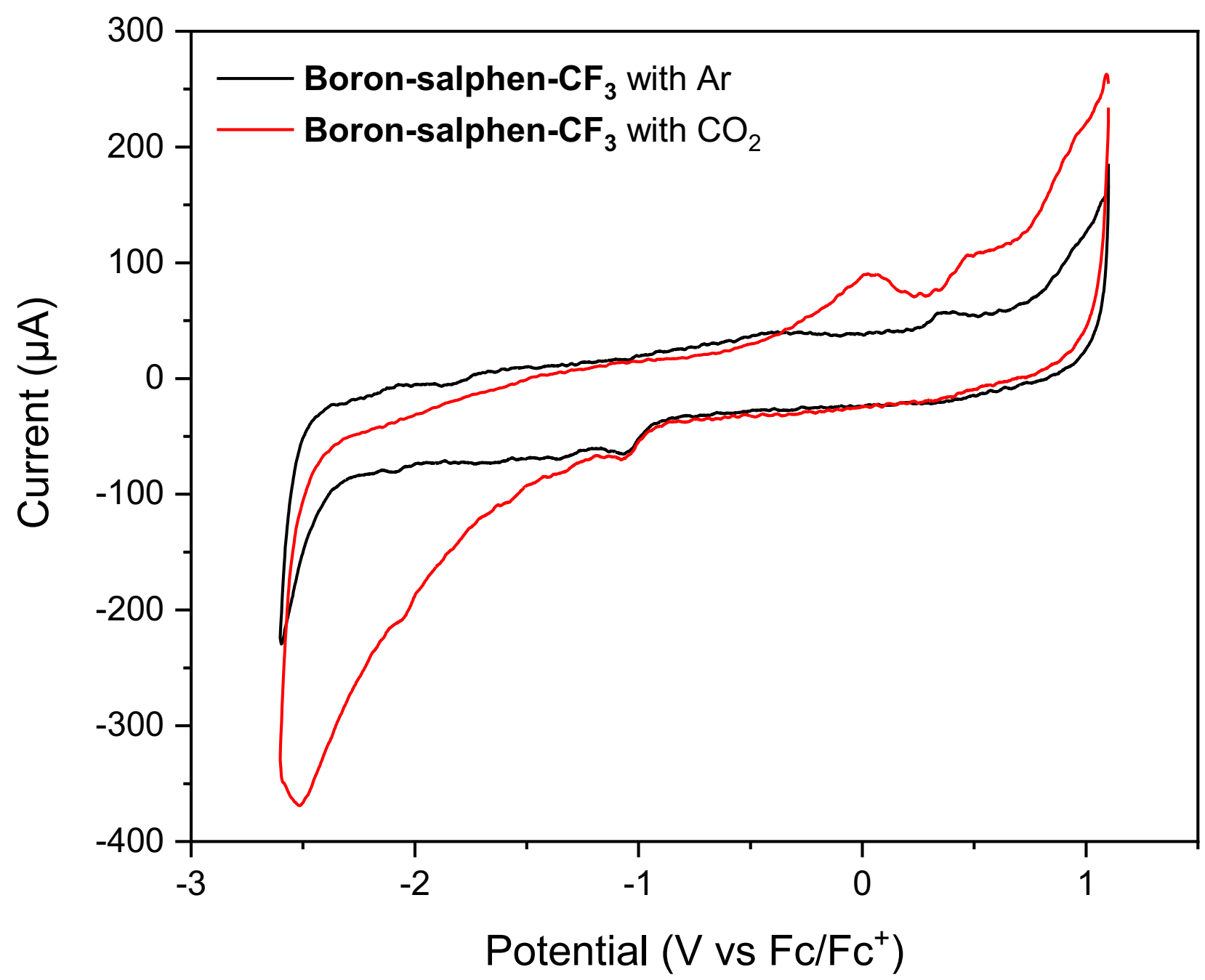

Figure S15. Cyclic voltammogram of 1.0 mM **boron-salphen-CF_3** in acetonitrile with 0.10 M $TBAPF_6$ supporting electrolyte at a scan rate of 100 mV/s, with an argon (black line) or CO_2 (red line) atmosphere.

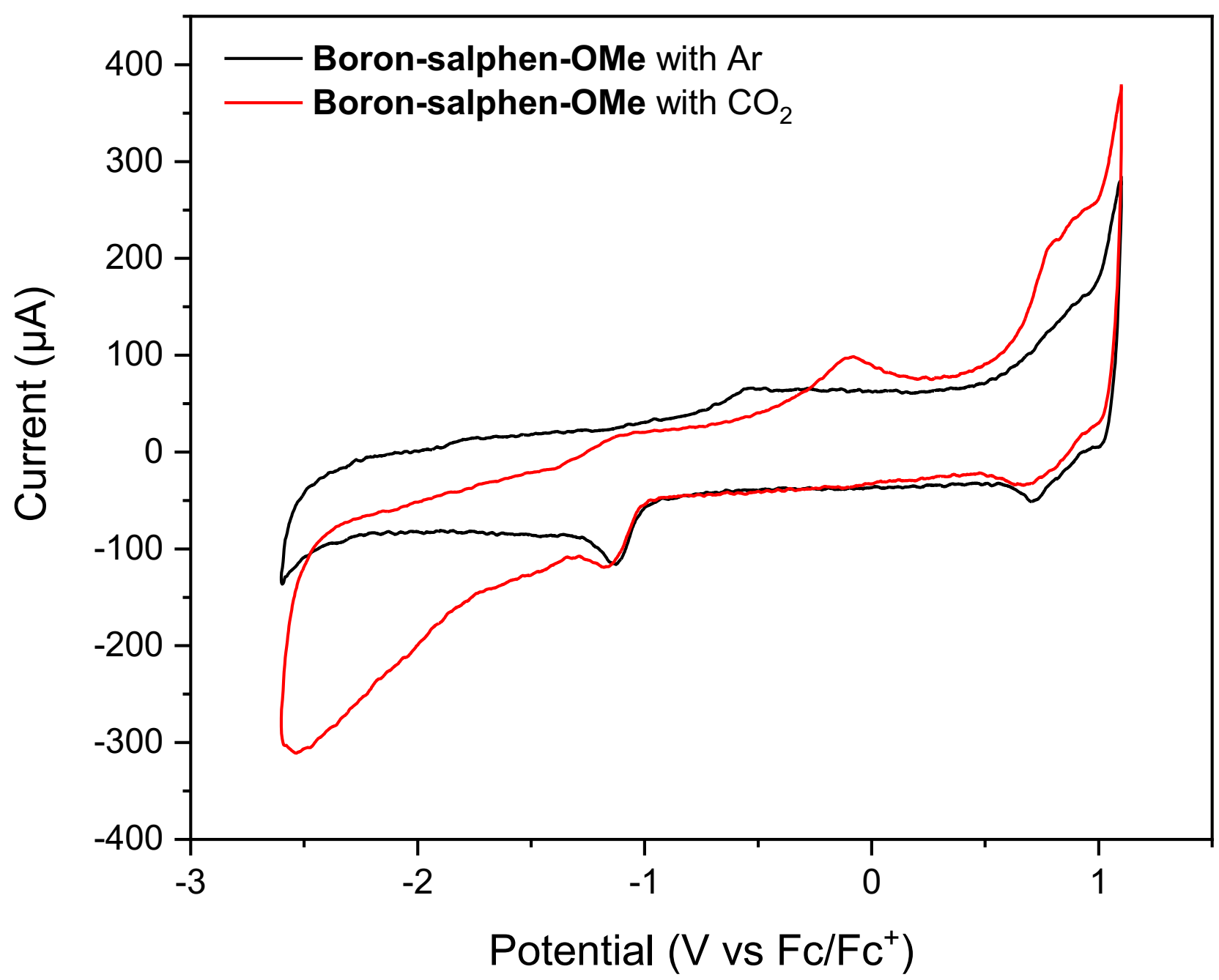

Figure S16. Cyclic voltammogram of 1.0 mM **boron-salphen-OMe** in acetonitrile with 0.10 M $TBAPF_6$ supporting electrolyte at a scan rate of 100 mV/s, with an argon (black line) or CO_2 (red line) atmosphere.

4. CO_2 electroreduction application

Chronoamperometry was performed at various potentials using a Palmsens4 potentiostat in a one-pot cell with glassy carbon (5 mm) as working electrode, Pt wire as counter electrode and $Ag/AgNO_3$ as reference electrode. Each measurement was performed in MeCN with 0.10 M $TBAPF_6$ as supporting electrolyte containing 1.0 mM of salen or salphen-based catalysts under constant CO_2 or Ar purge. In order to quantify the products of the reaction, gas chromatography was performed with N_2 as carrier gas under constant flow of CO_2 or Ar, in presence of a certain amount of a proton source. Gaseous products were quantified through the integration of peak areas corresponding to the products (H_2 and CO), which were measured using a series of calibration curves. The GC was calibrated using calibration gas (Air Liquide, 1% H_2, 1% CO, 1% CH_4) and diluted with Ar gas to different ratios using a mass flow controller. The following abbreviations are used in the next figures: Faradaic efficiency, FE; partial current density, PCD; open circuit potential, OCP; CO_2 electroreduction, CO_2RR; phenol, PhOH; trifluoroethanol, TFE; acetic acid, AcOOH.

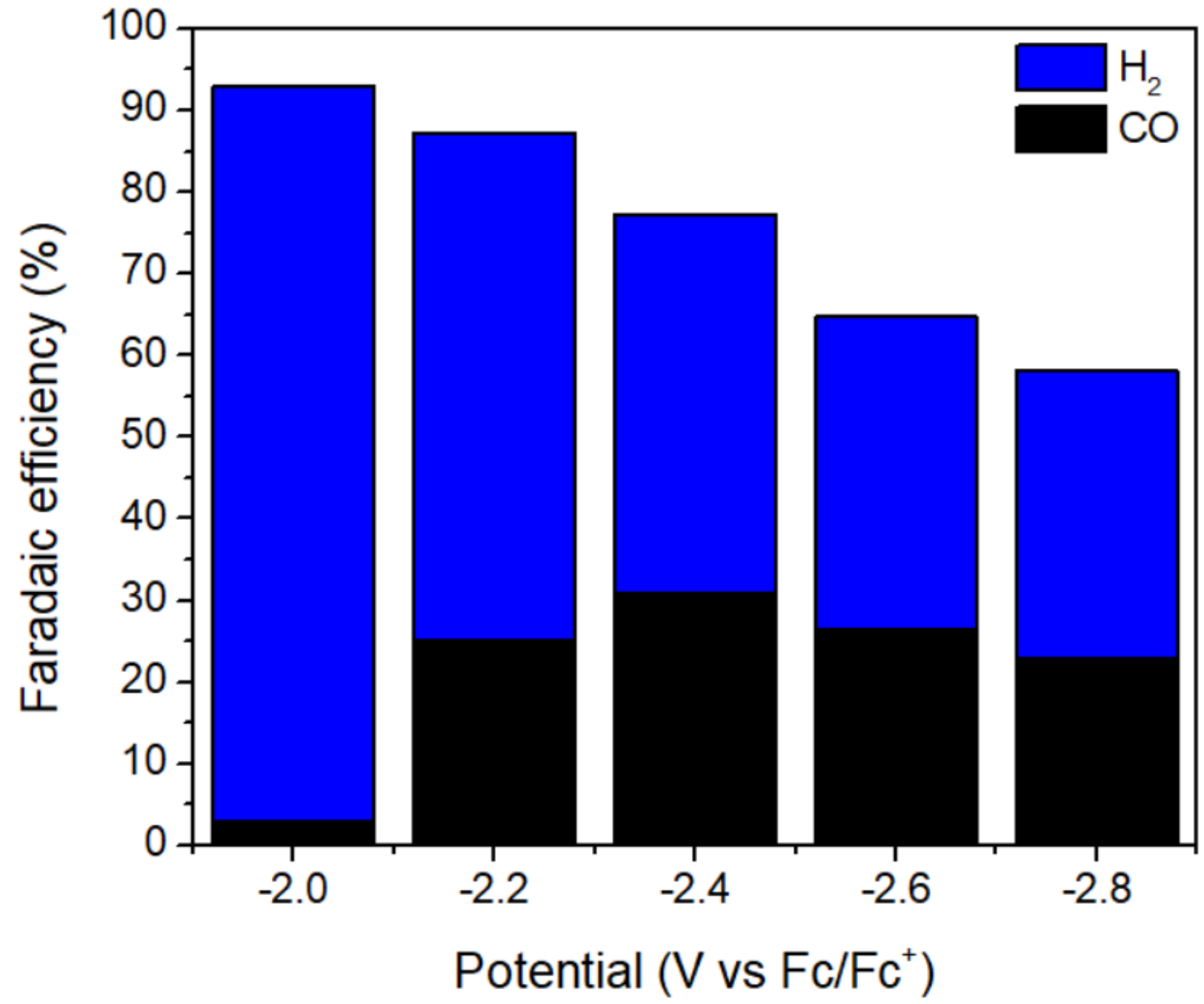

Figure S17. Total FE of all detected products from CO_2RR using 1.0 mM **salphen-CF$_3$** in acetonitrile with 0.10 M TBAPF$_6$ and 5% v/v water.

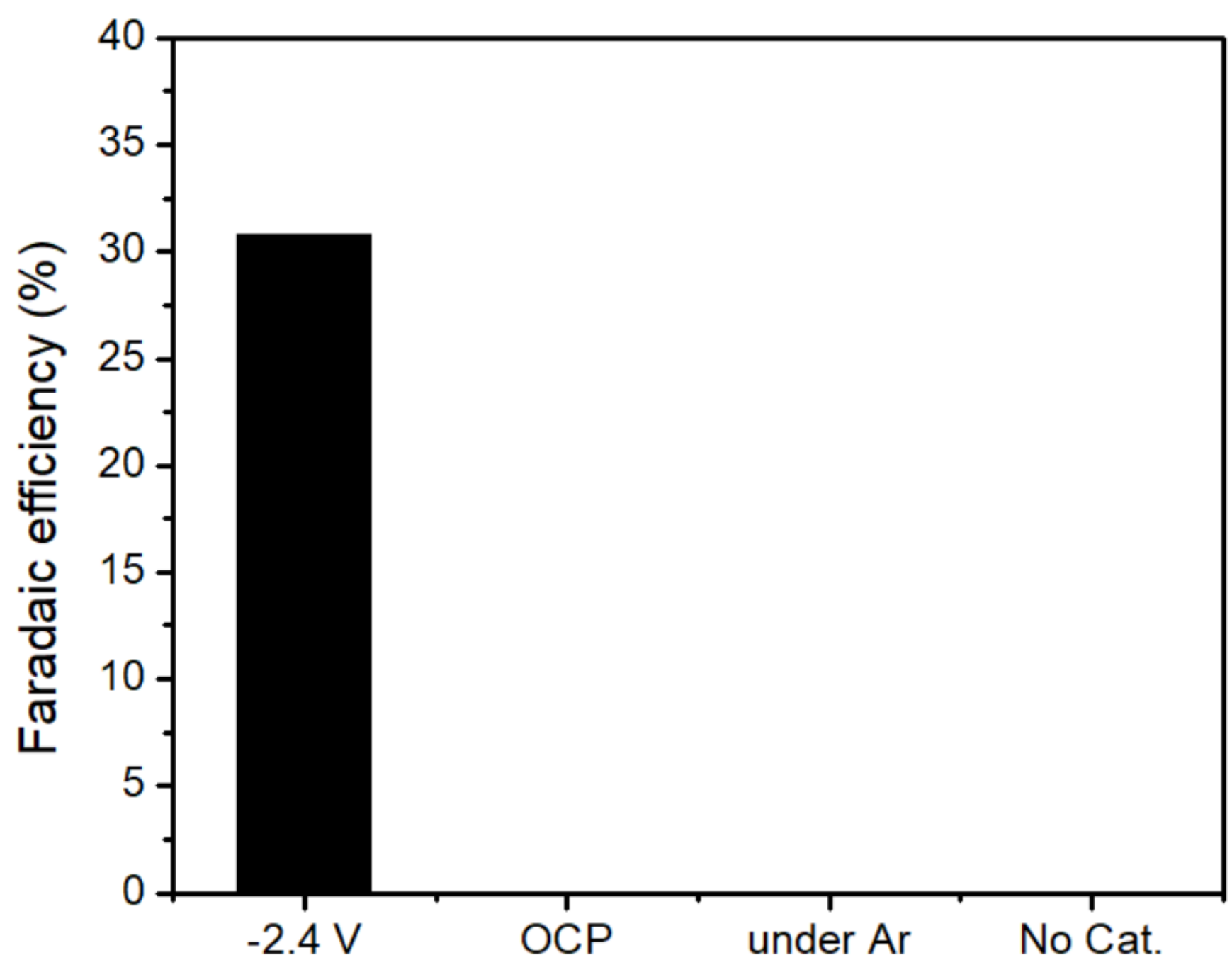

Figure S18. FE for CO production using 1.0 mM **salphen-CF$_3$** compared to blank experiments including OCP, under argon and in the absence of catalyst

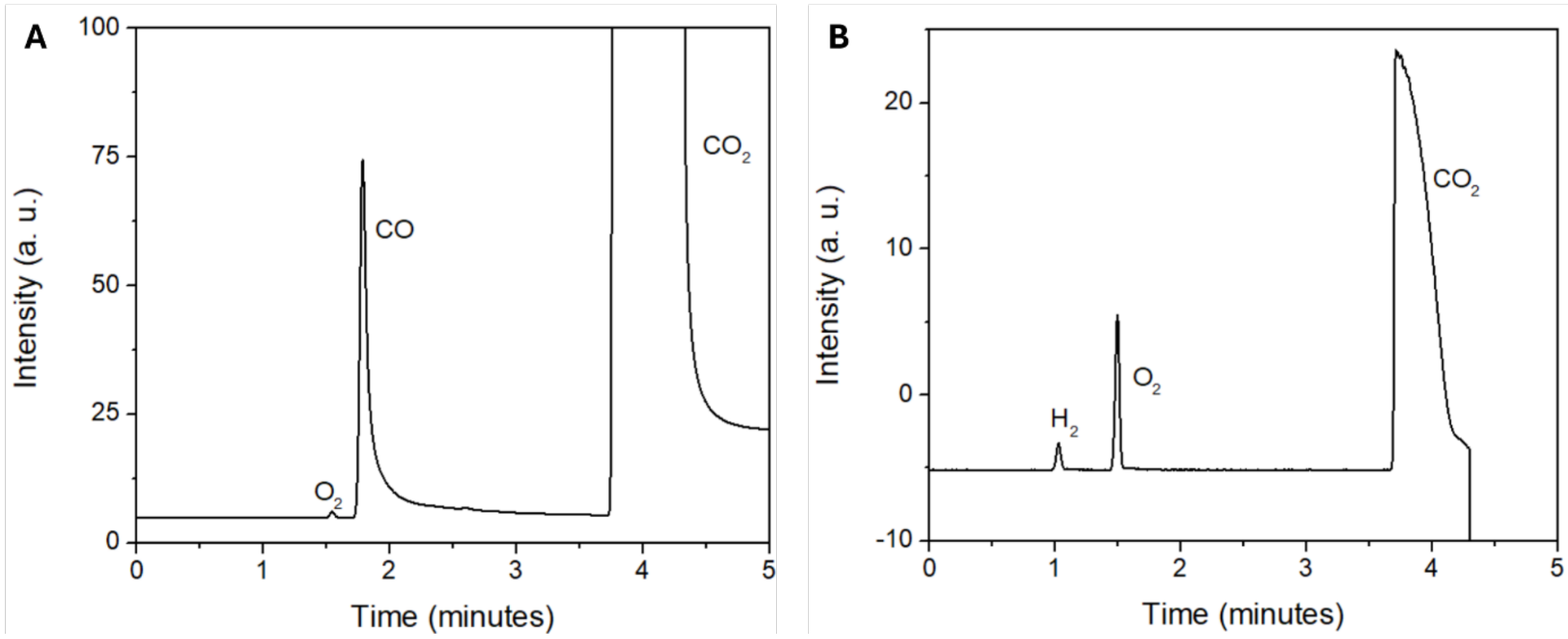

Figure S19. Gas chromatograms showing the formation of CO (**A**) and H_2 (**B**) from CO_2RR using 1.0 mM of **salphen-CF_3** at -2.4 V.

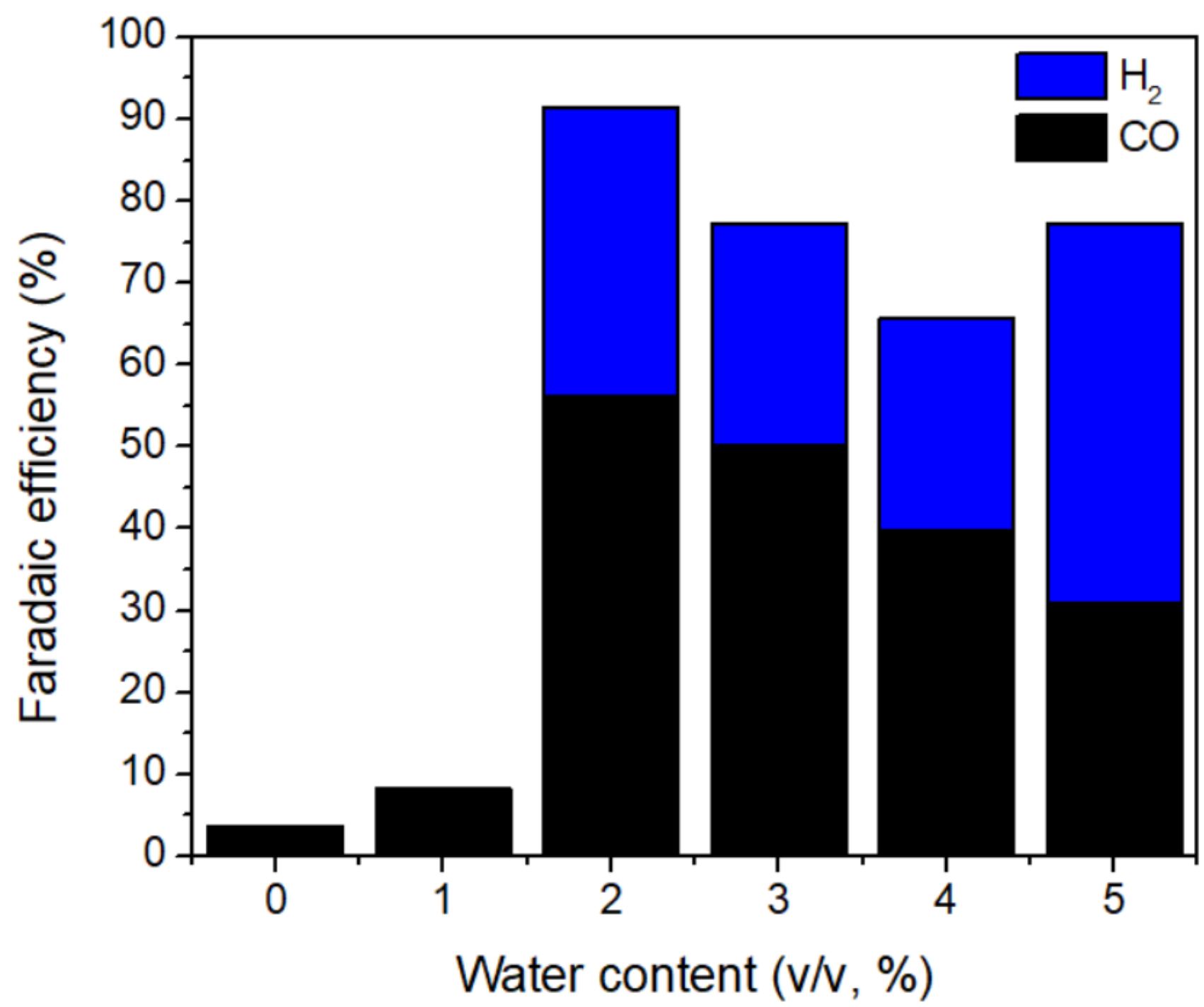

Figure S20. Total FE of all detected products from CO_2RR using 1.0 mM **salphen-CF_3** in acetonitrile with 0.10 M $TBAPF_6$ at varying concentrations of water.

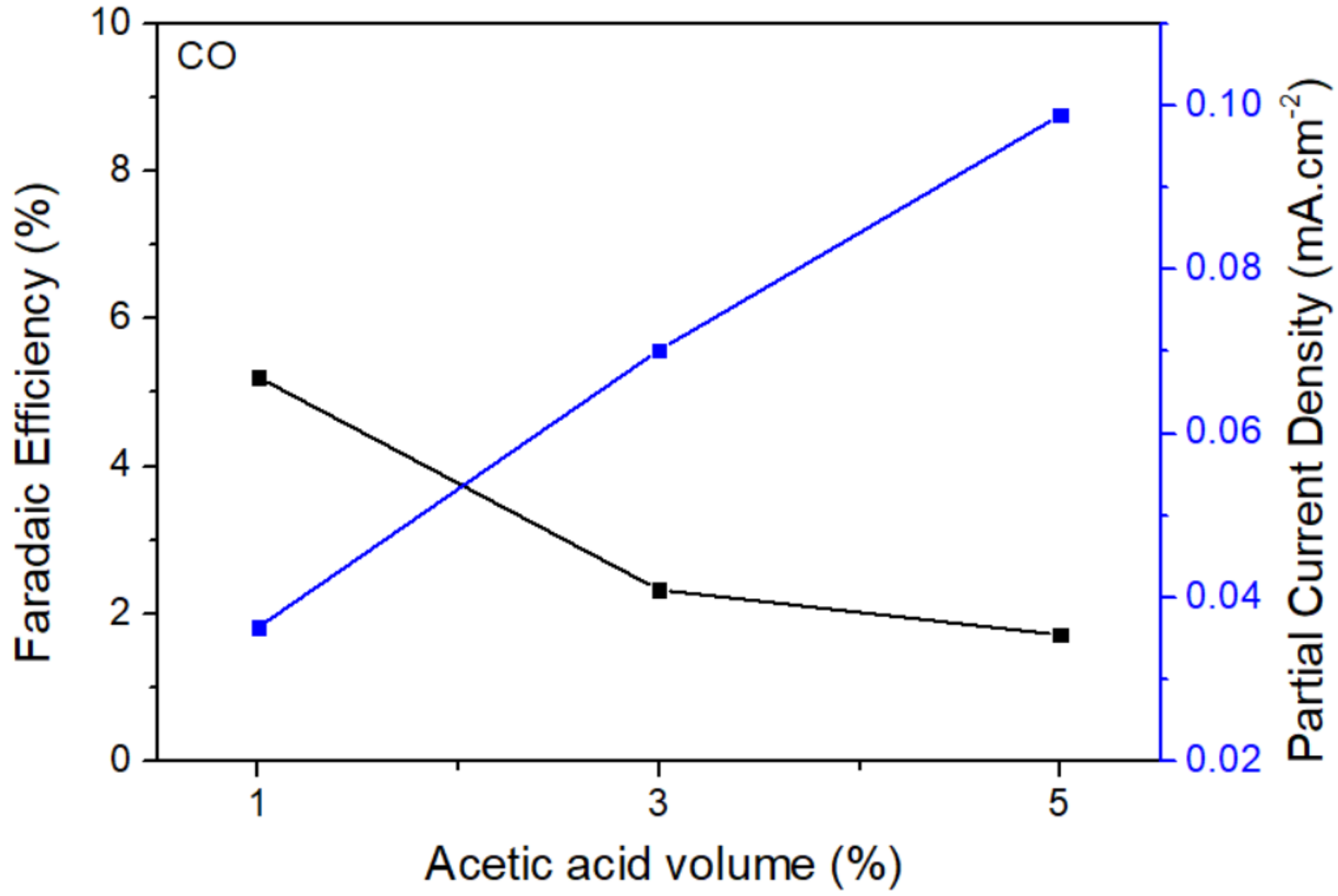

Figure S21. FE and PCD for CO production at different volume percents of acetic acid.

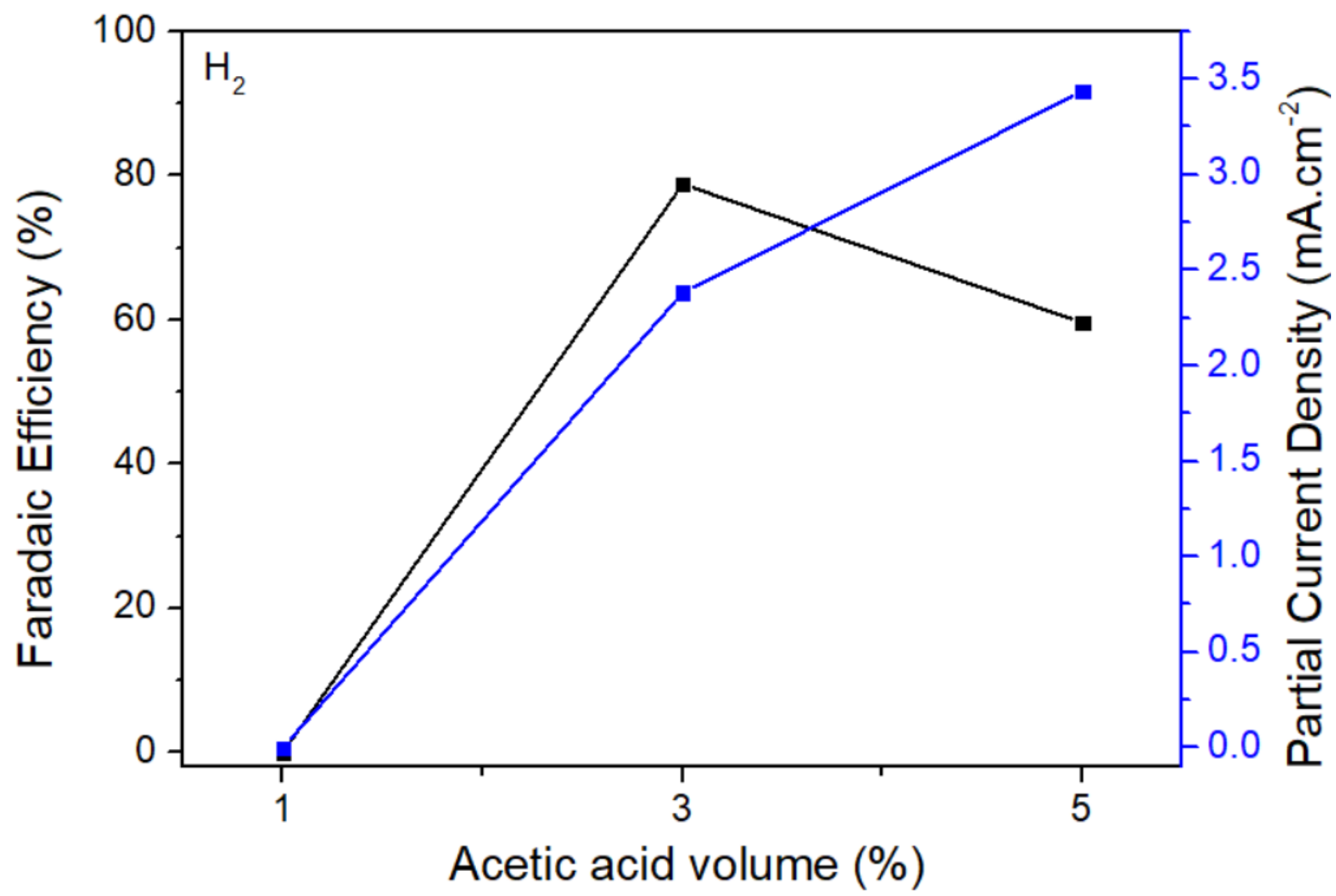

Figure S22. FE and PCD for H_2 production at different volume percents of acetic acid.

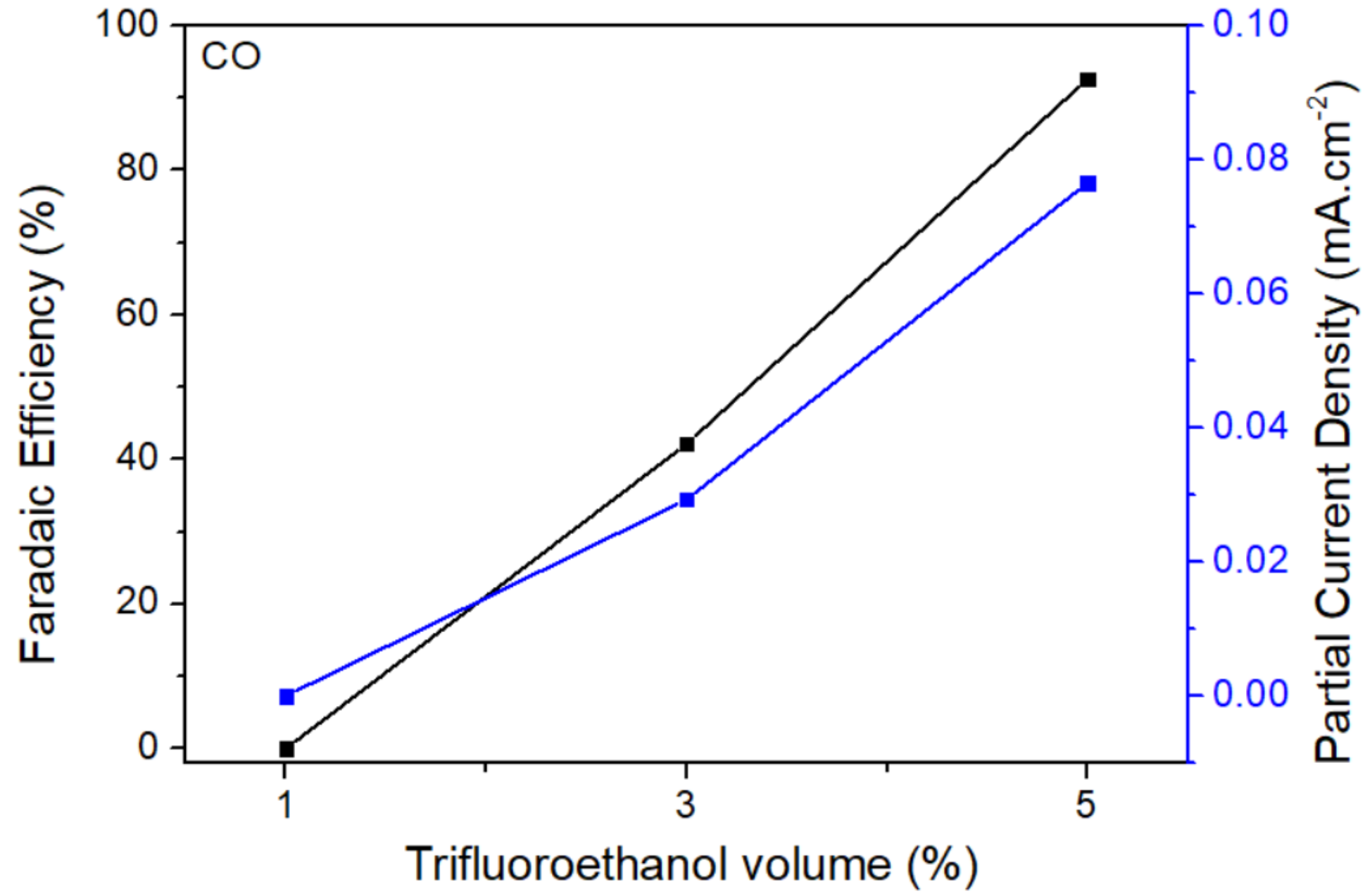

Figure S23. FE and PCD for CO production at different volume percents of TFE.

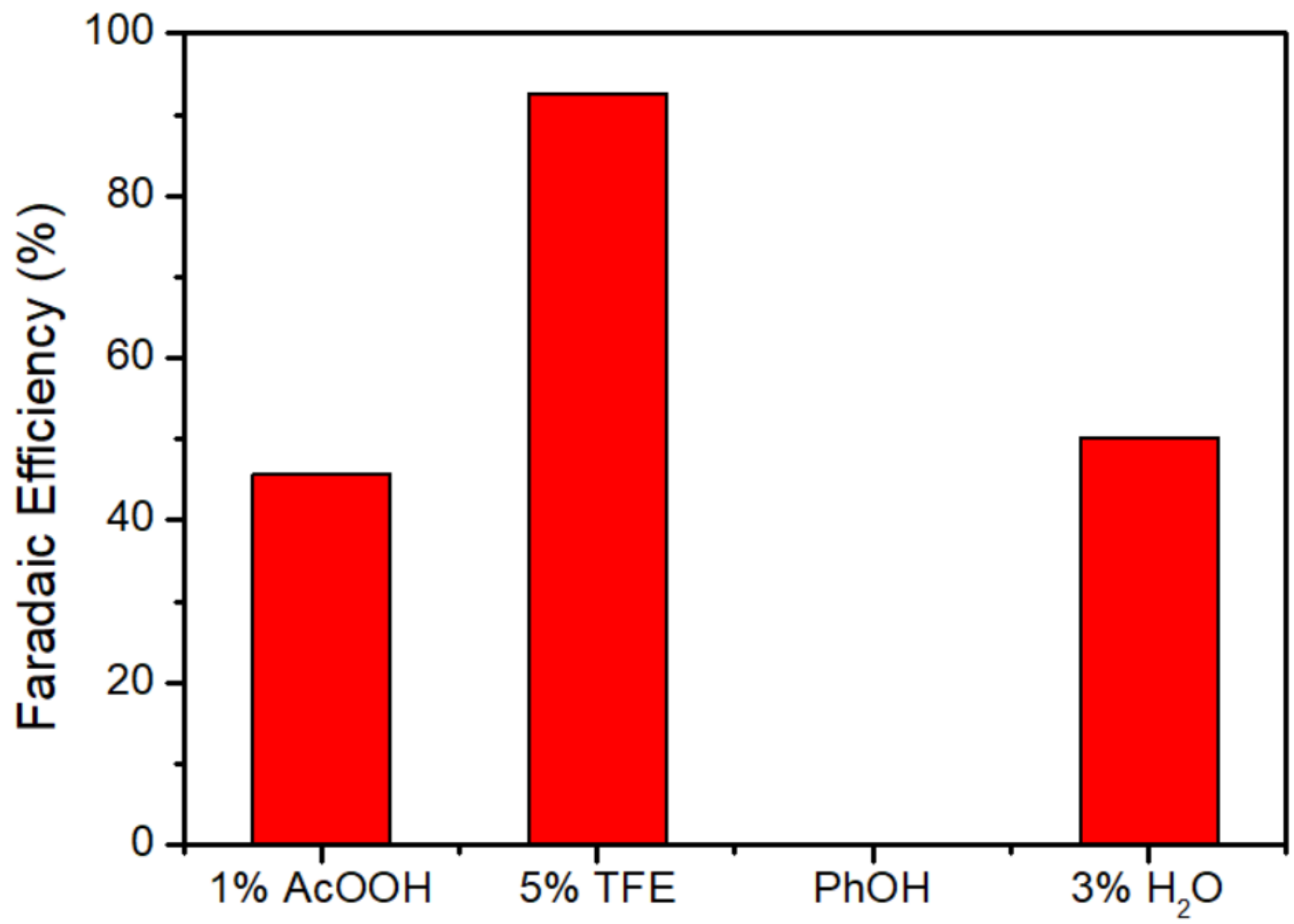

Figure S24. FE comparison for CO production optimised concentrations of different proton sources.

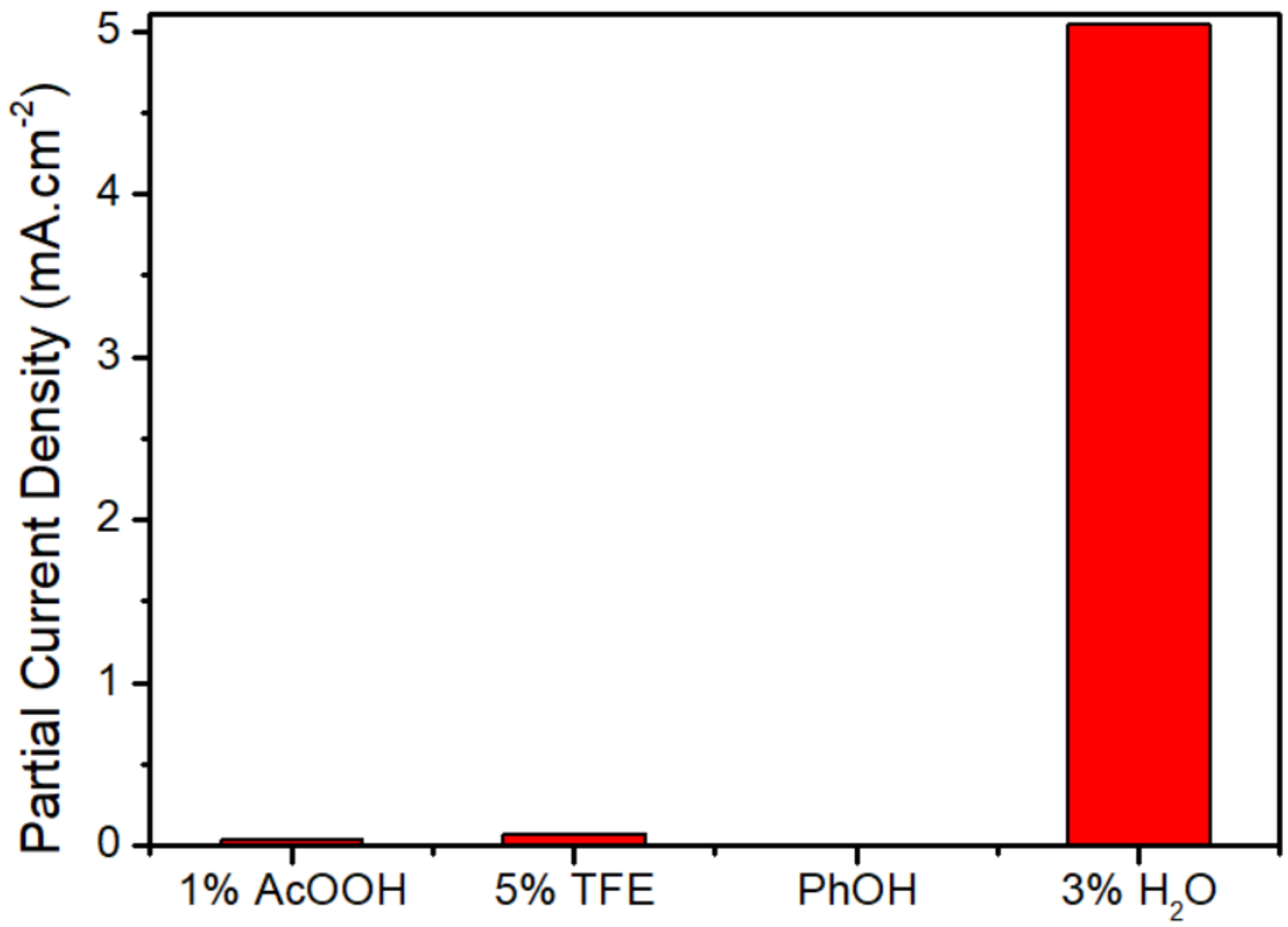

Figure S25. PCD comparison for CO production optimised concentrations of different proton sources.

Long-term electrolysis was conducted at −2.4 V vs Fc/Fc+ in MeCN containing 0.10 M $TBAPF_6$ supporting electrolyte and 3% v/v water under a CO_2 atmosphere. Continuous CO production was observed over the course of the experiment, corresponding to a turnover number of 103 with an overall CO FE of 41%. UV/Vis spectra recorded before and after electrolysis show minimal changes in the ligand absorption bands, indicating that the **salphen-CF_3** framework remains largely intact under the applied conditions.

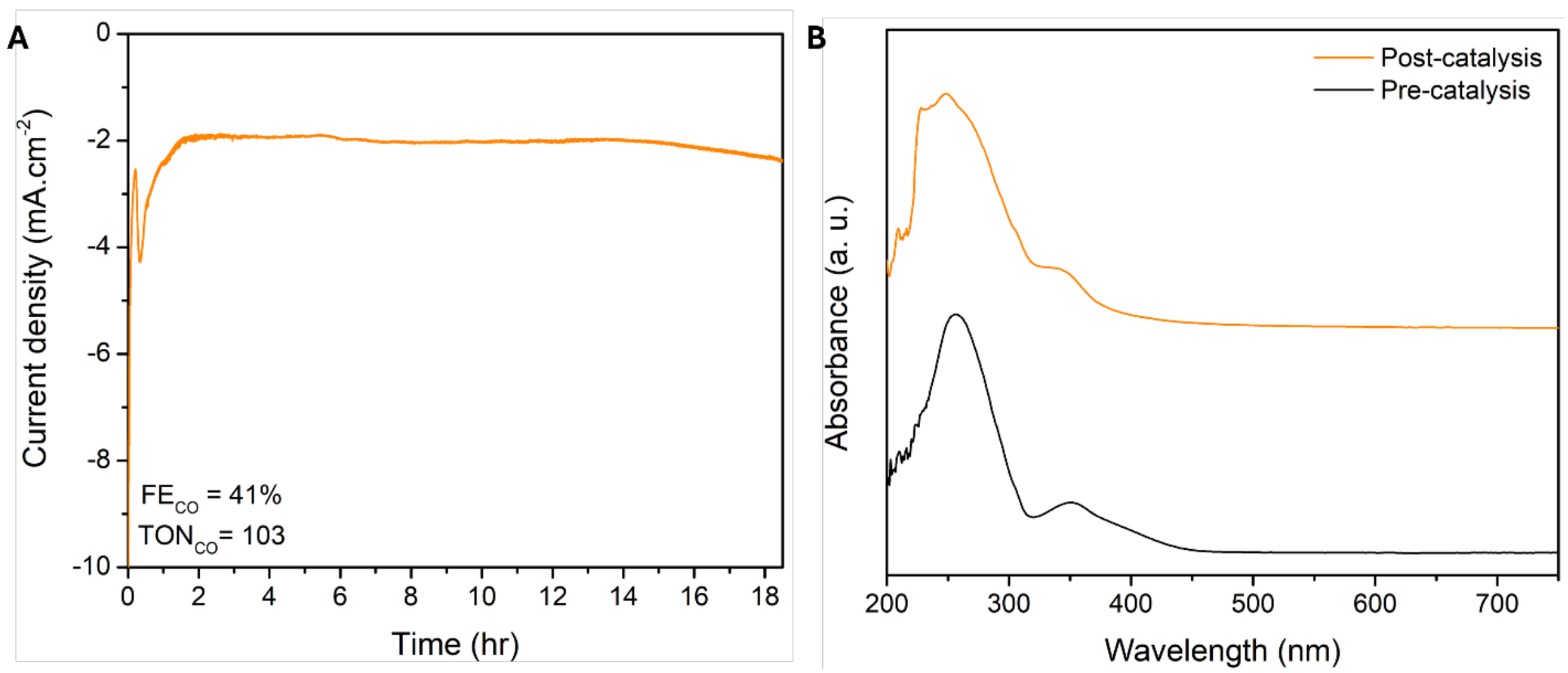

Figure S26. (**A**) Long-term electrolysis for CO_2 electroreduction at −2.4 V using 0.10 mM **salphen-CF_3** in MeCN with 0.10 M $TBAPF_6$. (**B**) UV/Vis spectra of **salphen-CF_3** before and after long-term electrolysis.

Table S1. Comparison of representative carbon-based, framework, and molecular CO_2 reduction electrocatalysts, including key performance metrics (FE, PCD, TON) and active- site definition, highlighting differences in mechanistic understanding.

System	Catalyst Type	Active Site Definition	Major Product	FE (%)	PCD ($mA.cm^{-2}$)	TON	Mechanistic Insight
**N-doped graphene quantum dots[61]**	Heterogeneous carbon nanomaterial	N-doped carbon sites	C_2H_4	CO: 40	CO: 23	N/A	No direct mechanistic data
**N-doped nanoporous carbon–CNT[62]**	Heterogeneous carbon composite	Carbon atoms with Lewis basicity next to pyridinic N	Formate	Formate: 80	Formate: 8.8	N/A	No direct mechanistic data
**Co-porphyrin COF[63]**	Macromolecular framework / heterogeneous	Periodic Co porphyrin sites	CO	CO: 90	CO: 1	290,000 per Co active site	Moderate; framework effects investigated by XAS
**Porphyrin systems[64]**	Molecular/framework hybrid systems	Well-defined metal porphyrins	CO / formate	Typically high	Moderate–high	Variable	Strong mechanistic understanding possible
**Salphen-CF_3 (this work)**	Molecular metal-free homogeneous catalyst	Structurally defined ligand-centered active site	CO	56	~9	103	*Operando* FTIR, DFT-supported ligand-centered intermediates

4.1. Mechanistic investigation*: In situ* attenuated total reflection IR spectroscopy (*in situ* ATR-IR) and kinetic isotope effect (KIE)

ATR-IR measurements were collected using a Bruker Tensor II FTIR spectrometer with an MCT detector and a Pike Technologies VeeMAX III ATR accessory. The spectra were collected with 4 cm^{-1} resolutions at an incidence angle of ~60°. To obtain the spectra, Au was sputtered onto the ATR crystal to create an Au film, which acts as the working electrode. A solution of carbon nanotubes was then dropped onto the surface of the Au film to mimic that of the carbon electrode used during electrolysis. $Ag/AgNO_3$ and Pt wire were also used as the reference and counter electrodes. For the measurements, the electrolyte employed was MeCN with 0.10 M $TBAPF_6$ and 1.0 mM of **salphen-CF_3** which was purged with either Argon or CO_2 gas.

For KIE investigation, general conditions are reported in **Section 1.3** *(Electrochemical reaction setup*) were applied, with the addition of water or deuterium oxide (3% by volume) and **salphen-CF_3** (1.0 mM).

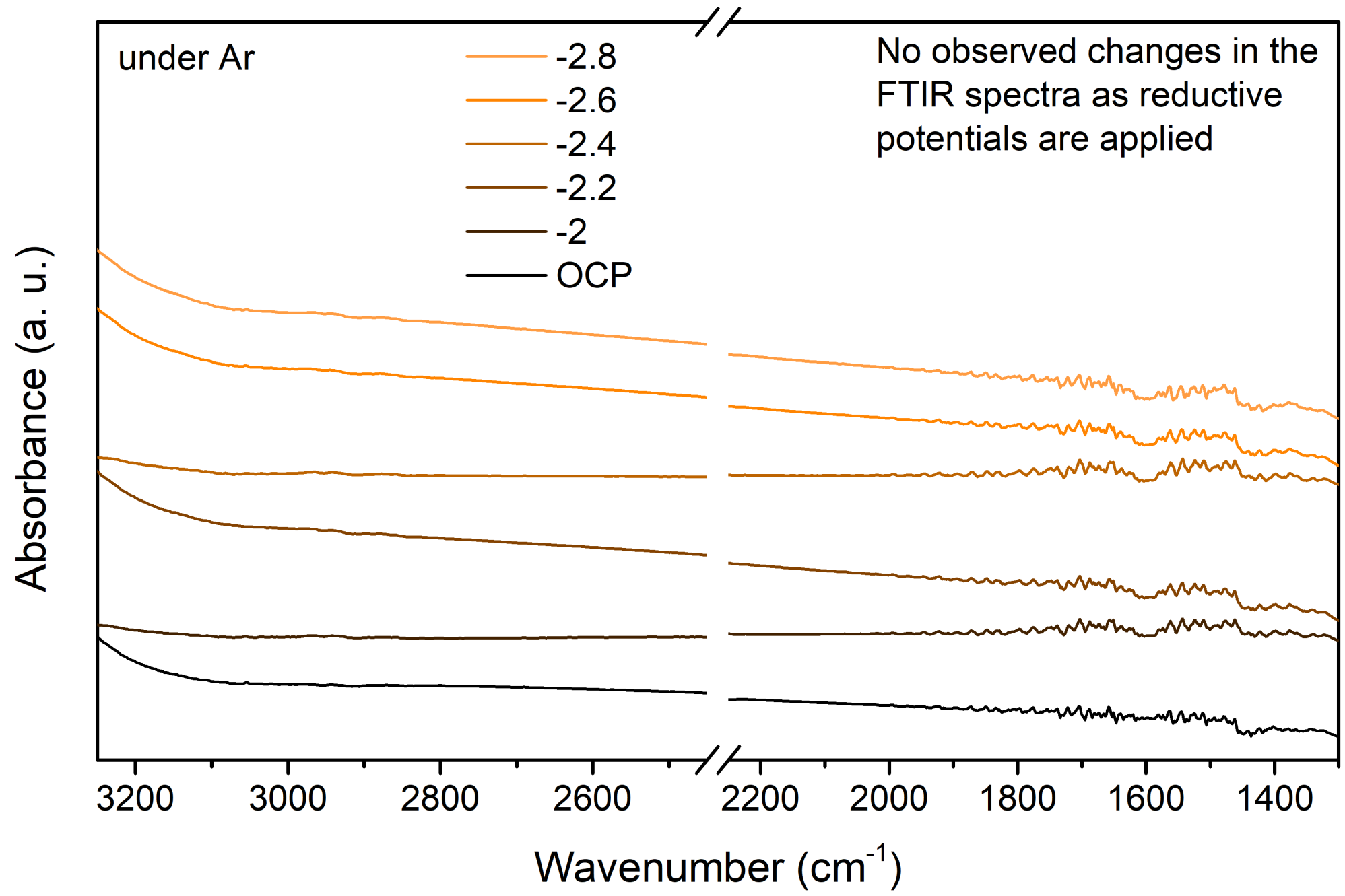

Figure S27. *In situ* FTIR spectra collected in acetonitrile containing 0.10 M $TBAPF_6$ and 3% v/v water saturated with argon, using 1.0 mM **salphen-CF_3** as catalyst.

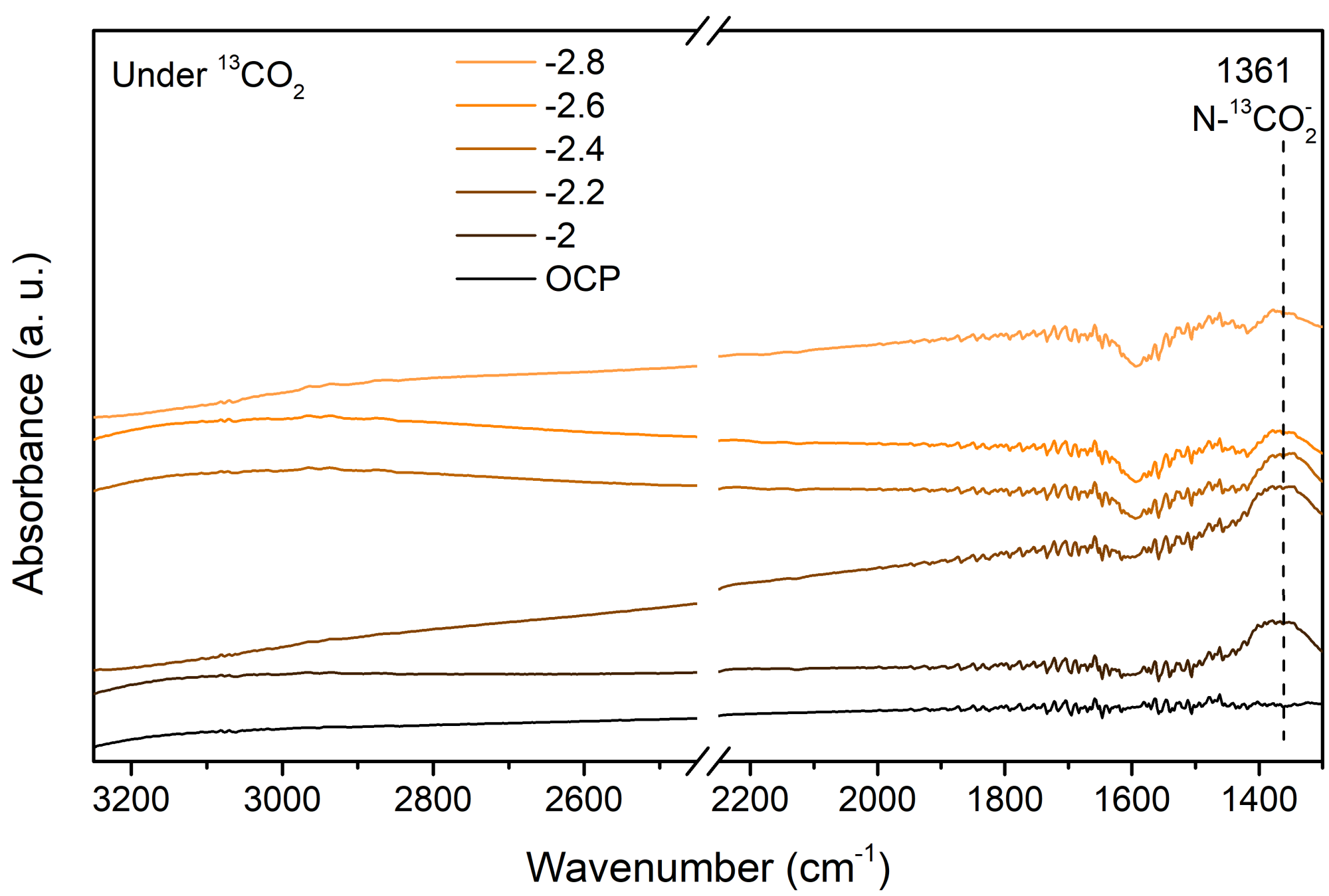

Figure S28. *In situ* FTIR spectra collected in acetonitrile containing 0.10 M $TBAPF_6$ and 3% v/v water saturated with $^{13}CO_2$, using 1.0 mM **salphen-CF_3** as catalyst.

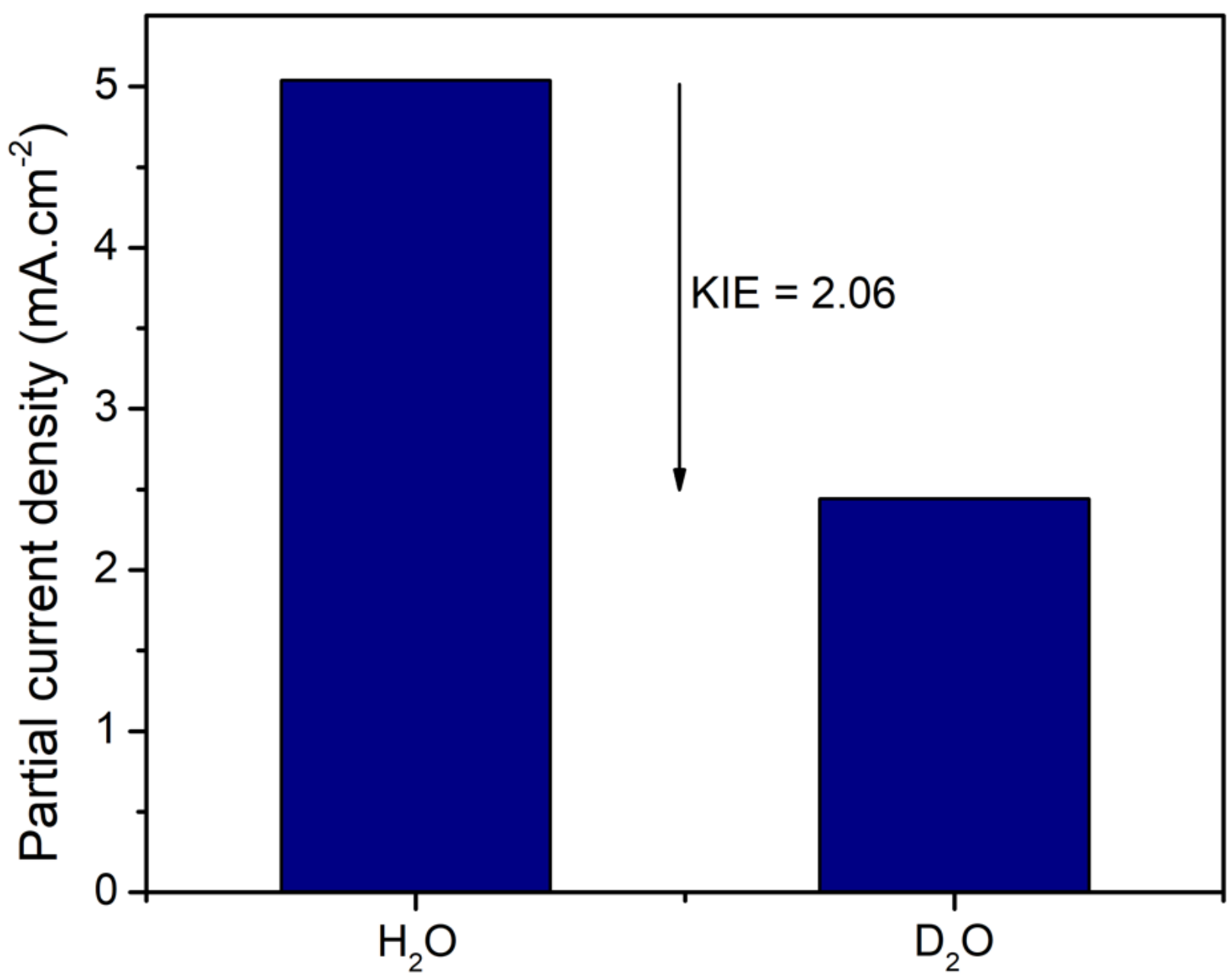

Figure S29. Kinetic isotope effect (KIE) experiments comparing the partial current density for CO production from CO_2RR with 3% H_2O or D_2O at an applied potential of −2.4 V vs Fc/Fc^+.

5. Spectroscopic Studies

5.1. General considerations

All measurements were conducted following the standard operating procedure reported below.

Stock solutions of all compounds were prepared. The stock solution was immediately diluted further in a cuvette. UV/Vis-measurements for the estimation of molar absorption coefficients ε were carried out in THF at room temperature from 250 nm to 700 nm. Respective molar absorption coefficients were determined via linear regression analysis by plotting the absorbance of a quintuple dilution series against the sample concentration. Emission and excitation spectra were measured with solutions of compounds in THF at room temperature. All spectra were recorded with a dwell time of 0.150 s (UV/Vis) or

0.200 s (emission) in data intervals of 1 nm with the bandwidths set to 1.0 nm (excitation) and 2.0 nm (emission). Photoluminescence lifetime spectra were obtained in toluene at 77 K using an EPL-365 and EPL-450 picosecond pulsed laser diode at a repetition frequency of 2 MHz.

5.2. UV/Vis spectra and determination of molar absorption coefficients (*ε*)

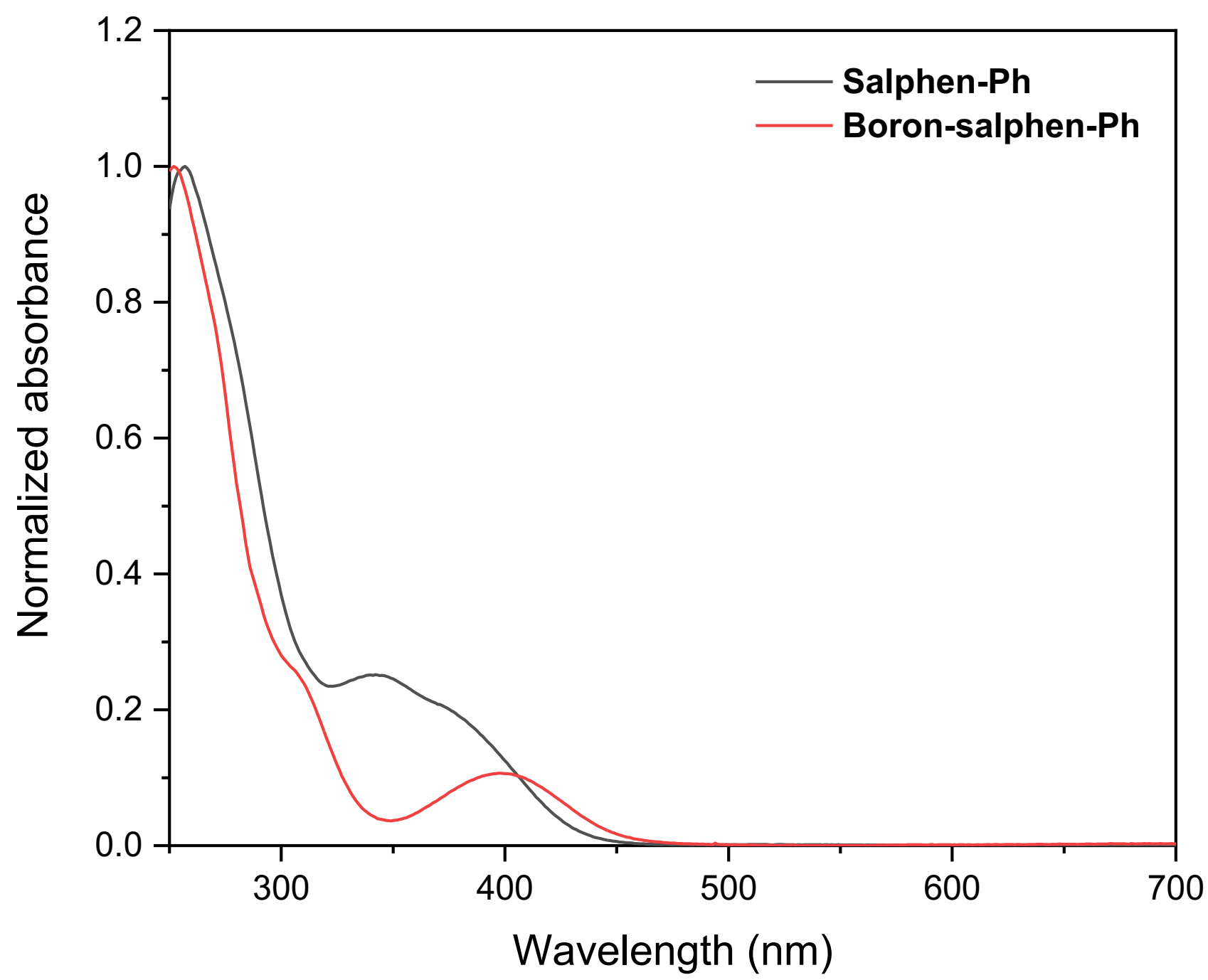

Figure S30. Normalized UV/Vis spectra of **salphen-Ph** (black line) and **boron-salphen-Ph** (red line) in THF at room temperature.

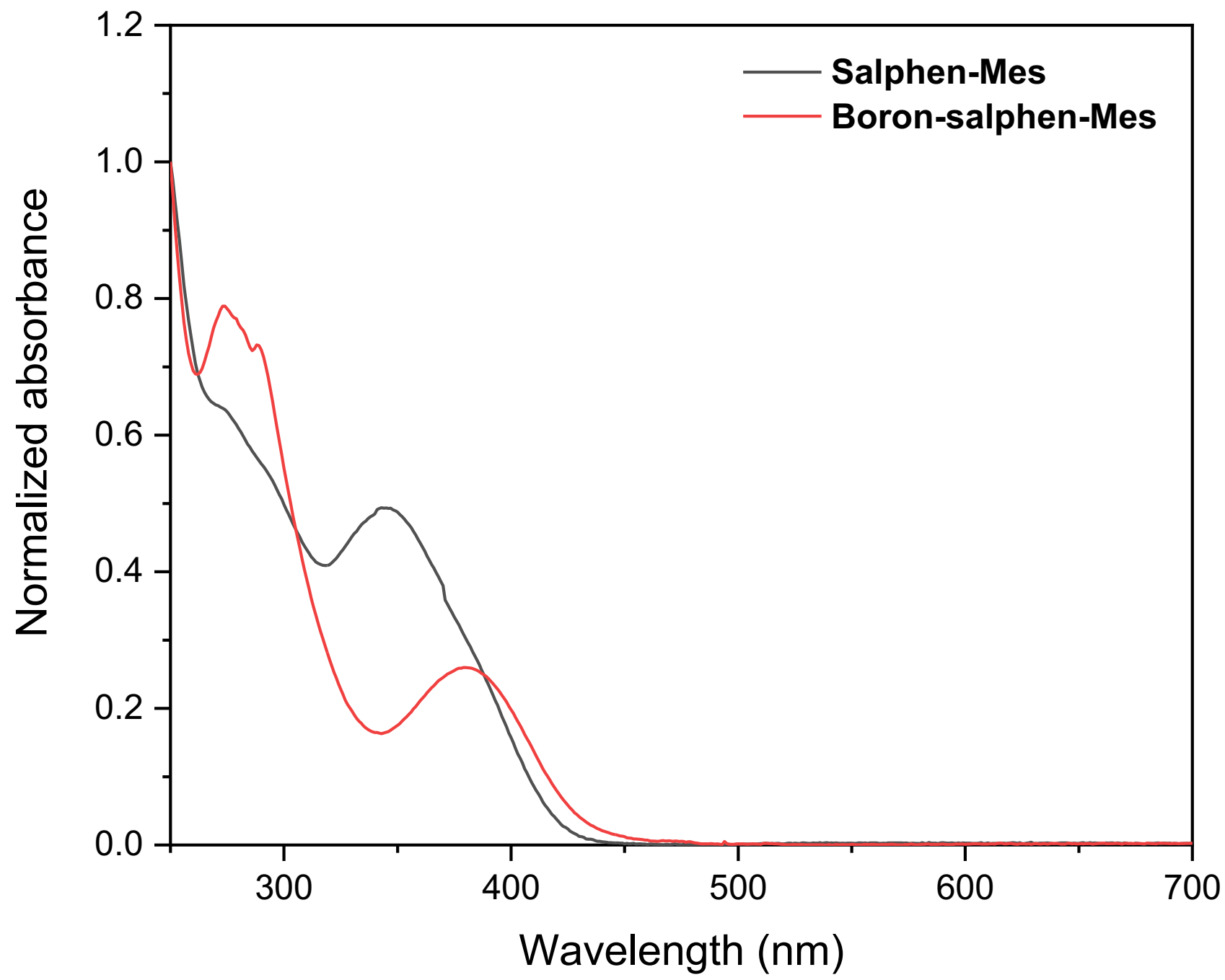

Figure S31. Normalized UV/Vis spectra of **salphen-Mes** (black line) and **boron-salphen-Mes** (red line) in THF at room temperature.

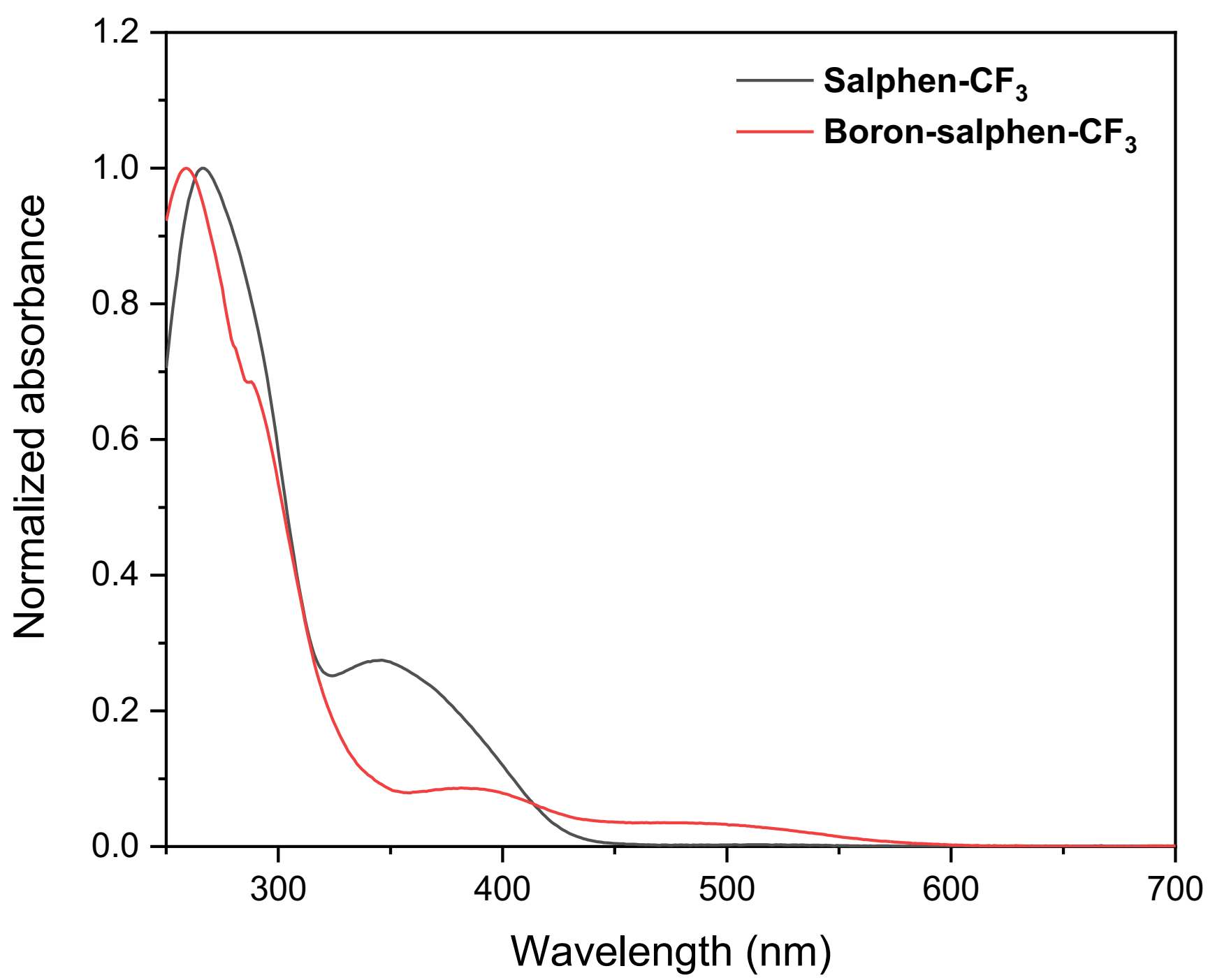

Figure S32. Normalized UV/Vis spectra of **salphen-CF_3** (black line) and **boron-salphen-CF_3** (red line) in THF at room temperature.

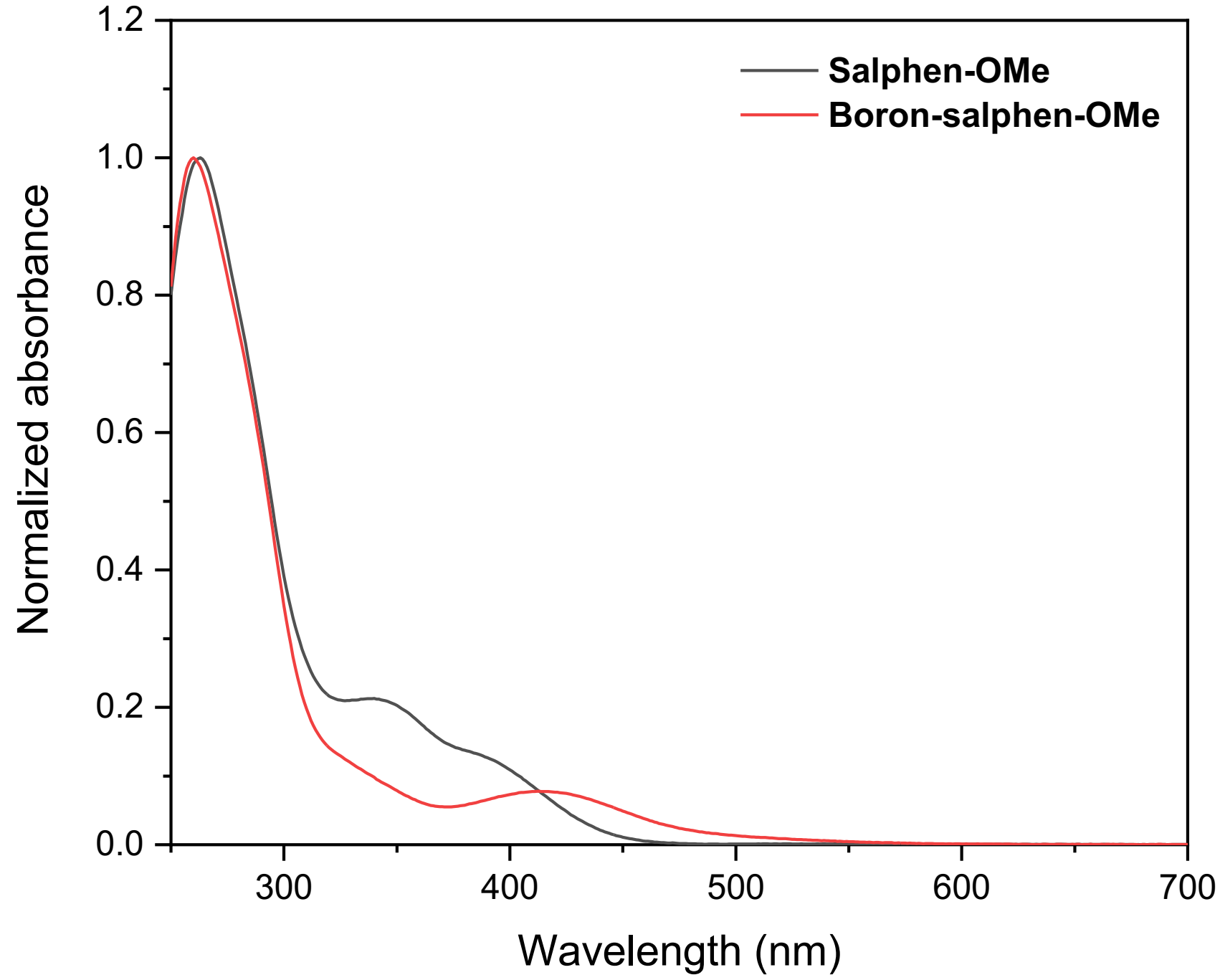

Figure S33. Normalized UV/Vis spectra of **salphen-OMe** (black line) and **boron-salphen-OMe** (red line) in THF at room temperature.

Molar absorption coefficients ε were obtained by plotting the absorbance A against sample concentration C as the slope *k* of the linear regression analysis as in:

$$\mathrm{A} = k \cdot \mathrm{C} + \mathrm{b}$$

where b is the *y*-axis intercept.

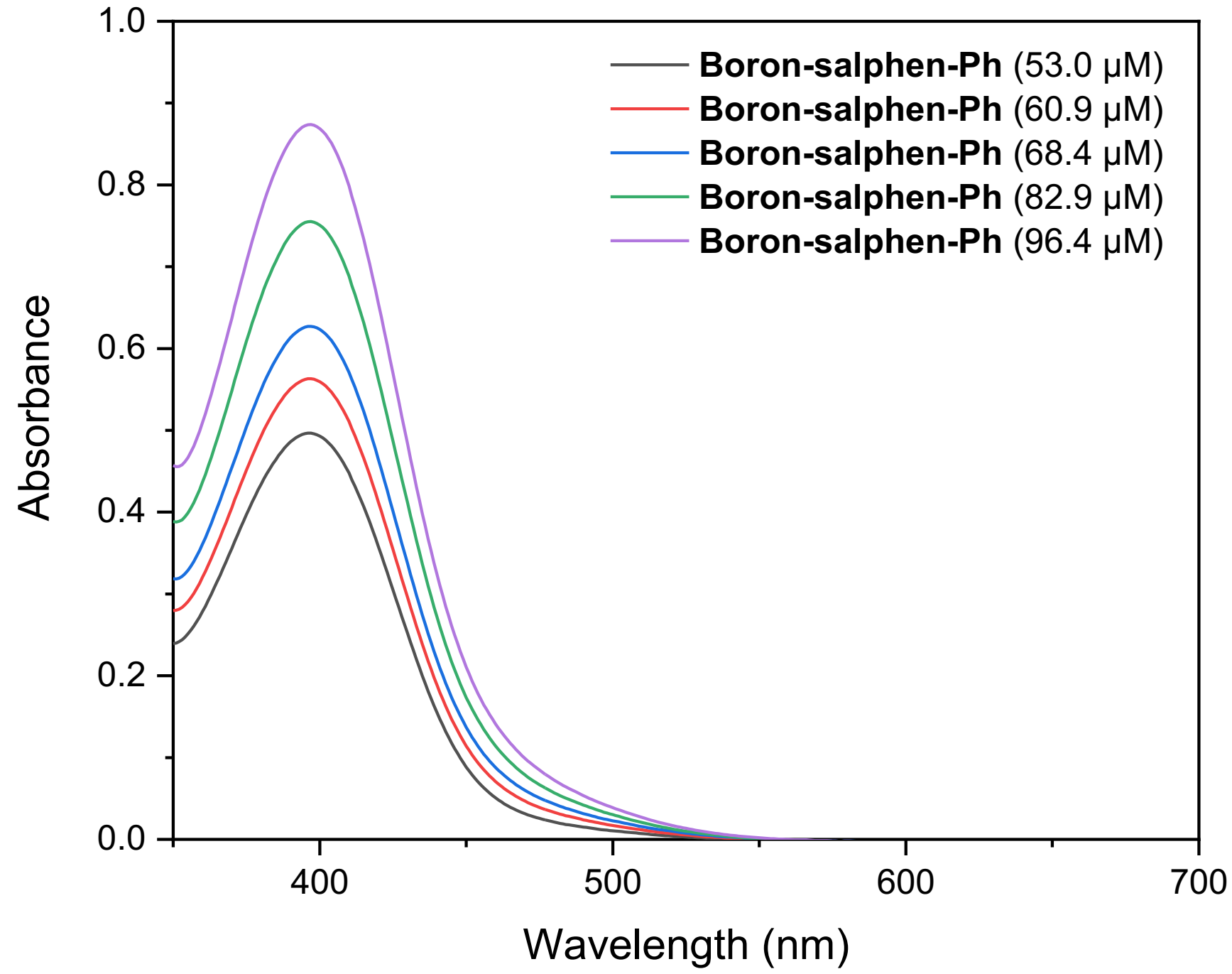

Figure S34. UV/Vis spectra of dilution series of **boron-salphen-Ph** in THF at room temperature (zoom on 350-700 nm window).

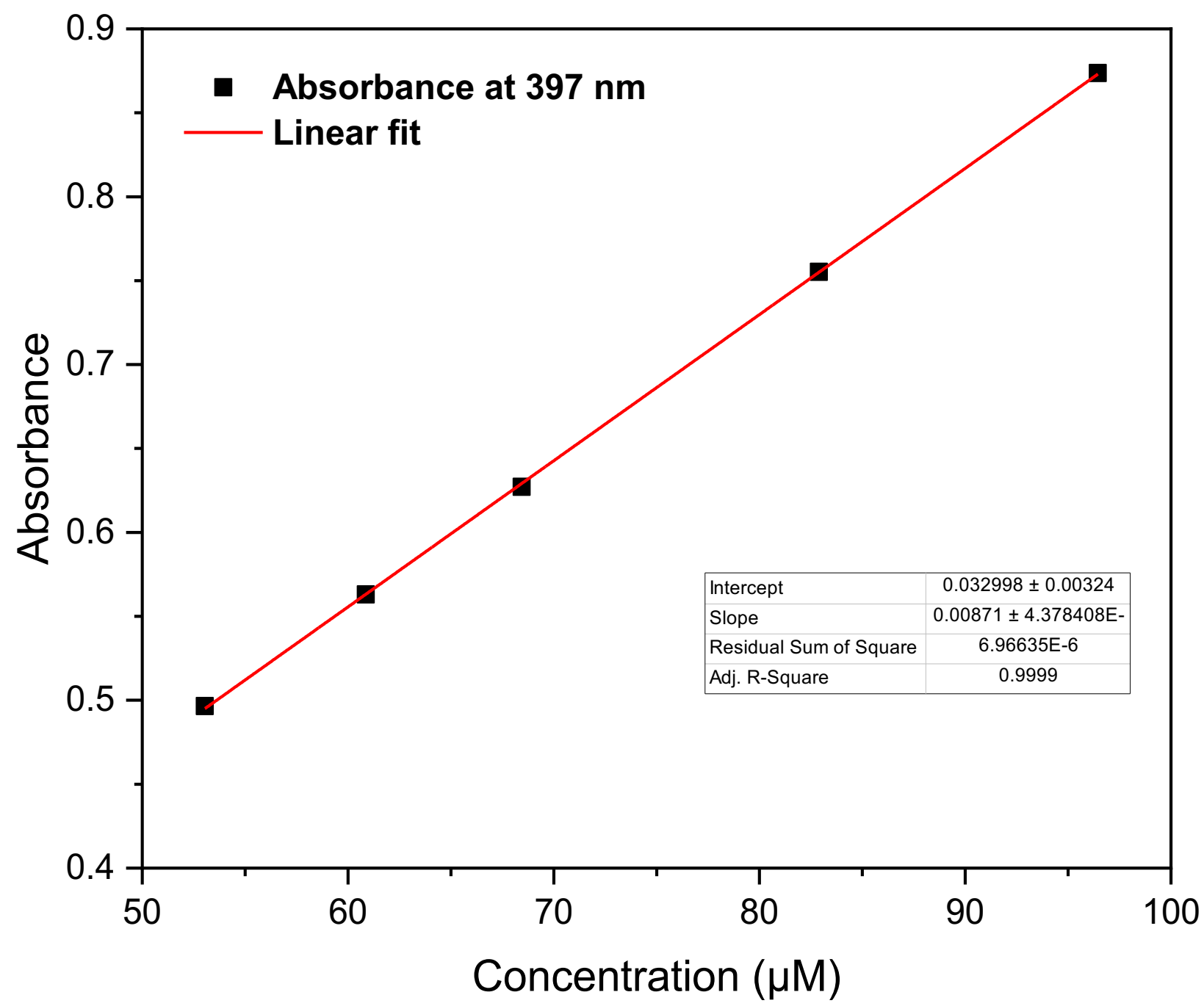

Figure S35. Linear regression analysis of absorbance at 397 nm of **boron-salphen-Ph** in THF at room temperature, plotted against the respective sample concentration.

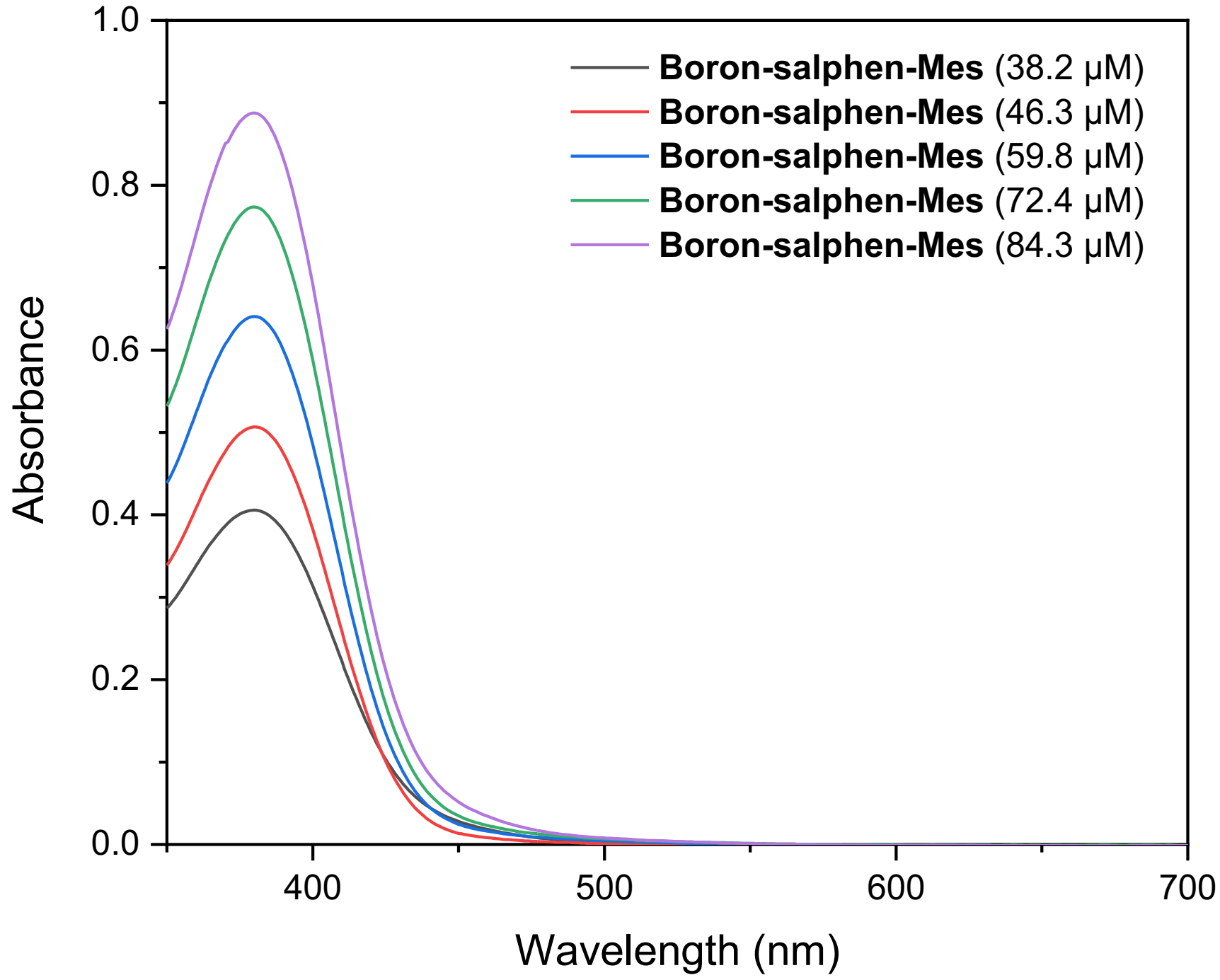

Figure S36. UV/Vis spectra of dilution series of **boron-salphen-Mes** in THF at room temperature (zoom on 350-700 nm window).

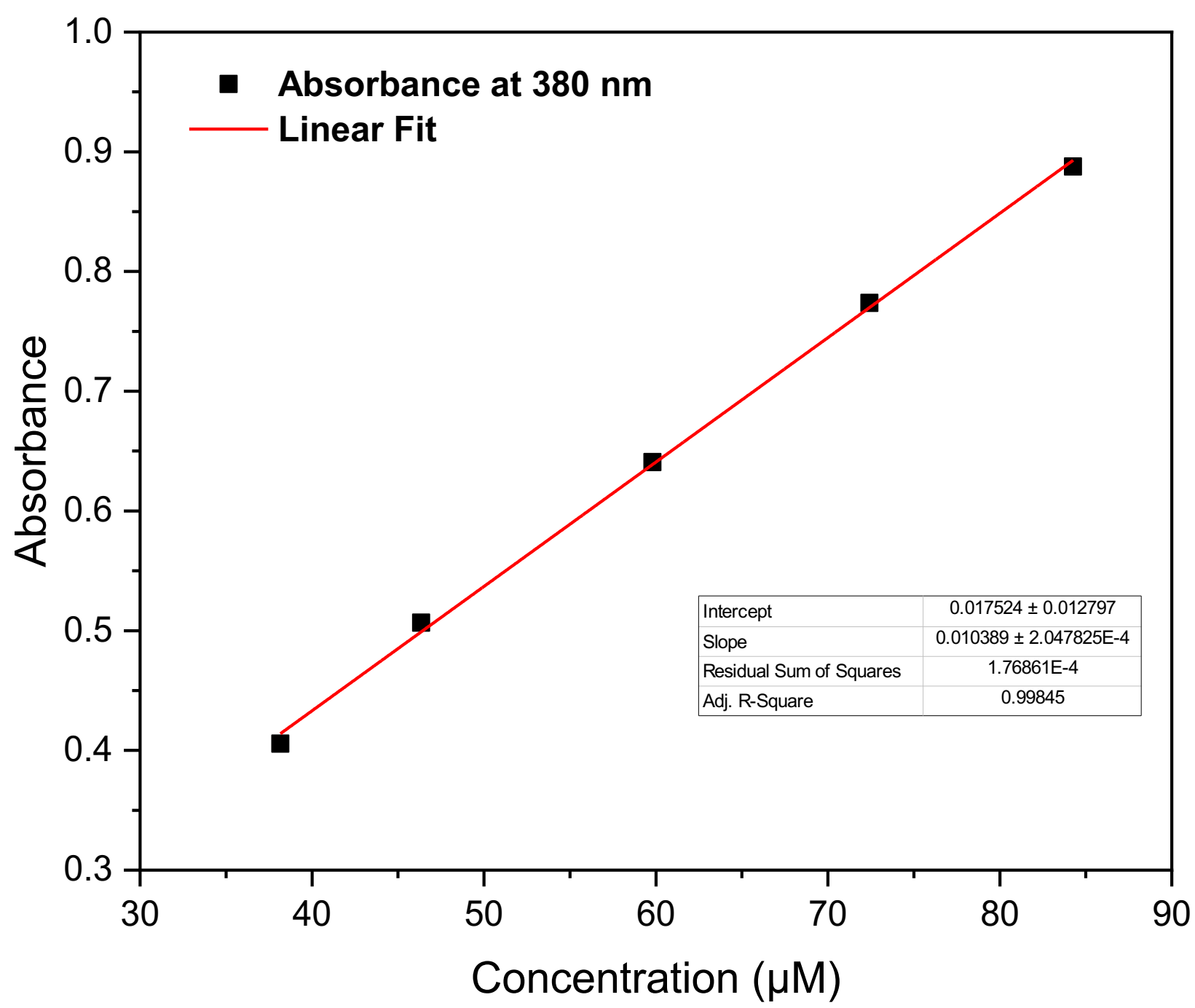

Figure S37. Linear regression analysis of absorbance at 380 nm of **boron-salphen-Mes** in THF at room temperature, plotted against the respective sample concentration.

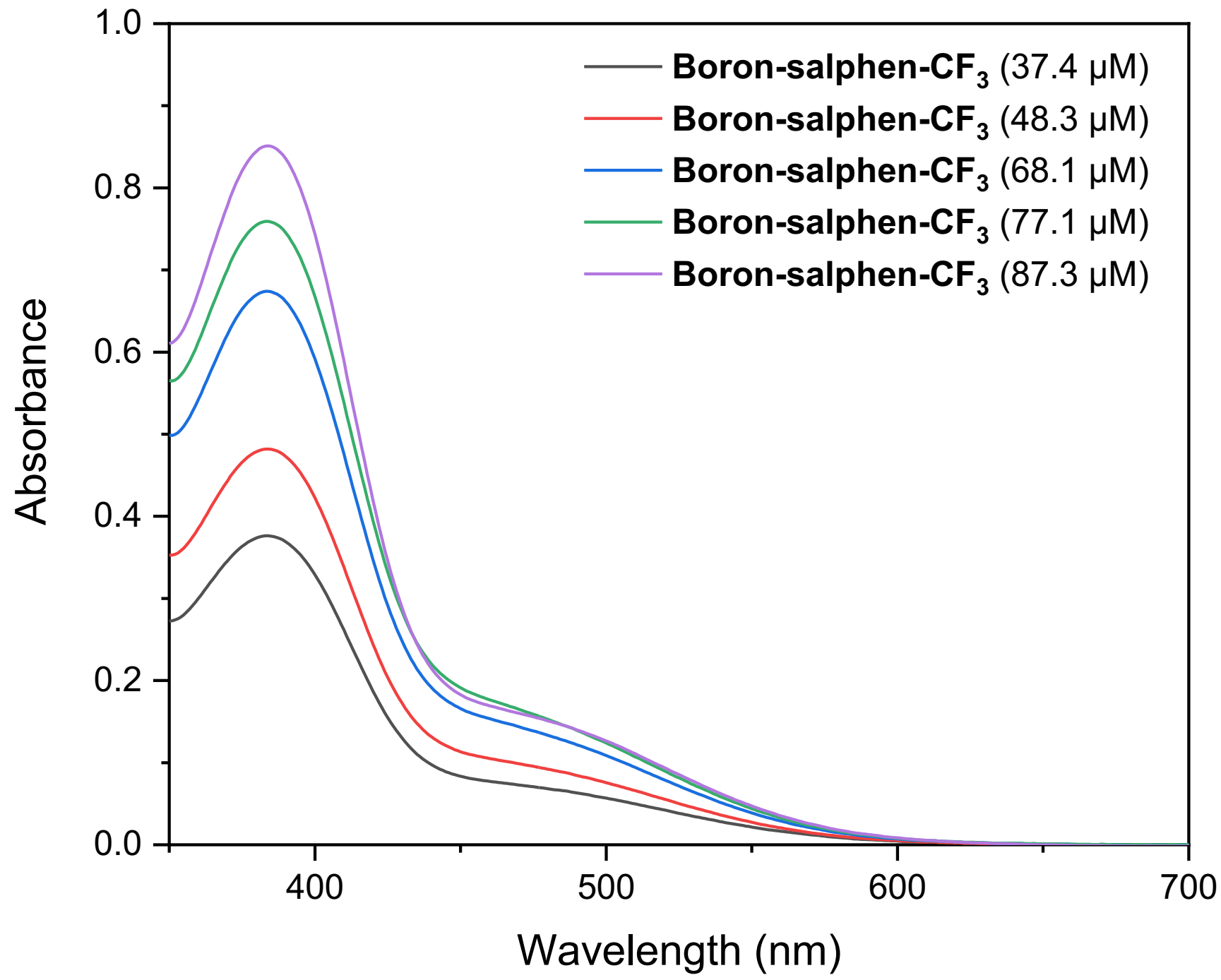

Figure S38. UV/Vis spectra of dilution series of **boron-salphen-CF_3** in THF at room temperature (zoom on 350-700 nm window).

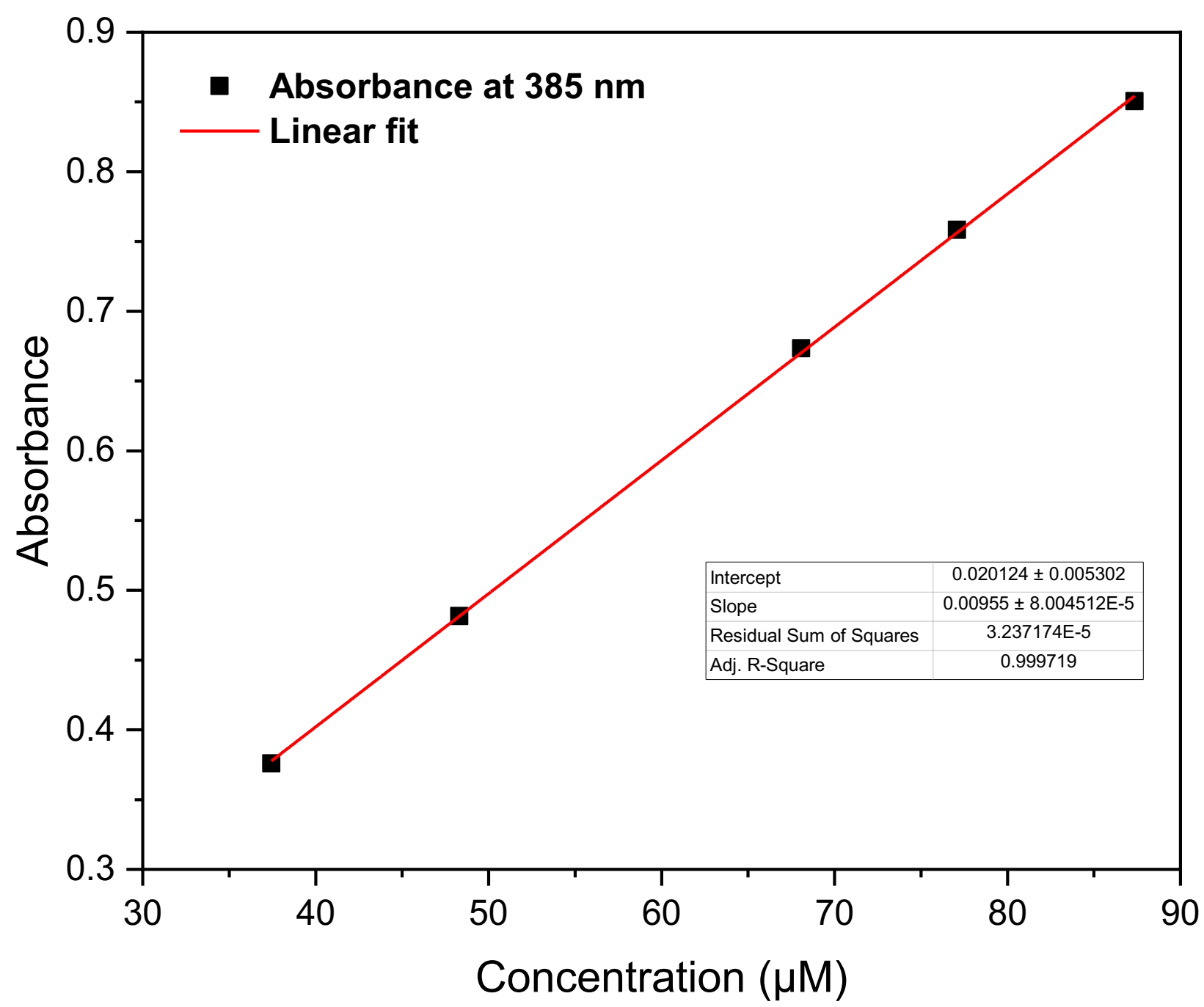

Figure S39. Linear regression analysis of absorbance at 385 nm of **boron-salphen-CF_3** in THF at room temperature, plotted against the respective sample concentration.

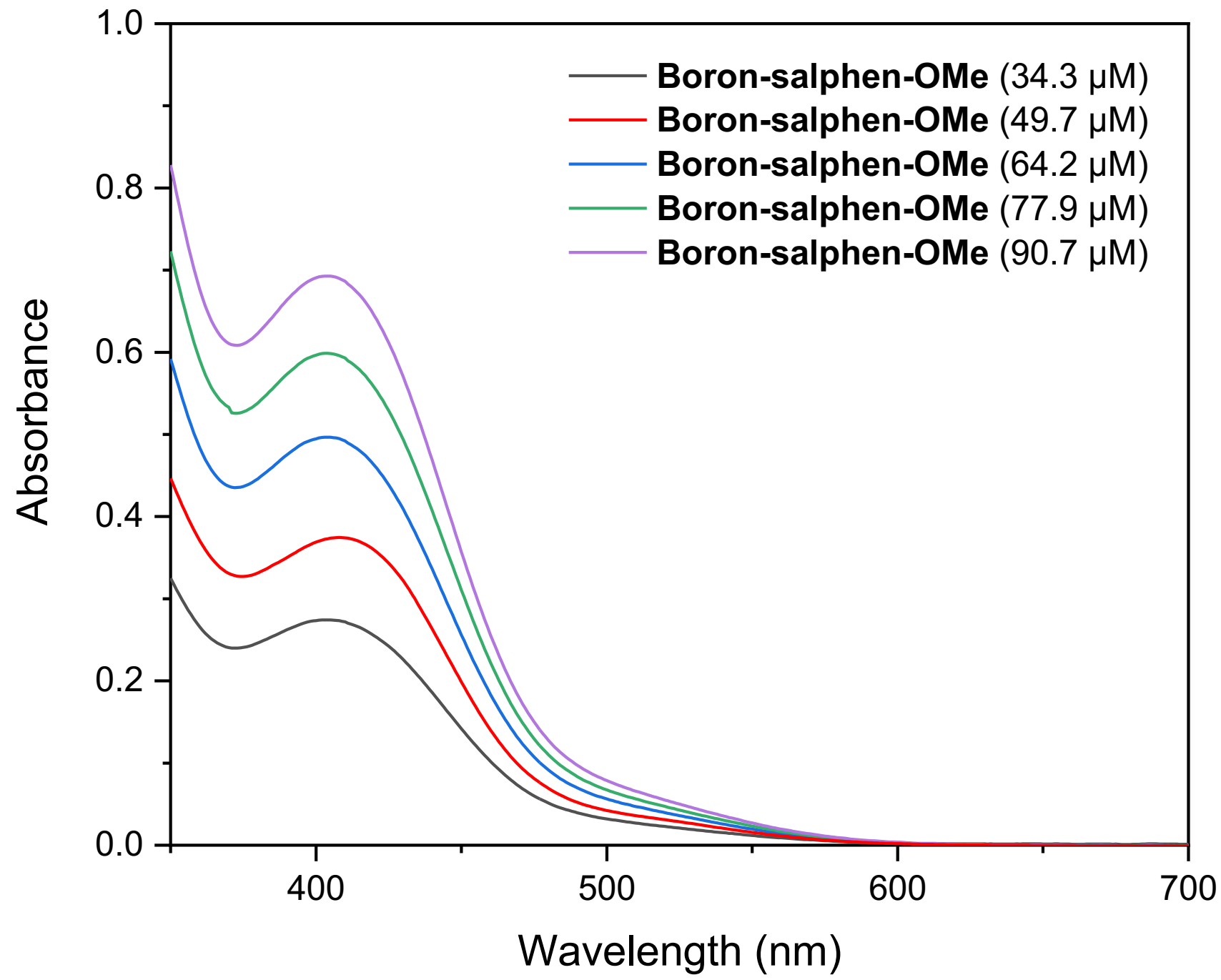

Figure S40. UV/Vis spectra of dilution series of **boron-salphen-OMe** in THF at room temperature (zoom on 350-700 nm window).

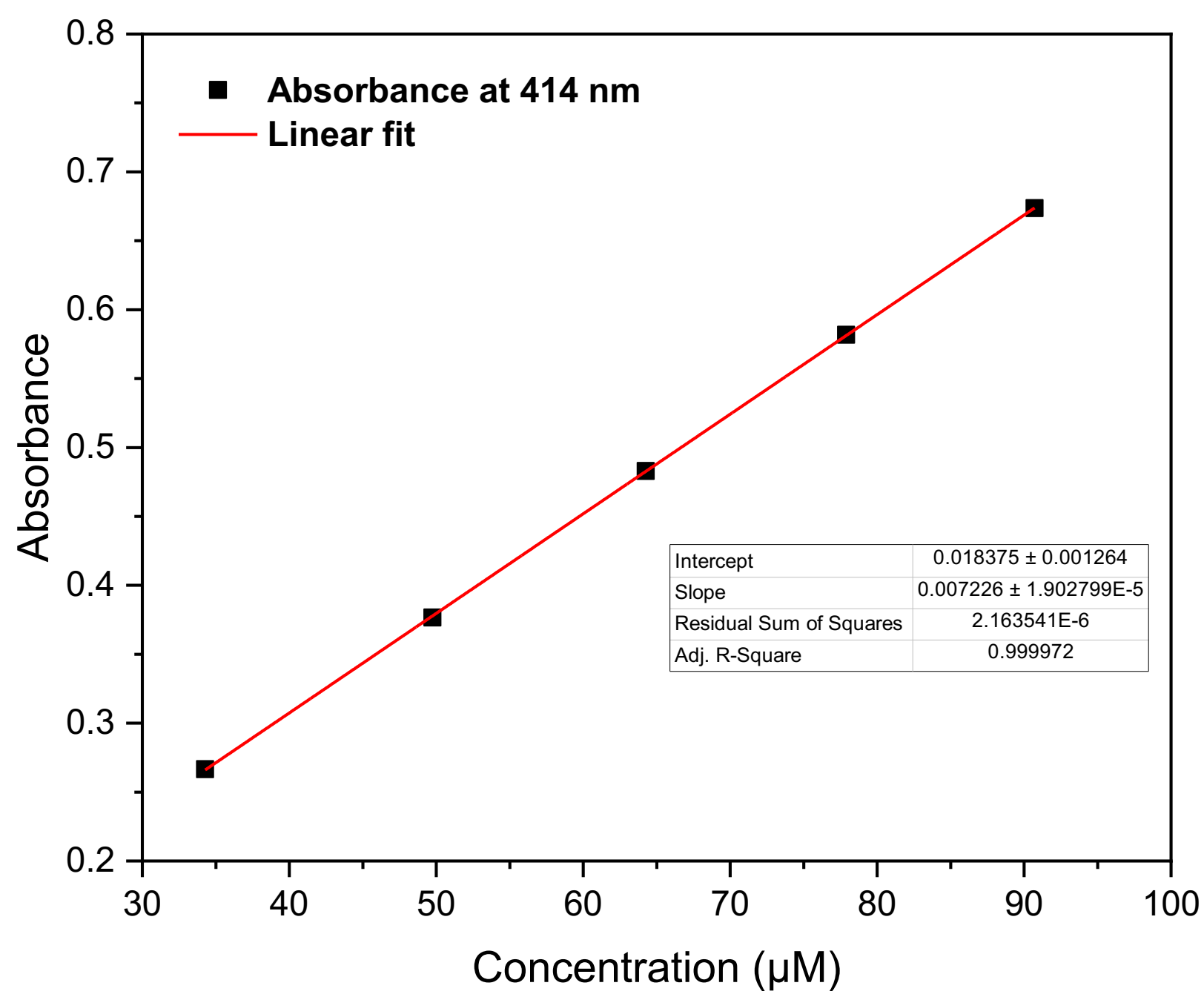

Figure S41. Linear regression analysis of absorbance at 414 nm of **boron-salphen-OMe** in THF at room temperature, plotted against the respective sample concentration.

5.3. Fluorescence spectroscopy

Emission and excitation spectra were measured at the most interesting transitions shown in the absorption spectrum. **Table S2** shows photophysical parameters for π-extended salphen ligands.

Table S2. Overview of photophysical parameters for π-extended salphen ligands.

Compound	λ_{abs} (nm)[a]	λ_{em} (nm) [λ_{ex} (nm)][a]
**Salphen-Ph**	375	627 [360]
**Salphen-Mes**	345	598 [345]
**Salphen-CF$_3$**	347	618 [350]
**Salphen-OMe**	393	614 [390]

[a] Reported for the most red-shifted abs. band.

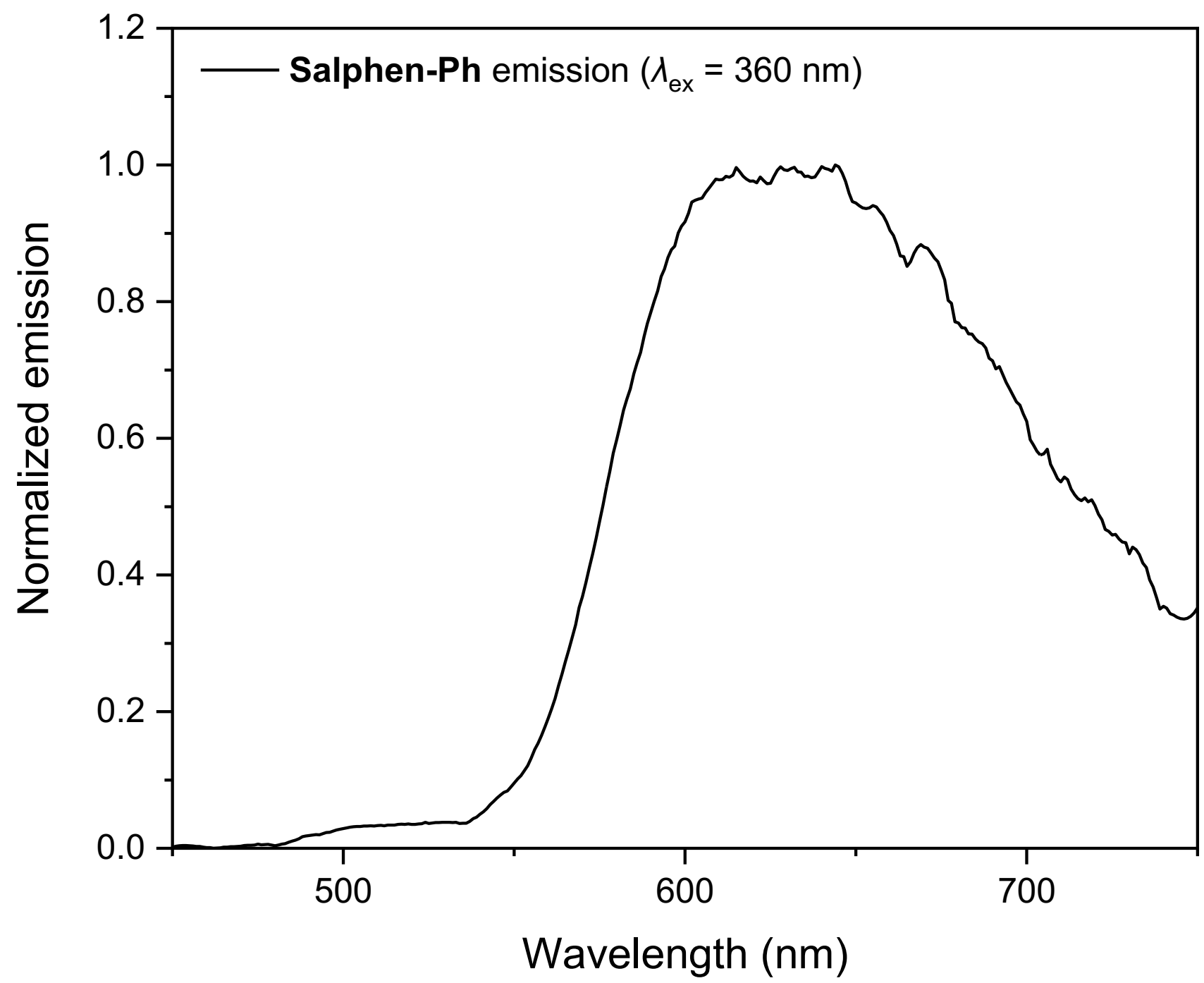

Figure S42. Normalized fluorescence emission of **salphen-Ph** in THF at room temperature, recorded at an excitation wavelength of 360 nm.

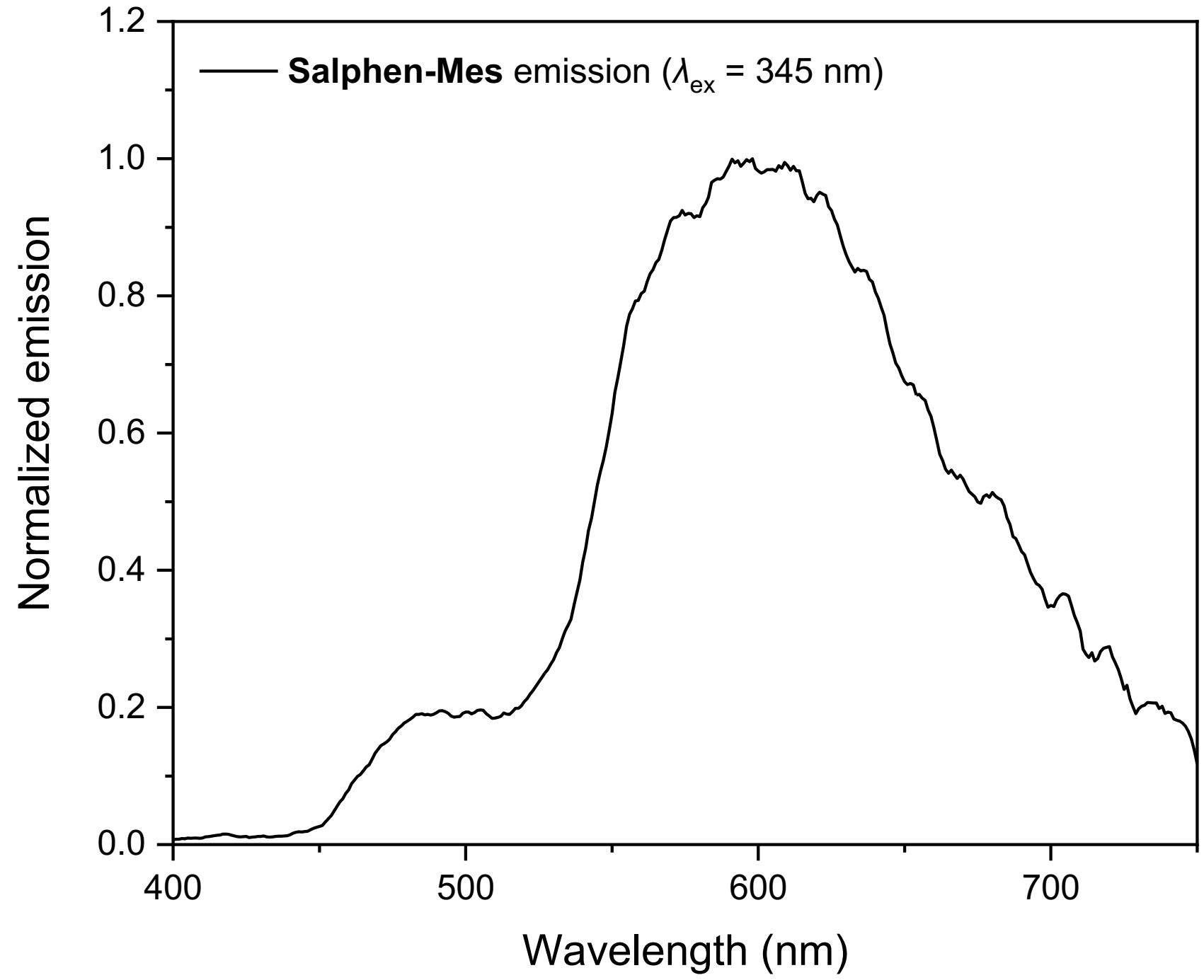

Figure S43. Normalized fluorescence emission of **salphen-Mes** in THF at room temperature, recorded at an excitation wavelength of 345 nm.

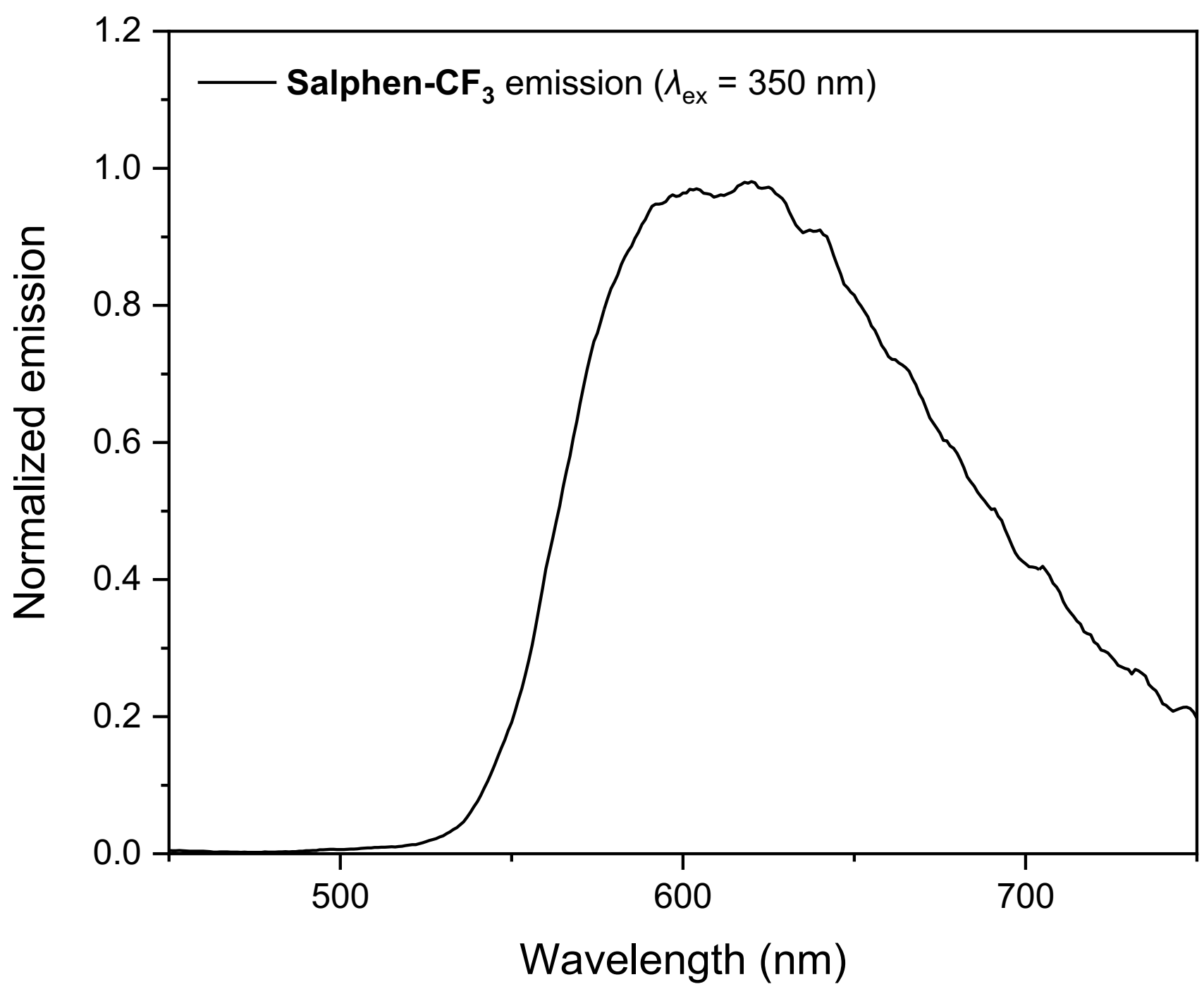

Figure S44. Normalized fluorescence emission of **salphen-CF_3** in THF at room temperature, recorded at an excitation wavelength of 350 nm.

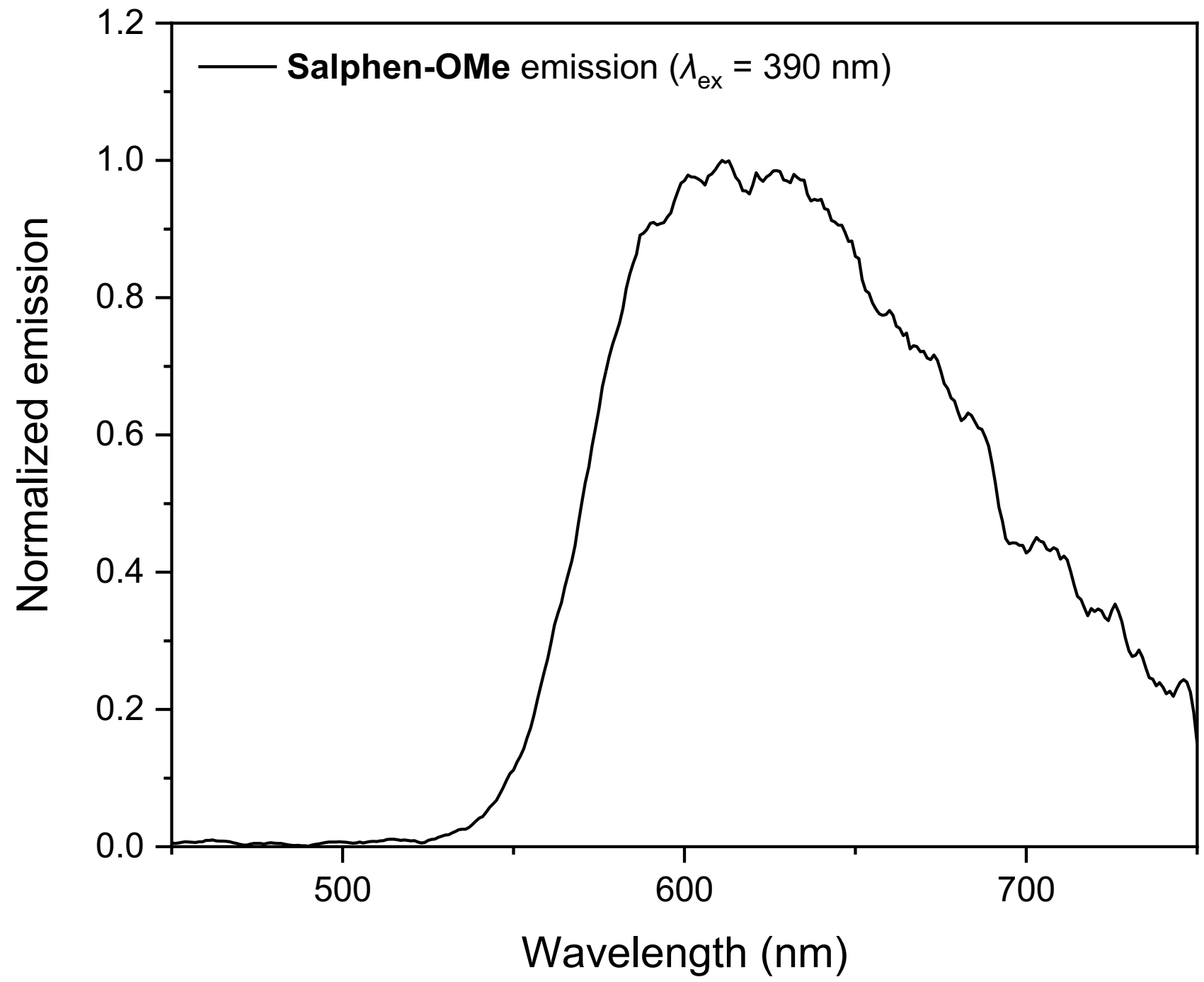

Figure S45. Normalized fluorescence emission of **salphen-OMe** in THF at room temperature, recorded at an excitation wavelength of 390 nm.

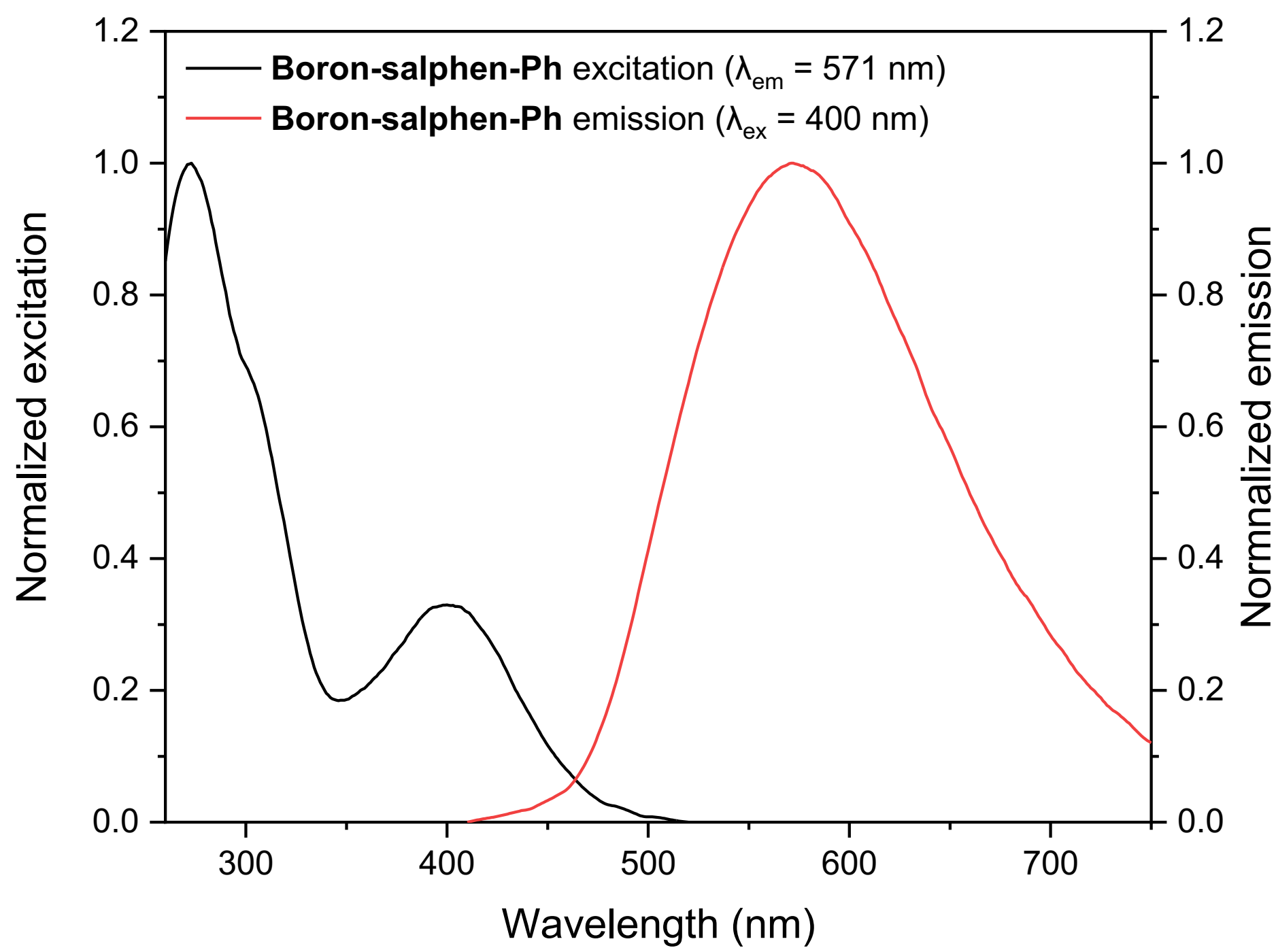

Figure S46. Normalized fluorescence emission and excitation spectra of **boron-salphen-Ph** in THF at room temperature. Excitation spectrum was recorded at an emission wavelength of 571 nm. Emission spectrum was recorded at an excitation wavelength of 400 nm.

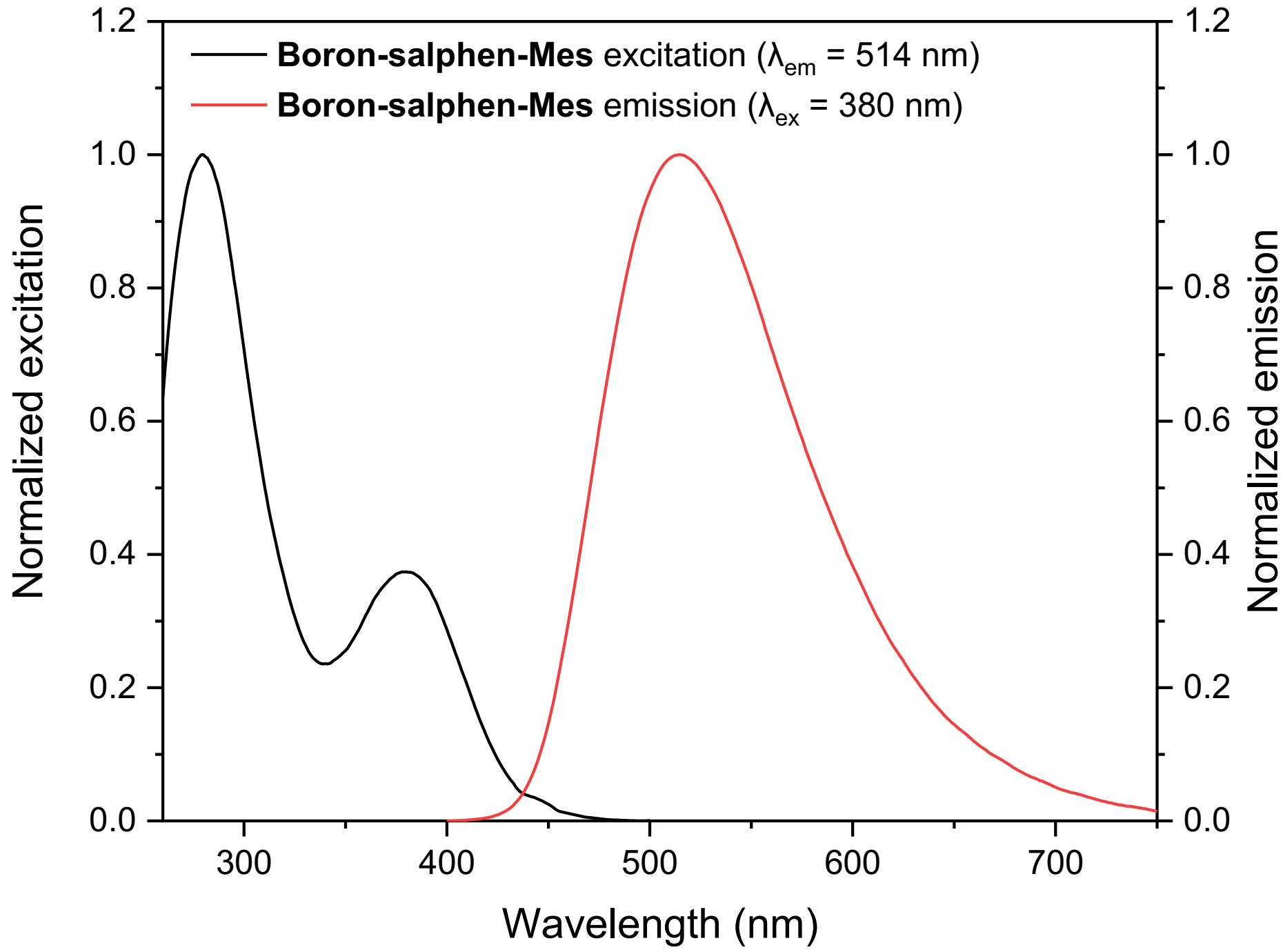

Figure S47. Normalized fluorescence emission and excitation spectra of **boron-salphen-Mes** in THF at room temperature. Excitation spectrum was recorded at an emission wavelength of 514 nm. Emission spectrum was recorded at an excitation wavelength of 380 nm.

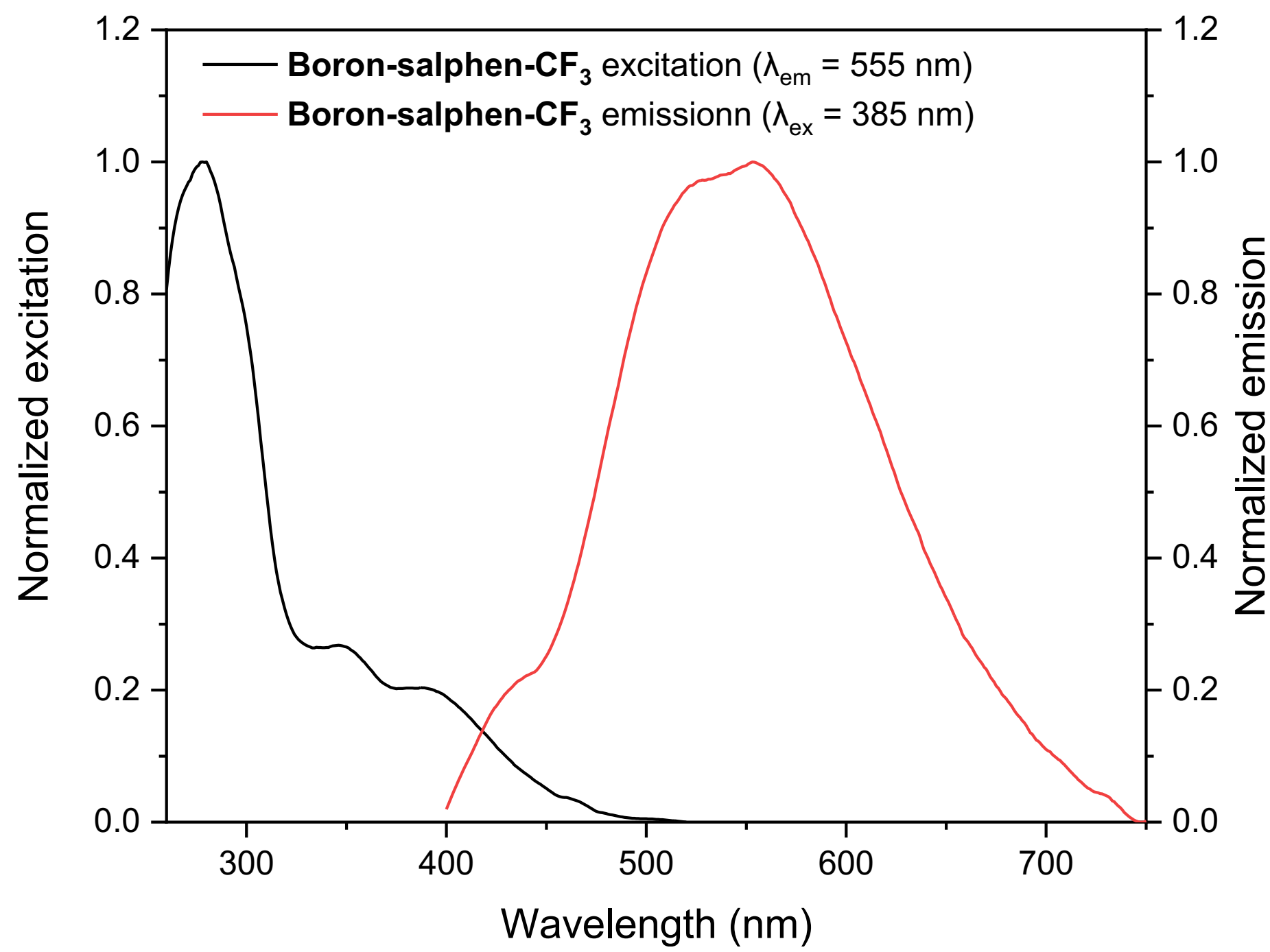

Figure S48. Normalized fluorescence emission and excitation spectra of **boron-salphen-CF_3** in THF at room temperature. Excitation spectrum was recorded at an emission wavelength of 555 nm. Emission spectrum was recorded at an excitation wavelength of 385 nm.

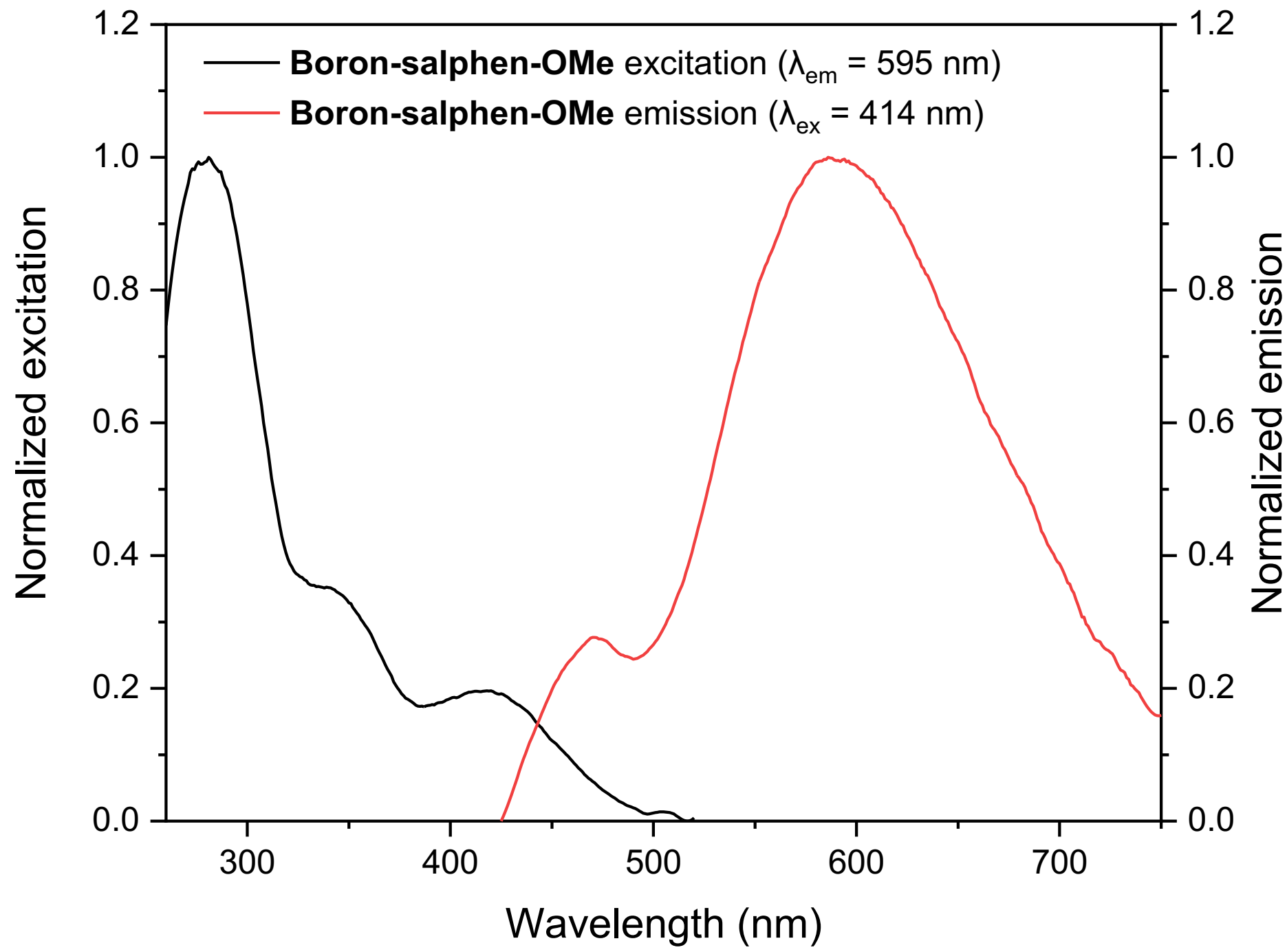

Figure S49. Normalized fluorescence emission and excitation spectra of **boron-salphen-OMe** in THF at room temperature. Excitation spectrum was recorded at an emission wavelength of 595 nm. Emission spectrum was recorded at an excitation wavelength of 414 nm.

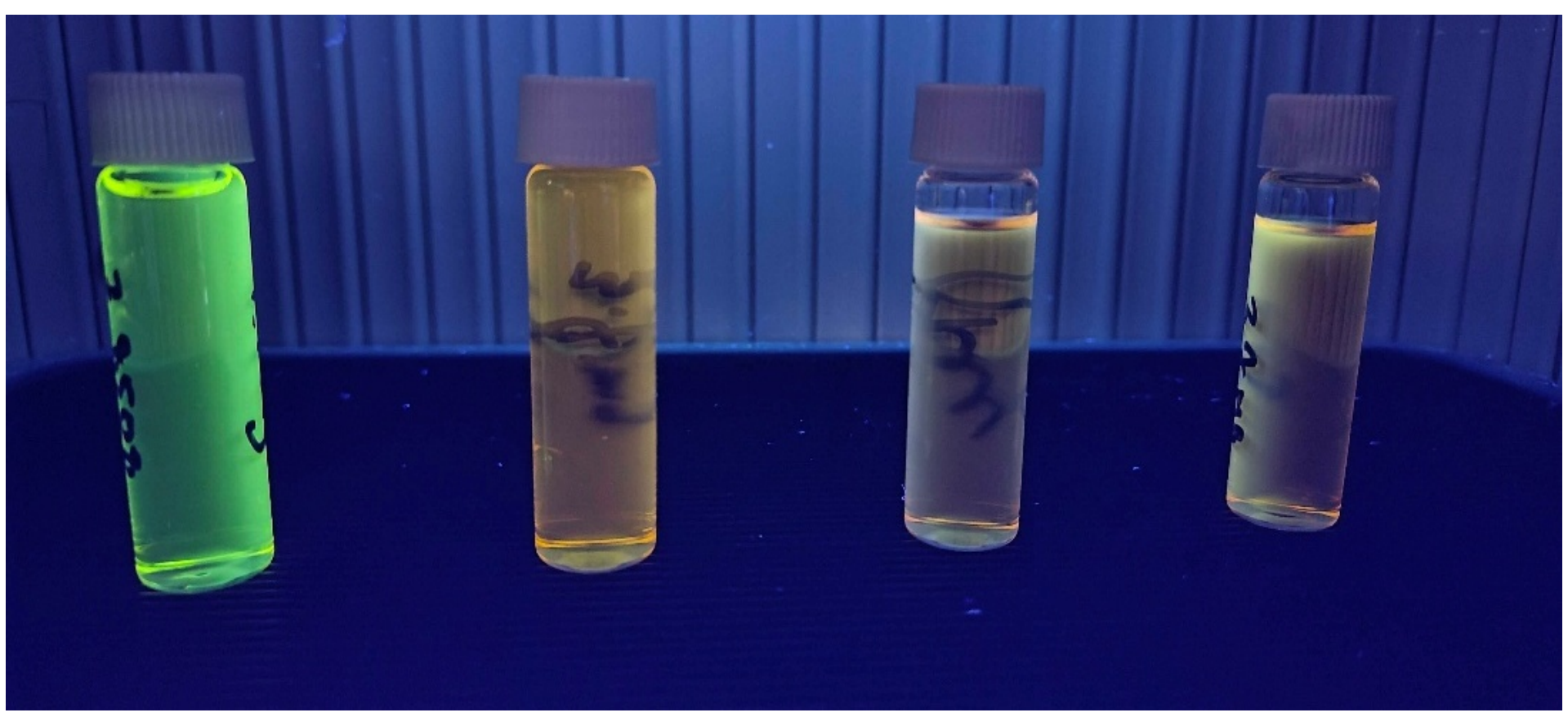

Figure S50. THF solutions of boron complexes under 366 nm light (from left to right: **boron-salphen-Mes**, **boron-salphen-Ph**, **boron-salphen-OMe** and **boron-salphen-CF$_3$**).

Stokes shifts ($\Delta\tilde{\nu}$, in cm^{-1}), were calculated considering the maxima of excitation (λ_{ex}, in nm) related to the second transition, and emission (λ_{em}, in nm) according to the formula:

$$\Delta\tilde{\nu} = \left(\frac{10^7}{\lambda_{ex}} - \frac{10^7}{\lambda_{em}}\right)$$

Table S3 shows Stokes shifts for π-extended boron-salphen complexes.

Table S3. Stokes shifts for π-extended boron-salphen complexes.

Complex	Stokes shift (cm^{-1})
**Boron-salphen-Ph**	7487
**Boron-salphen-Mes**	6861
**Boron-salphen-CF_3**	7956
**Boron-salphen-OMe**	7349

5.4. Relative quantum yield determination

Relative quantum yields Φ_{rel} of boron complexes were obtained at room temperature in THF using an ethanol solution of Coumarin 153 (Φ_{ref} = 0.53) as a reference.[65] The quantum yields were determined by plotting the integrated fluorescence emission between 420 and 750 nm against the absorbance of five differently-concentrated solutions of test compounds in THF with absorbance values ranging from 0.02 to 0.10. Respective quantum yields Φ_{rel} were calculated using the equation:

$$\Phi_{\mathrm{rel}} = \Phi_{\mathrm{ref}} \cdot \frac{\mathrm{m}}{\mathrm{m}_{\mathrm{ref}}} \cdot \left(\frac{\mathrm{n}}{\mathrm{n}_{\mathrm{ref}}}\right)^2$$

where m is the slope of the respective boron complex, m_{ref} is the slope of the reference sample, n is the refractive index of the solvent (THF),[66] whereas n_{ref} is the refractive index of the reference solvent (ethanol).[67]

5.5. Luminescence lifetime measurements

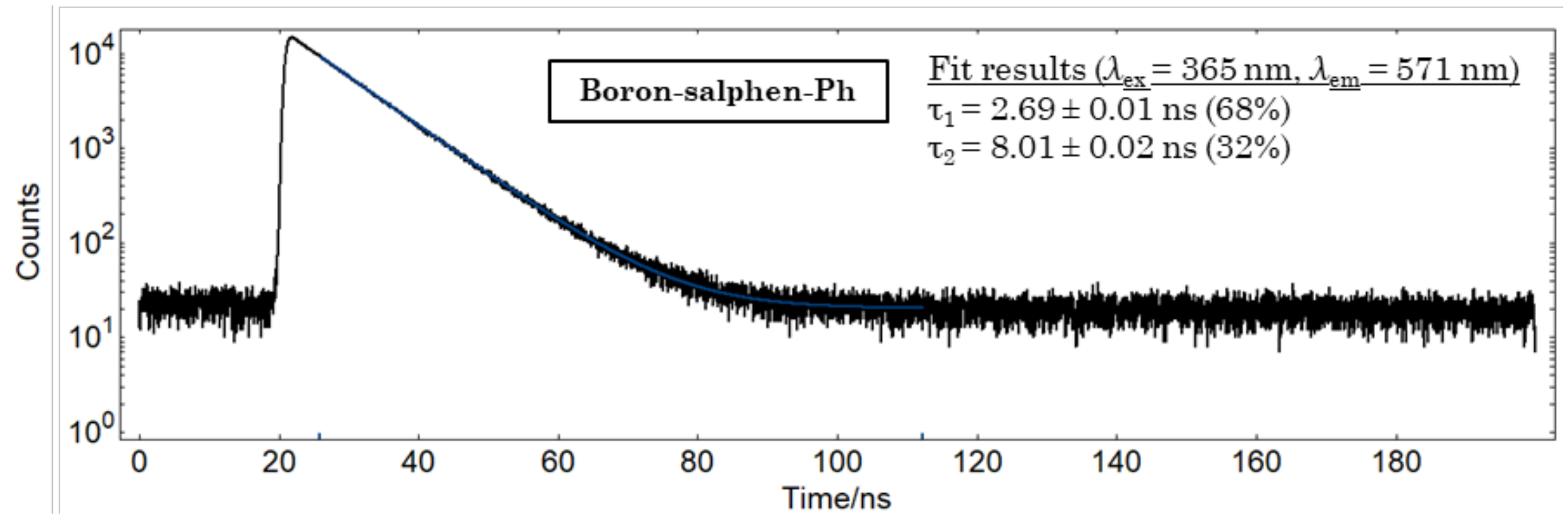

Figure S51. Photoluminescence lifetime decay of **boron-salphen-Ph** at 77 K in toluene.

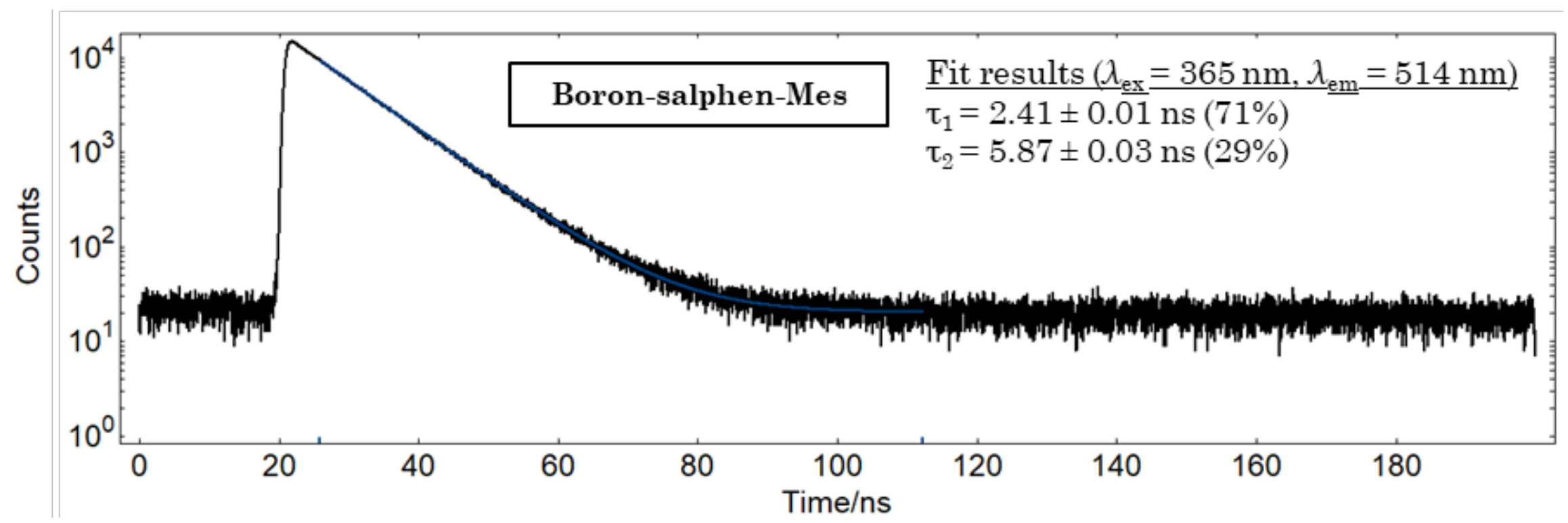

Figure S52. Photoluminescence lifetime decay of **boron-salphen-Mes** at 77 K in toluene.

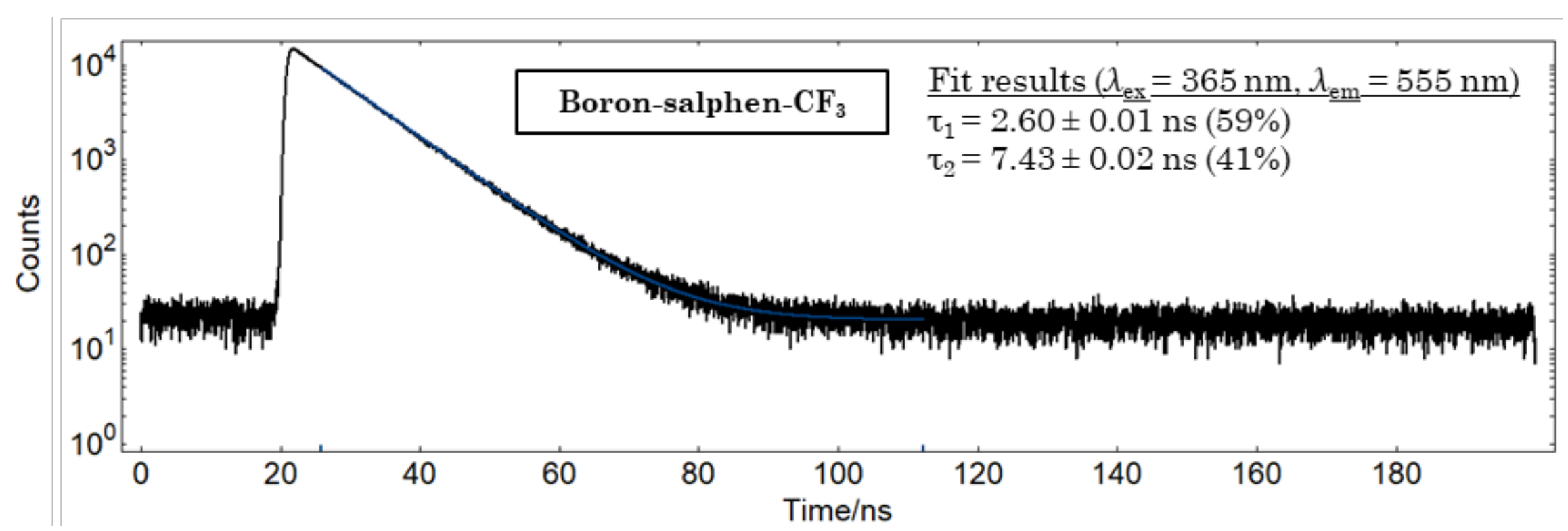

Figure S53. Photoluminescence lifetime decay of **boron-salphen-CF_3** at 77 K in toluene.

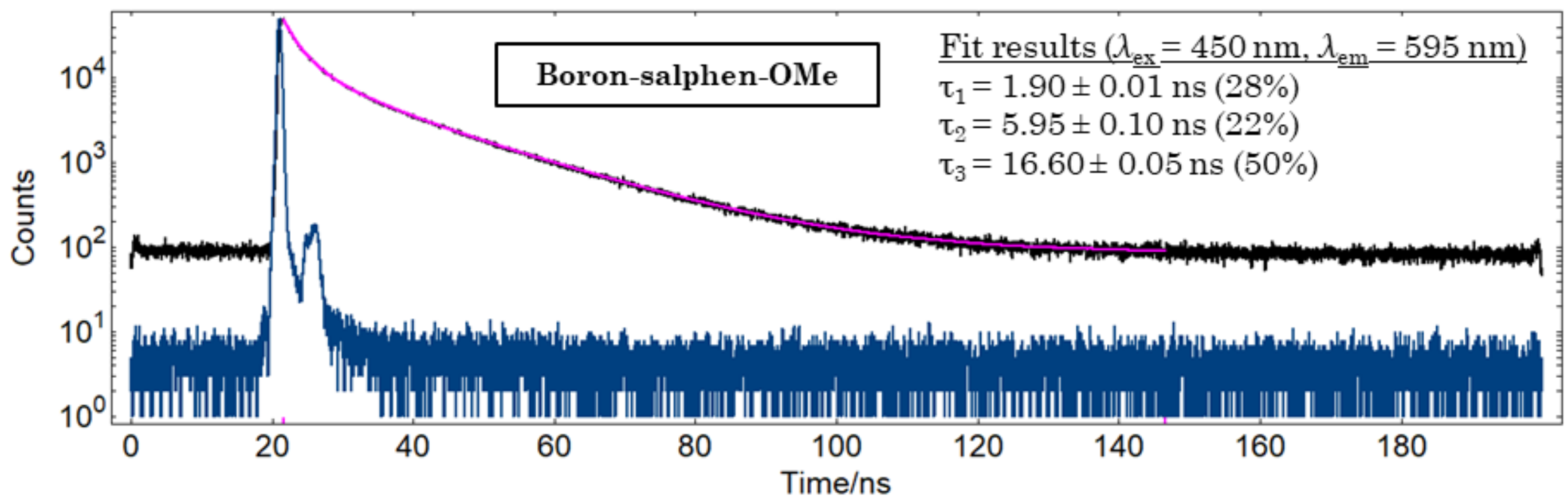

Figure S54. Photoluminescence lifetime decay of **boron-salphen-OMe** at 77 K in toluene (the black line is the decay, the blue line is the instrument response, the pink line is the reconvolution fit).

6. Photosensitizer application

The optimization of the oxidation of adamantilydeneadamantane (**13**) was conducted using the setup described previously.

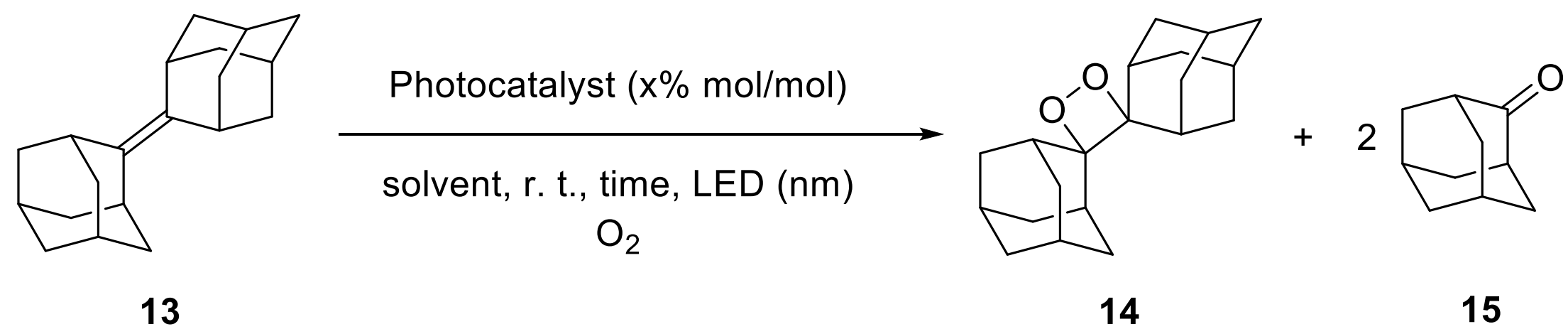

Scheme S4. Oxidation of adamantilydeneadamantane (**13**).

An 8 mL screw-cap vial equipped with a rubber septum and a stirring bar was charged with starting material **13** (1 equiv., 0.050 mmol, 0.0133 g), an appropriate amount of catalyst (from stock solution), and solvent (0.5 mL). Oxygen (circa 0.2 bar) was put inside [via FPT: the reaction mixture was prepared in a Schlenk tube, frozen to -196°C, degassed one time and backfilled with oxygen; via balloon: a balloon full of oxygen was connected via needle; via bubbling: oxygen was bubbled inside the system for few seconds]. The reaction vessel was irradiated with different lights for a certain time at room temperature. After that, the solvent was removed under reduced pressure and a ^{1}H NMR of the crude was recorded with 1,3,5-trimethoxybenzene (0.6 equiv., 0.0300 mmol, 0.0050 g), as internal standard, in $CDCl_3$.

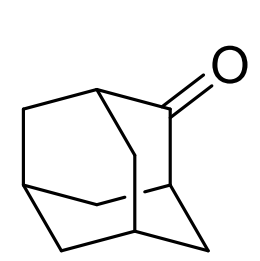

15: **^{1}H NMR** (500 MHz, $CDCl_3$): *δ* 2.53 (s, 2H), 2.08-1.92 (m, 12H). NMR data were in accordance with those previously reported.[68]

The NMR conversions and molar fractions have been determined by integration of diagnostic signals, which are related to the protons on the carbon vicinal to the one

involved in the double bond or bonded with the oxygen. For the reagent **13** $(-(C\underline{H})_2C=)_2$, it is at 2.90 ppm (4H), while for dioxetane **14** $(-(C\underline{H})_2CO)_2$ and ketone **15** $2(-(C\underline{H})_2CO)$, it resonates respectively at 2.64 (4H) and 2.54 ppm (2 x 2H). Each signal has been highlighted in **Figure S55**, using red, green and blue marks, respectively, for the reagent ($\mathbf{I}_R$), dioxetane ($\mathbf{I}_D$) and ketone ($\mathbf{I}_K$). The conversion and the products' molar fractions have been evaluated by the following equations:

$$\text{Conversion (\%)} = 100 \cdot \left(1 - \frac{\boldsymbol{I_R}}{\boldsymbol{I_R} + \boldsymbol{I_D} + \boldsymbol{I_K}}\right)$$

$$\text{Dioxetane molar fraction (\%)} = 100 \cdot \left(\frac{\boldsymbol{I_D}}{\boldsymbol{I_D} + \boldsymbol{I_K}}\right)$$

$$\text{Ketone molar fraction (\%)} = 100 \cdot \left(\frac{\boldsymbol{I_K}}{\boldsymbol{I_D} + \boldsymbol{I_K}}\right)$$

The internal standard has been used to exclude side-reactivity.

For example, in **Figure S55**, $\boldsymbol{I}_R$ = 10.67, $\boldsymbol{I}_K$ = 2.69, and $\boldsymbol{I}_D$ = 6.06, thus the conversion is 45%, the ketone molar fraction is 69% and the dioxetane molar fraction is 31%.

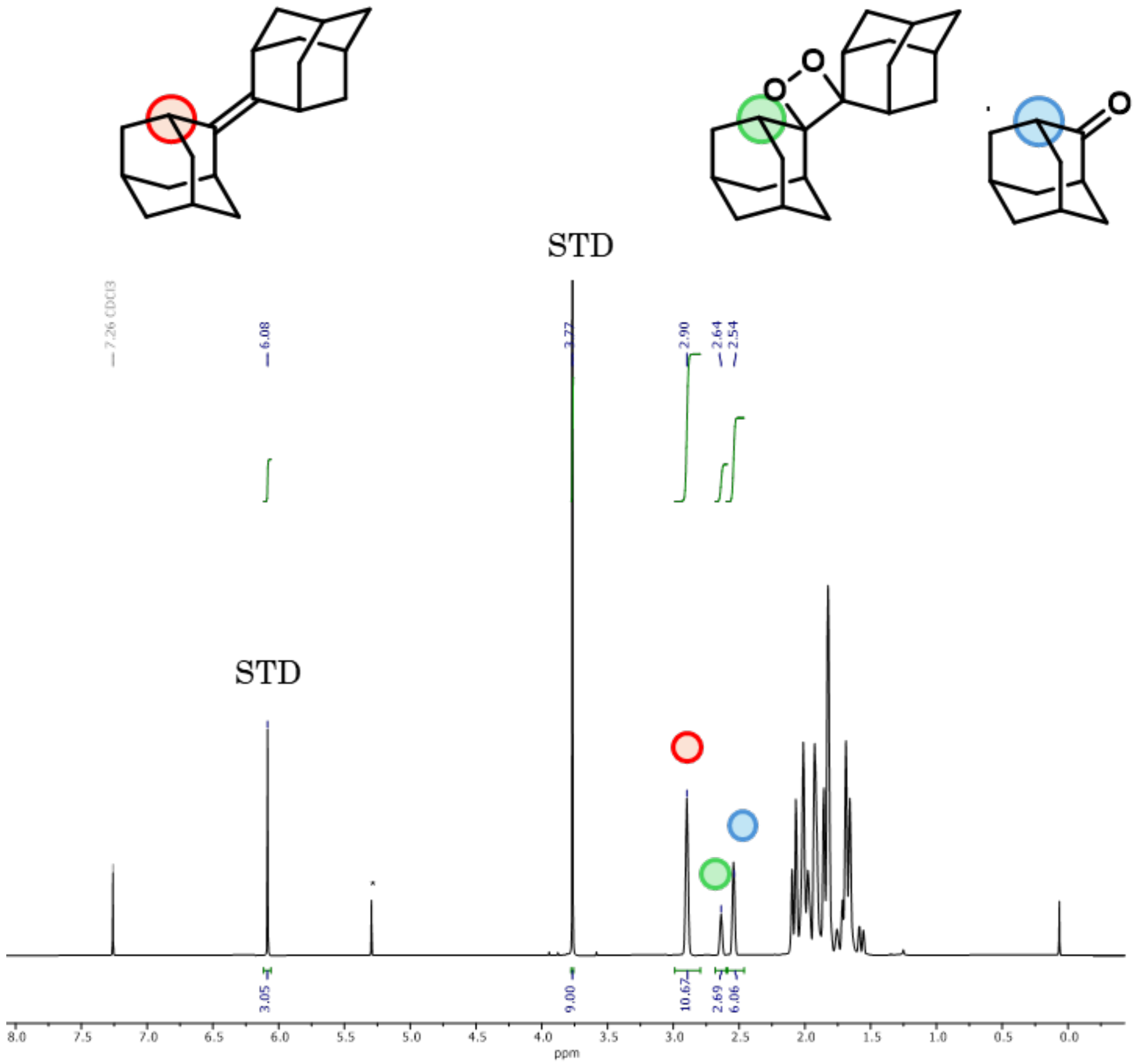

Figure S55. Crude ^{1}H NMR (400 MHz) in $CDCl_3$ and relevant signals for ^{1}H NMR conversion and molar fraction calculations of oxidation of **13**. The symbol (*) marks dichloromethane.

6.1. Light wavelengths

The data concerning the screening of light wavelength are reported in **Table S4**.

Table S4. Screening of light wavelengths. Reaction conditions: solvent = DCM 0.1 M, catalyst = **boron-salphen-OMe**, catalyst loading = 250 ppm with respect to **13**, O_2 (bubbling), temperature = r. t., time = 12 h.

Light	Conversion (%)[a]	Dioxetane molar fraction (%)[a]	Ketone molar fraction (%)[a]
367 nm[b]	100	25	75
390 nm	52	0	100
427 nm	35	5	95
457 nm	25	7	93
525 nm	0	0	0
640 nm	0	0	0

[a] Conversion evaluated by ^1H NMR using an internal standard, error = ± 2%. [b] Other side-products were observed.

6.2. Oxygen systems

The data concerning the screening of oxygen systems are reported in **Table S5**.

Table S5. Screening of oxygen systems. Reaction conditions: solvent = DCM 0.1 M, catalyst = **boron-salphen-OMe**, catalyst loading = 250 ppm with respect to **13**, LED = 390 nm, temperature = r. t., time = 12 h.

Oxygen system	Conversion (%)[a]	Dioxetane molar fraction (%)[a]	Ketone molar fraction (%)[a]
FPT	52	0	100
O_2 balloon	52	0	100
Bubbling	52	0	100

[a]Conversion evaluated by ^1H NMR using an internal standard, error = ± 2%.

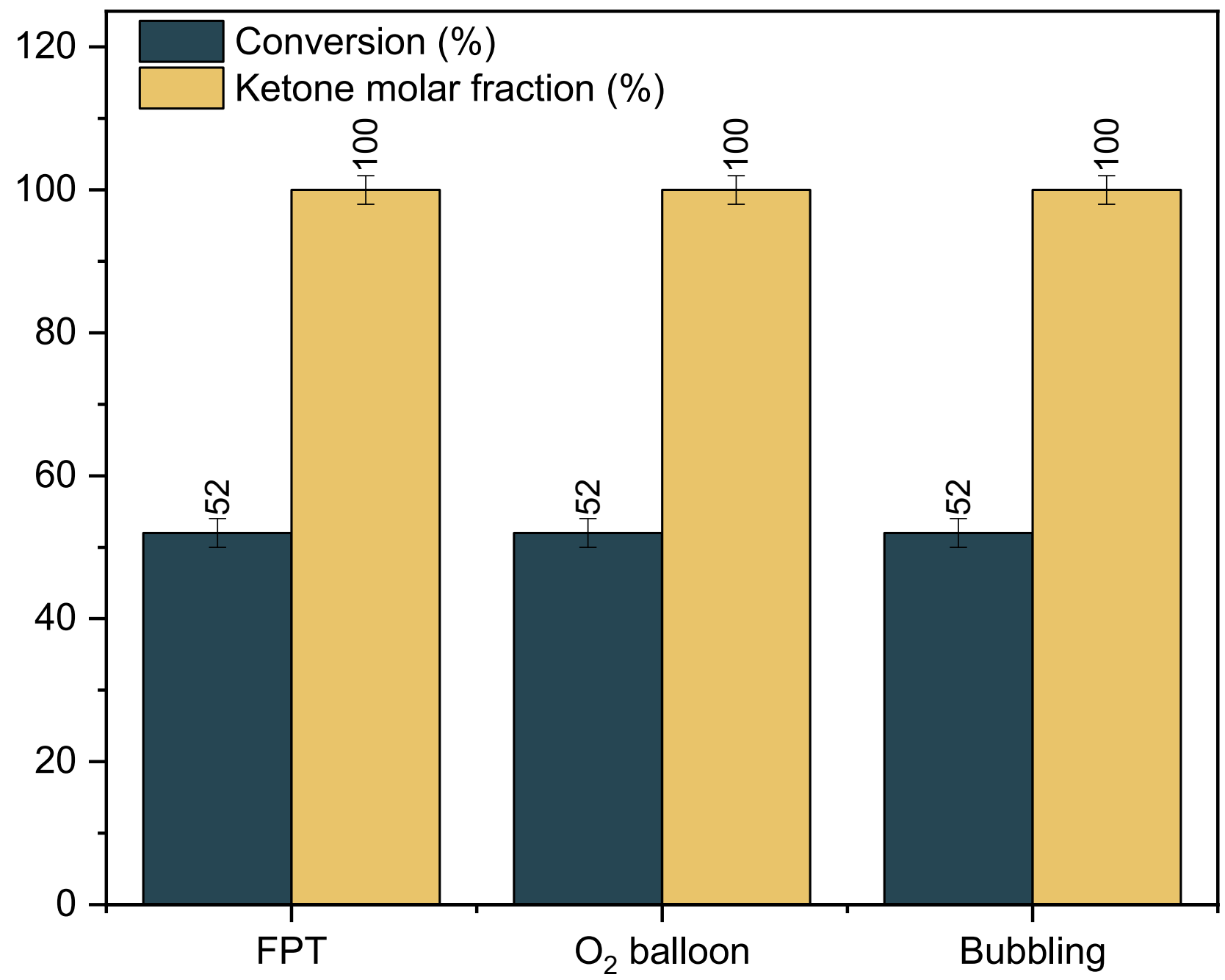

Figure S56. Screening of oxygen systems. The reaction conditions are shown in **Table S5**.

6.3. Photocatalysts

The data concerning the screening of photocatalysts are reported in **Table S6**.

Table S6. Screening of photocatalysts. Reaction conditions: solvent = DCM 0.1 M, catalyst loading = 250 ppm with respect to **13**, O_2 (bubbling), LED = 390 nm, temperature = r. t., time = 12 h.

Catalyst	Conversion (%)[a]	Dioxetane molar fraction (%)[a]	Ketone molar fraction (%)[a]
**Boron-salphen-Ph**	41	0	100
**Boron-salphen-Mes**	44	0	100
**Boron-salphen-CF$_3$**	44	0	100
**Boron-salphen-OMe**	52	0	100
**Salphen-OMe**	25	15	85

[a] Conversion evaluated by ^{1}H NMR using an internal standard, error = ± 2%.

6.4. Solvents

The data concerning the screening of solvents are reported in **Table S7**.

Table S7. Screening of solvents. Reaction conditions: 0.1 M, catalyst = **boron-salphen-OMe**, catalyst loading = 250 ppm with respect to **13**, O_2 (bubbling), LED = 390 nm, temperature = r. t., time = 12 h.

Solvent	Conversion (%)[a]	Dioxetane molar fraction (%)[a]	Ketone molar fraction (%)[a]
Dichloromethane	52	0	100
2-Methyltetrahydrofuran	10	0	100
Cyclopentyl methyl ether	4	0	100
Diethyl carbonate	30	0	100
Dimethyl carbonate	25	0	100
Hexane	15	0	100
Methyl *tert*-butyl ether	15	0	100
Acetonitrile	12	0	100
Acetone	23	0	100
Ethyl acetate	22	0	100
Toluene	37	0	100
Diethyl ether	17	0	100
Tetrahydrofuran	12	0	100

[a] Conversion evaluated by ^{1}H NMR using an internal standard, error = ± 2%.

6.5. Catalyst loading

The data concerning the screening of catalyst loadings are reported in **Table S8**.

Table S8. Screening of catalyst loadings. Reaction conditions: solvent = DCM 0.1 M, catalyst = **boron-salphen-OMe**, O_2 (bubbling), LED = 390 nm, temperature = r. t., time = 12 h.

Catalyst loading	Conversion (%)[a]	Dioxetane molar fraction (%)[a]	Ketone molar fraction (%)[a]
100 ppm	25	40	60
250 ppm	52	0	100
500 ppm	95	0	100
1000 ppm	98	0	100
2500 ppm	100	0	100

[a] Conversion evaluated by ^1H NMR using an internal standard, error = ± 2%.

6.6. Kinetics

The data concerning the screening of catalyst loadings are reported in **Table S9**.

Table S9. Kinetics. Reaction conditions: solvent = DCM 0.1 M, catalyst = **boron-salphen-OMe**, catalyst loading = 500 ppm with respect to **13**, O_2 (bubbling), LED = 390 nm, temperature = r. t.

Time	Conversion (%)[a]	Dioxetane molar fraction (%)[a]	Ketone molar fraction (%)[a]
0 h	0	0	0
1 h	3	100	0
2 h	8	89	11
3 h	17	79	21
4 h	28	68	32
6 h	41	60	40
9 h	69	48	52
12 h	95	16	84
14 h	100	0	100

[a] Conversion evaluated by ^1H NMR using an internal standard, error = ± 2%.

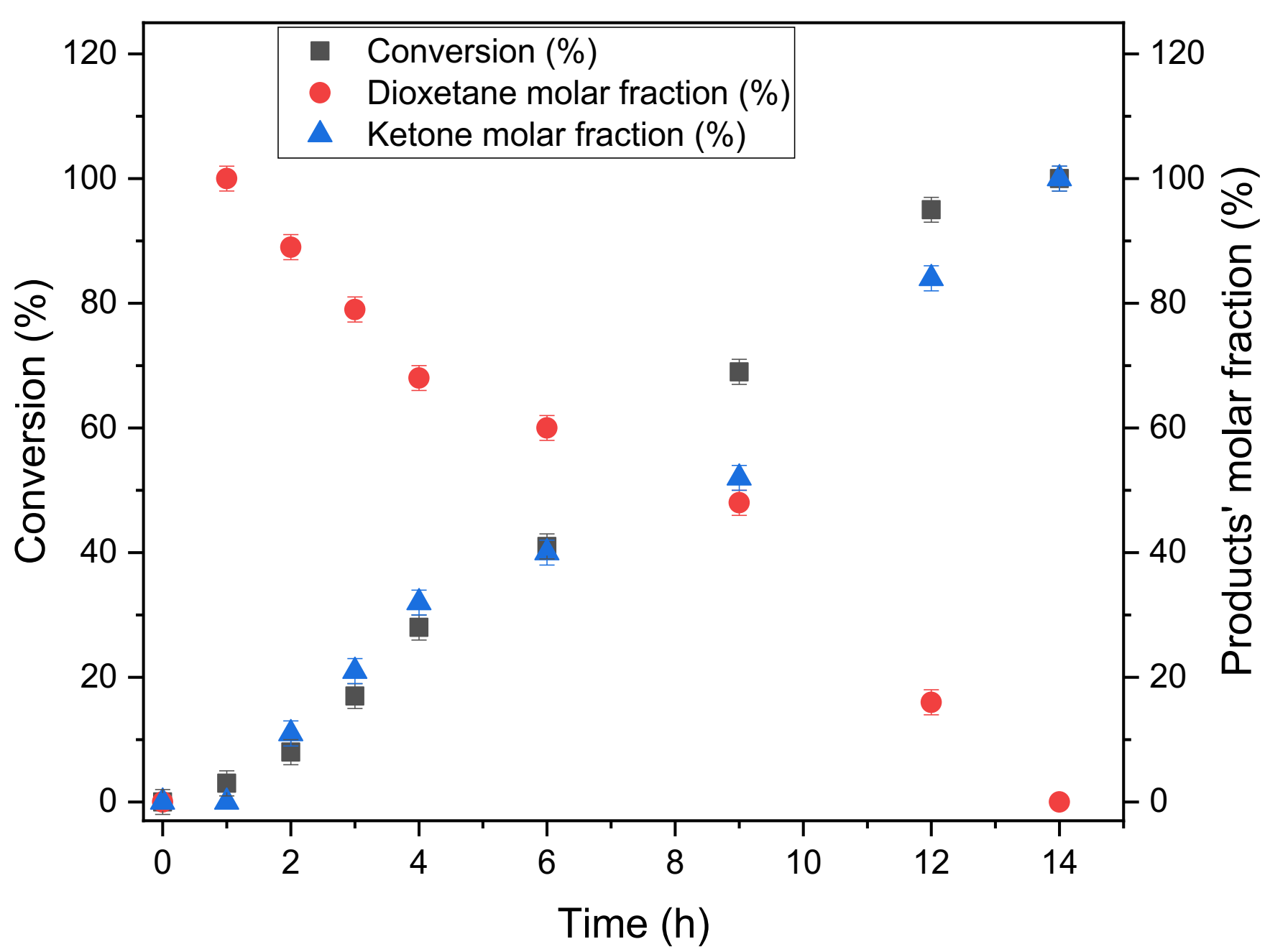

Figure S57. Kinetics. The reaction conditions are shown in **Table S9**.

6.7. Control experiments

The data concerning the control experiments are reported in **Table S10**.

Table S10. Control experiments. Reaction conditions: solvent = DCM 0.1 M, catalyst loading = 250 ppm with respect to **13**, O_2 (bubbling), LED = 390 nm, temperature = r. t., time = 12 h.

Control	Catalyst	Conversion (%)[a]	Dioxetane molar fraction (%)[a]	Ketone molar fraction (%)[a]
1[b]	**Boron-salphen-OMe**	0	0	0
2	None	5	0	100
3[c]	$BF_3{\cdot}Et_2O$	11	100	0
4[d]	**Boron-salphen-OMe**	6	0	100

[a] Conversion evaluated by 1H NMR using an internal standard, error = ± 2%. [b] Experiment performed in the absence of light. [c] Other side-products were observed. [d] Experiment performed with the addition of 1.0 equiv. of DABCO with respect to **13**.

7. Theoretical analysis

7.1. Computational details

Conformational sampling was carried out using CREST (Conform-Rotamer Ensemble Sampling Tool) v3.0.2,[69] employing the GFN-FF force field.[70] The resulting conformer ensembles were then re-optimized at the GFN2-xTB level of theory[71,72] with the ALPB (acetonitrile) implicit solvation model,[73] and subsequently filtered using the CREGEN (Conformer–Rotamer Ensemble GENeration) tool included in the CREST package. The obtained conformers were further optimized at the DFT level using the B97-3c functional with the SMD implicit solvation model[74] (THF or MeCN) employing ORCA v6.0.1. Frequency calculations were performed at this level to obtain thermal corrections (G_{thermo}) as well as the predicted IR spectra. All optimized structures were verified as stationary points on the potential energy surface; however, several conformers exhibited negligible imaginary frequencies (<-50 cm^{-1}) despite multiple re-optimization attempts. These cases are documented by providing the corresponding Cartesian coordinates. For the prediction of UV/Vis absorption spectra, TD-DFT calculations (with TDA approximation) were carried out on the optimized structures using the M06-2X/def2-TZVP level of theory with SMD solvation (THF). These calculations provided refined electronic energies (E_{elec}) and excitation energies. Final Gibbs free energies for each conformer were obtained from:

$$G_{Gibb} = E_{elec} + G_{thermo}$$

These Gibbs free energies were used to determine Boltzmann distribution of the conformers, and all predicted spectra were generated as Boltzmann-weighted averages over the conformers.

Redox potentials were computed following a literature[75] protocol based on B3LYP/6-31+G(d,p). Single-point electronic energies of oxidized and reduced species were calculated at the B3LYP/6-31+G(d,p)/SMD(MeCN) level using the previously optimized geometries.

The half-wave potentials were estimated according to:

$$E_{1/2}\ (\text{vs Fc/Fc}^{+}) = |E_{ox} - E_{red}| - 4.28 - 0.64$$

where 4.28 eV corresponds to the absolute potential of the SHE and 0.64 V converts to the Fc/Fc^{+} scale.

All geometries are collected in a Figshare repository with DOI 10.6084/m9.figshare.31702333.

7.2. HOMO-LUMO analysis

To compare the influence of π-extension on the electronic structure, salphen derivatives with their corresponding salen derivatives were computed (**Scheme S5** and **Figure S58**). The HOMO and LUMO of salphen derivatives are generally well delocalized over the entire conjugated backbone, showing efficient π-conjugation across the ligand framework. In contrast, although salen derivatives also exhibit π-type orbitals, their delocalization is more limited and does not extend across the full molecular backbone. These results highlight the efficient conjugation and electronic distribution in the salphen scaffold relative to the salen scaffold. In agreement with the observed orbital distribution, the calculated HOMO-LUMO energy gaps (**Table S11**) are consistently smaller for the salphen derivatives than for the corresponding salen analogues. The reduced energy

gaps in the salphen series can be attributed to increased π-conjugation, which causes more effective LUMO stabilization through enhanced delocalization. Accordingly, structural modification of the ligand framework *via* π-extension leads to significant modulation of the electronic structure.

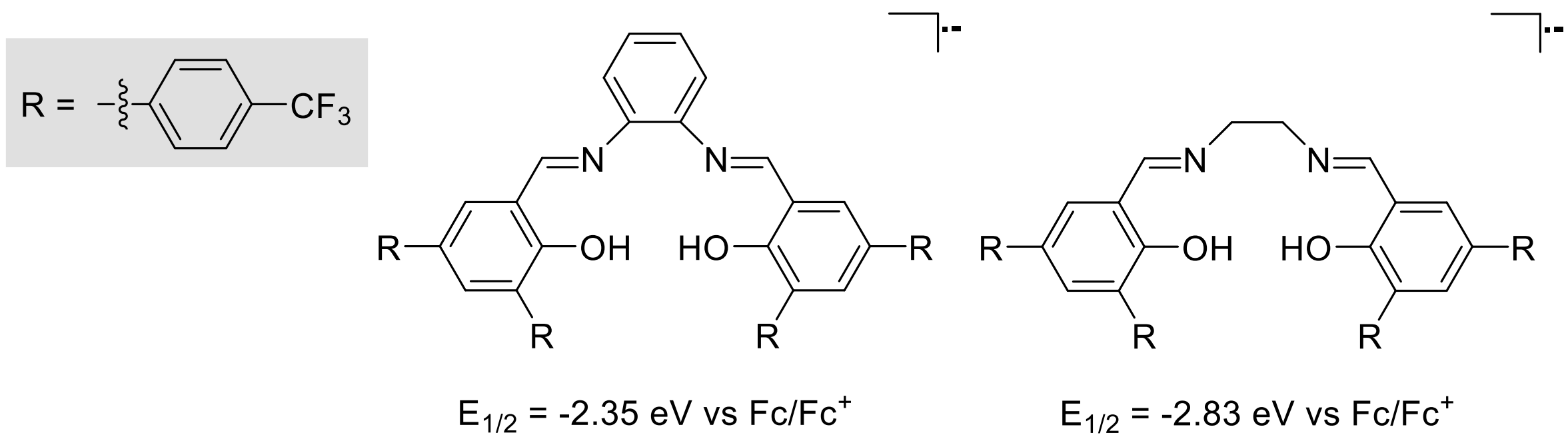

Scheme S5. Half-wave potential computed for **salphen-CF_3** and **salen-CF_3** at the B3LYP/6-31+G(d,p)/SMD(MeCN)//B97-3c(MeCN) level of theory.

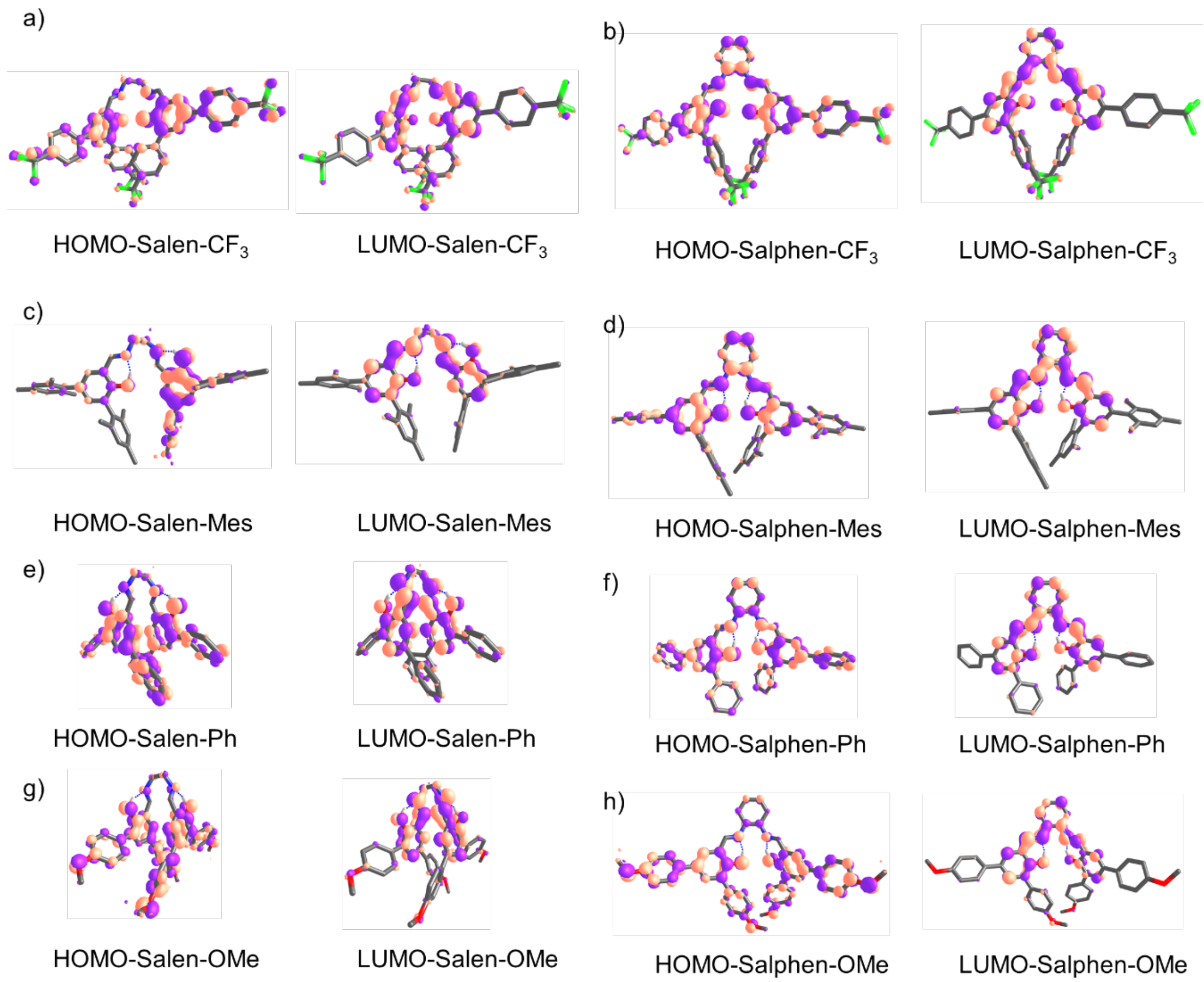

Figure S58. Visualized HOMO and LUMO orbitals of salen and salphen derivatives.

Table S11. The HOMO-LUMO gap with different salen and salphen derivatives.

	Salen			Salphen		
Substituents	HOMO	LUMO	gap	HOMO	LUMO	gap
p-C_6H_4-CF_3	-5.20	-2.35	2.85	-5.12	-2.77	2.35
Mes	-5.10	-2.17	2.93	-5.00	-2.71	2.40
Ph	-4.67	-2.19	2.93	-4.72	-2.61	2.29
p-C_6H_4-OMe	-5.10	-2.17	2.48	-5.05	-2.65	2.11

The relative stability of the **salphen-R** (CF_3, Mes or OMe) radical species compared to **salphen-Ph** radical was computed with B97-3c/SMD(MeCN). The Gibbs free energy difference (**Table S12**) was defined as:

$$\Delta G = G(\textbf{salphen-Ph} + \textbf{salphen-R}^{\bullet}) - G(\textbf{salphen-Ph}^{\bullet} + \textbf{salphen-R})$$

such that negative values indicate an energetic preference for formation of the substituted radical (**salphen-R$^{\bullet}$**) relative to the **salphen-Ph** radical reference, whereas positive values indicate the opposite.

Table S12. Relative Gibbs free energy for radical stability.

R	$\Delta \boldsymbol{G}_{\{(\boldsymbol{Ph}+\boldsymbol{R}^{\cdot})-(\boldsymbol{Ph}^{\cdot}+\boldsymbol{R})\}}$ (kcal/mol)
**Salphen-CF_3**	-2.5
**Salphen-Mes**	1.2
**Salphen-OMe**	1.2

7.3. IR analysis

Experimentally, a prominent IR band at 1391 cm^{-1} appears and grows continuously at increasingly negative potentials. Isotopic labelling experiments using $^{13}CO_2$ lead to a clear shift of this band to 1361 cm^{-1}, supporting its assignment to a CO_2-derived carbamate intermediate.

To validate these experimental observations, several plausible CO_2(H) adduct intermediates were examined computationally, including neutral, singly reduced, and doubly reduced species bearing either CO_2 or CO_2H adducts (**Scheme S6**).

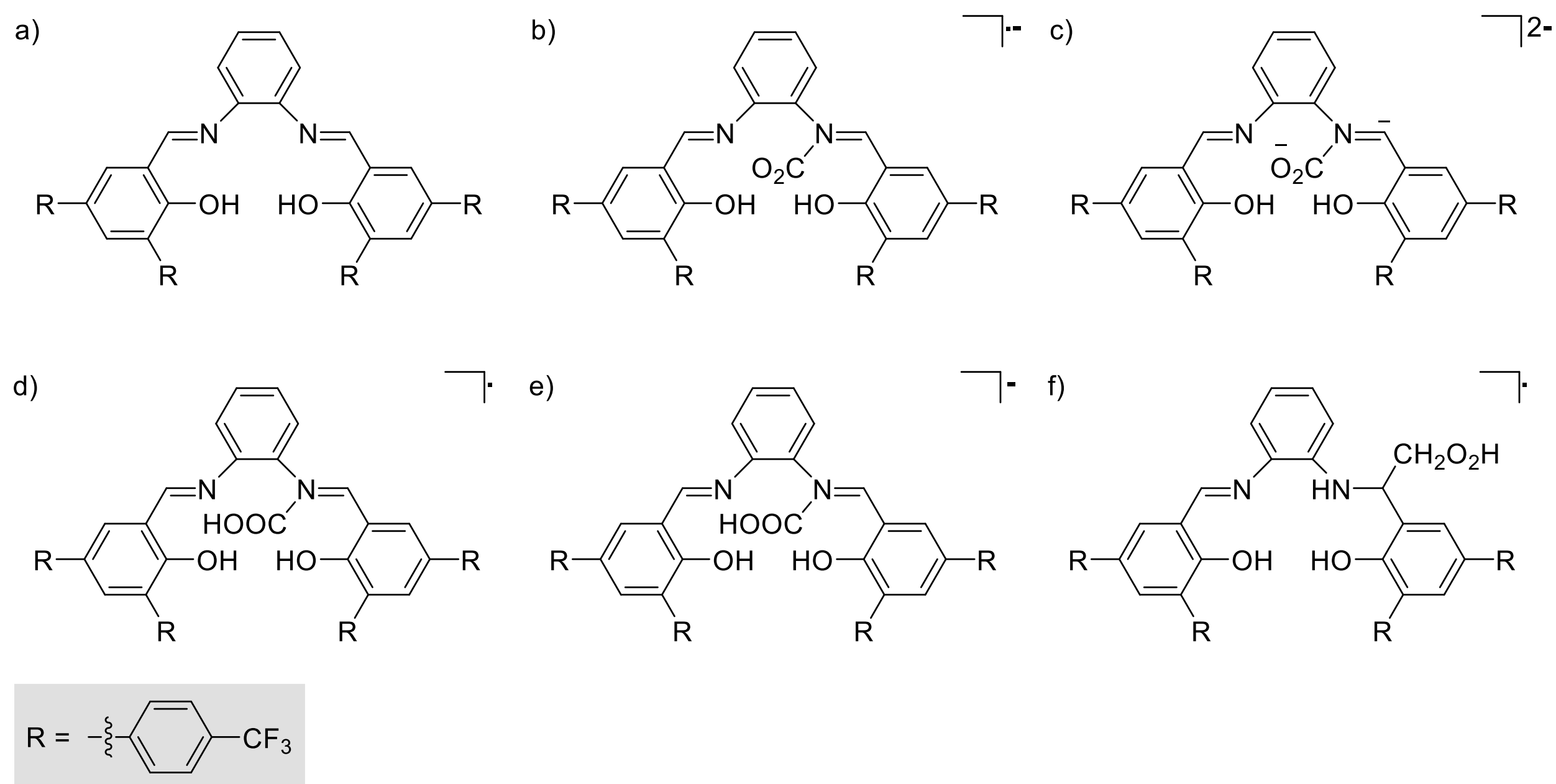

Scheme S6. Different CO_2(H) adduct intermediates of **salphen-CF$_3$** computed in this study. We considered: **a**) the neutral ligand; **b**) the singly reduced species bonded with CO_2; **c**) the doubly reduced species bonded with CO_2; **d**) the singly reduced, protonated, species bonded with CO_2; **e**) the doubly reduced, protonated, species bonded with CO_2; **f**) the singly reduced, protonated, species bonded with CO_2 with attack at the carbon instead of the CC=N nitrogen.

In all cases, we analyzed the IR obtained after Boltzmann weighing all conformers obtained in our preliminary screening (**Figure S59**). For species **b** and **c**, no meaningful signals related to -NCO_2^- vibrational mode were observed around 1300-1600 cm^{-1} (**Figure S59**, red and blue traces respectively). Notably, the calculated IR spectra show that singly reduced **salphen-CF$_3$** species with N-bound CO_2H adduct (**d**) exhibit -NCO_2H based vibrational modes around 1390 cm^{-1}, which shows a good agreement with experimental observation (**Figure 5** in the main text; **Figure S59** green trace). The vibrational mode is a combination between a bending of the N-C bond and a wagging of the C-O-H angle. A similar vibrational mode was also observed in the doubly reduced same adduct (**e**) (**Figure S59** purple trace); however, the redox potential of the species was calculated to be -2.83 eV, which would be challenging to achieve under the given

experimental conditions. Additionally, the carbons at the imine (C=N) were also considered as a potential binding site for CO_2 attachment (**f**), as reported in related systems.[76] However, the corresponding -CCO_2H vibrational modes were calculated near 1160 cm^{-1}, far from the experimentally observed wavenumbers. Therefore, we propose **d** as the most plausible species for this CO_2 activation system. **Scheme S7** shows our mechanistic hypothesis. The salphen ligand is reduced, forming its corresponding radical anion. The single-electron reduction activates catalysts that can interact with CO_2 dissolved in the reaction mixture, forming a C-N bond. Protonation of the activated CO_2 follows, possibly thanks to the mediation of the hydroxy groups on the phenol moieties of the catalyst.

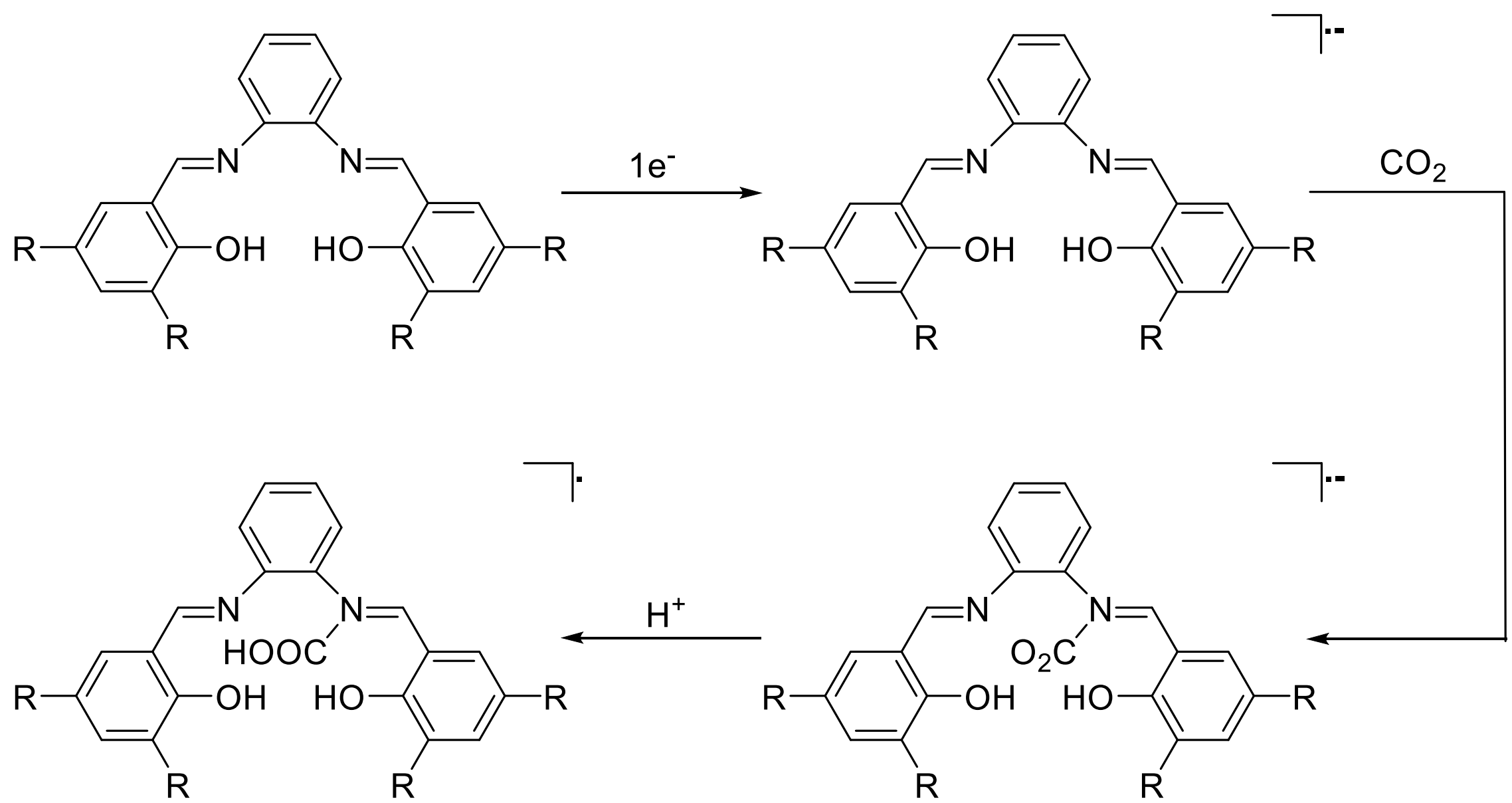

Scheme S7. Mechanistic hypothesis for the stepwise CO_2 addition to the salphen ligands.

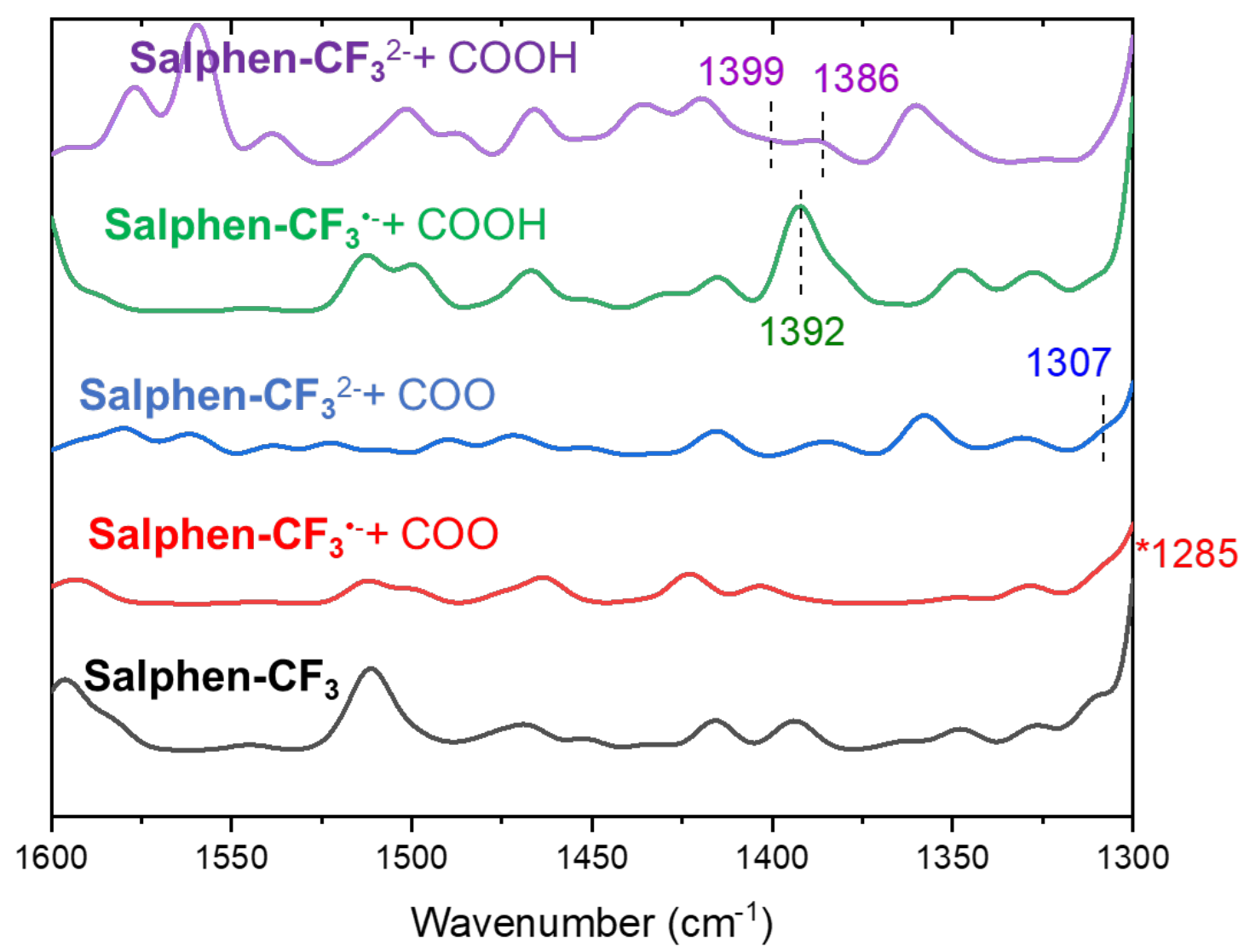

Figure S59. IR spectra of reduced **salphen-CF$_3$** with COO(H) adducts. The labelled peaks are assigned to vibrational modes associated with the -COO- group.

7.4. TD-DFT analysis

The experimentally investigated salphen derivatives and their corresponding boron adducts were subjected to computational analysis to check the effect of boron coordination on their electronic structures. As experimentally demonstrated by red shifts in the absorption spectra upon boron coordination, complexation changes the electronic structure of the π-conjugated framework. The computational results confirm this trend, showing systematic red shifts for the boron-salphen complexes relative to the free ligands, in good agreement with the experimental observations (**Figure S60**).

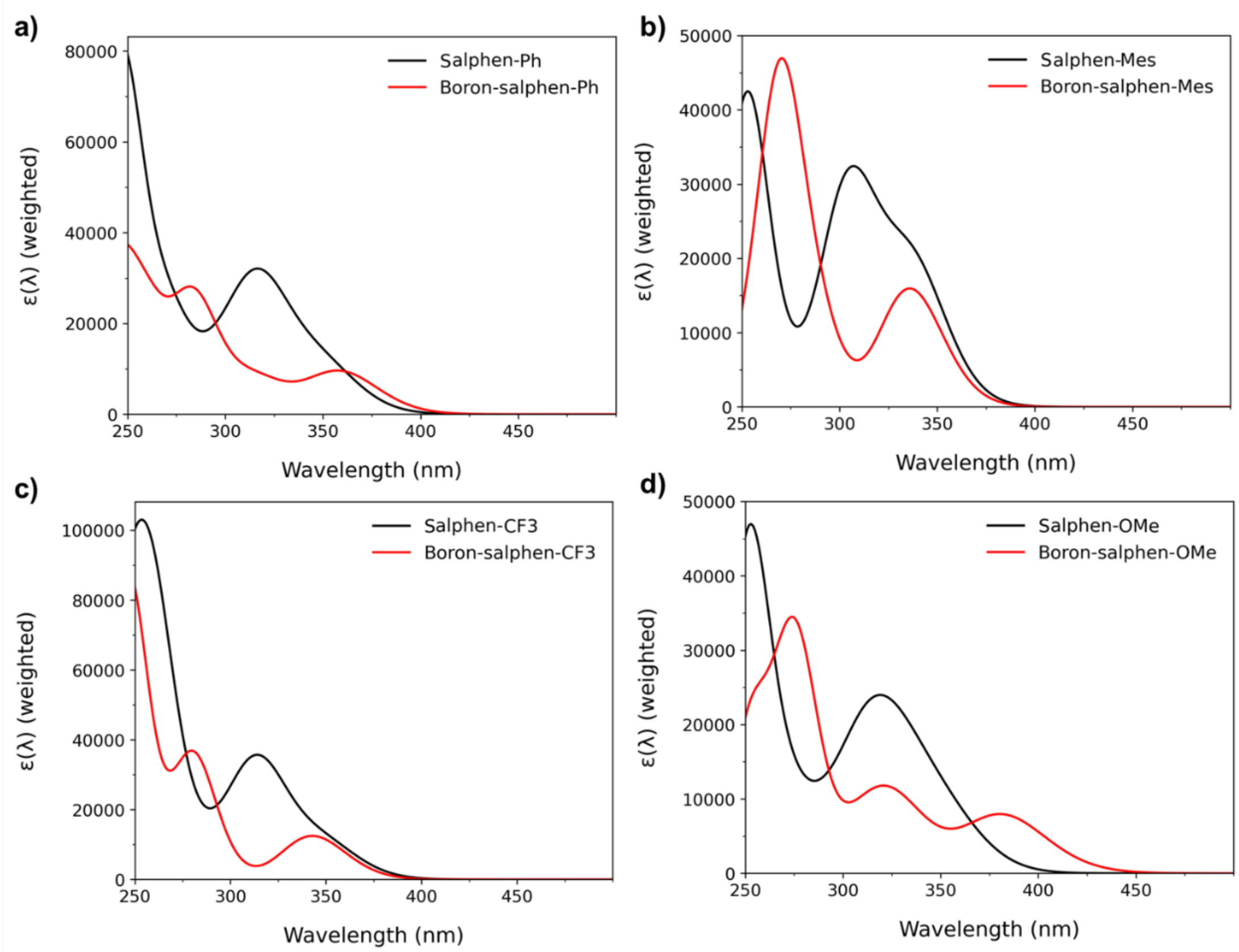

Figure S60. Calculated UV/Vis absorption spectra of salphen (black) and boron-salphen (red) derivatives bearing (**a**) -Ph, (**b**) -Mes, (**c**) -*p*-C_6H_4-CF_3, and (**d**) -*p*-C_6H_4-OMe substituents. The spectra were obtained from TD-DFT calculations at the M06-2X/def2-TZVP/SMD(THF)//B97-3c/SMD(THF) level. All provided spectra are Boltzmann-weighted over all the optimized conformers.

8. NMR spectra

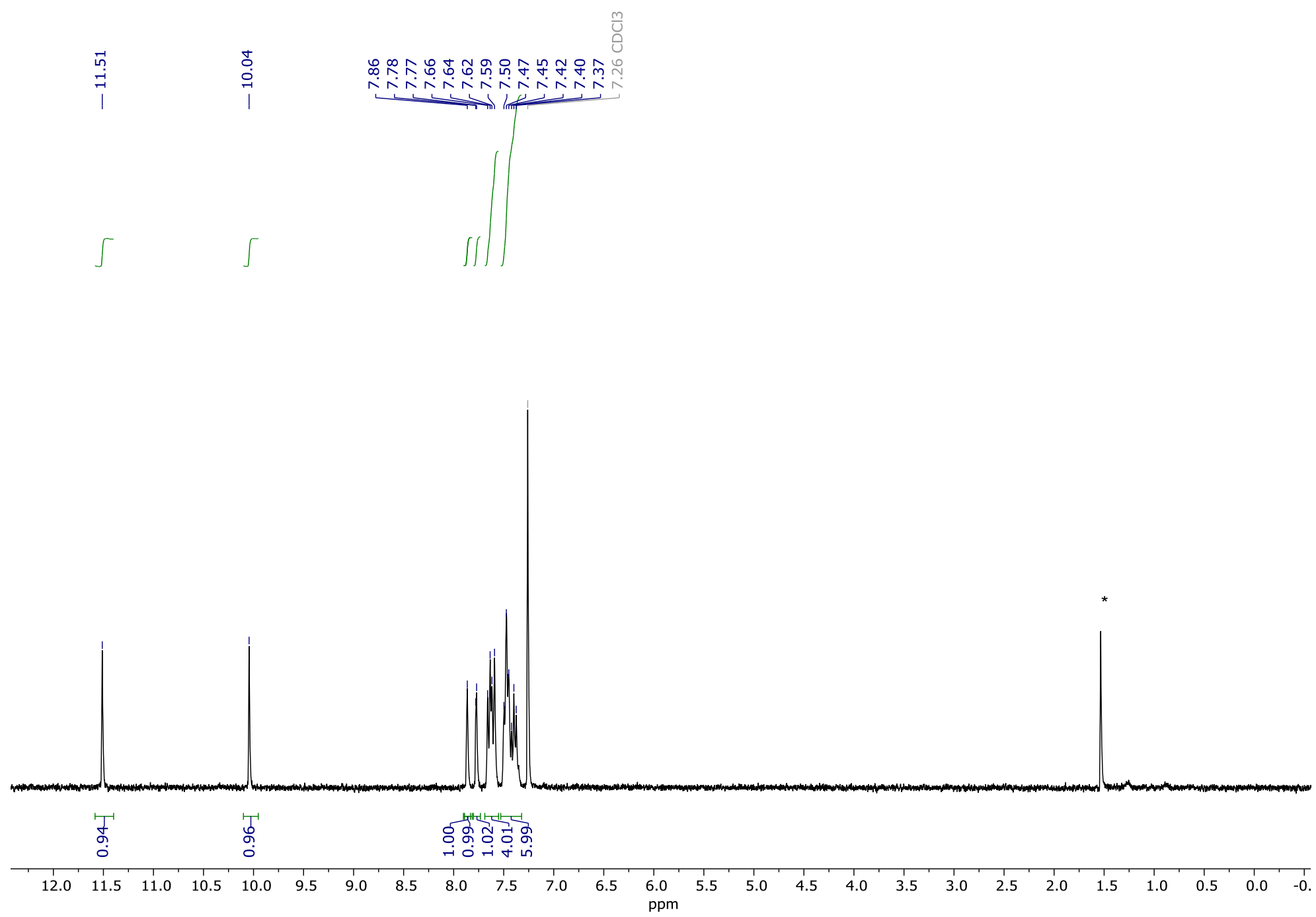

Figure S61. ^{1}H NMR (300 MHz) spectrum of salicylaldehyde **1** in $CDCl_3$. The symbol (*) marks water.

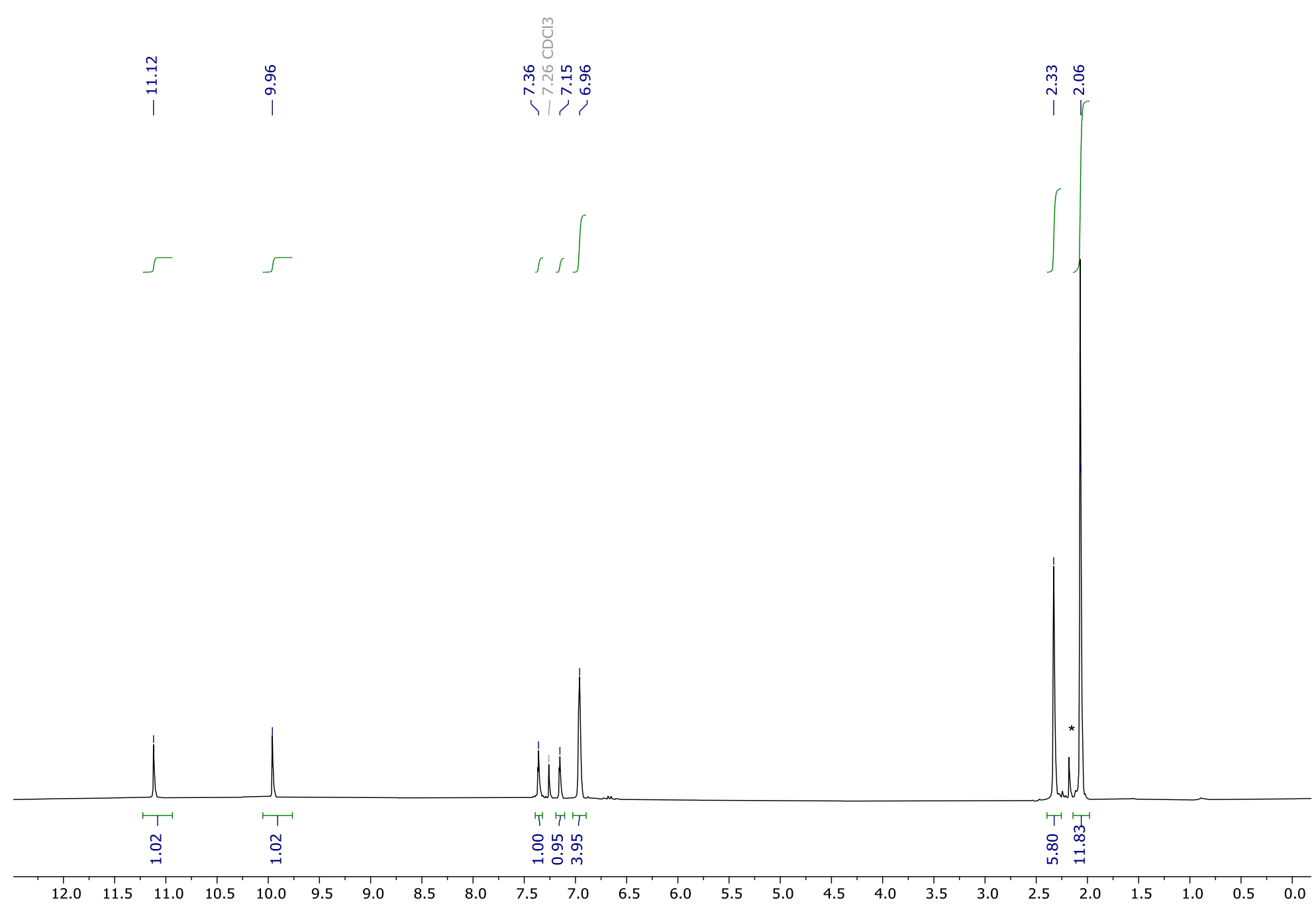

Figure S62. ^{1}H NMR (300 MHz) spectrum of salicylaldehyde **2** in $CDCl_3$. The symbol (*) marks water.

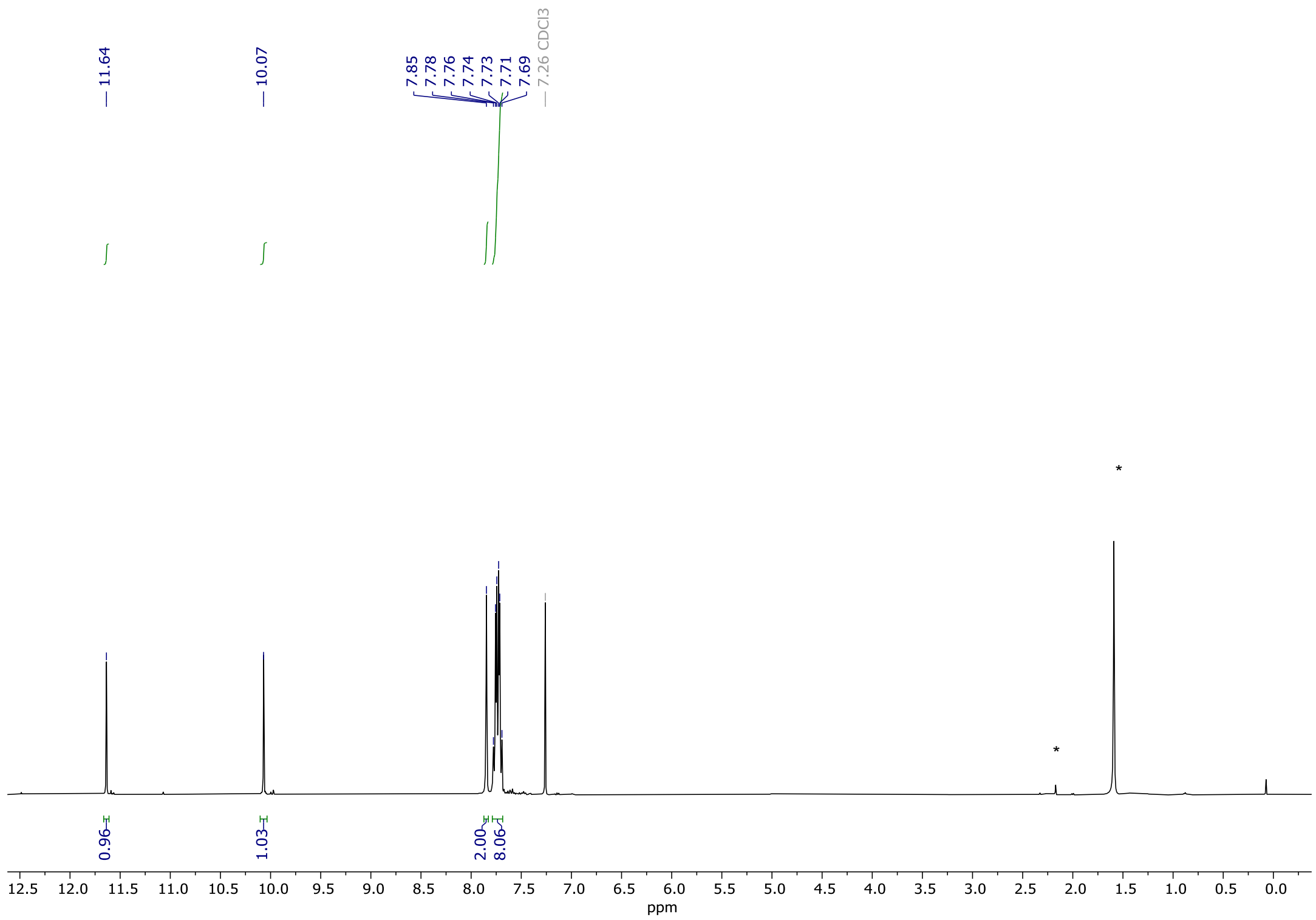

Figure S63. ^{1}H NMR (400 MHz) spectrum of salicylaldehyde **3** in $CDCl_3$. The symbol (*) marks acetone and water.

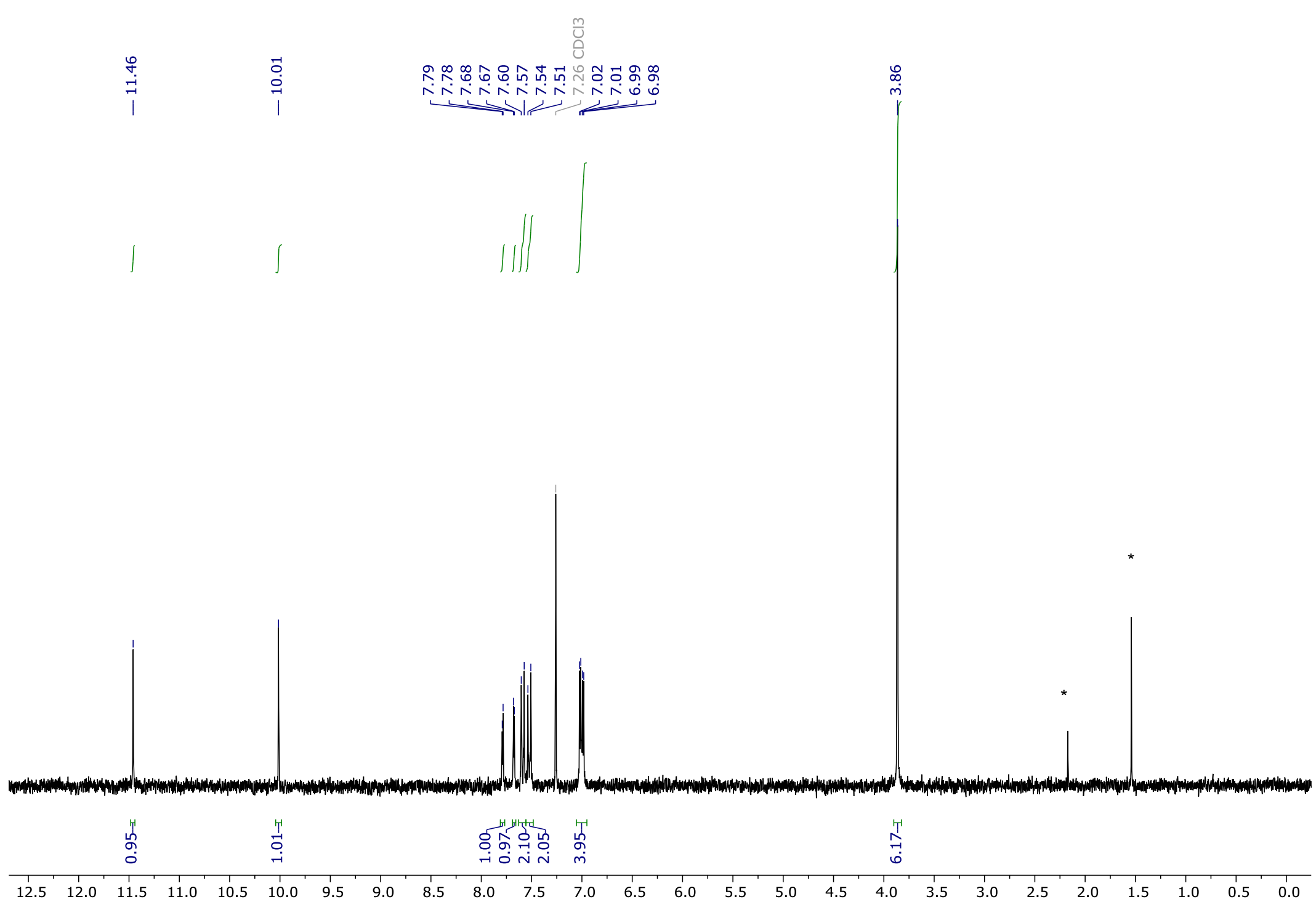

Figure S64. ^{1}H NMR (300 MHz) spectrum of salicylaldehyde **4** in $CDCl_3$. The symbol (*) marks acetone and water.

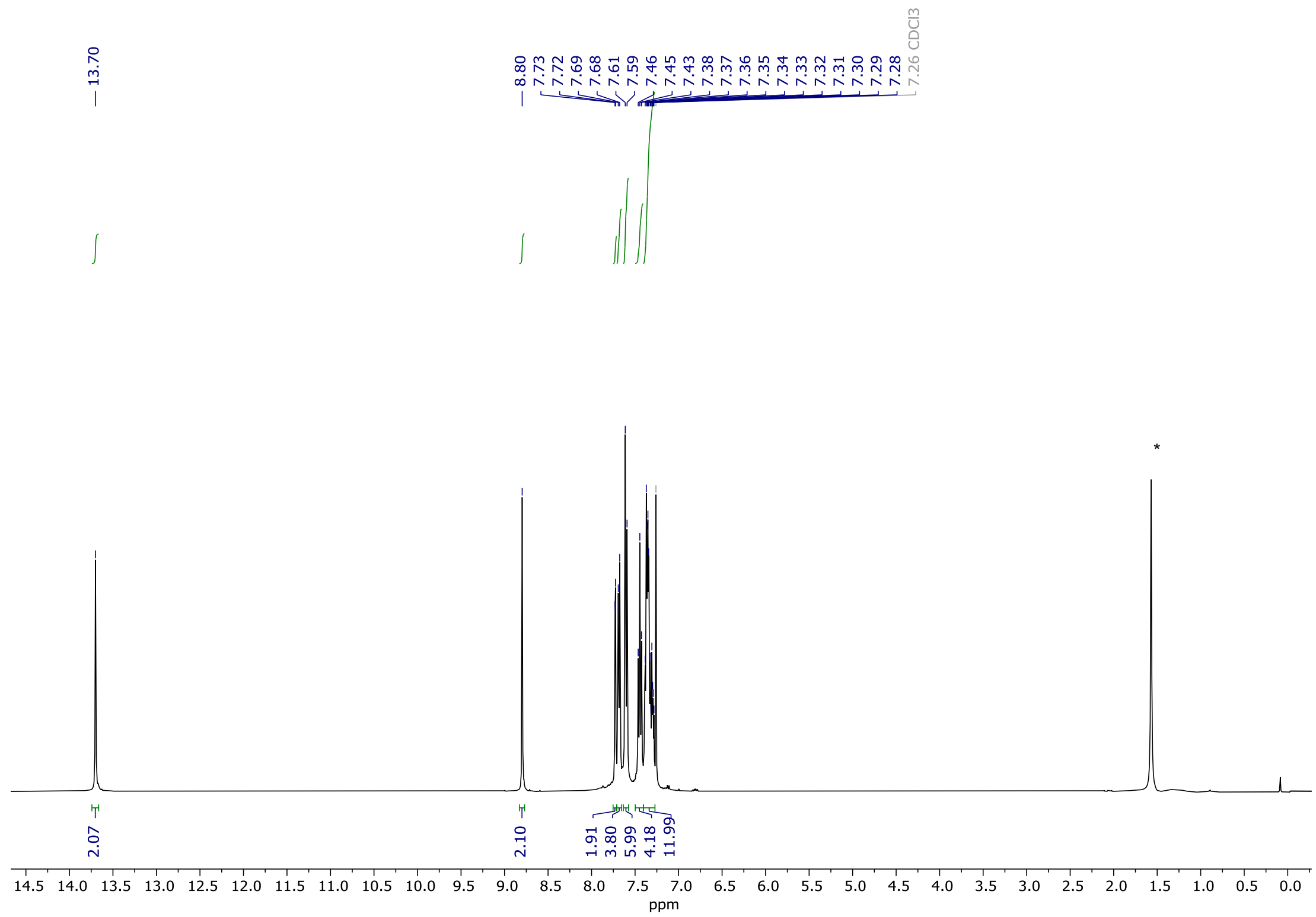

Figure S65. ^{1}H NMR (400 MHz) spectrum of salphen ligand **5** in $CDCl_3$. The symbol (*) marks water.

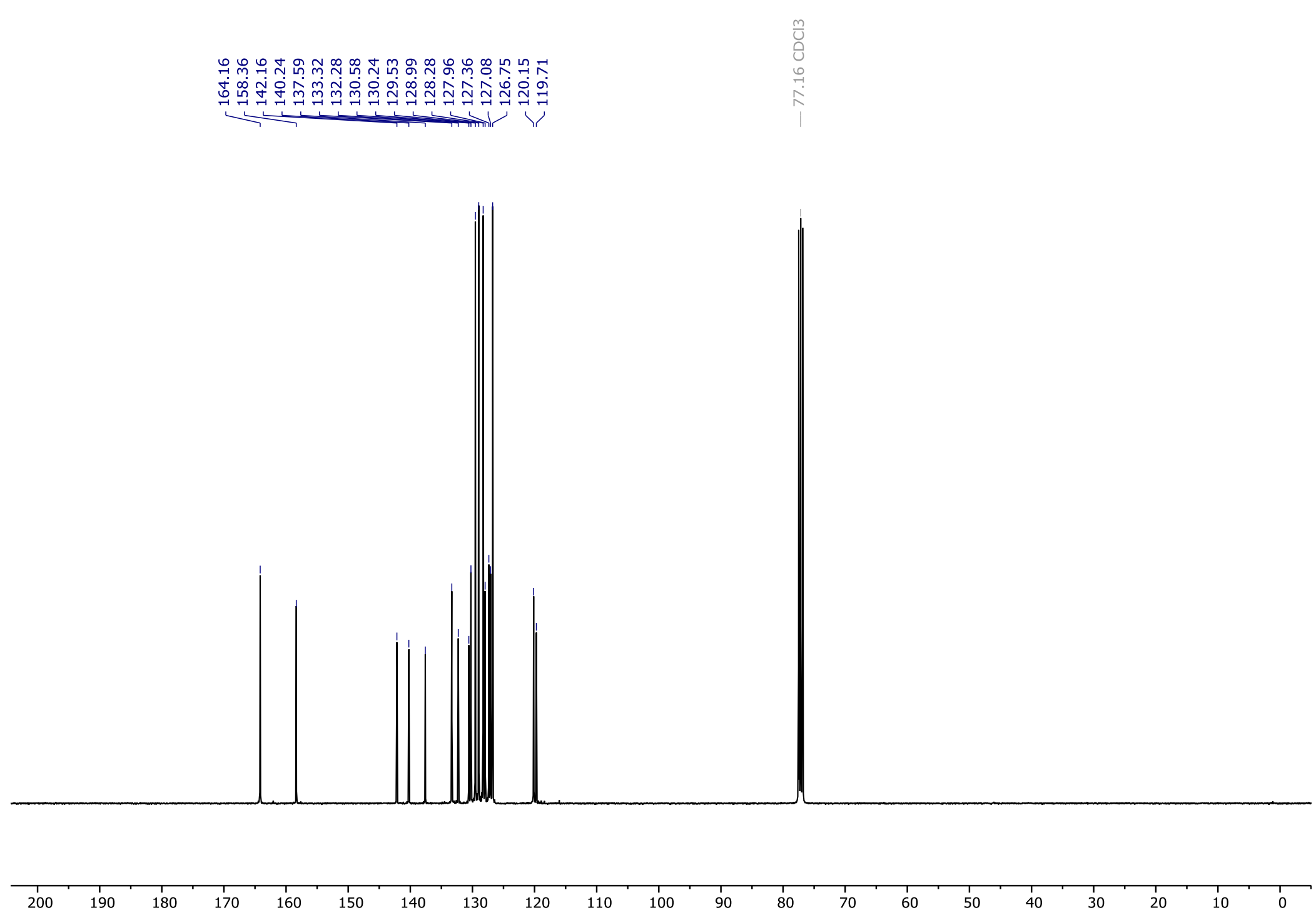

Figure S66. $^{13}C\{^{1}H\}$ NMR (101 MHz) spectrum of salphen ligand **5** in $CDCl_3$.

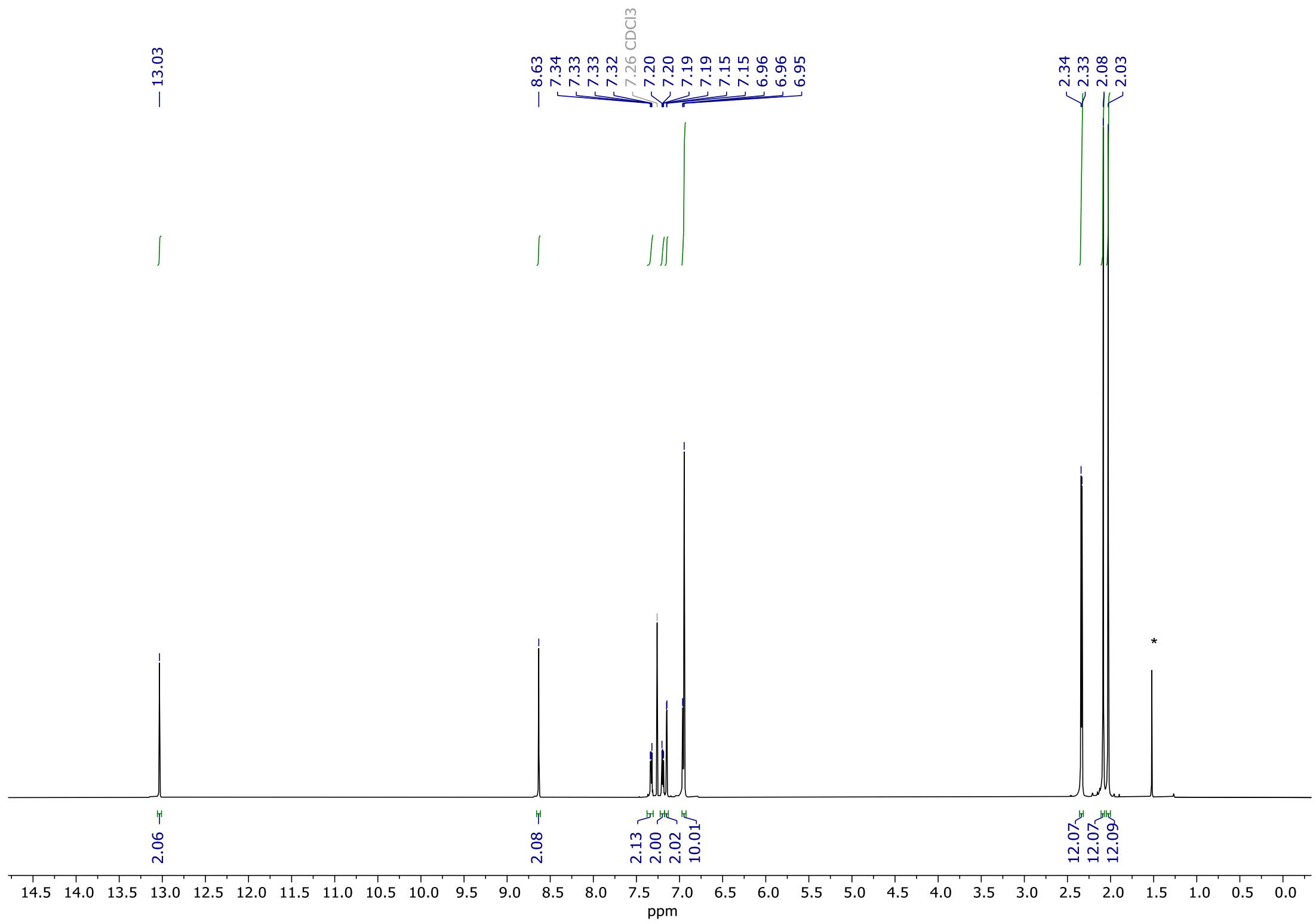

Figure S67. ^{1}H NMR (500 MHz) spectrum of salphen ligand **6** in $CDCl_3$. The symbol (*) marks water.

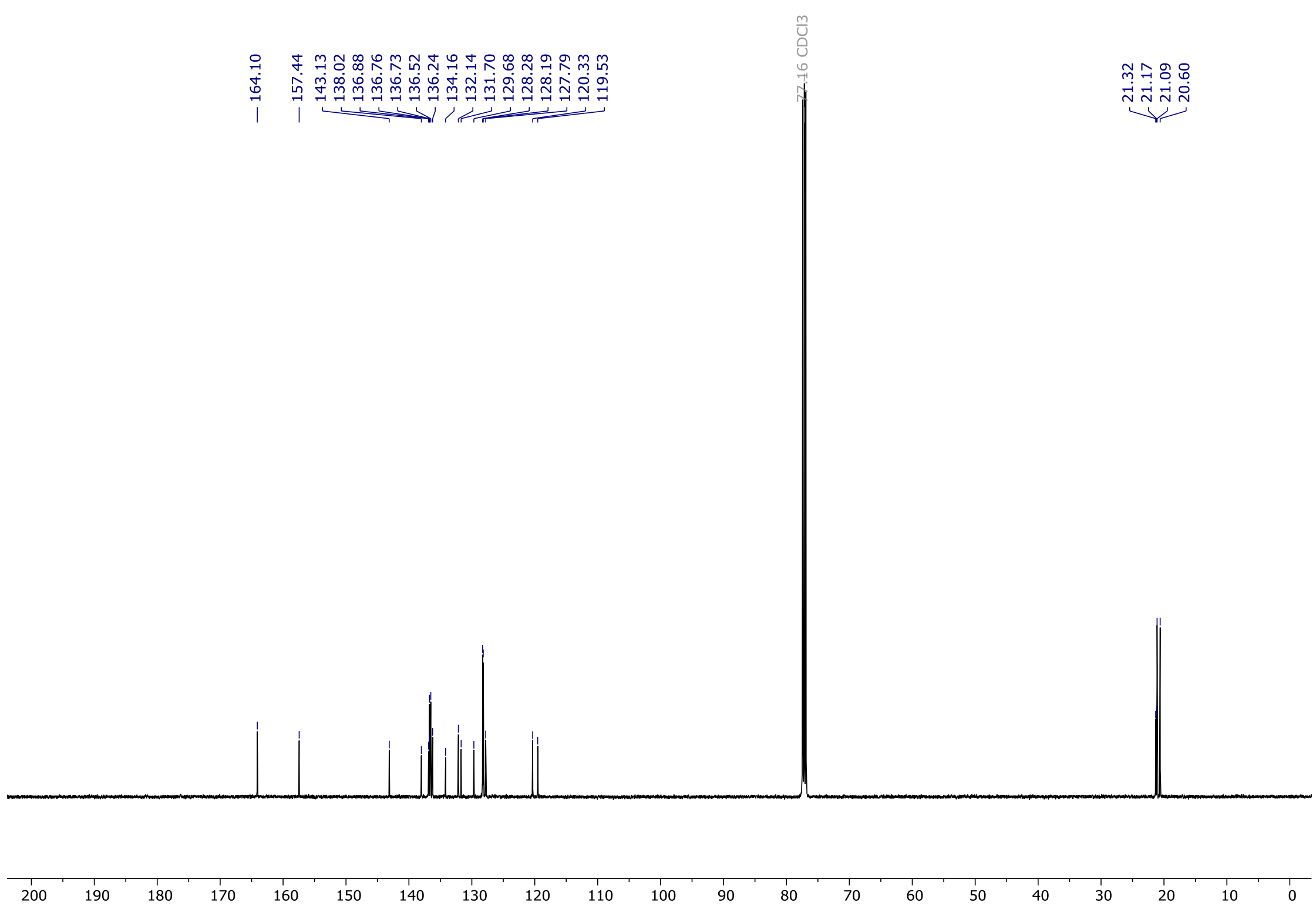

Figure S68. $^{13}C\{^{1}H\}$ NMR (126 MHz) spectrum of salphen ligand **6** in $CDCl_3$.

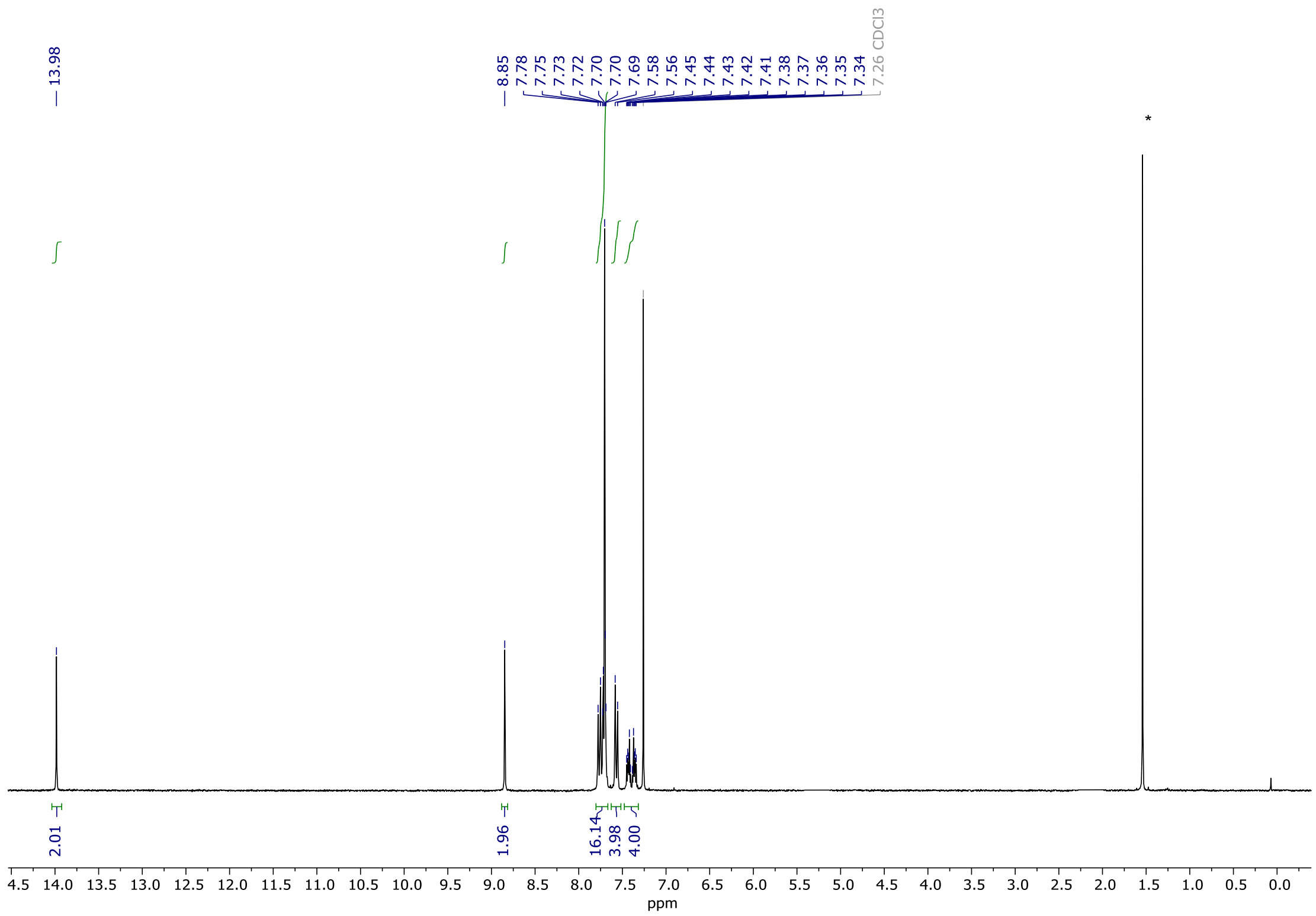

Figure S69. ^{1}H NMR (300 MHz) spectrum of salphen ligand **7** in $CDCl_3$. The symbol (*) marks water.

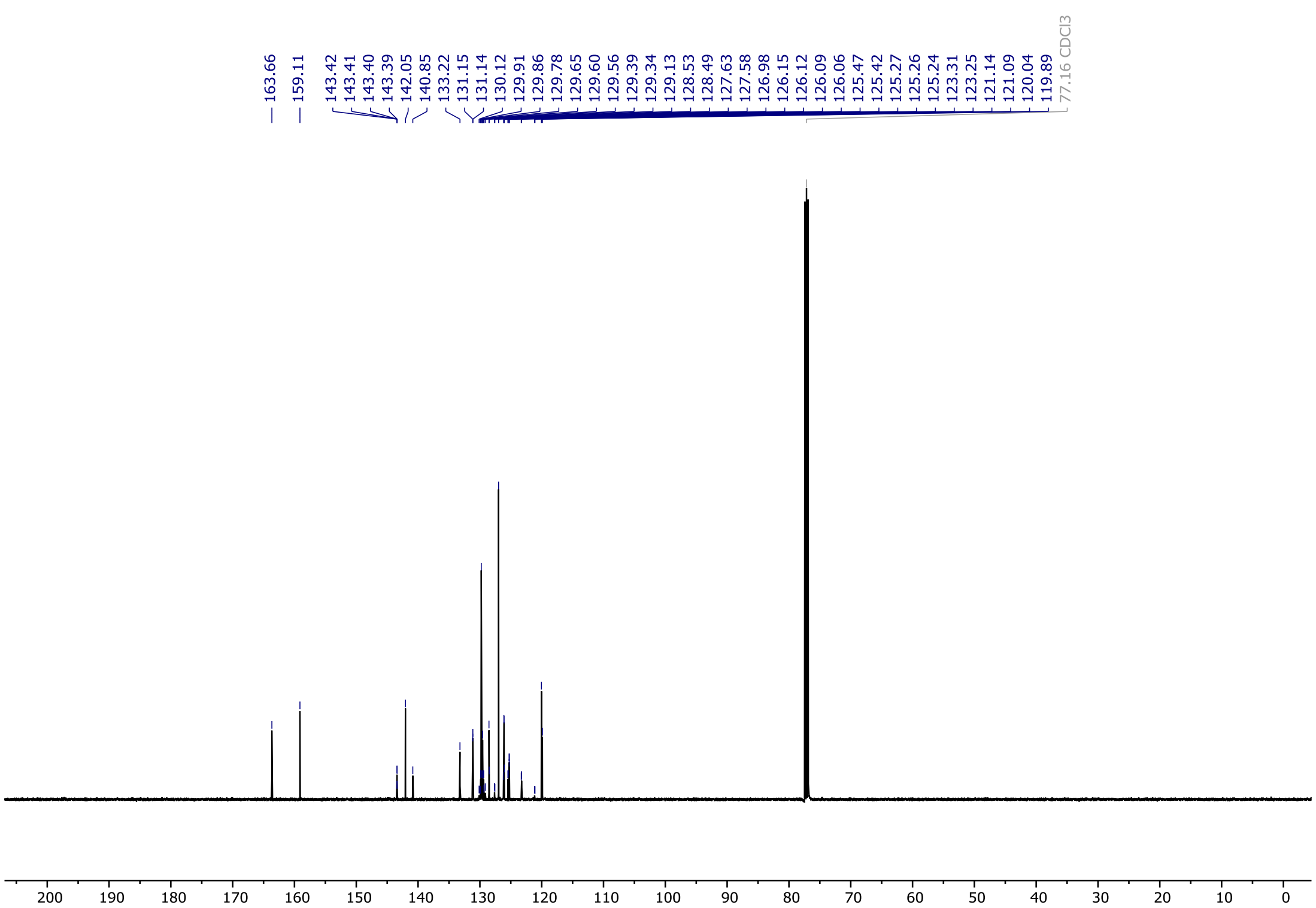

Figure S70. $^{13}C\{^{1}H\}$ NMR (126 MHz) spectrum of salphen ligand **7** in $CDCl_3$.

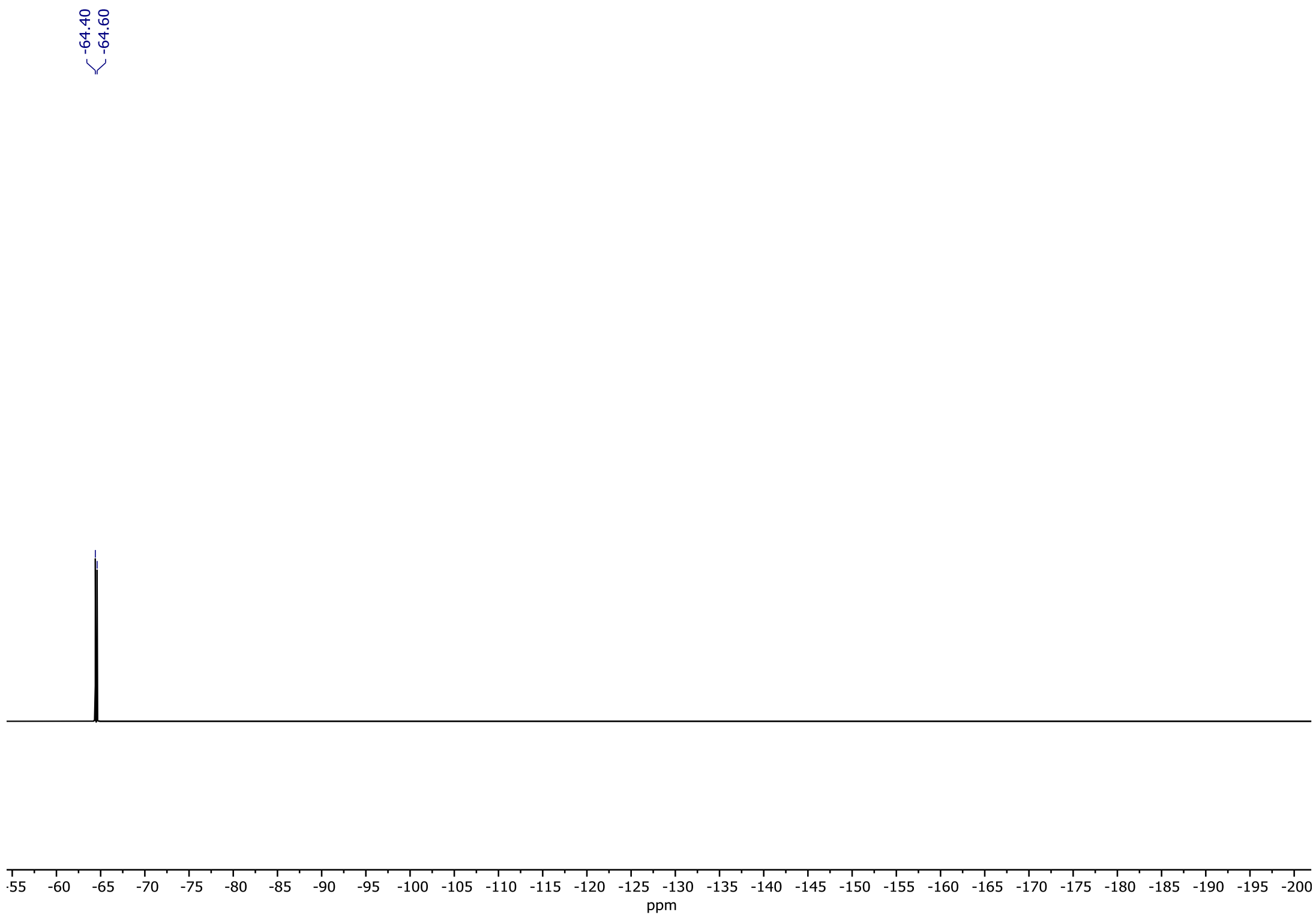

Figure S71. $^{19}F\{^1H\}$ NMR (470 MHz) spectrum of salphen ligand **7** in $CDCl_3$.

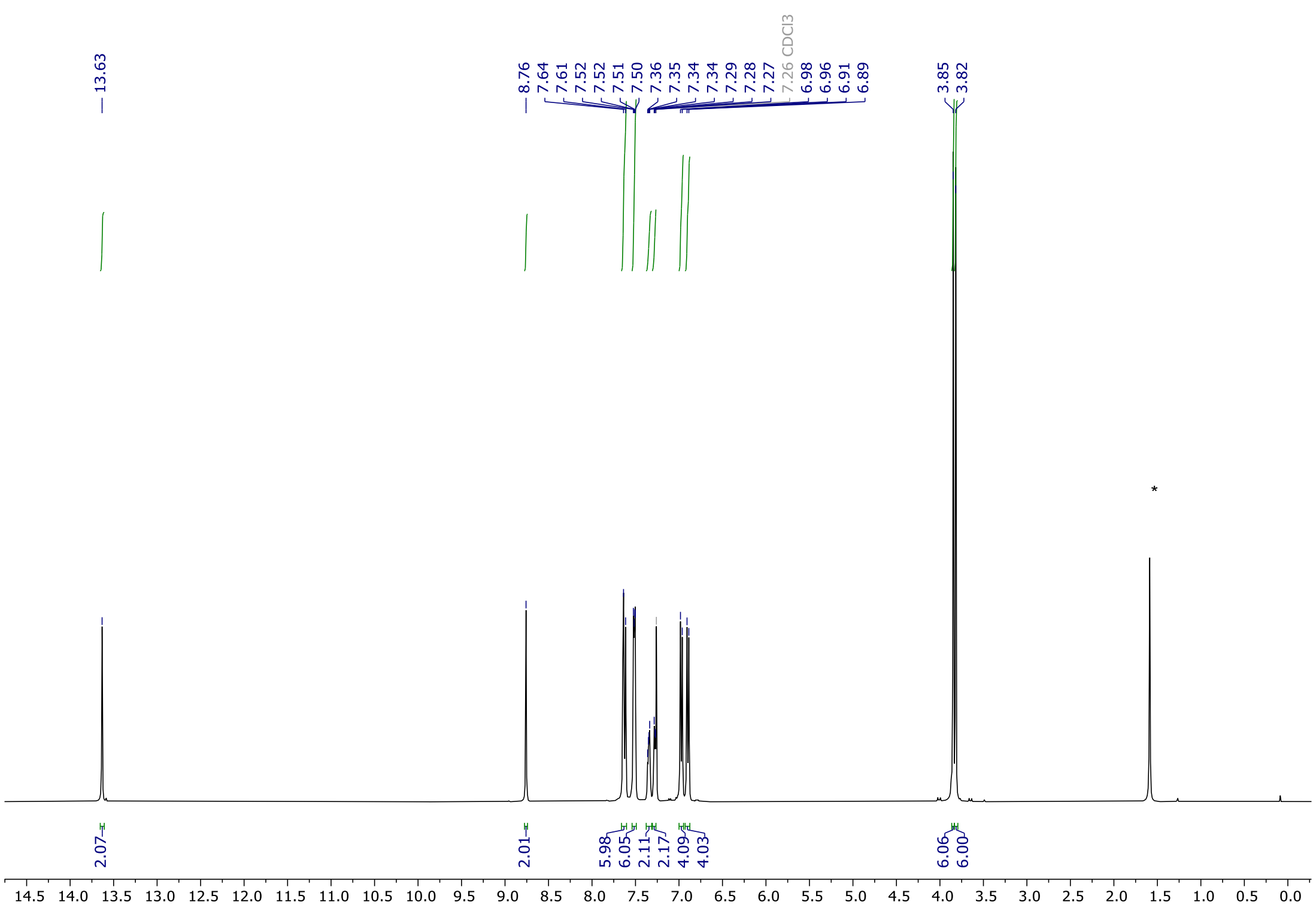

Figure S72. ^1H NMR (400 MHz) spectrum of salphen ligand **8** in $CDCl_3$. The symbol (*) marks water.

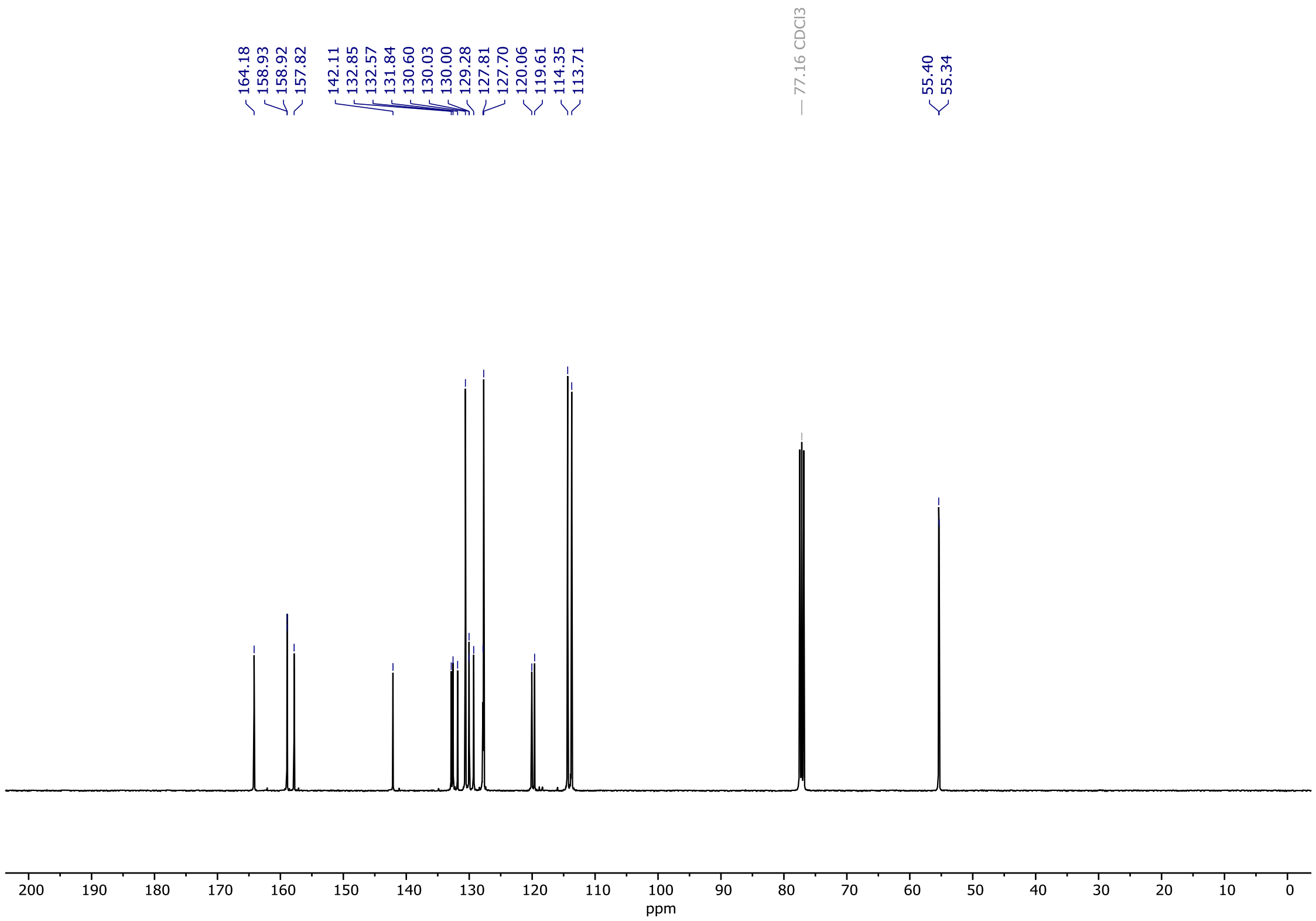

Figure S73. $^{13}C\{^{1}H\}$ NMR (101 MHz) spectrum of salphen ligand **8** in $CDCl_3$.

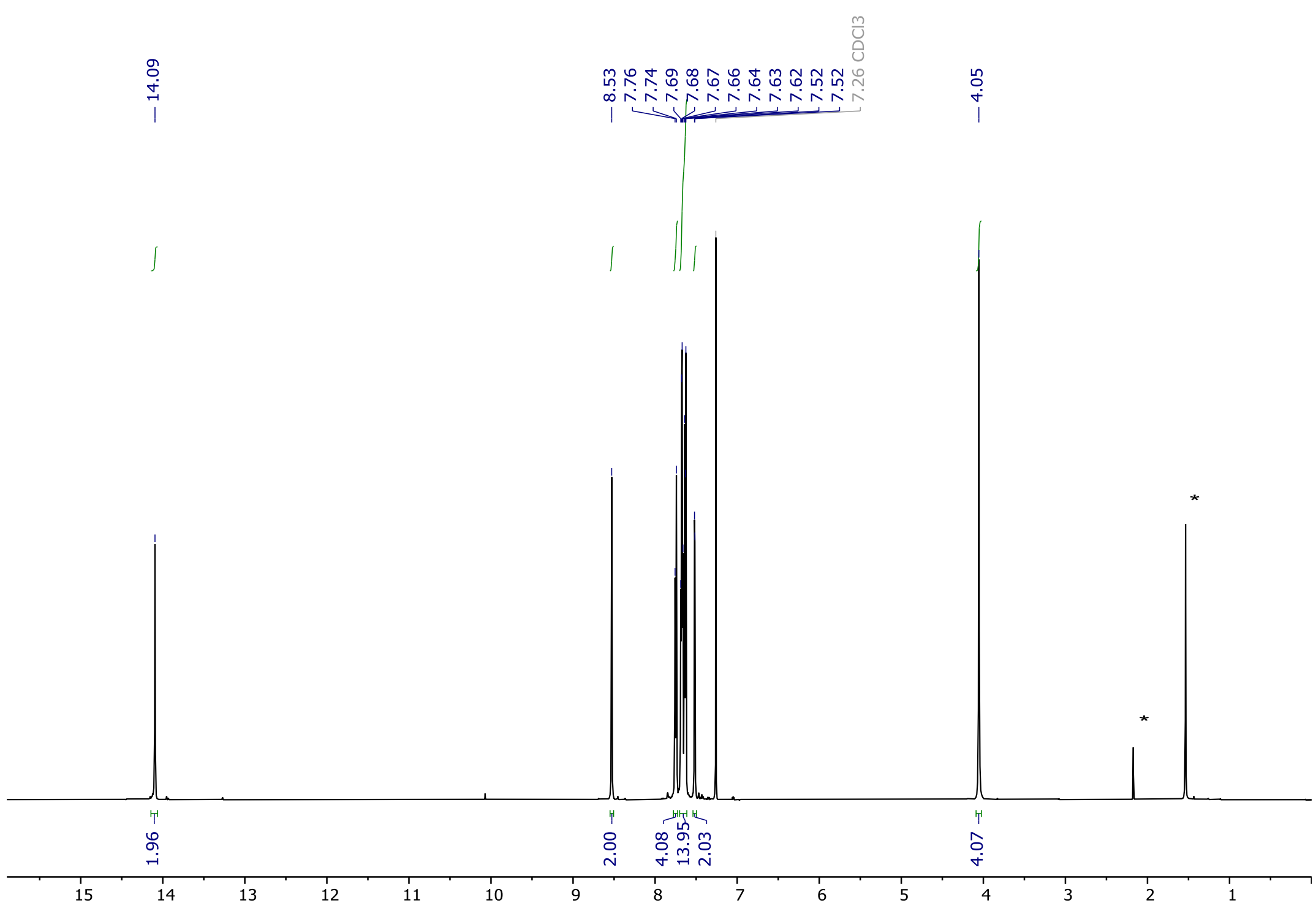

Figure S74. ^{1}H NMR (500 MHz) spectrum of salen ligand **7'** in $CDCl_3$. The symbol (*) marks water and acetone.

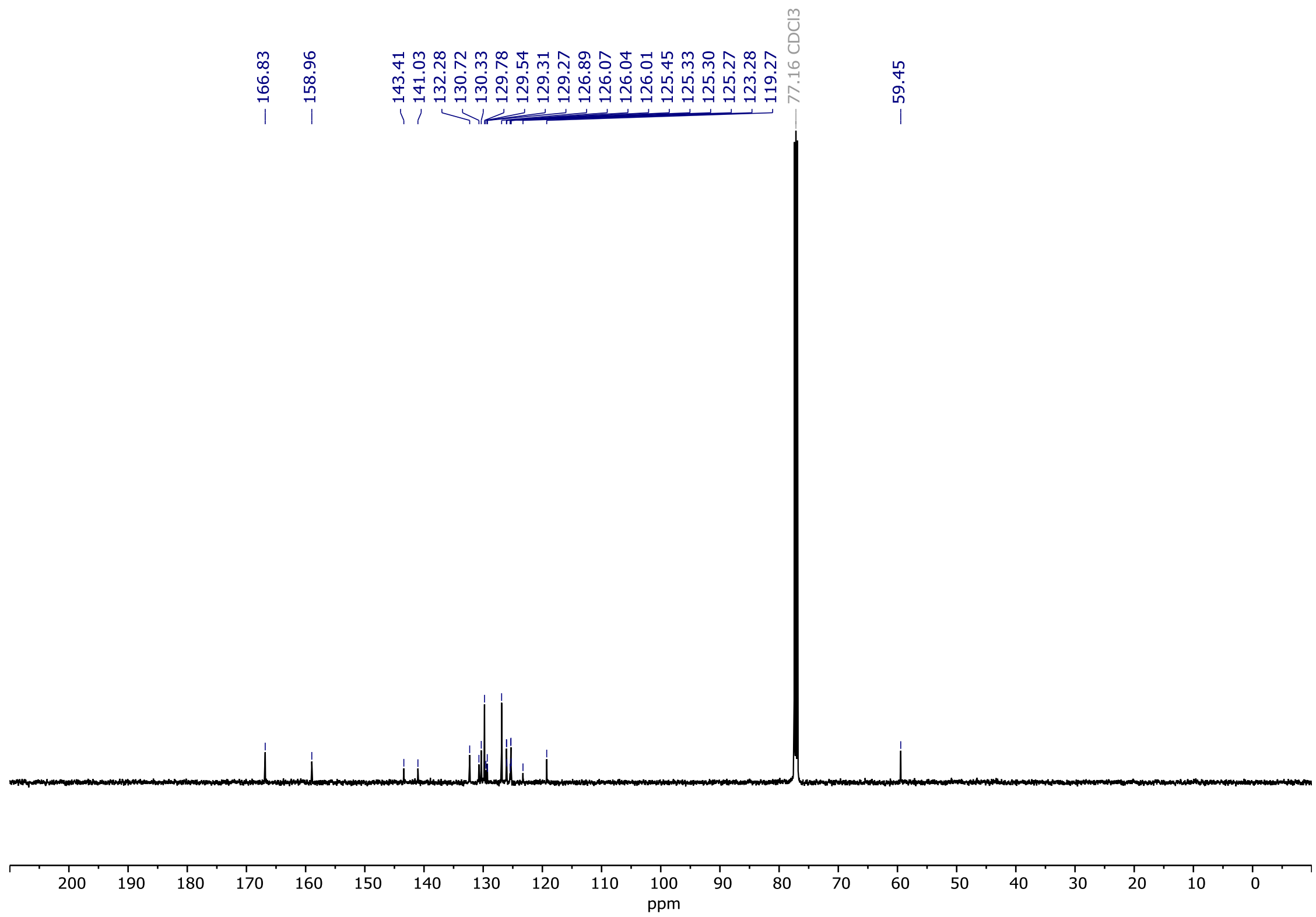

Figure S75. $^{13}C\{^1H\}$ NMR (126 MHz) spectrum of salen ligand **7'** in $CDCl_3$.

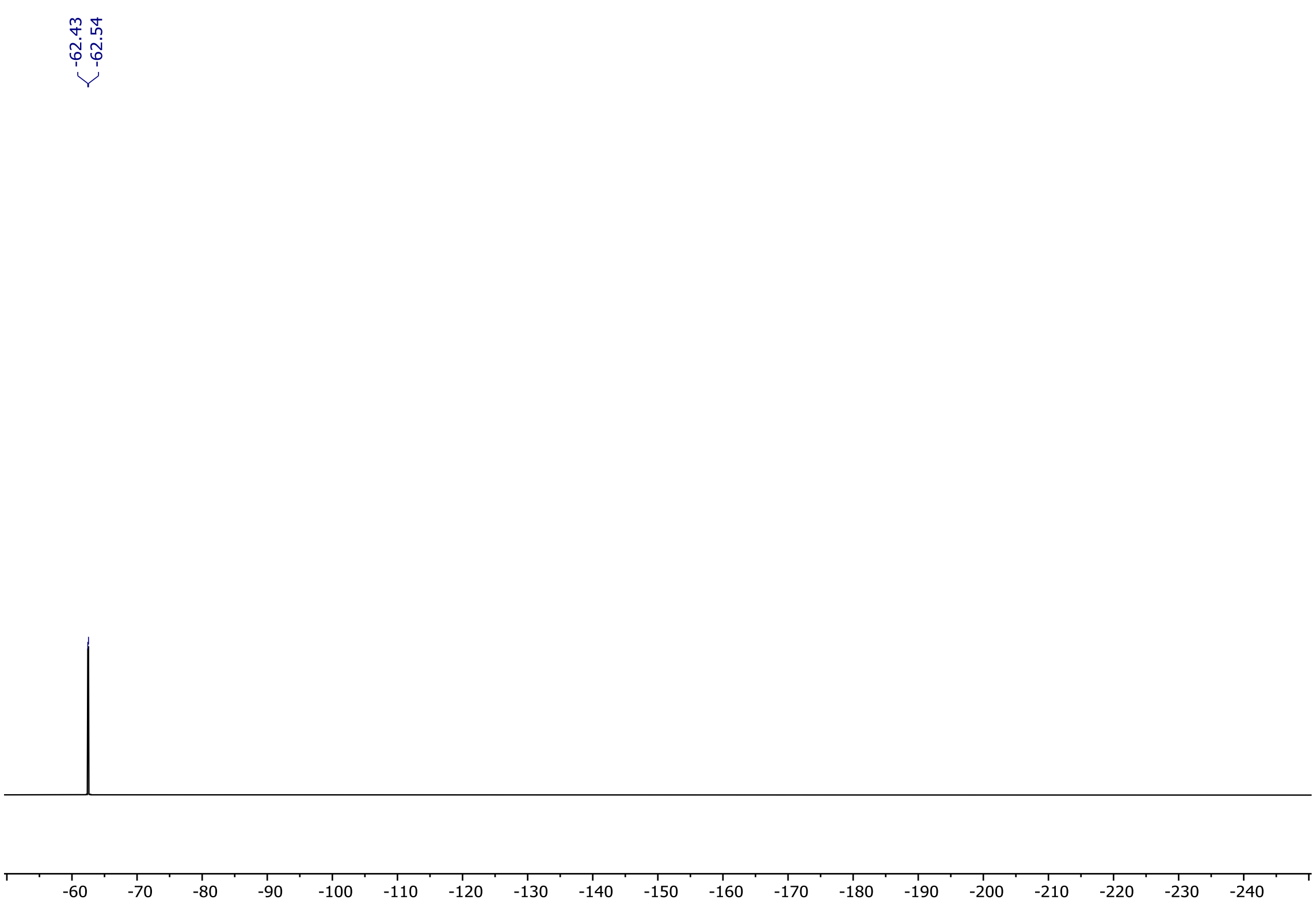

Figure S76. $^{19}F\{^1H\}$ NMR (470 MHz) spectrum of salen ligand **7'** in $CDCl_3$.

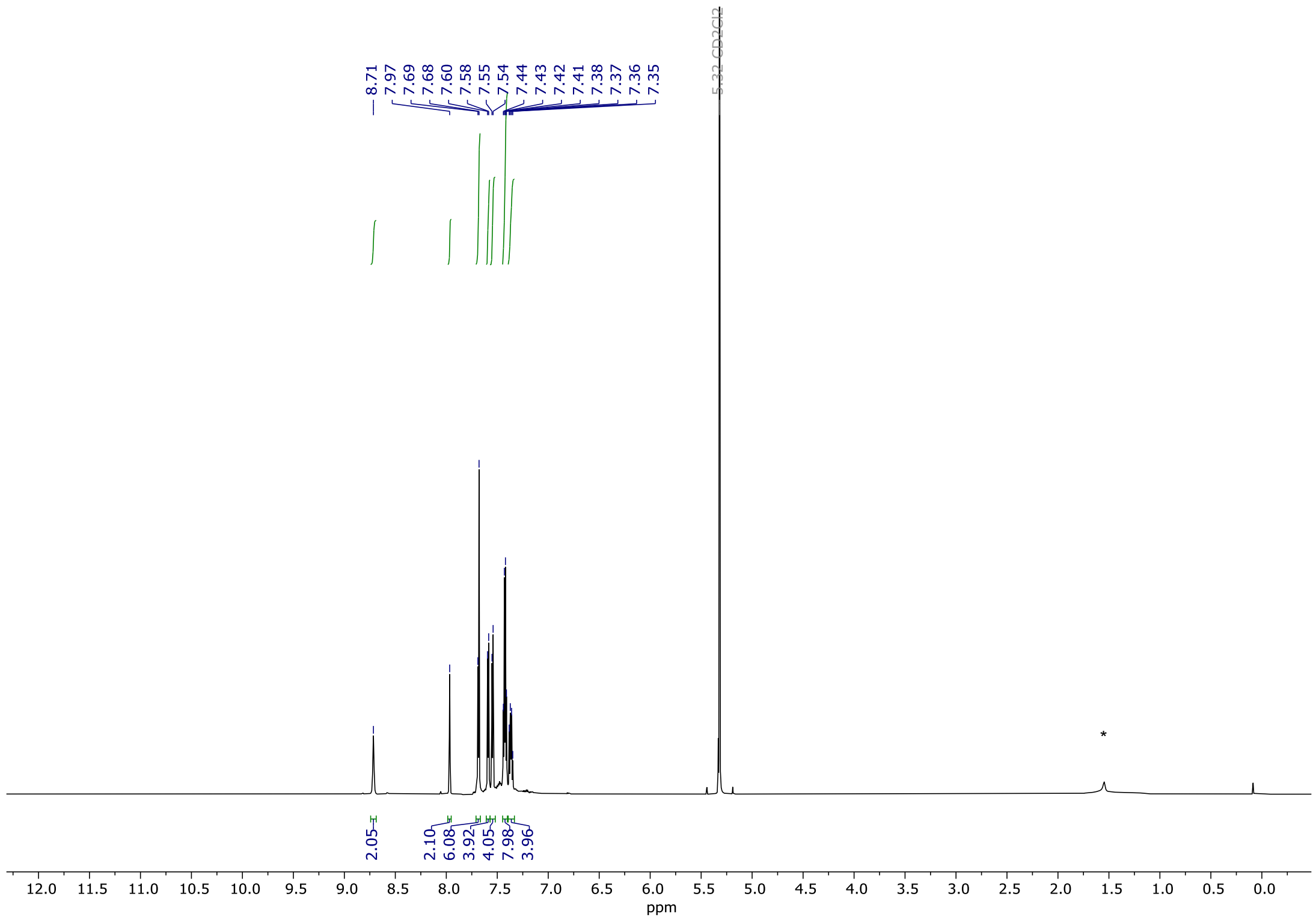

Figure S77. ^{1}H NMR (700 MHz) spectrum of boron complex **9** in CD_2Cl_2. The symbol (*) marks water.

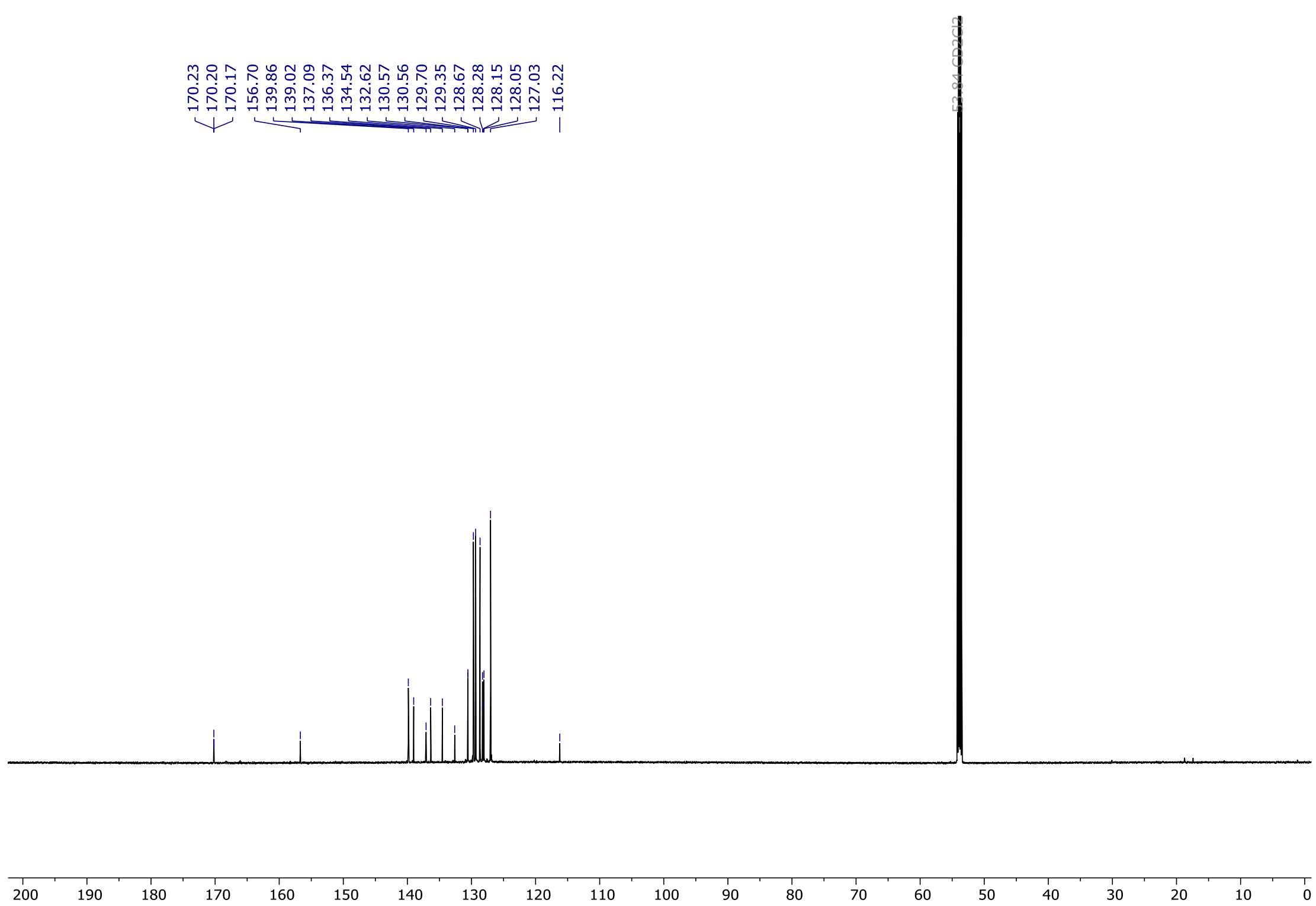

Figure S78. $^{13}C\{^{1}H\}$ NMR (176 MHz) spectrum of boron complex **9** in CD_2Cl_2.

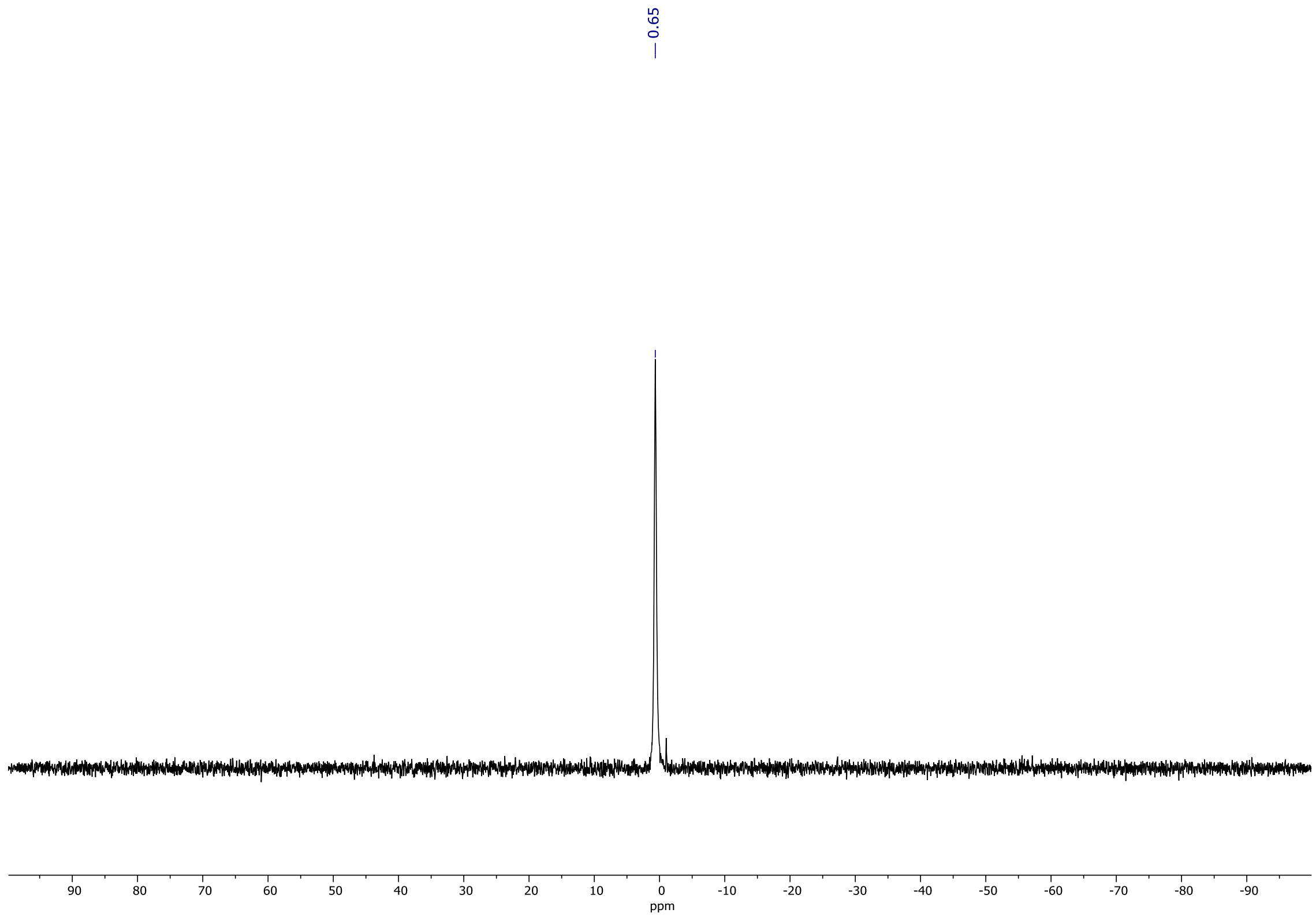

Figure S79. $^{11}B\{^1H\}$ NMR (160 MHz) spectrum of boron complex **9** in CD_2Cl_2.

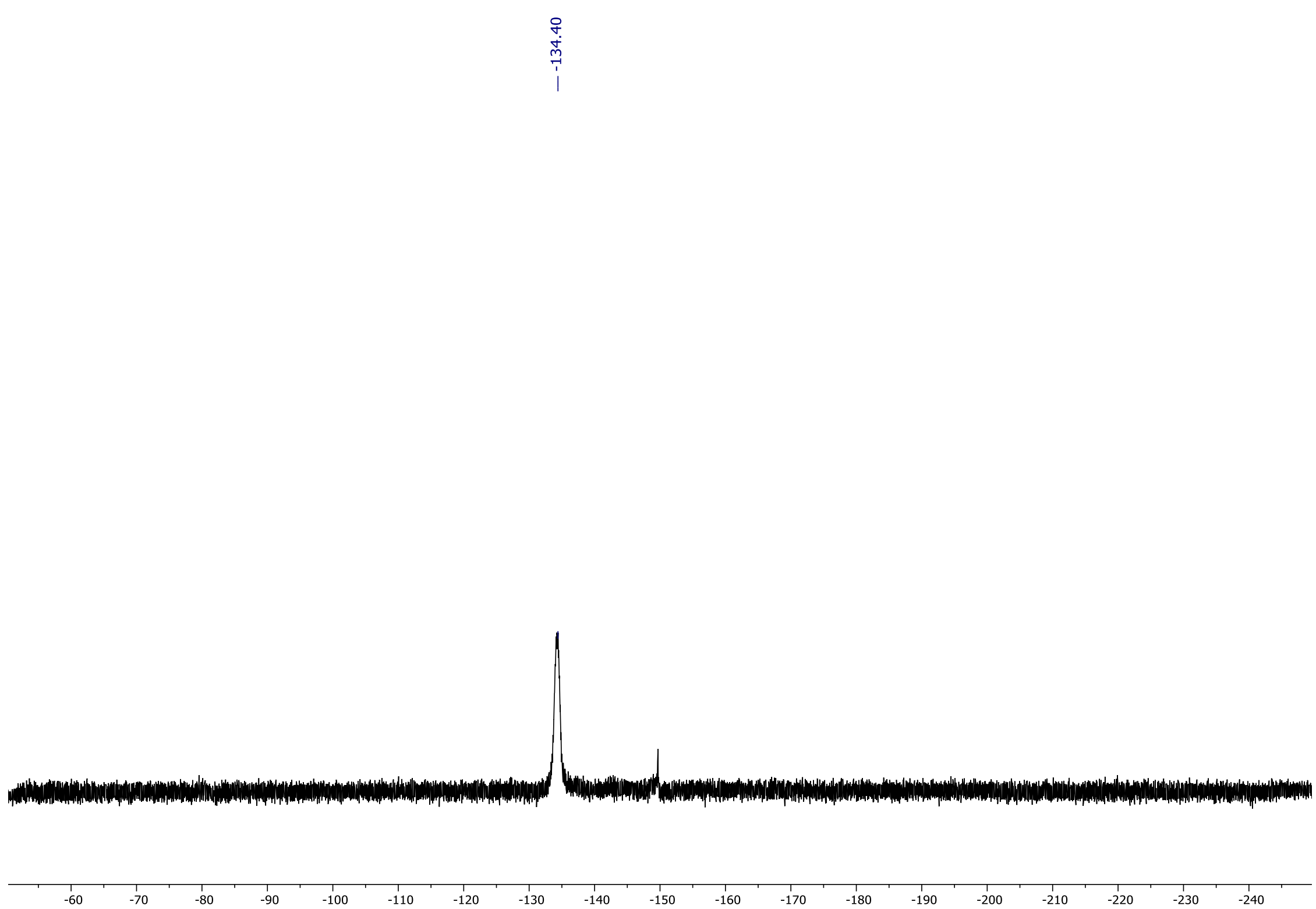

Figure S80. $^{19}F\{^1H\}$ NMR (470 MHz) spectrum of boron complex **9** in CD_2Cl_2.

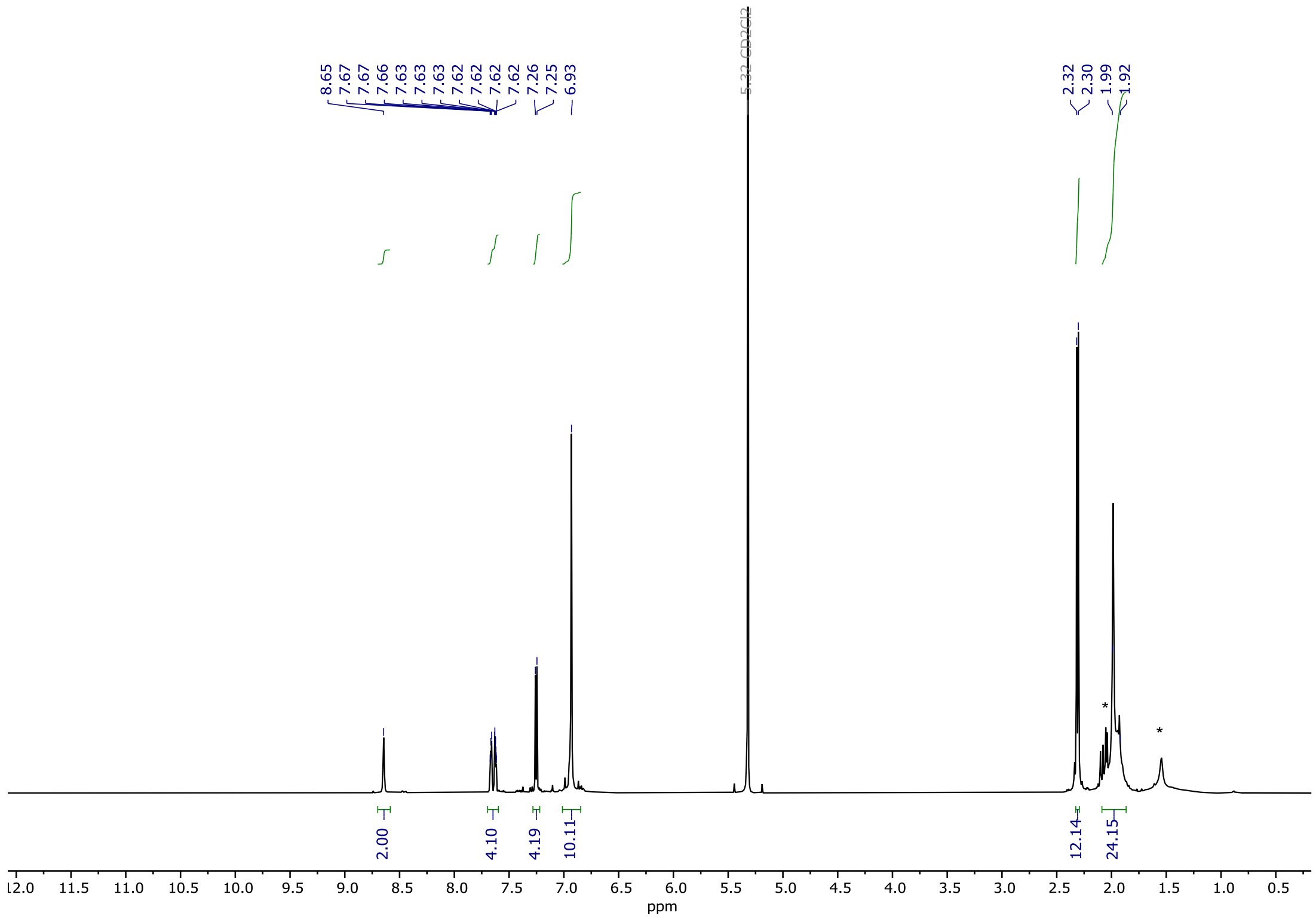

Figure S81. ^{1}H NMR (700 MHz) spectrum of boron complex **10** in CD_2Cl_2. The symbol (*) marks water and acetone.

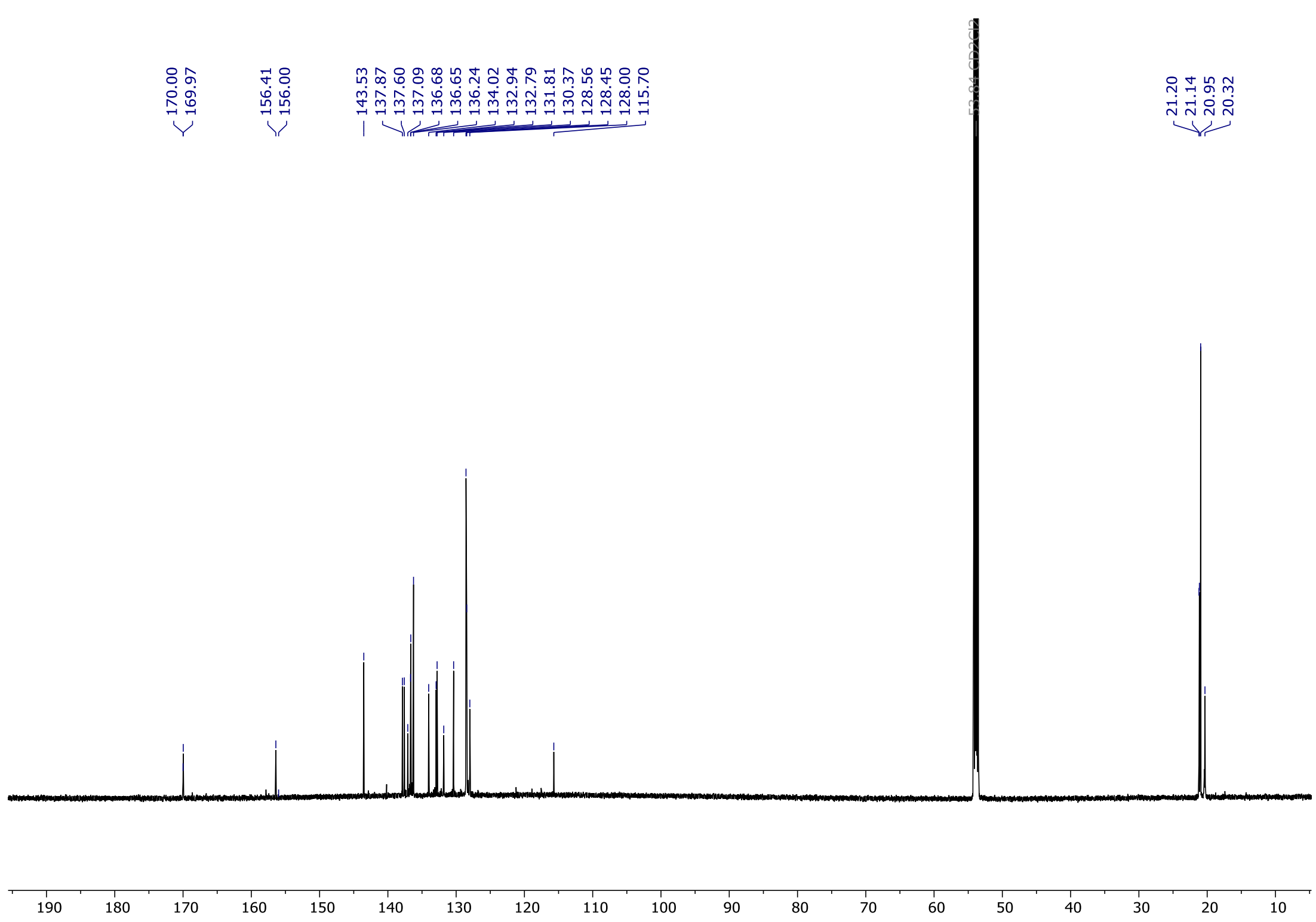

Figure S82. $^{13}C\{^1H\}$ NMR (176 MHz) spectrum of boron complex **10** in CD_2Cl_2.

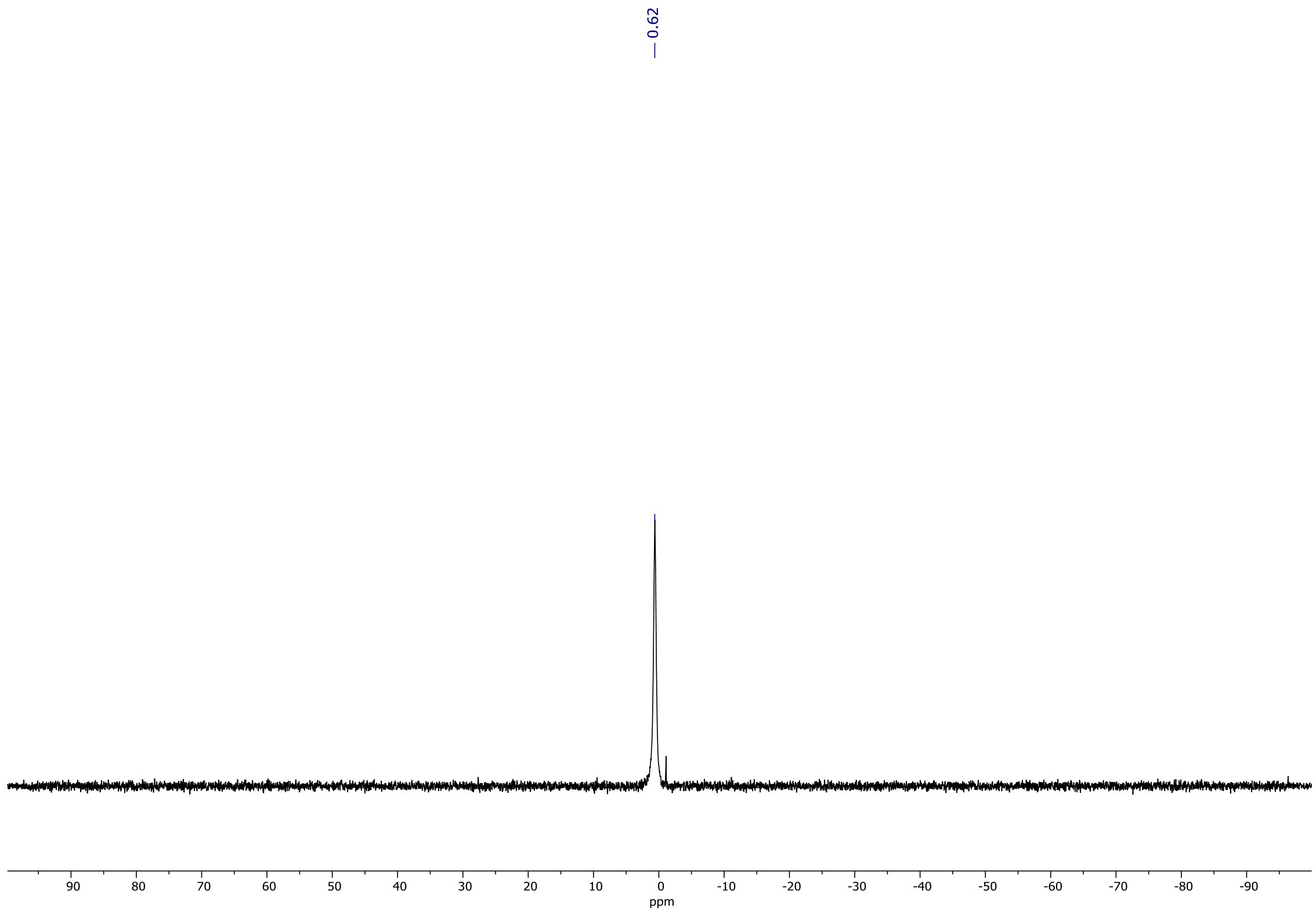

Figure S83. $^{11}B\{^1H\}$ NMR (160 MHz) spectrum of boron complex **10** in CD_2Cl_2.

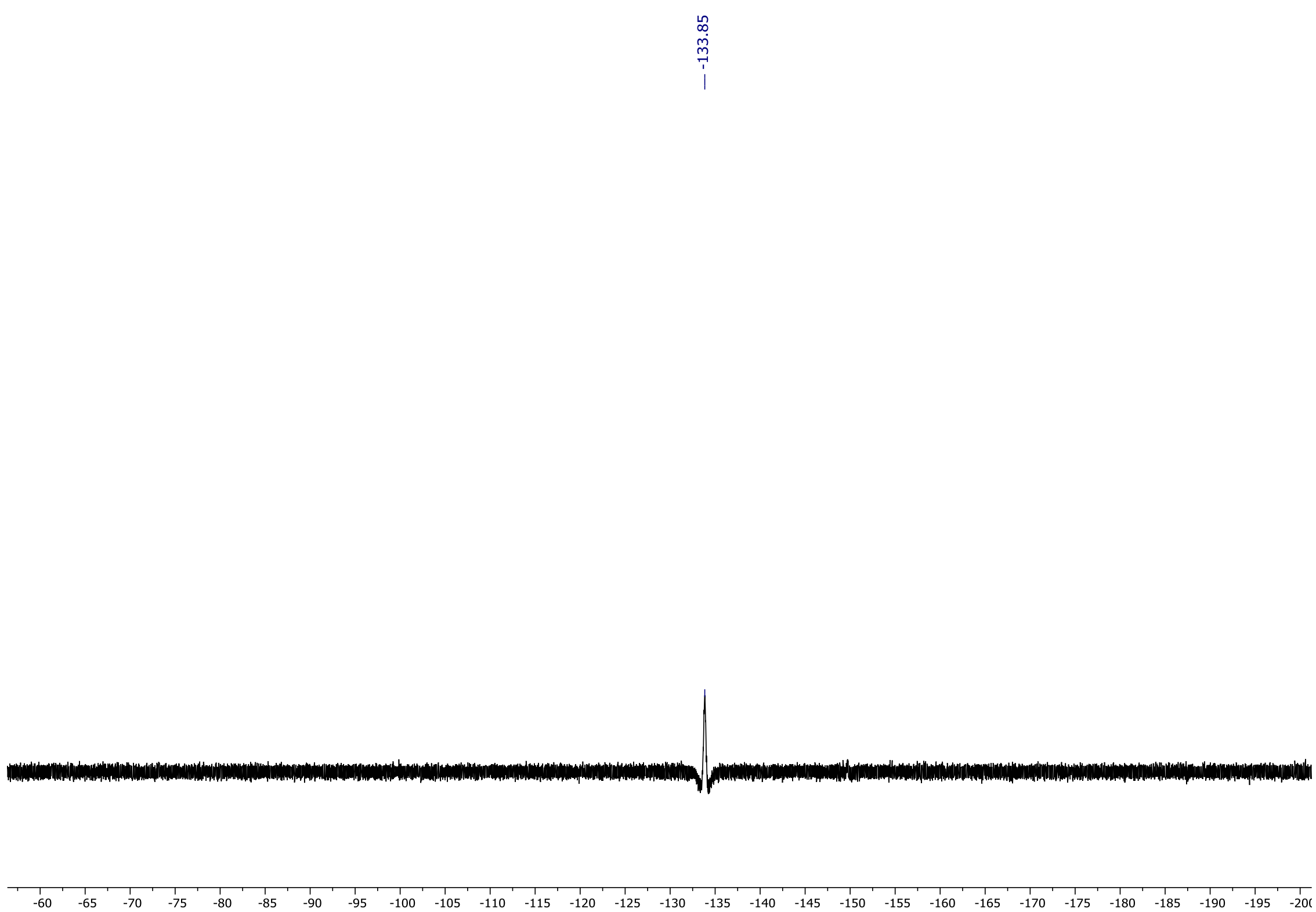

Figure S84. $^{19}F\{^1H\}$ NMR (470 MHz) spectrum of boron complex **10** in CD_2Cl_2.

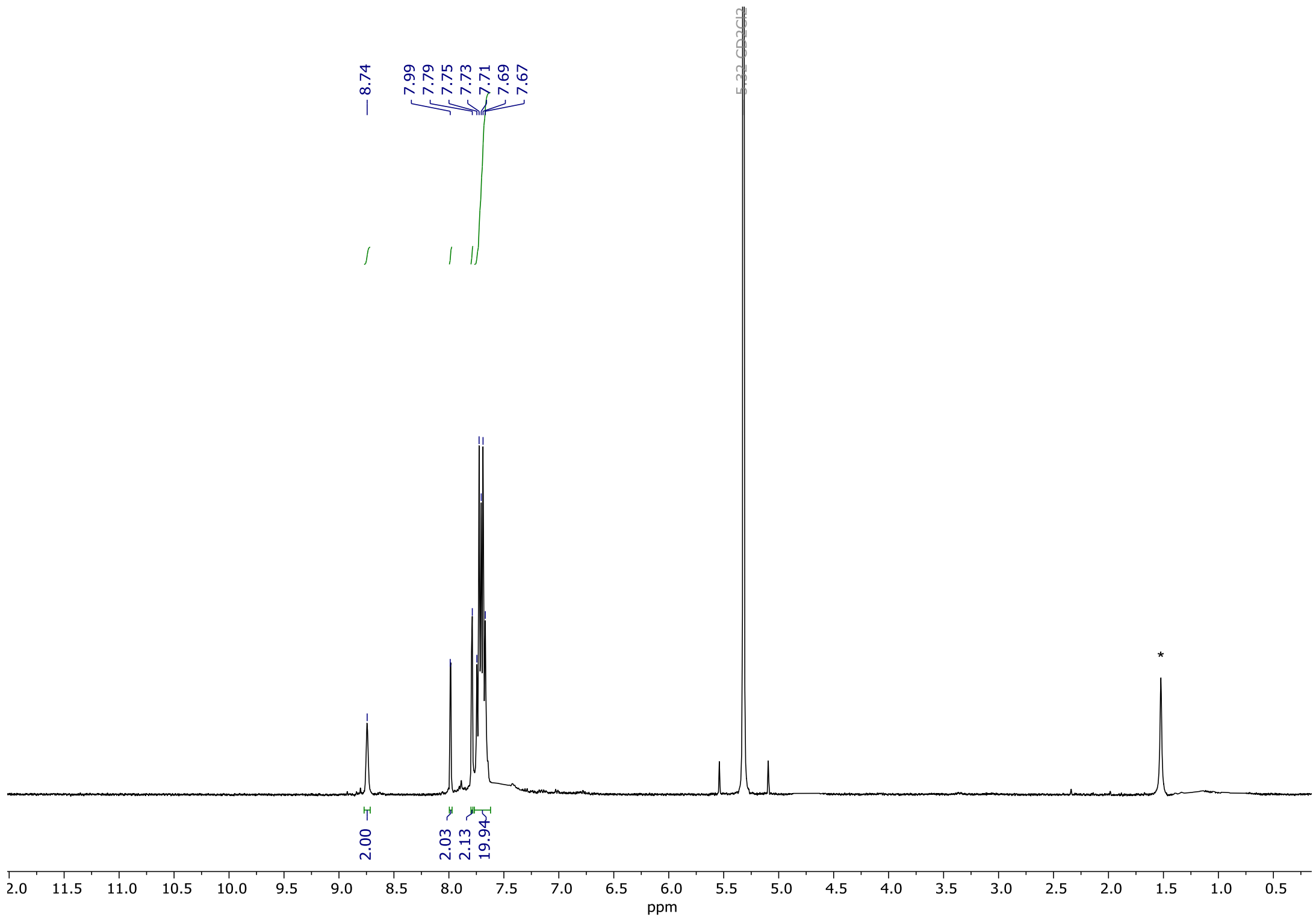

Figure S85. ^{1}H NMR (400 MHz) spectrum of boron complex **11** in CD_2Cl_2. The symbol (*) marks water.

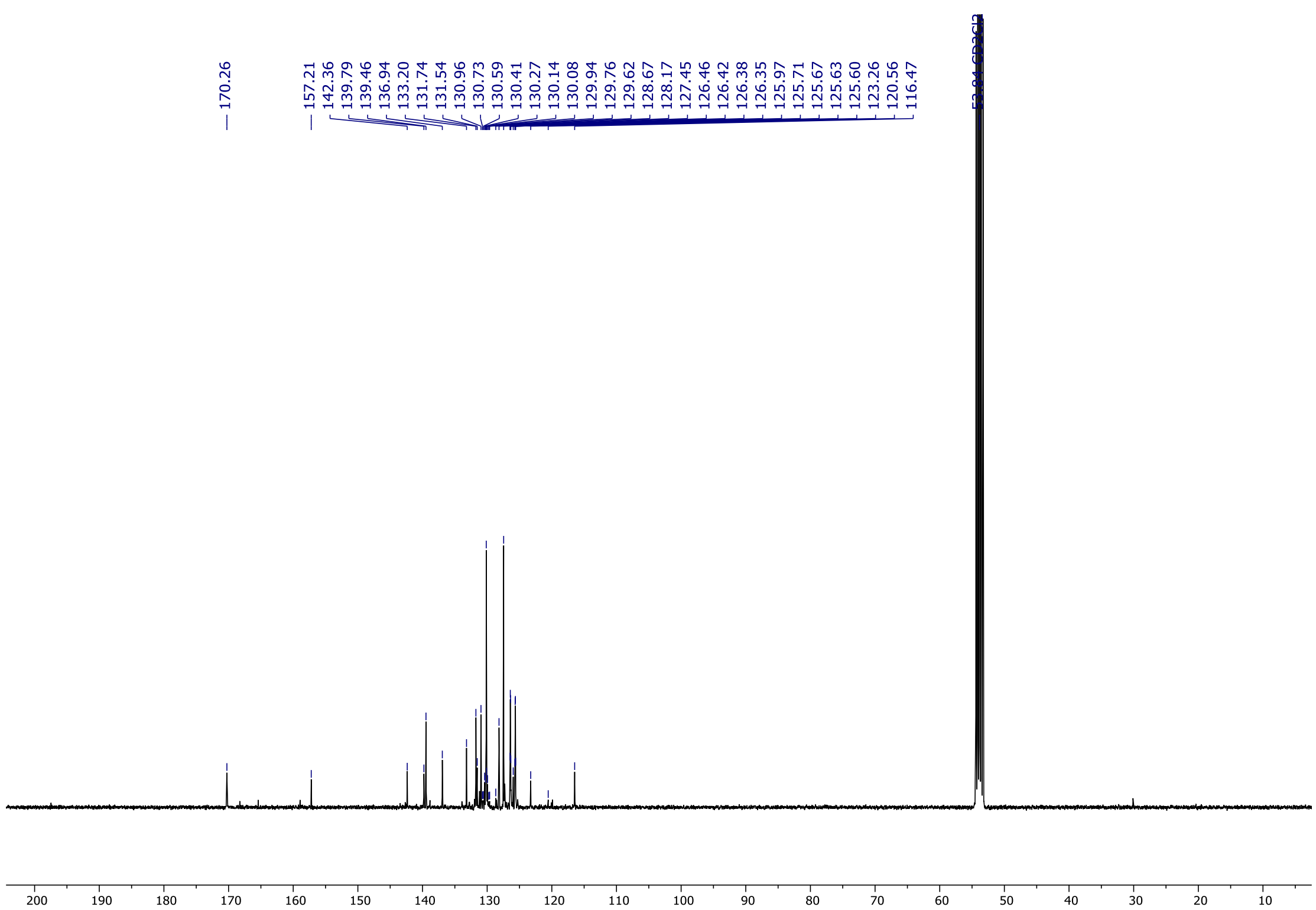

Figure S86. $^{13}C\{^{1}H\}$ NMR (101 MHz) spectrum of boron complex **11** in CD_2Cl_2.

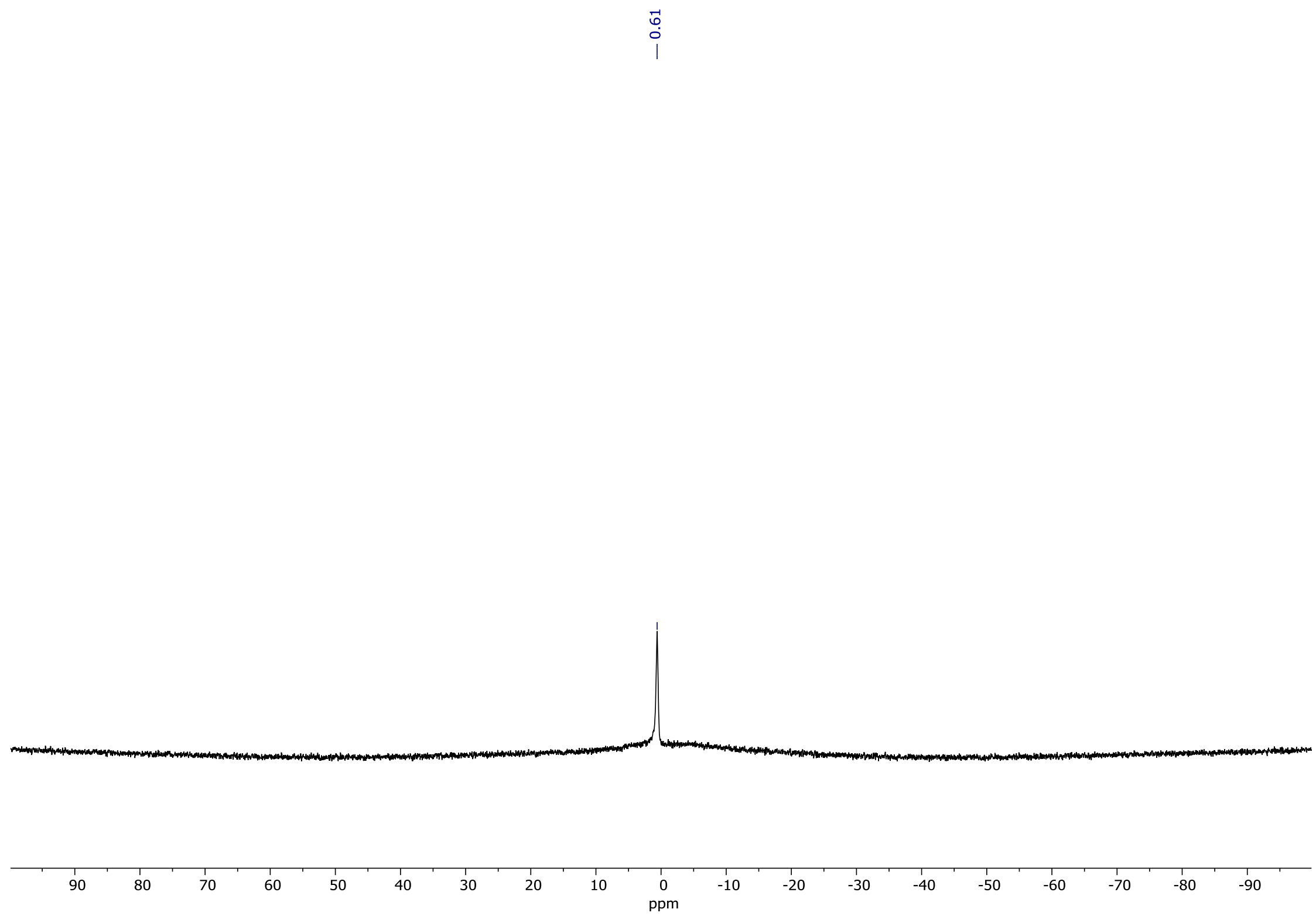

Figure S87. $^{11}B\{^{1}H\}$ NMR (128 MHz) spectrum of boron complex **11** in CD_2Cl_2.

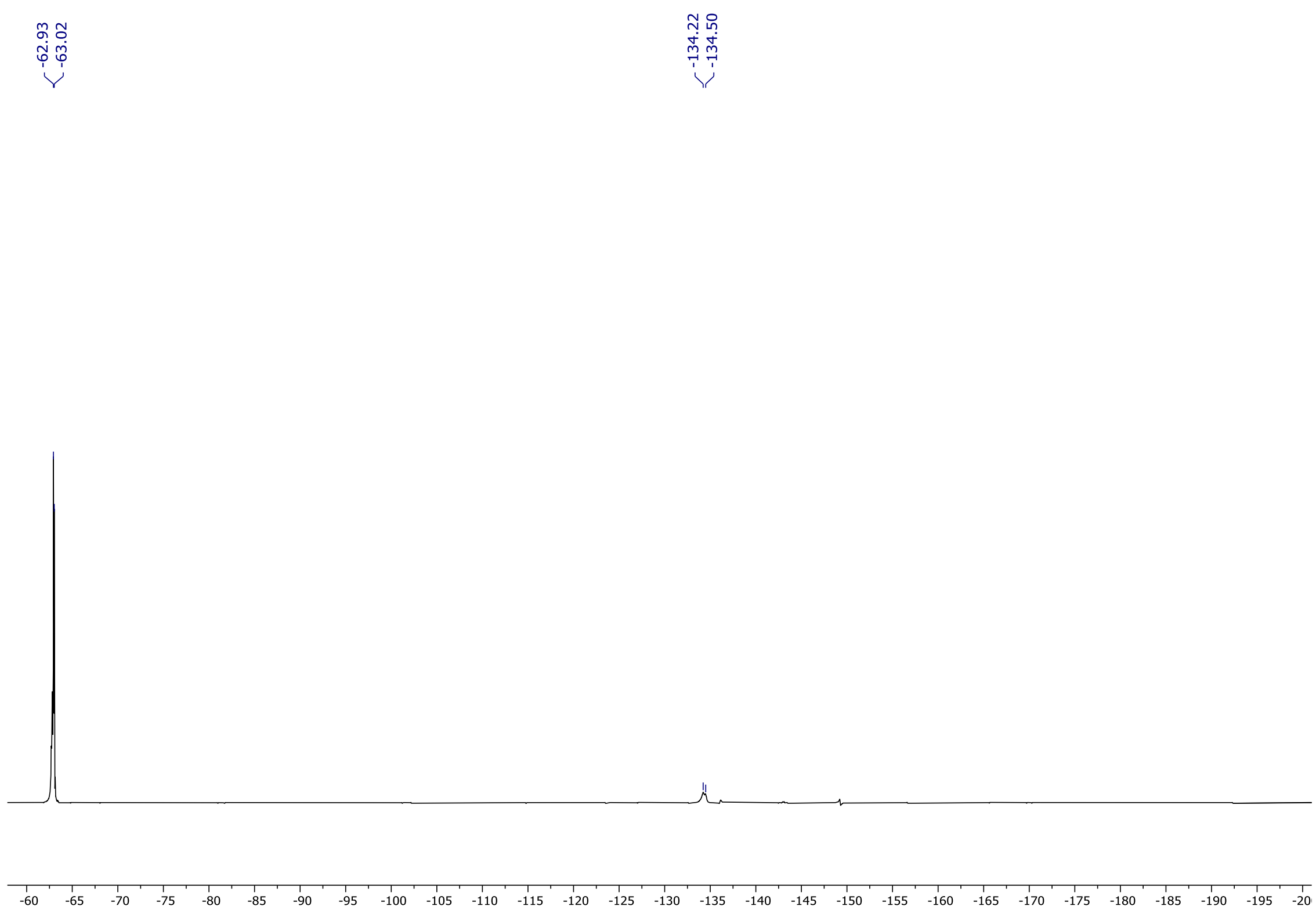

Figure S88. $^{19}F\{^{1}H\}$ NMR (470 MHz) spectrum of boron complex **11** in CD_2Cl_2.

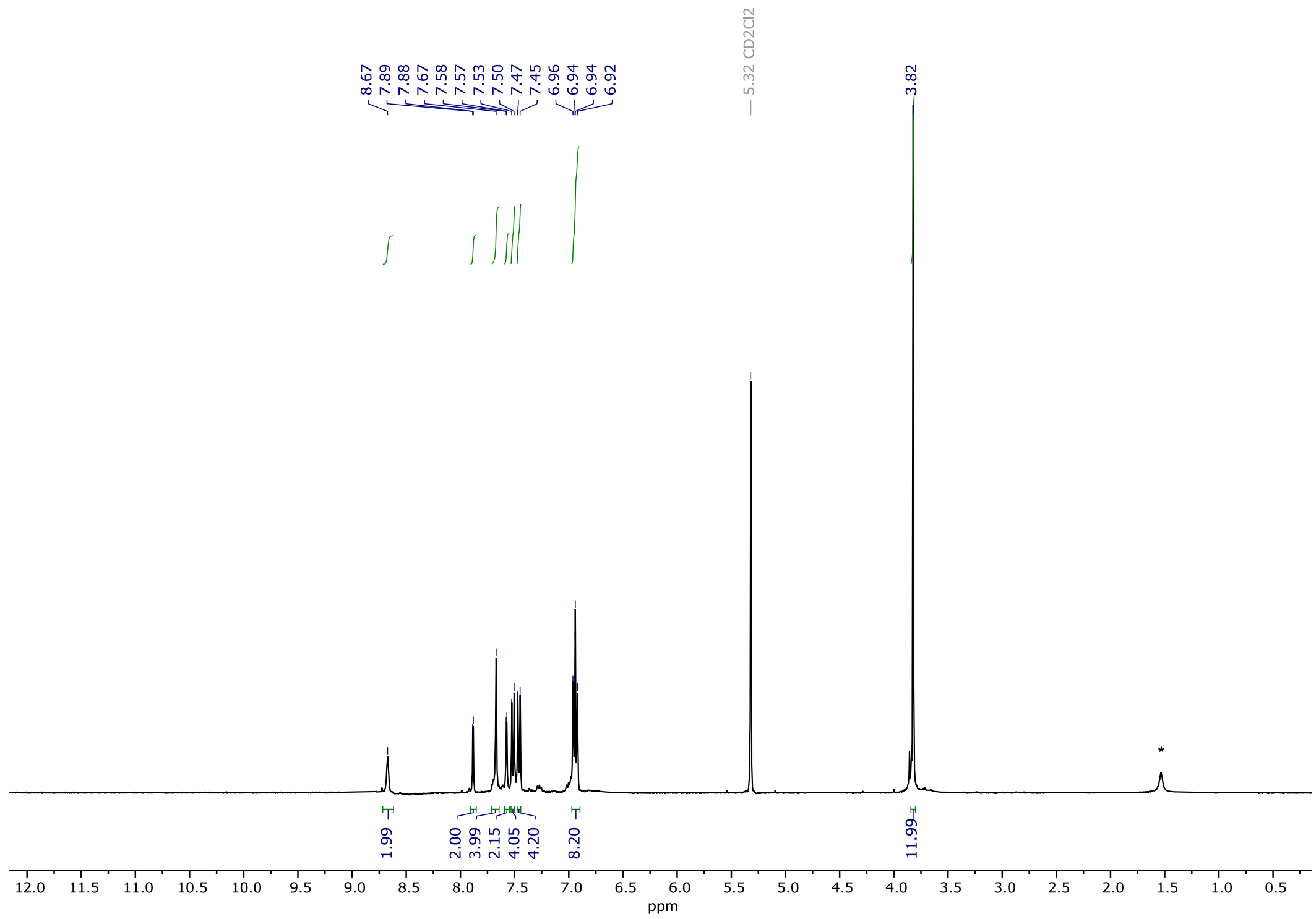

Figure S89. ^{1}H NMR (400 MHz) spectrum of boron complex **12** in CD_2Cl_2. The symbol (*) marks water.

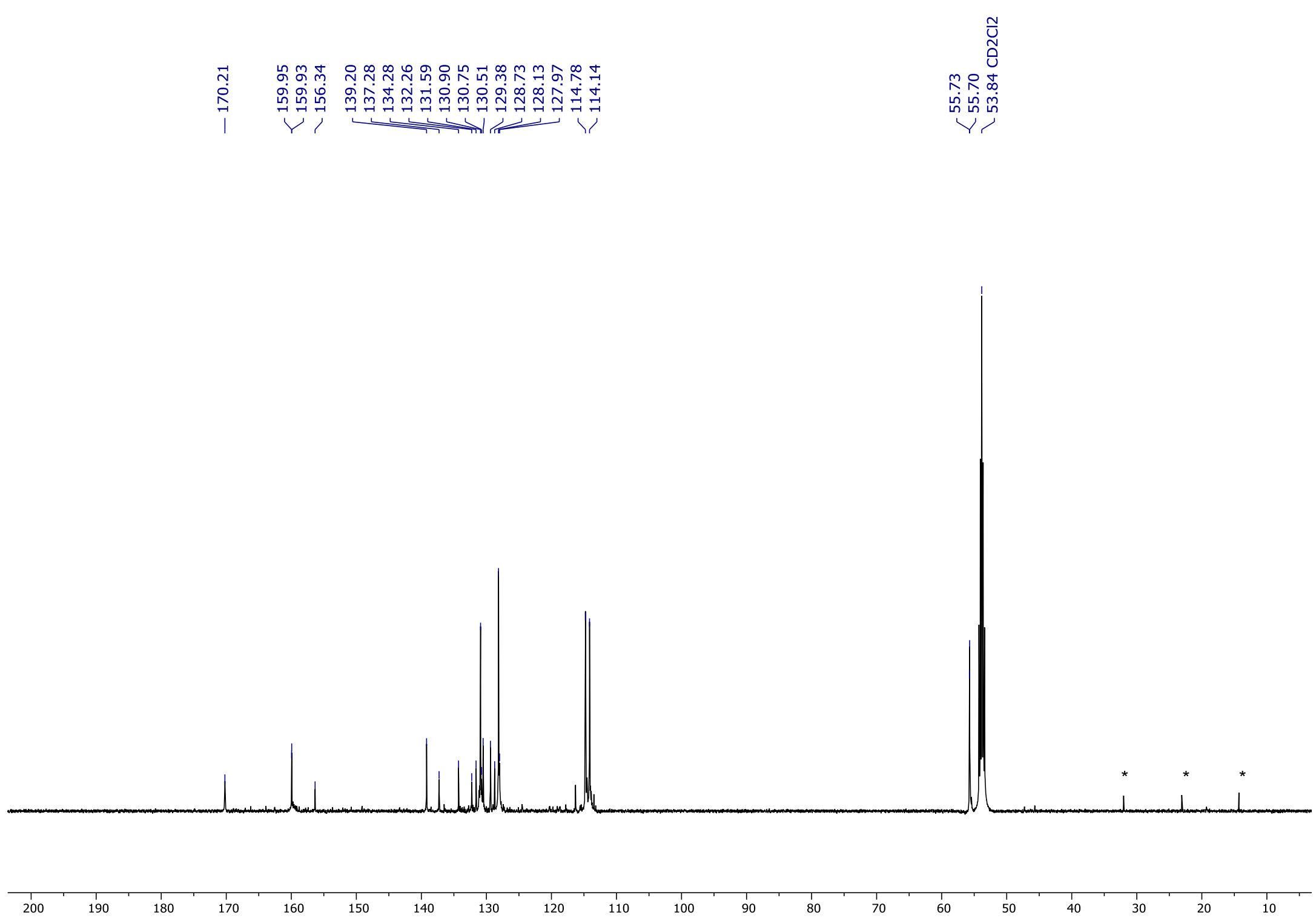

Figure S90. $^{13}C\{^1H\}$ NMR (126 MHz) spectrum of boron complex **12** in CD_2Cl_2. The symbol (*) marks *n*-hexane.

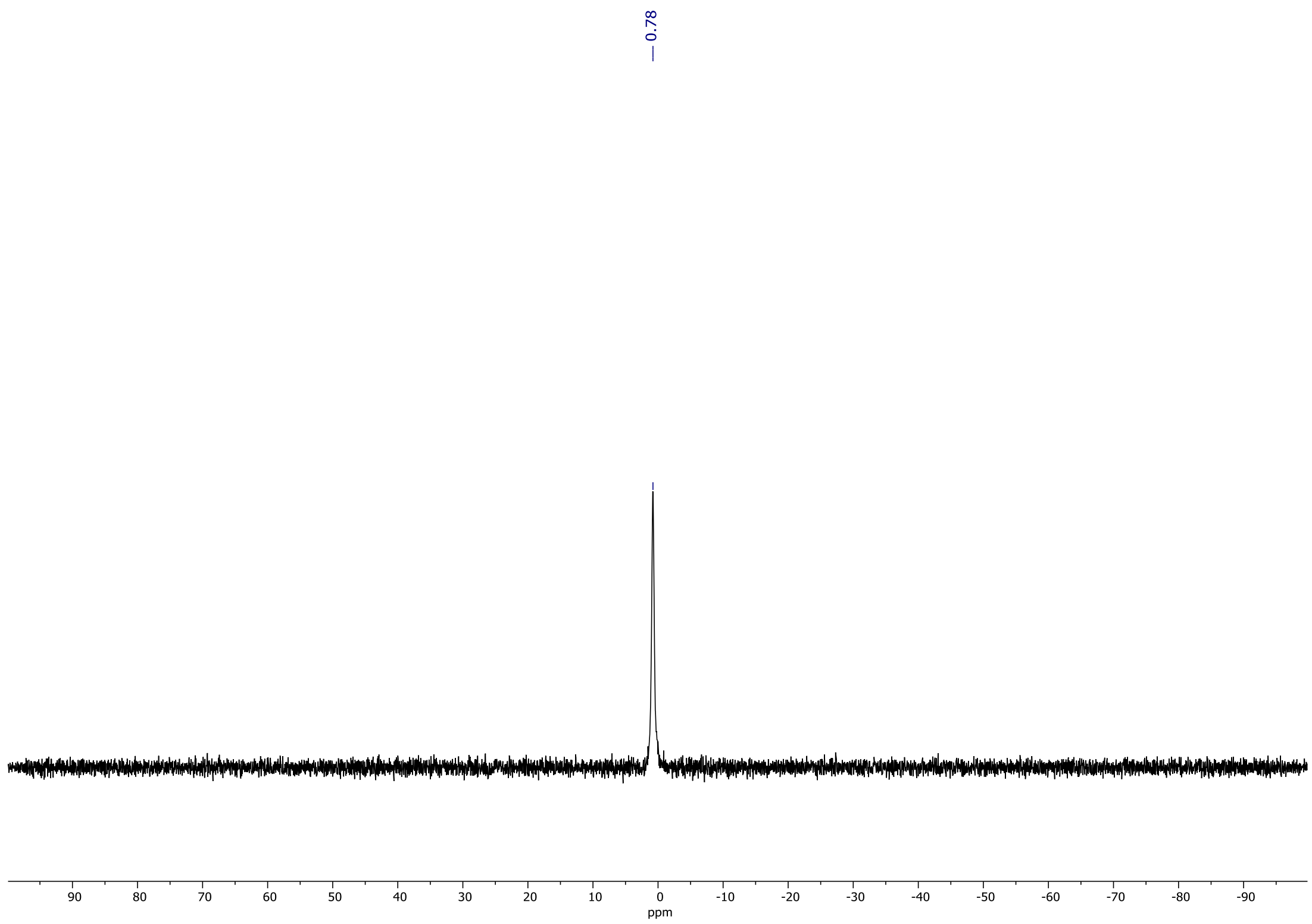

Figure S91. $^{11}B\{^1H\}$ NMR (128 MHz) spectrum of boron complex **12** in CD_2Cl_2.

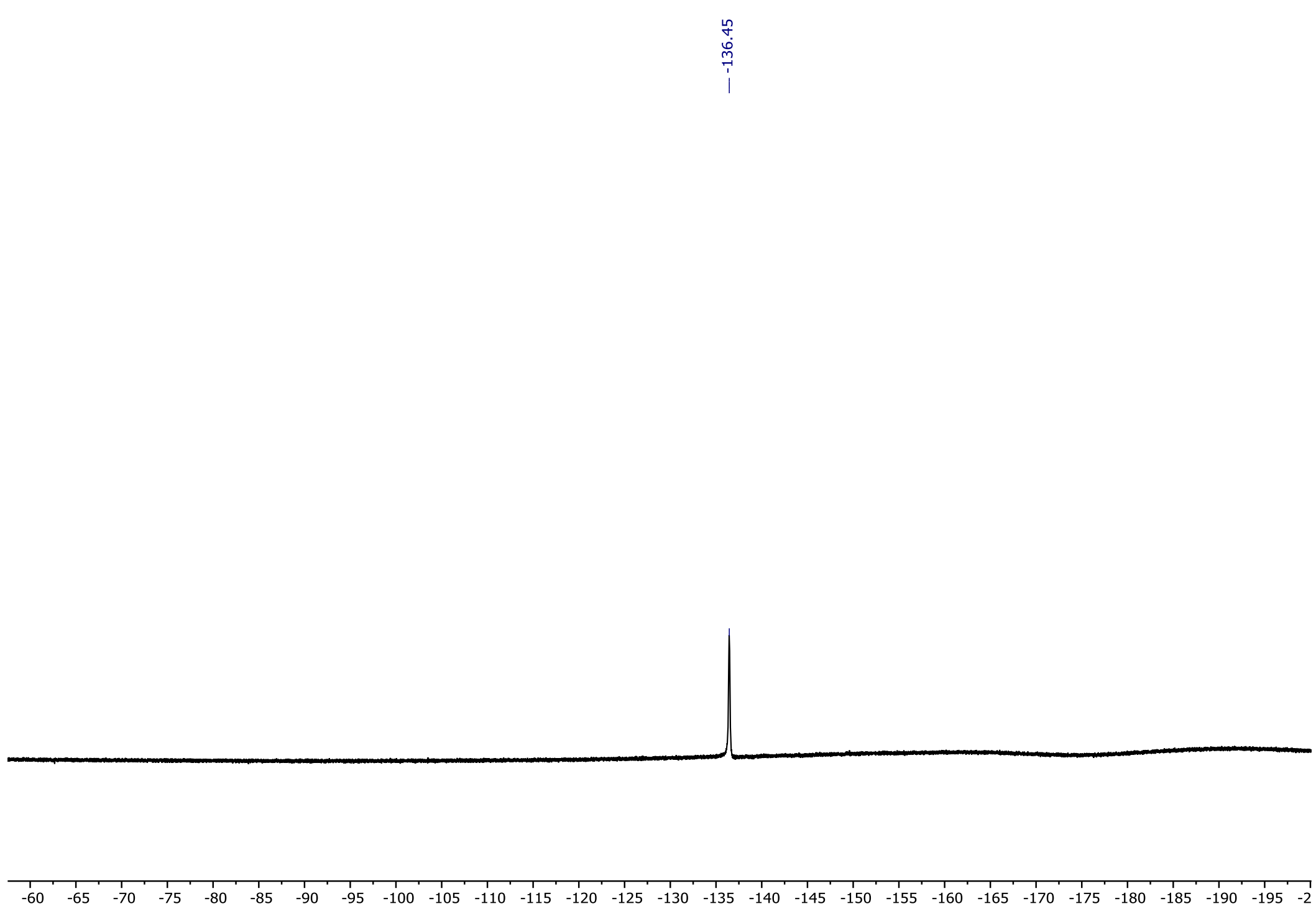

Figure S92. $^{19}F\{^1H\}$ NMR (470 MHz) spectrum of boron complex **12** in CD_2Cl_2.

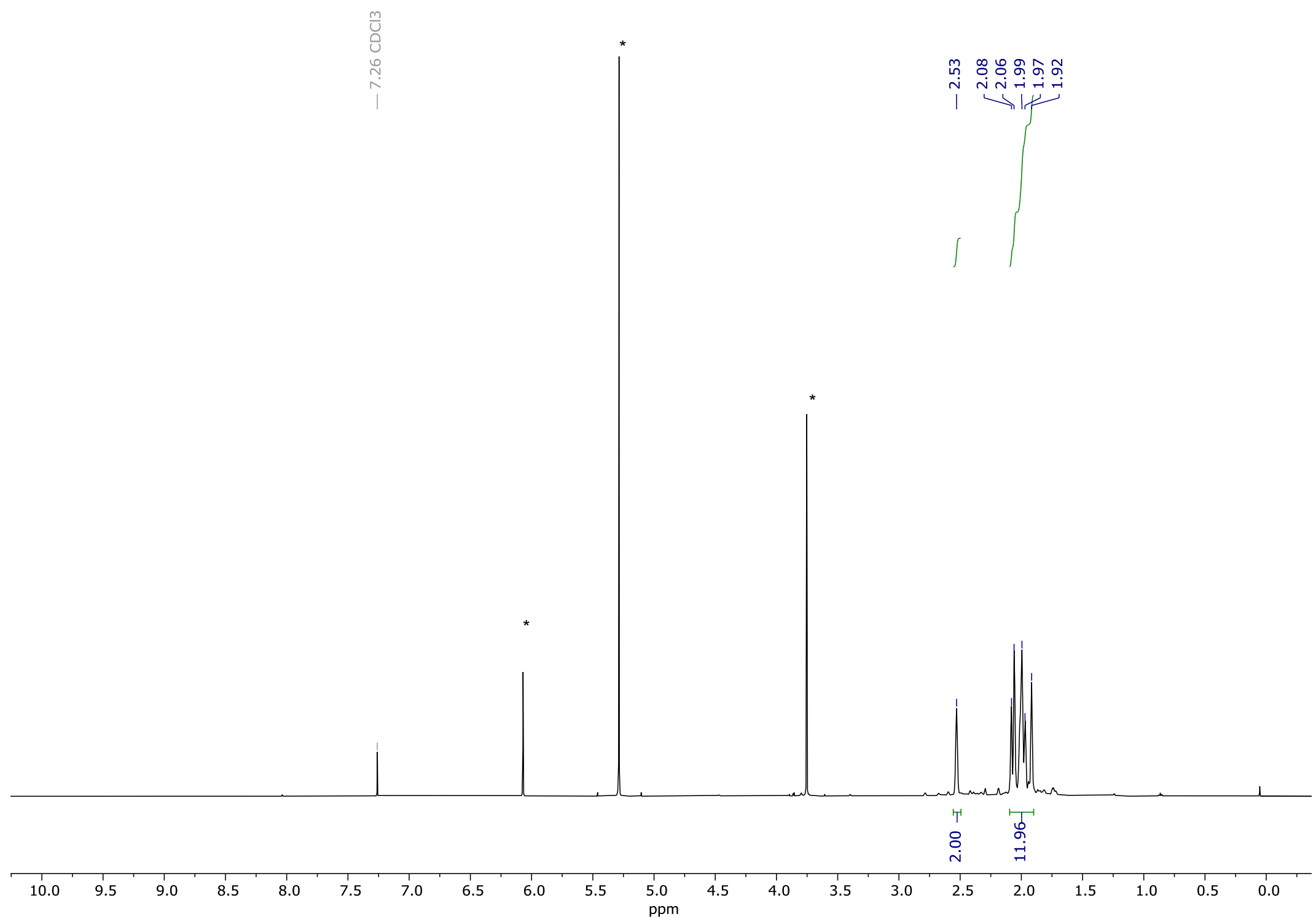

Figure S93. ^1H NMR (500 MHz) spectrum of crude compound **15** (after reaction without purification) in CD_3Cl. The symbol (*) marks DCM and the internal standard (1,3,5-trimethoxybenzene).

9. Single-crystal X-ray diffraction analysis

Crystals were obtained by saturated solutions of each compound in CH_2Cl_2 by slow diffusion of a specific non-solvent at room temperature. Details are listed in **Table S13**:

Table S13. Crystallization details.

Compound #	Abbreviation	Non-solvent
**7**	**Salphen-CF$_3$**	*n*-Hexane
**7'**	**Salen-CF$_3$**	Isopropanol
**9**	**Boron-salphen-Ph**	*n*-Dodecane

A clear orange plank of **7** (0.23 x 0.08 x 0.05 mm^3, a clear yellow needle of **7‘** (0.30 x 0.10 x 0.05 mm^3), a clear yellow plate of **9** (0.22 x 0.16 x 0.05 mm) were mounted on a STOE STADIVARI Eulerian 4-circle diffractometer equipped with a low-temperature device (100(2)K, Oxford Cryostream 800er series, Oxford Cryosystems) by using Cu-*K*α-radiation (λ = 1.54186 Å, ASTIX++-optics) and a PILATUS 200K Pixel detector system. Intensities were measured by fine-slicing φ- and ω-scans and corrected for background, polarization and Lorentz effects. A semi-empirical absorption correction by scaling of reflection intensities with a subsequent spherical absorption correction was performed with LANA.[77]

Structure solution was done using intrinsic phasing methods included in the SHELXT program system[78] and refined by full matrix least-squares/difference Fourier synthesis with ShelXL-2019/3).[79] All non-hydrogen atoms were refined anisotropically; hydrogen atoms were placed in geometrically calculated positions and included using a riding model

on the bound carbon atoms and relative isotropic displacement parameters. Olex2 was used for graphical representations of the molecular structures.[80]

CCDC numbers CCDC-2541422 (**7**), CCDC-2541423 (**7’**), and CCDC-2541424 (**9**) contain the supplementary crystallographic data for this paper, which can be obtained free of charge from the Cambridge Crystallographic Data Centre via http://www.ccdc.cam.ac.uk/data request/cif.

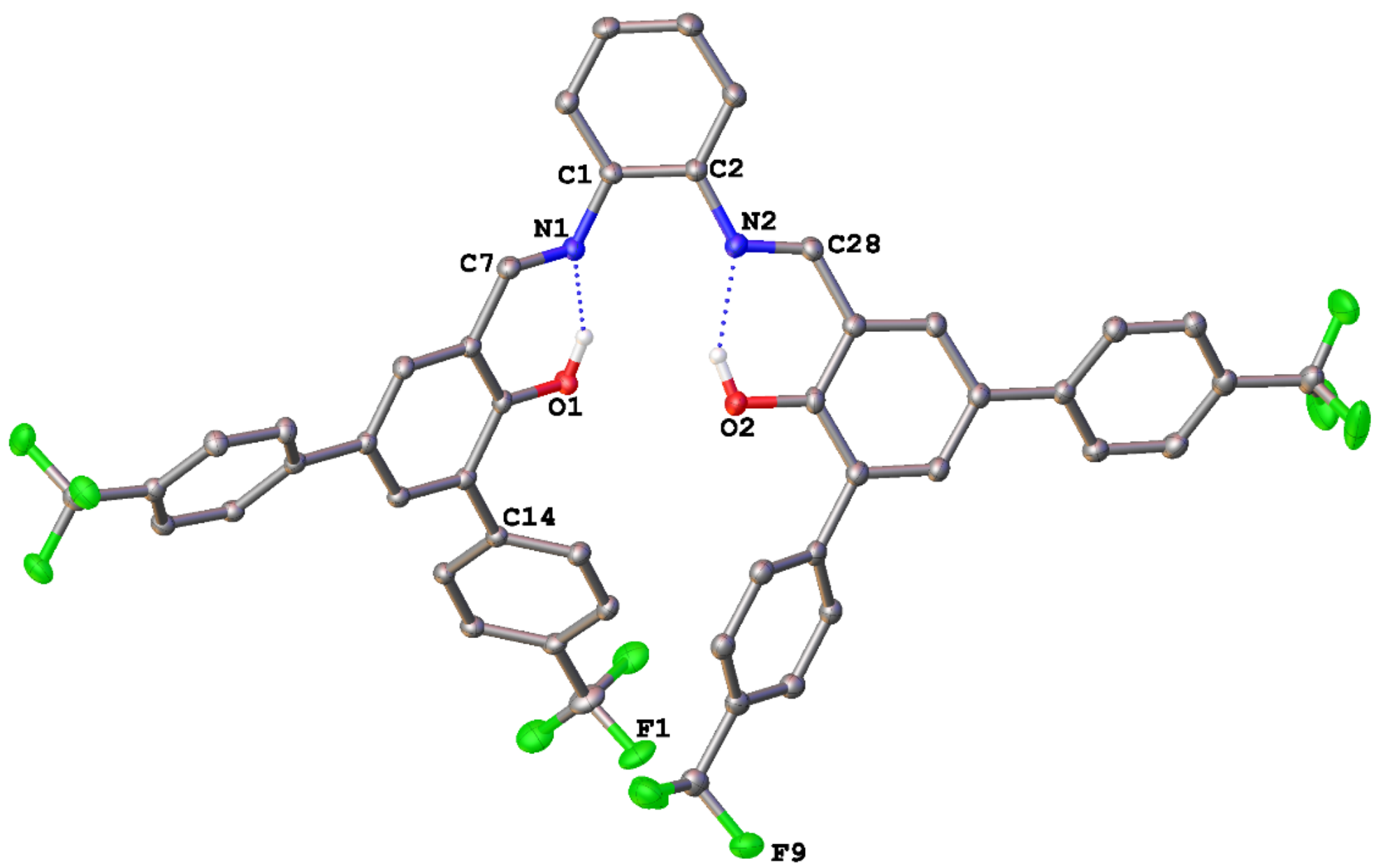

Figure S94. Solid state structure of **7**. Hydrogen atoms and the site occupancies of a triply rotationally disordered CF_3-group are omitted for clarity. Thermal ellipsoids are set to 50% probability level.

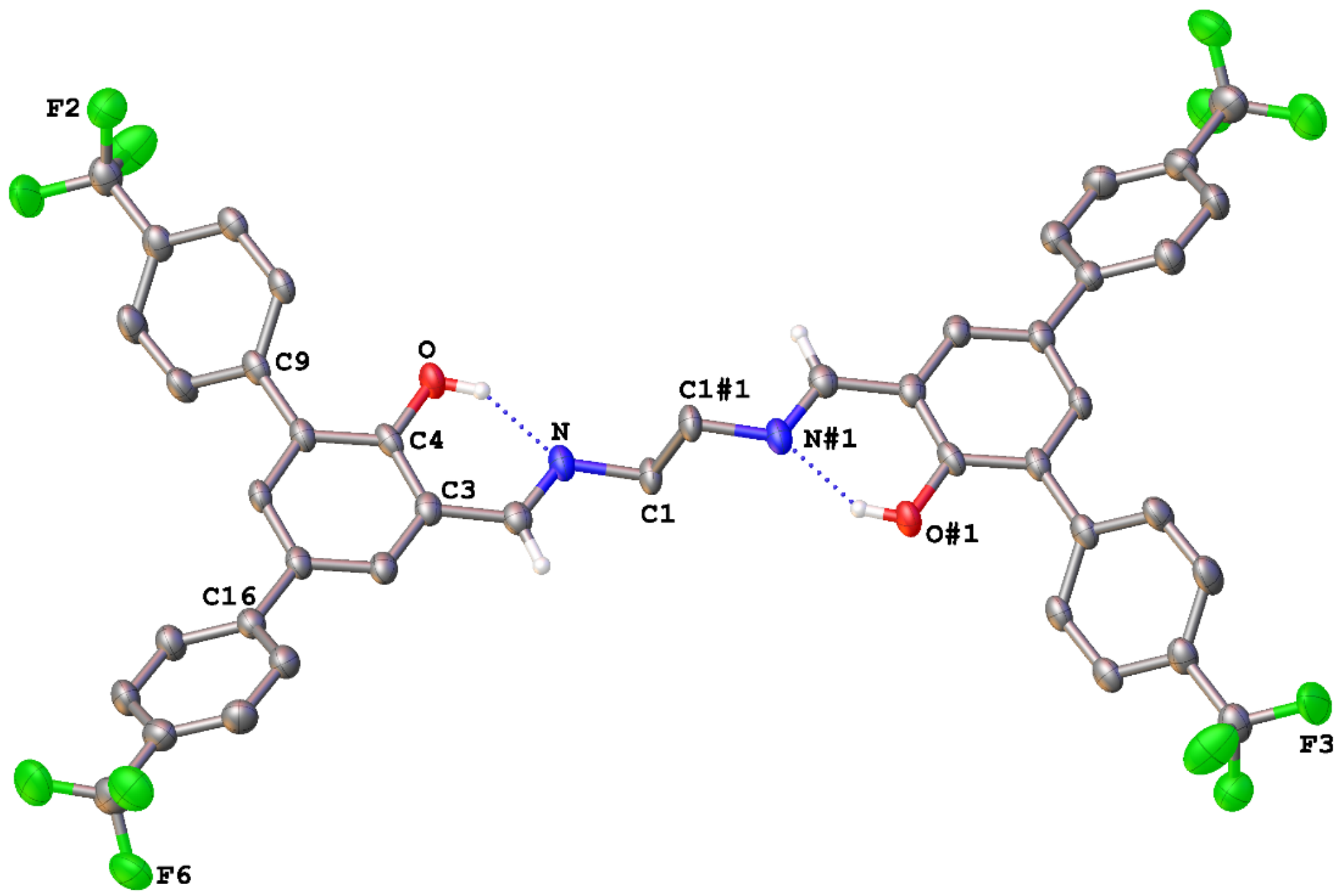

Figure S95. Solid state structure of **7'**. Hydrogen atoms and the site occupancies of a rotationally disordered CF_3-group are omitted for clarity. The molecule resides on a crystallographic inversion center lying on the midpoint of the C1···C1# bond. Thermal ellipsoids are set to 50% probability level.

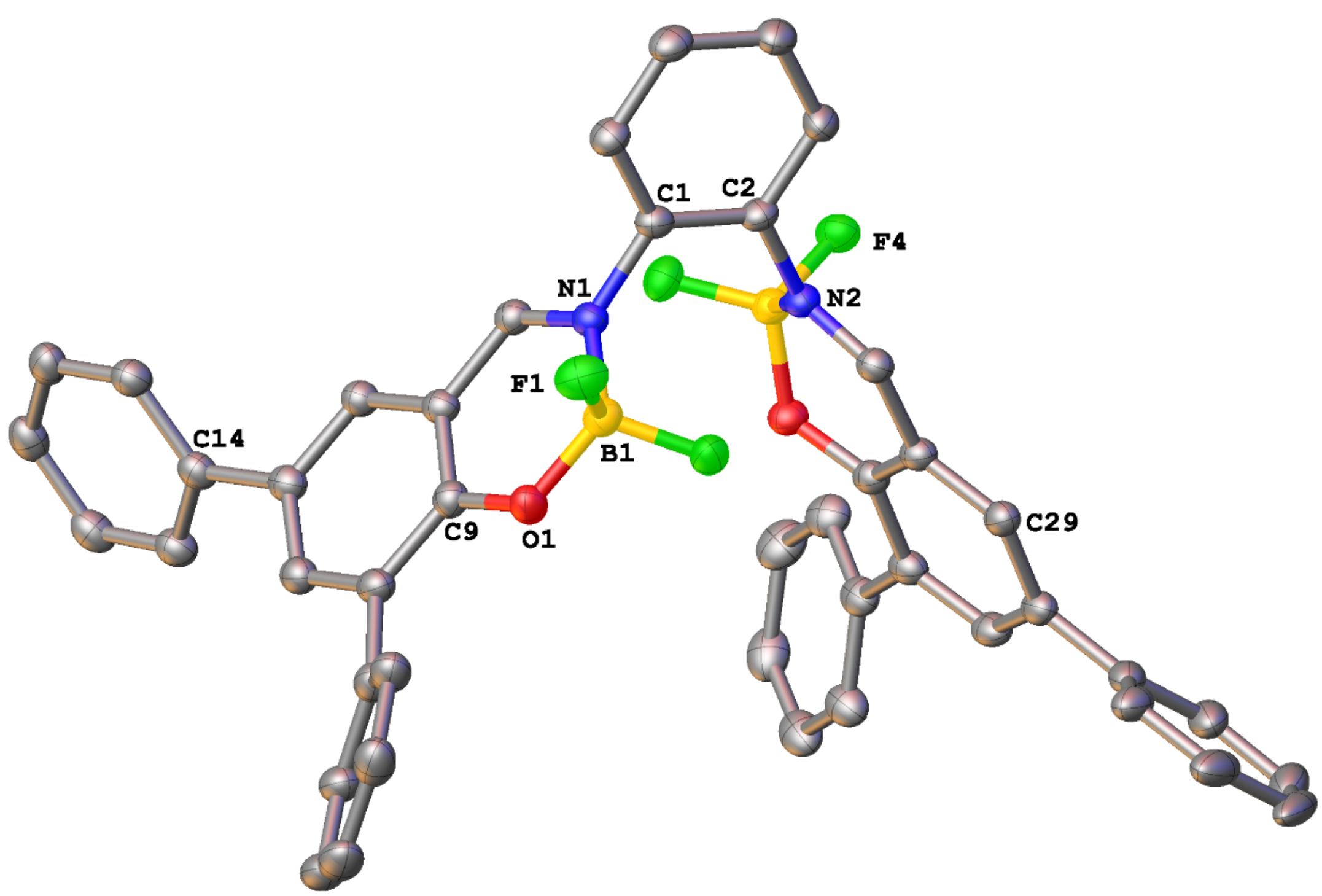

Figure S96. Solid state structure of **9**. Hydrogen atoms are omitted for clarity. Thermal ellipsoids are set to 50% probability level.

10. References

[58] J. M. Fraile, N. García, C. I. Herrerías, J. A. Mayoral, “Integration of heterogeneous catalysts into complex synthetic routes: sequential vs. one-pot reactions in a (Knoevenagel + Mukaiyama–Michael + hydrogenation + transesterification) sequence” *Catal. Sci. Technol.* **2013**, *3*, 436–443.

[59] T. Senthamarai, V. G. Chandrashekhar, M. B. Gawande, N. V. Kalevaru, R. Zbořil, P. C. J. Kamer, R. V. Jagadeesh, M. Beller, “Ultra-small cobalt nanoparticles from molecularly-defined Co–salen complexes for catalytic synthesis of amines” *Chem. Sci.* **2020**, *11*, 2973–2981.

[60] D. Lenoir, “The Application of Low-Valent Titanium Reagents in Organic Synthesis” *Synthesis* **2002**, *1989*, 883–897.

[61] J. Wu, S. Ma, J. Sun, J. I. Gold, C. S. Tiwary, B. Kim, L. Zhu, N. Chopra, I. N. Odeh, R. Vajtai, A. Z. Yu, R. Luo, J. Lou, G. Ding, P. J. A. Kenis, P. M. Ajayan, “A metal-free electrocatalyst for carbon dioxide reduction to multi-carbon hydrocarbons and oxygenates” *Nat. Commun.* **2016**, 7, 13869.

[62] H. Wang, J. Jia, P. Song, Q. Wang, D. Li, S. Min, C. Qian, L. Wang, Y. F. Li, C. Ma, T. Wu, J. Yuan, M. Antonietti, G. A. Ozin, “Efficient Electrocatalytic Reduction of CO_2 by Nitrogen-Doped Nanoporous Carbon-Carbon Nanotube Membranes - A Step Towards the Electrochemical CO_2 Refinery” *Angew. Chem. Int. Ed.* **2017**, *56*, 7847–7852.

[63] S. Lin, C. S. Diercks, Y.-B. Zhang, N. Kornienko, E. M. Nichols, Y. Zhao, A. R. Paris, D. Kim, P. Yang, O. M. Yaghi, C. J. Chang, “Covalent organic frameworks comprising cobalt porphyrins for catalytic CO_2 reduction in water” *Science* **2015**, 349,1208-1213.

[64] W. Sun, Z. Li, X. Meng, N. Li, X. Liang, Y. Ding, “Recent Advances and Perspectives on Porphyrin for Photocatalytic CO_2 Reduction: From Molecules to Framework Materials” *Chem. Rec.* **2025**, 25, 12, e202500101.

[65] C. Würth, M. Grabolle, J. Pauli, M. Spieles, U. Resch-Genger, “Relative and absolute determination of fluorescence quantum yields of transparent samples” *Nat. Protoc.* **2013**, *8*, 1535–1550.

[66] A. K. Nain, “Refractive Indices of Binary Mixtures of Tetrahydrofuran with Aromatic Hydrocarbon at Temperatures from (288.15 to 318.15) K” *J. Chem. Eng. Data* **2008**, *53*, 850–853.

[67] J. Ortega, “Densities and refractive indices of pure alcohols as a function of temperature” *J. Chem. Eng. Data* **1982**, *27*, 312–317.

[68] X. Jia, Y. Zhang, P. Zhang, Z.-X. Guo, “Catalytic oxidation of alcohols to aldehydes and ketones with ferric nitrate nonahydrate/trifluoroacetic acid *via* mechanochemical reaction” *Tetrahedron Lett.* **2024**, *142*, 155079.

[69] P. Pracht, S. Grimme, C. Bannwarth, F. Bohle, S. Ehlert, G. Feldmann, J. Gorges, M. Müller, T. Neudecker, C. Plett, S. Spicher, P. Steinbach, P. A. Wesołowski, F.

Zeller, "CREST—A program for the exploration of low-energy molecular chemical space" *J. Chem. Phys.* **2024**, *160*, 114110.

[70] S. Spicher, S. Grimme, "Robust Atomistic Modeling of Materials, Organometallic, and Biochemical Systems" *Angew. Chem. Int. Ed.* **2020**, *59*, 15665–15673.

[71] C. Bannwarth, S. Ehlert, S. Grimme, "GFN2-xTB—An Accurate and Broadly Parametrized Self-Consistent Tight-Binding Quantum Chemical Method with Multipole Electrostatics and Density-Dependent Dispersion Contributions" *J. Chem. Theory Comput.* **2019**, *15*, 1652–1671.

[72] C. Bannwarth, E. Caldeweyher, S. Ehlert, A. Hansen, P. Pracht, J. Seibert, S. Spicher, S. Grimme, "Extended tight-binding quantum chemistry methods" *WIREs Comput. Mol. Sci.* **2021**, *11*, e1493.

[73] S. Ehlert, M. Stahn, S. Spicher, S. Grimme, "Robust and Efficient Implicit Solvation Model for Fast Semiempirical Methods" *J. Chem. Theory Comput.* **2021**, *17*, 4250–4261.

[74] S. Grimme, J. G. Brandenburg, C. Bannwarth, A. Hansen, "Consistent structures and interactions by density functional theory with small atomic orbital basis sets" *J. Chem. Phys.* **2015**, *143*, 054107.

[75] A. R. McNeill, S. E. Bodman, A. M. Burney, C. D. Hughes, D. L. Crittenden, "Experimental Validation of a Computational Screening Approach to Predict Redox Potentials for a Diverse Variety of Redox-Active Organic Molecules" *J. Phys. Chem. C* **2020**, *124*, 24105–24114.

[76] A. Dmitrieva, J. J. Medvedev, X. V. Medvedeva, E. Krivoshapkina, A. Klinkova, "Synthesis of α-Amino Acids via Electrochemical Fixation of CO2 to Imines Using Different Metal Cathodes" *J. Electrochem. Soc.* **2023**, *170*, 075501.

[77] J. Koziskova, F. Hahn, J. Richter, J. Kožíšek, "Comparison of different absorption corrections on the model structure of tetrakis(μ_2 -acetato)-diaqua-di-copper(II)" *Acta Chim. Slovaca* **2016**, *9*, 136–140.

[78] G. M. Sheldrick, "SHELXT – Integrated space-group and crystal-structure determination" *Acta Crystallogr. Sect. Found. Adv.* **2015**, *71*, 3–8.

[79] G. M. Sheldrick, "Crystal structure refinement with SHELXL" *Acta Crystallogr. Sect. C Struct. Chem.* **2015**, *71*, 3–8.

[80] O. V. Dolomanov, L. J. Bourhis, R. J. Gildea, J. a. K. Howard, H. Puschmann, "OLEX2: a complete structure solution, refinement and analysis program" *J. Appl. Crystallogr.* **2009**, *42*, 339–341.
